# A Novel Combined Scientific and Artistic Approach for the Advanced Characterization of Interactomes: The Akirin/Subolesin Model

**DOI:** 10.3390/vaccines8010077

**Published:** 2020-02-08

**Authors:** Sara Artigas-Jerónimo, Juan J. Pastor Comín, Margarita Villar, Marinela Contreras, Pilar Alberdi, Israel León Viera, Leandro Soto, Raúl Cordero, James J. Valdés, Alejandro Cabezas-Cruz, Agustín Estrada-Peña, José de la Fuente

**Affiliations:** 1 SaBio. Instituto de Investigación en Recursos Cinegéticos IREC-CSIC-UCLM-JCCM, Ronda de Toledo s/n, 13005 Ciudad Real, Spain; sartigasjeronimo@gmail.com (S.A.-J.); margaritam.villar@uclm.es (M.V.); marinelacr@hotmail.com (M.C.); maria.alberdi@uclm.es (P.A.); 2 Centro de Investigación y Documentación Musical CIDoM-UCLM-CSIC, Facultad de Educación de Ciudad Real, Ronda Calatrava 3, 13071 Ciudad Real, Spain; juanjose.pastor@uclm.es; 3 León Viera Studio, Calle 60 No. 338 M por 31, Colonia Alcalá Martín, Mérida 97000, Mexico; leonvieraisrael@gmail.com; 4 Artesadhana Studio, Mérida 97000, Mexico; sotoananda@gmail.com; 5 Raúl Cordero Studio, Calle Rio Elba 21-8, Colonia Cuauhtémoc, CDMX 06500, Mexico; raulcorderostudio@gmail.com; 6 Faculty of Science, University of South Bohemia, 37005 České Budějovice, Czech Republic; valdjj@gmail.com; 7 Institute of Parasitology, Biology Centre, Czech Academy of Sciences, Branišovská 1160/31, 37005 České Budějovice, Czech Republic; 8 Department of Virology, Veterinary Research Institute, Hudcova 70, 62100 Brno, Czech Republic; 9 UMR BIPAR, INRA, ANSES, Ecole Nationale Vétérinaire d’Alfort, Université Paris-Est, Maisons-Alfort 94700, France; cabezasalejandrocruz@gmail.com; 10 Facultad de Veterinaria, Universidad de Zaragoza, 50013 Zaragoza, Spain; aestrada@unizar.es; 11 Department of Veterinary Pathobiology, Center for Veterinary Health Sciences, Oklahoma State University, Stillwater, OK 74078, USA

**Keywords:** akirin, subolesin, interactome, art, evolution, music, yeast two-hybrid, NF-κB, vaccine, quantum vaccinomics, protective epitope

## Abstract

The main objective of this study was to propose a novel methodology to approach challenges in molecular biology. Akirin/Subolesin (AKR/SUB) are vaccine protective antigens and are a model for the study of the interactome due to its conserved function in the regulation of different biological processes such as immunity and development throughout the metazoan. Herein, three visual artists and a music professor collaborated with scientists for the functional characterization of the AKR2 interactome in the regulation of the NF-κB pathway in human placenta cells. The results served as a methodological proof-of-concept to advance this research area. The results showed new perspectives on unexplored characteristics of AKR2 with functional implications. These results included protein dimerization, the physical interactions with different proteins simultaneously to regulate various biological processes defined by cell type-specific AKR–protein interactions, and how these interactions positively or negatively regulate the nuclear factor kappa-light-chain-enhancer of activated B cells (NF-κB) signaling pathway in a biological context-dependent manner. These results suggested that AKR2-interacting proteins might constitute suitable secondary transcription factors for cell- and stimulus-specific regulation of NF-κB. Musical perspective supported AKR/SUB evolutionary conservation in different species and provided new mechanistic insights into the AKR2 interactome. The combined scientific and artistic perspectives resulted in a multidisciplinary approach, advancing our knowledge on AKR/SUB interactome, and provided new insights into the function of AKR2–protein interactions in the regulation of the NF-κB pathway. Additionally, herein we proposed an algorithm for quantum vaccinomics by focusing on the model proteins AKR/SUB.

## 1. Introduction

Biology and art have collaborated before for communication in areas such as human physiology and evolution [1,2,3,4], even proposing that art may have biological basis [5]. As scientists, art reminds us of the power of curiosity, which frequently gets lost during research, and asks questions that are relevant for investigation, thus supporting the fact that science benefits when artists get involved in research [6,7]. Art visual and musical representations translate into complex or unrecognized concepts and provide a way to better understand and approach scientific challenges [8,9,10]. Art also contributes to highlighting social concerns addressed by scientists [4]. In this way, art and science converge in the purpose of moving society forward, and both benefit from the learning process [9,10,11].

In this study, we provided a perspective of the combined scientific and artistic approach to the interactome using as a model the regulatory protein Akirin, from the Japanese “akiraka ni suru” meaning “making things clear” (AKR; also known as Subolesin (SUB) in ticks). AKR/SUB were first identified in *Drosophila melanogaster*, *Mus musculus*, and *Ixodes scapularis* as transcription factors involved in the regulation of immune deficiency (IMD) and tumor necrosis factor (TNF)/Toll-like receptor (TLR)-nuclear factor kappa-light-chain-enhancer of activated B cells (NF-κB) (TNF/TLR) signaling pathways, and developmental processes [12,13,14,15]. Recent results have shown that AKR/SUB evolved with conserved sequence and structure, suggesting a functional role in cell interactome and regulome in response to pathogen infection and other stimuli [16,17,18]. Additionally, AKR/SUB have shown protection in vaccines for the control of multiple ectoparasite infestations and pathogen/infection transmission [17]. Therefore, AKR/SUB constitute a good model for the study of structural, functional, and evolutionary biology due to their conserved function in the regulation of different biological processes throughout the metazoan [16,17,18]. However, differences in results available on the AKR interactome and its functional implications pose a challenge for the understanding of the function of this protein [17,18,19,20,21,22,23,24,25,26,27,28]. For example, it is unknown how AKR/SUB-protein interactions regulate signaling pathways such as the NF-κB involved in the regulation of immune response.

To address this question, herein we proposed a novel methodology to approach challenges in molecular biology using as a model the characterization of the AKR2 interactome and its functional role in the regulation of the NF-κB signaling pathway in human placenta cells. The results of this approach advanced our knowledge in this area by showing that AKR/SUB protein dimerization/multimerization and the physical interactions with different proteins simultaneously are involved in the regulation of various biological processes defined by cell type-specific AKR2-protein interactions and the role of these interactions in the positive and negative regulation of the NF-κB pathway.

The characterization of protein–protein interactions and vaccinomics have been proposed as novel approaches for vaccine development [29]. Considering the structural and functional conservation of AKR/SUB proteins [17], the characterization of the AKR2 interactome may have implications in quantum vaccinomics as a new approach for the development of vaccines for the control of vector infestations and infection/transmission of vector-borne pathogens. In this context, quantum vaccinomics could be focused on cell interactome and regulome for the identification of protective epitopes in peptide sequences involved in protein–protein interactions or selected interacting domains (SID) that are particularly relevant for proteins such as AKR/SUB that function through these interactions.

## 2. Materials and Methods 

### 2.1. Approaching Collaboration between Visual and Musical Artists and Scientists

The methodological approach used in this study consisted of collaborations between artists and scientists to address questions in the molecular biology of interactomes using the regulatory factor AKR/SUB as a model. Three visual artists, Israel León Viera, Leandro Soto, and Raúl Cordero were selected for participating in this project. Israel León Viera and Leandro Soto participated in a leading art group known as “Volumen Uno” (Volume One) who challenged artistic establishment in Cuba in the 1980s, and have shown a continued interest in social, historical, and cultural subjects [30]. Raúl Cordero is an artist with an interest in science and technical challenges in visual art and communication [31]. These artists were invited to read a simplified version of our recent review on AKR/SUB functional evolution [17], with special attention paid to the figures included in the paper. The artists were posed with the challenge that these model proteins represent based on their conserved function in the regulation of different biological processes throughout the metazoan [16,17,18]. Artists and scientists were in contact to exchange information regarding these proteins and to address artists’ questions. In response to this challenge, the artists contributed the pieces and interpretations shown in the paper to provide their view on this matter. Then, these pieces served to inspire scientists to discuss and find new perspectives on unexplored characteristics of these proteins with putative functional implications. A professor of music and musical education was also invited to participate in this research to further develop and apply a musical algorithm developed by us back in 1995 to the study of AKR evolution [8]. The results of these musical representations served to inspire scientists to propose the possibility of using this approach to further characterize AKR–protein interactions, which was faced by the artist resulting in additional support for the results presented in the paper.

### 2.2. Yeast Two-Hybrid Y2H Screening for the Identification of Human AKR2 Interacting Proteins

The ULTImate Y2H screening was performed by Hybrigenics Services (Paris, France; www.hybrigenics-services.com) following previously described methods [32,33]. The human AKR2 (amino acids 1-203; Uniprot ID Q53H80) bait was PCR-amplified, sequenced, cloned in the pB27 (N-LexA-AKR2-C fusion) vector, and used for screening using a human placenta RP6 fragment library as prey. In the test screen, we found that the bait did not autoactivate the system, but it was inducing too much signal. Therefore, we used a medium containing 10 mM of 3-amino-1,2,4-triazole (*3-AT*), a competitive inhibitor of the *HIS3* reporter gene product. A total of 330 prey fragments of the positive clones were amplified by PCR and sequenced at their 5′ and 3′ junctions. The resulting sequences were used to identify the corresponding interacting proteins in the GenBank database (NCBI) using a fully automated procedure.

### 2.3. Interaction Confidence Scoring

A confidence score for Predicted Biological Score (PBS) was attributed to each interaction as previously described [33] (Data S1). The PBS score represents the probability of an interaction being non-specific and is computed to assess the interaction reliability. PBS is an e-value, primarily based on the comparison between the number of independent prey fragments found for an interaction and the chance of finding them at random (background noise). The value in the scores A to D varies between 0 and 1 (A < 1e^-10^ < B < 1e^-5^ < C < 1e^-2.5^ < D < 1). Several thresholds were arbitrarily defined in order to rank the results in the following scores:

- A: Very high confidence in the interaction.
- B: High confidence in the interaction.
- C: Good confidence in the interaction.
- D: Moderate confidence in the interaction. This category is the most difficult to interpret because it mixes two classes of interactions: (i) false-positive interactions and (ii) interactions hardly detectable by the Y2H technique (e.g., low representation of the mRNA in the library, prey folding, prey toxicity in yeast).
- E: Interactions involving highly connected (or relatively highly connected) prey domains, warning of non-specific interaction. The total number of screens performed on each organism is taken into account to set this connectivity threshold to 20 interactions to different bait proteins in the entire human database. They can be classified in different categories: (i) prey proteins that are known to be highly connected due to their biological function and (ii) proteins with a prey interacting domain that contains a known protein interaction motif or a biochemically promiscuous motif.
- F: Experimentally proven technical artifacts.
- N/A: The PBS is a score that is automatically computed through algorithms and cannot be attributed for the following reasons: (i) all the fragments of the same reference coding sequence (CDS) are antisense, (ii) the 5p sequence is missing, (iii) all the fragments of the same reference CDS are either all out of frame (OOF1 or OOF2), and (iv) all the fragments of the same reference CDS lie in the 5′ or 3′ untranslated region (UTR).

### 2.4. Annotation of Identified AKR2-Interacting Proteins

DomSight (Hybrigenics Services) was applied to compare the bait fragment and the selected interacting domain (SID) of the prey proteins with the functional and structural domains generated by Pfam (https://pfam.xfam.org), SMART (http://smart.embl-heidelberg.de), TMHMM (http://www.cbs.dtu.dk/services/TMHMM/), SignalP (http://www.cbs.dtu.dk/services/SignalP/), and COILS (https://embnet.vital-it.ch/software/COILS_form.html) algorithms (Figure S1). N/A annotations (Data S1) were not considered as interactions. Annotations for biological process and subcellular localization were performed according to Uniprot (https://www.uniprot.org; searched on October 2018). Comparison with previously published datasets for AKR2-interacting proteins were performed using datasets for human AKR2 (http://www.humanmine.org/humanmine/portal.do?externalids=55122&class=Gene&origin=FlyMine) [19,20,21,22,23]. For validation, AKR2–protein interactions were searched in the public databases STRING (https://string-db.org/cgi/input.pl?sessionId=BIqHpYhivYPx&input_page_show_search=on) and NCBI (https://www.ncbi.nlm.nih.gov/gene?Db=gene&Cmd=DetailsSearch&Term=55122) reporting interactions using alternative interactome models such as ingenuity pathway analysis (IPA).

### 2.5. Corroboration of AKR2–Protein Interactions by Protein Pull-Down

First, the human AKR2-protein interactions identified by Y2H with very high (A-score) and high (B-score) PBS were used to corroborate these interactions in vitro. Recombinant human (Myc (EQKLISEEDL)-DDK-tagged) AKR2 (Q53H80; RC200881, OriGene, Rockville, MD, USA) was used for interactions with the identified AKR interacting recombinant proteins AKR2, actin related protein 10 (ACTR10; Q9NZ32; H00055860-P01, Novus Biologicals, Littleton, CO, USA), estrogen-related receptor gamma (ESRRG; P62508; ab152371, Abcam, Cambridge, United Kingdom), phosphatidylinositol transfer protein alpha isoform (PITPNA; Q00169; ab101666, Abcam), RING finger protein 10 (RNF10; Q8N5U6; H00009921-P01, Novus Biologicals), splicing factor 3a subunit 1 (SF3A1; Q15459; ab160931, Abcam), and mediator of RNA polymerase II transcription subunit 16 (THRAP5/MED16; Q9Y2 × 0; ab132365, Abcam). Additionally, interferon regulatory factor 6 (IRF6; O14896; ab132057, Abcam) and protein Wnt-2 (WNT2; P09544; ab152803, Abcam) with moderate confidence (D-score) PBS were included in the analysis. The U1 small nuclear ribonucleoprotein 70 kDa (SNRNP70; P08621; H00006625-P01, Abnova GmbH, Heidelberg, Germany) with score F (experimentally proven technical artifact) was included as a negative control. Interacting proteins were incubated with AKR2 for 2 h and then for 30 min with c-Myc magnetic beads (c-Myc-Tag IP/Co-IP kit, Thermo Fisher Scientific, Waltham, MA, USA) specific for the AKR2 protein tag following the manufacturer’s recommendations. A negative control was also performed, incubating only the interacting proteins with the c-Myc magnetic beads. Both incubations were performed with shaking at room temperature (RT). After immunoprecipitation, protein complexes were eluted using Laemmli sample buffer, and the supernatants were used for Western blot analyses. Supernatants derived from AKR2–protein interactions and negative controls, together with positive control recombinant protein were run and separated by electrophoresis in a 12% sodium dodecyl sulfate (SDS) polyacrylamide precast gel (ClearPage, Cole-Parmer, Vermon Hills, IL, USA) and stained with Bio-Safe Coomassie Stain (Bio-Rad Laboratories, Hercules, CA, USA) or transferred to a nitrocellulose blotting membrane (GE Healthcare Life Sciences, Pittsburgh, PA, USA). The membrane was blocked with 3% bovine serum albumin (BSA) (Sigma-Aldrich, St. Louis, MO, USA) in Tris-buffered saline (TBS; 150 mM NaCl, 50 mM Tris-HCl, pH 7.5) for 2 h at RT and washed four times with TBS-Tween 20 (150 mM NaCl, 50 mM Tris-Cl, pH 7.5, 0.05% Tween 20). Different interacting protein-specific primary rabbit or mouse antibodies were used for ACTR10 (mouse H00055860-B01P, Novus Biologicals, 1:500), ESRRG (mouse ab171816, Abcam, 1 µg/mL), PITPNA (rabbit ab96519, Abcam, 1:500), RNF10 (mouse H00009921-B01P, Novus Biologicals, 1:500), SF3A1 (rabbit ab69903, Abcam, 0.25 µg/mL), THRAP5/MED16 (rabbit ab130996, Abcam, 1:2000), SNRNP70 (rabbit VPA00459, Bio-Rad Laboratories, 1:1000), IRF6 (mouse ab123880, Abcam, 1:1000), AKR2 (rabbit ab221475, Abcam, 1:1000), and WNT2/IRP (rabbit ab27794, Abcam, 2µg/mL). Antibodies were diluted in TBS and incubated with membranes overnight at 4 °C. Goat anti-mouse or anti-rabbit immunoglobulin (IgG) (whole molecule) peroxidase antibody (Sigma-Aldrich) 1:1000 diluted in TBS with 3% BSA were used as secondary antibodies and incubated with membranes for 2 h at RT. The membranes were finally washed five times with TBS-Tween 20, and immunoreactive proteins were visualized with chemiluminescence by incubating the membranes for 2 min with Pierce ECL Western blotting substrate (Thermo Fisher Scientific). In order to identify unrelated proteins eluted from the c-Myc magnetic beads, a similar experiment was conducted with selected proteins ESRRG and RNF10 but incubating only with the secondary antibody. Additionally, c-Myc magnetic beads alone were eluted using Laemmli sample buffer and the supernatant analyzed by SDS-PAGE stained with Bio-Safe Coomassie Stain.

### 2.6. In Vitro Characterization of AKR–AKR Protein Interactions

Recombinant *I. scapularis* AKR/SUB (Q4VRW2) and B7PDL0 negative control proteins were produced in *Escherichia coli* using the Champion pET101 Directional TOPO Expression kit (Invitrogen, Carlsbad, CA, USA), as previously described [34,35]. These proteins and human AKR2 (Q53H80) were used for the in vitro characterization of protein–protein interactions. For interactions, 5 µg of each protein was incubated in 20 µl PBS with shaking at 4 °C overnight. Then, the proteins were analyzed by electrophoresis under non-denaturing conditions in 12% polyacrylamide precast gels (Bio-Rad Laboratories, Inc., Hercules, CA, USA) and stained with Coomassie-blue. Precision Plus Protein Standard (Bio-Rad Laboratories, Inc.) was used as molecular weight marker. For Western blot analysis, 10 μg of the interactions (5 µg of each interacting protein) were separated as before, and were transferred to a nitrocellulose membrane. The membrane was blocked with 5% BSA (Sigma-Aldrich) for 2 h at RT and washed three times with TBS (50 mM Tris-HCl, pH 7.5, 150 mM NaCl, 0.5% Tween 20). Rabbit IgG against tick SUB were used as primary antibodies at a 1:300 dilution in TBS, and the membrane was incubated overnight at 4 °C and washed three times with TBS. The membrane was then incubated with an anti-rabbit IgG-horseradish peroxidase (HRP) conjugate (Sigma-Aldrich) diluted 1:1000 in TBS with 3% BSA. The membrane was washed four times with TBS and finally developed with TMB (3,3′, 5,5′-tetramethylbenzidine)-stabilized substrate for HRP (Promega, Madrid, Spain) according to the manufacturer’s recommendations.

Size exclusion chromatography (SEC) was performed to provide additional support for AKR2–AKR2 and SUB–SUB interactions. The SEC experiment was conducted using the GE AKTA Prime Plus FPLC System (GE Healthcare Life Sciences, Chicago, IL, USA) and the HiPrep 16/60 Sephacryl S-100 HR column (GE Healthcare Life Sciences, 17-1165-01) following the manufacturer’s recommendations for size exclusion chromatography (https://www.sigmaaldrich.com/content/dam/sigma-aldrich/docs/Sigma-Aldrich/General_Information/1/ge-size-exclusion-chromatography.pdf). A calibration curve was prepared using the gel filtration calibration kit (GE Healthcare Life Sciences) with conalbumin (C; 75 kDa), ovalbumin (O; 44 kDa), carbonic anhydrase (CA; 29 kDa), ribonuclease A (R; 13.7 kDa), and aprotinin (Apr; 6.5 kDa) proteins to calculate the partition coefficient (Kav) following the manufacturer’s protocol (Kav = Ve − Vo/Vc – Vo, where Ve is the determined elution volume, Vo is the void volume = 36 mL, and Vc is the geometric column volume = 120 mL). The equation of the resulting calibration curve (*R^2^* = 0.97) was protein molecular weight (MW) = 166086e^−3.377Kav^. Individual proteins were injected separately for calibration in 0.5 mL sample injection volume (140 ng/mL protein concentration in 50 mM sodium phosphate, 150 mM sodium chloride, pH 7.2) at a flowrate of 0.8 mL/min. Recombinant AKR2 (30 kDa) and SUB (28 kDa) were incubated in PBS overnight at 4 °C before dilution in the column buffer for analysis.

### 2.7. Prediction Models of Protein–Protein Binding Sites and Structure

The proteins with a PBS of A, THRAP5 (Q9Y2X0), and RNF10 (Q8N5U6) were selected to model interactions with AKR2 (Q53H80). Human interferon alpha 1 (IFN-a1; P01562) was used as a negative control protein. For human AKR2 and *I. scapularis* tick, AKR/SUB (Q4VRW2), AKR2-AKR2, and SUB–SUB interactions were also modeled. Predictions were made using the iFrag server (http://sbi.imim.es/web/index.php/research/servers/iFrag?) [36] at default settings except for e-value ≥ 10 and identities ≥ 10. Protein structure models were obtained from The Protein Model Portal for RNF10 (https://www.proteinmodelportal.org/query/uniprot/Q8N5U6) and THRAP5 (https://www.proteinmodelportal.org/query/uniprot/Q9Y2X0).

### 2.8. Musical Scores and Ensembles

The algorithm used to translate DNA coding sequences into music was as previously reported [8], except that each codon was metrically equivalent to a bar and had a differentiated rhythmic and melodic character. The modified algorithm provides for each codon either a constant base of a note on which one or two more notes may follow, or a base of two musical notes followed by one or two more. We provided a ternary structure (three beats of a quarter note) and a binary subdivision measure (3/4), where the base of a single note occupies the entire measure (UGC = T, dotted-half-note (underlined base)), and the base of two notes was expressed as a half-plus-quarter-note (CAA = MF). If the base is a single note accompanied by others, we always chose to extend the base in the first two beats of the measure as half-note when it had the succession of a single note (UCG = SR half-plus-quarter-note) or two notes (UCC = SMR, half-plus-two-eighth notes). If the base was of two notes, we established for its expression a different metrical scheme: dotted-quarter-note-plus-eighth-note followed by a quarter-note (GCG = DRR), or by two eighth-notes (GCC = DRMR). When the algorithm provided the same melodic formula for a double base (GGA = SL; GGC = SLDR) and for a single base (UCA = S; UCU = SL), we opted for the metric scheme half-plus-quarter-note in the first case and dotted-quarter-note-plus-dotted-quarter-note in the second case. In this way, each codon had a unique rhythmic and melodic definition. All sound files and scores were prepared using the Finale (v. 2018) program (https://www.finalemusic.com). Finding the best match by trial and error produced musical ensembles. Audio files were uploaded and can be found at https://freesound.org/people/josedelafuente/sounds/478998 to 479009.

### 2.9. NF-κB Reporter Assay

The NF-κB reporter kit (BPS Bioscience, San Diego, CA, USA) was used following the manufacturer’s recommendations for monitoring the activity of the NF-κB signaling pathway in human Hs795.PI (ATCC CRL-7526) placenta cultured cells maintained in Dulbecco’s Modified Eagle’s Medium (DMEM) medium supplemented with 10% fetal bovine serum (Thermo Fisher Scientific). The reporter contained a firefly luciferase gene under the control of multimerized NF-κB-responsive element located upstream of a minimal promoter. The NF-κB reporter and a non-inducible firefly luciferase vector were premixed with constitutively expressing *Renilla* luciferase vector, which served as an internal control for transfection efficiency. To obtain the normalized luciferase activity for NF-κB reporter, the background luminescence was subtracted and then the ratio of firefly luminescence from the NF-κB reporter to *Renilla* luciferase vector control was calculated and used for analysis.

### 2.10. Gene Knockdown by RNA Interference (RNAi)

The ON-TARGETplus Human Gene SMARTpool small interfering RNAs (siRNAs) (*n* = 4 per gene) were designed and synthesized by Dharmacon, Inc. (Lafayette, CO, USA) for *AKR2* and the interacting protein coding genes *AKR1*, *RNF10*, *WNT2*, *IRF6*, *THRAP5*, and *ESRRG*. Transfection of human placenta-cultured cells with siRNAs for single or combined gene targets was performed for 24 h. Combined siRNAs for *NF-κB1* (p50) and *NF-κB2* (p52) (*NF-κB1/B2*) and Accell Green Non-Targeting siRNAs (Dharmacon, Inc.) were used as positive and negative control, respectively. Each gene knockdown experiment was performed in both human placenta cells transfected with the NF-κB luciferase reporter vector and the non-inducible luciferase vector by incubating cells with 25 nM of siRNAs diluted in serum-free medium and Lipofectamine 300 (Invitrogen) in 96-well plates using 6 wells per treatment and following the manufacturer’s recommendations. After 24 h, the Dual Luciferase (Firefly-*Renilla*) Assay System (BPS Bioscience) was performed following the manufacturer’s protocol. Luciferase luminescence lecture (kinetic duration 15 s, interval time 2 s, integration time 1 s) was determined using a Tecan Infinite M200 lector (Mannedorf, Switzerland). Background luminescence was subtracted and then the ratio of firefly luminescence from the NF-κB reporter to *Renilla* luciferase vector control was calculated and used for analysis. The normalized luciferase activity for NF-κB reporter was compared between groups by Student’s *t*-test with unequal variance and by one-way ANOVA (https://www.socscistatistics.com/tests/anova/default2.aspx) (*p* = 0.05; *n* = 6 biological replicates). After luciferase detection, cells were collected for RNA extraction for analysis of mRNA levels by qRT-PCR. Normalized Ct values were compared between test siRNA-treated placenta cells and controls treated with non-targeting siRNA by Student's *t*-test with unequal variance (*p* = 0.05; *n* = 6).

### 2.11. Treatment of Human Placenta Cells with Lipopolysaccharides (LPS)

Human placenta cells were treated with 10 µg/mL of LPS from *Salmonella enterica* serotype typhimurium (L6143; Sigma-Aldrich) in a 24-well plate using 6 wells per treatment. LPS-treated and PBS-treated control cells were harvested after 24 h of treatment. Total RNA was extracted from placenta cells using TriReagent (Sigma-Aldrich) following the manufacturer’s recommendations and was used to characterize the mRNA levels of *AKR2*, *AKR1*, *ESRRG*, *RNF10*, *THRAP5*, *IRF6*, *WNT2*, *IFN-β*, and *IL-6* genes by qRT-PCR. Normalized LPS- to PBS-treated control (C-) Ct values were calculated, and normalized Ct values were compared between LPS-treated and C-cells by chi^2^ test (* *p* = 0.01; *n* = 6 biological replicates).

### 2.12. Analysis of Gene Expression by qRT-PCR

Total RNA was extracted from placenta cells using TriReagent (Sigma-Aldrich) following the manufacturer’s recommendations, and was used to characterize the mRNA levels of selected genes by qRT-PCR using gene-specific oligonucleotide forward (F) and reverse (R) primers (*AKR2*, F: 5′-CGGAGCCACTCTGAAAAGGA-3′, R: 5′-GAGATACTTCTGCGGCGAGG-3′; *AKR1*, F: 5′-CCCTCCGACAAGTTGGCATA-3′, R: 5′-TAGCTTGTTGGCCTTGTCCC-3′; *RNF10*, F: 5′-GCTGGAGTATCTGTCTGCCT-3′, R: 5′-TCAGTGCAAATGGTCCCCTC-3′; *THRAP5*, F: 5′-CTGACCCGCATGATCCACAT-3′, R: 5′-CTATTAGCCAGGTGGTCCGC-3′; *ESRRG*, F: 5′-CAGCCAGCCAAAAAGCCATAT-3′, R: 5′-TATGCTTCGCCCATCCAATGA-3′; *IRF6*, F: 5′-GCTCATCTGGCTACACAGGG-3′, R: 5′-AGCTGGGCCTTCCATTTAGC-3′; *WNT2*, F: 5′-AACCAGGATGGCACAGGTTTC-3′, R: 5′-CCTCTCCCACAGCACATGAC-3′; *interferon beta IFN-β*, P01574, F: 5′-CGCCGCAGTGACCATCTAT-3′, R: 5′-TCATGCGTTTTCCCCTGGTG-3′; *NF-κB1*, P19838, F: 5′-AATGGGCTACACCGAAGCAA -3′, R: 5′-CTGTCGCAGACACTGTCACT-3′; *NF-κB2*, Q00653, F: 5′ GCGTTGTCAACCTCACCAAC -3′, R: 5′-GAGTCTCCATGCCGATCCAG-3′; interleukin-6 (*IL-6)*, P05231, F: 5’ CCTGAGAAAGGAGACATGTAACAAGA-3’, R: 5’ GGCAAGTCTCCTCATTGAATCC3′), using the Kapa SYBR Fast One-Step qRT-PCR Kit (Sigma-Aldrich) and the Rotor-Gene Real-Time PCR Detection System (Qiagen, Hilden, Germany). A dissociation curve was run at the end of the reaction to ensure that only one amplicon was formed and that the amplicons denatured consistently in the same temperature range for every sample. The mRNA levels were normalized against human *β-actin* (F: 5′-CTCGCCTTTGCCGATCC-3; R: 5′-CGCCCACATAGGAATCCTTC-3′) using the genNorm Delta-Delta-Ct (ddCt) method as described previously [37].

### 2.13. Protein Transfection

After 24 h of NF-κB reporter transfection as described above, human placenta cells were transfected with 0.5 ng proteins per well of AKR2, RNF10, WNT2, IRF6, and combinations with AKR2 using the Pierce Protein Transfection Reagent Kit (Thermo Scientific). Proteins were diluted in HEPES (4-(2-hydroxyethyl)-1-piperazineethanesulfonic acid) buffer (10 mM HEPES, 150 mM NaCl, pH 7.0) and used as solvent for transfection reagent. Transfection reagent/protein complexes were resuspended in serum-free medium and delivered to the cells. After 4 h of incubation at 37 °C, one volume of 20% serum-containing medium was added directly to the wells. As a positive control, 0.25 µg per well of fluorescein isothiocyanate (FITC)-antibody was transfected following the same protocol. Negative control cells were transfected with 0.5 ng ESRRG protein. After 24 h, the Dual Luciferase (Firefly-*Renilla*) Assay System (BPS Bioscience) was performed following the manufacturer’s protocol. Luciferase luminescence lecture (kinetic duration 15 s, interval time 2 s, integration time 1 s) was determined by a Tecan Infinite M200 lector. Background luminescence was subtracted and then the ratio of firefly luminescence from the NF-κB reporter to *Renilla* luciferase vector control was calculated and used for analysis. The normalized luciferase activity for NF-κB reporter was compared between groups by Student’s *t*-test with unequal variance (*p* = 0.05; *n* = 4 biological replicates). Protein transfection was confirmed in positive control-transfected cells and in comparison with untreated negative control cells by fluorescence microscopy. Cells were mounted in ProLong Antifade with 4’-6-diamidino-2-phenylindole (DAPI) reagent (Molecular Probes, Eugene, OR, USA) and examined using a Zeiss LSM 800 laser scanning confocal microscope (Carl Zeiss, Oberkochen, Germany) with a 63× oil immersion objective.

### 2.14. Prediction and Characterization of AKR/SUB Protective Epitopes

The identification and prediction of protective linear B-cell epitopes, mimotopes, and conformational discontinuous epitopes were previously described using overlapping oligopeptides scan (pepscan), solution-phase panning with affinity bead capture, and phage-display screening [38]. Protein disorder regions and three-dimensional models were predicted using Disopred [39], Robetta server [40], I-TASSER [41], Lomets [42], Qmean server [43], and FirstGlance in Jmol (http://firstglance.jmol.org), as previously reported [17,38]. The design, production, and characterization of Q38 and Q41 chimeric antigens based on AKR/SUB protective linear B-cell, mimotopes, and conformational discontinuous epitopes was reported by Moreno-Cid et al. [34]. Protein sequence alignment was performed with human AKR2 (Q53H80), human AKR1 (Q9H9L7), and *Ixodes scapularis* SUB (Q4VRW2) using the constraint-based multiple alignment tool (COBALT; https://www.ncbi.nlm.nih.gov/tools/cobalt/cobalt.cgi?LINK_LOC=BlastHomeLink).

## 3. Results

### 3.1. Artist New Perspectives AKR Dimerization and Multiple Simultaneous AKR2–Protein Interactions with Functional Implications

The objective of this study was to provide a multidisciplinary approach complementing scientific and artistic perspectives to address a challenging biological question posed by the functionality of multiple AKR2–protein interactions. Initially, two visual artists were invited to read a simplified version of our recent review on AKR/SUB functional evolution [17]. The artists were posed with the challenge that these model proteins represent based on their conserved function [16,17,18]. In response to this challenge, the artists provided their perspectives on AKR/SUB structure and function. Then, these pieces served to inspire scientists to discuss and find new perspectives on unexplored characteristics of AKR/SUB interactome with putative functional implications. To address this question, a Y2H screening was performed with the human AKR2 as a LexA-bait fusion in a human placenta cell library, and results were corroborated by in vitro studies and modeling. A professor of music and musical education was also invited to participate in this study to further develop and apply a musical algorithm proposed by us previously to AKR/SUB [8]. The results of these musical representations also led scientists to propose the possibility of using this approach to further characterize AKR–protein functional interactions. Finally, a third visual artist was invited to further contribute to the functional characterization of AKR2–protein interactions in the regulation of the NF-κB signaling pathway.

**Figure 1:**
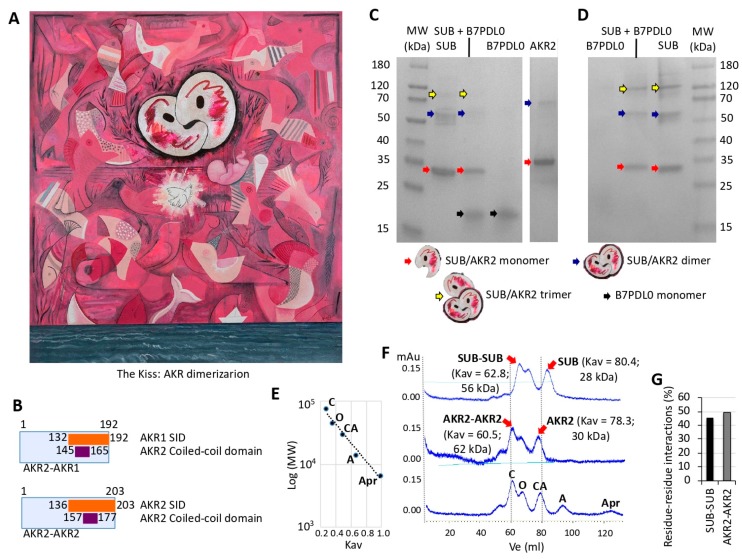
Akirin (AKR) dimerization/multimerization. (**A**) The piece “El Beso” (The Kiss) by Israel León Viera (mixed media on canvas, 2018, 150 × 133 cm; dedicated to his daughter Sofía León Jorge; courtesy KGJ Collection, Spain) represents protein–protein interactions that play a key role in AKR function, and a new and unexplored facet of possible AKR dimerization. (**B**) Protein interacting regions identified by Y2H. SID (selected interaction domain) is the amino acid sequence shared by all prey fragments matching the same reference protein, which have been found to contain structural or functional domains. Only regions containing bait fragments, SIDs, or predicted functional and structural domains were considered. (**C**,**D**) In vitro characterization of AKR–AKR protein interactions using *Ixodes scapularis* SUB (Subolesin; Q4VRW2), human AKR2 (Q53H80), and *I. scapularis* B7PDL0 negative control. SUB, AKR2, and B7PDL0 were incubated alone and SUB also in combination with B7PDL0 (SUB + B7PDL0), followed by analysis in (**C**) polyacrylamide gels or (**D**) Western blot using anti-SUB polyclonal IgG antibodies. (**E**) Protein gel filtration calibration curve (*R^2^* = 0.97) using conalbumin (C; 75 kDa), ovalbumin (O; 44 kDa), carbonic anhydrase (CA; 29 kDa), ribonuclease A (R; 13.7 kDa), and aprotinin (Apr; 6.5 kDa). (**F**) Size exclusion chromatography (SEC) analysis of AKR2 and SUB. The milli absorbance units (mAu) and elution volume (Ve) are shown. The partition coefficient (Kav) and molecular weight (MW) of AKR2/SUB proteins’ monomer and dimer were calculated using the equations derived from the calibration curve, Kav = Ve − Vo/Vc − Vo and MW = 166086e^−3.377Kav^. (**G**) Predicted model of the percentage of amino acids involved in residue–residue interactions for tick SUB–SUB and human AKR2–AKR2 interactions. Predictions were made using the iFrag server (http://sbi.imim.es/web/index.php/research/servers/iFrag?).

In the piece “El Beso” (The Kiss) (Figure 1A), the artist represented protein–protein interactions that appear to play a key role in the evolution and function of AKR/SUB regulatory factors [17]. According to artist’s statement, “this piece talks about the origins of life, with multiple geometric images that interact to illustrate in different species the conserved function of these proteins in biological processes represented by the sea, paper boat, Picasso’s dove, and a fetus growing in mother’s womb while opening the eyes to the world”. However, from a scientist’s perspective, a new and unexplored facet of possible AKR dimerization was also proposed in this piece (Figure 1A). This structural conformation and its functional implications have not been investigated before. Herein, the results from the Y2H experiment supported this prediction by showing AKR2–AKR2 and AKR2–AKR1 interactions and confirmed previous reports for AKR2–AKR1 interactions [23] (Figure 1B and Figure S1, Table 1, and Data S1). The SID of AKR1 (aa 132–192) binds to the coiled-coil domain of AKR2 (aa 145–165) and the SID of AKR2 (aa 136–203) binds to the coiled-coil domain of AKR2 (aa 157–177) (Figure 1B). Additionally, in vitro experiments using PAGE and Western blot (Figure 1C,D) or SEC (Figure 1F) and predictive models (Figure 1G) supported AKR–AKR interactions resulting in protein dimerization/multimerization that require attention.

**Figure 2:**
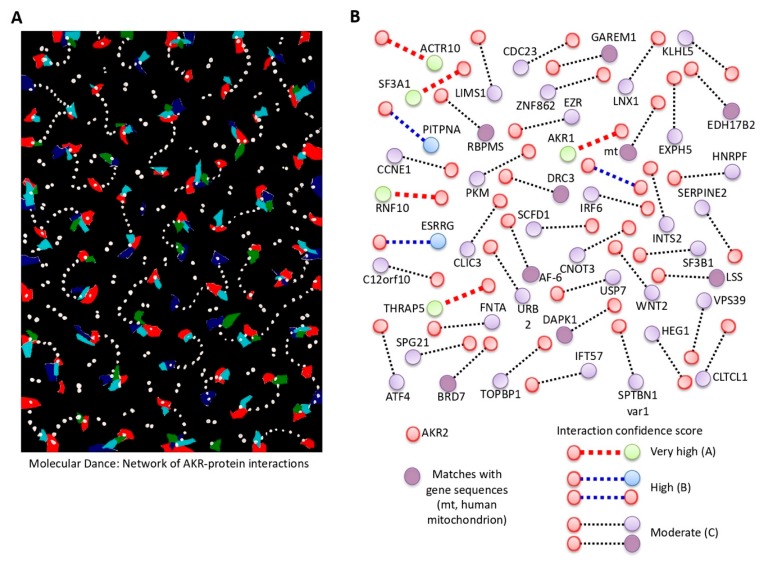
Human AKR2–protein interactions. (**A**) The piece “La Danza Molecular” (Molecular Dance) by Leandro Soto (mixed media on paper, 2018, 51 × 71 cm; courtesy KGJ Collection, Spain) also represents protein–protein interactions, and the possibility that AKR/SUB physically interacts with different proteins simultaneously to regulate various biological processes defined by tissue/cell-specific protein–protein interactions. (**B**) Results of the Y2H analysis of human AKR2 interactions in human placenta cells. Only proteins identified with A–D scores as potential candidates for interactions with AKR2 are shown. The full description of the proteins is shown in Table 1, Figure S1, and Data S1.

The piece “La Danza Molecular” (Molecular Dance) (Figure 2A) also represents protein–protein interactions, which the artist describes according to his words, “the constant movement of these proteins translated into the visual rhythm of repetitive interconnected interactions of forms and colors”. Again, in this case the artist suggested a new structural and functional component of AKR/SUB, which from a scientist’s perspective translates into the possibility that AKR/SUB physically interacts with different proteins simultaneously to regulate various biological processes defined by cell-specific AKR–protein interactions (Figure 2A).

**Figure 3:**
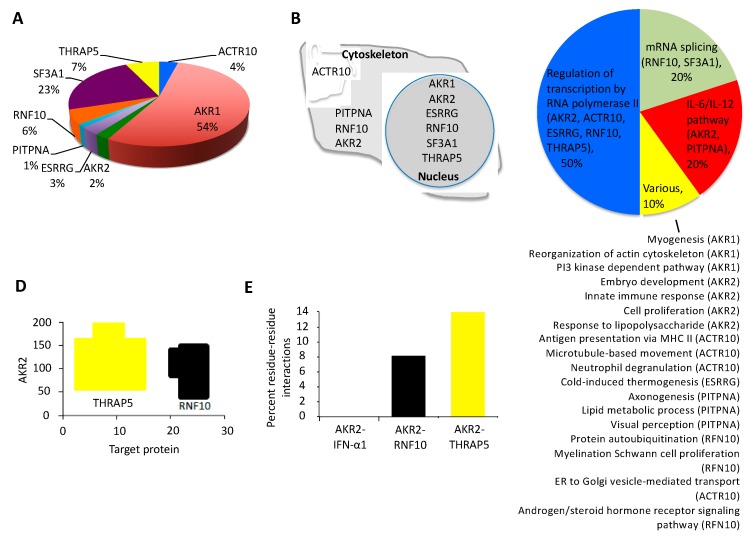
Characterization of human AKR2–protein interactions with highest confidence. (**A**) Representation of the percentage of total interactions after Y2H by proteins with very high and high confidence of interactions with human AKR2 in human placenta cells. (**B**) Subcellular localization of AKR2-interacting proteins. (**C**) Biological processes associated with AKR2-interacting proteins. (**D**) Prediction of specific interacting residues of human AKR2 with THRAP5 (Q9Y2X0) and RNF10 (Q8N5U6) proteins. The interacting residues formed by AKR2 dimer (amino acids 59-159) are not shown. (**E**) Predicted model of the percentage of amino acids involved in residue–residue interactions for human AKR2–interferon alpha 1 (IFN-a1) (negative control), AKR2–RNF10, and AKR2–THRAP5. Predictions in (**D,E**) were made using the iFrag server (http://sbi.imim.es/web/index.php/research/servers/iFrag?), and protein structure models were obtained from The Protein Model Portal.

In the Y2H screening used in this study, we did not detect any toxicity, and more than 77 million interactions were tested, which corresponds to a high library coverage (approximately sevenfold the complexity of the library). The results confirmed that the bait was well produced and folded in yeast. A total of 330 prey fragments of the positive clones were amplified by PCR and sequenced at their 5′ and 3′ junctions (Data S1 and Figure S1). Of them, 47 proteins were identified with A–D scores as candidates for interaction with human AKR2 (Figure 2B and Table 1). Focusing on the proteins with very high (A-score) and high (B-score) confidence of interactions, eight interacting proteins were identified (Figure 3A and Table 1). Among the identified prey proteins, AKR1 and AKR2 represented 56% of the interactions (Figure 3A). The AKR2-interacting proteins were annotated as located in the cytoskeleton, cytoplasm, and nucleus (Figure 3B), suggesting that interactions with AKR2 may occur not only in the nucleus but also in other cell compartments. Functionally, these proteins were annotated as involved in multiple biological processes (Figure 3C). The regulation of transcription by RNA polymerase II, mRNA splicing, and interleukin (IL)-6/IL-12 pathways were the most represented processes, supporting AKR2 role in the regulation of transcription and immune response among other biological processes [17]. To further validate the interaction of these proteins with AKR2 (Figure S1), prediction models illustrated the interacting regions (Figure 3D) and the percent of residue–residue interactions for selected identified proteins but not for the human interferon alpha 1 (IFN-α1) used as negative control (Figure 3E).

**Figure 4:**
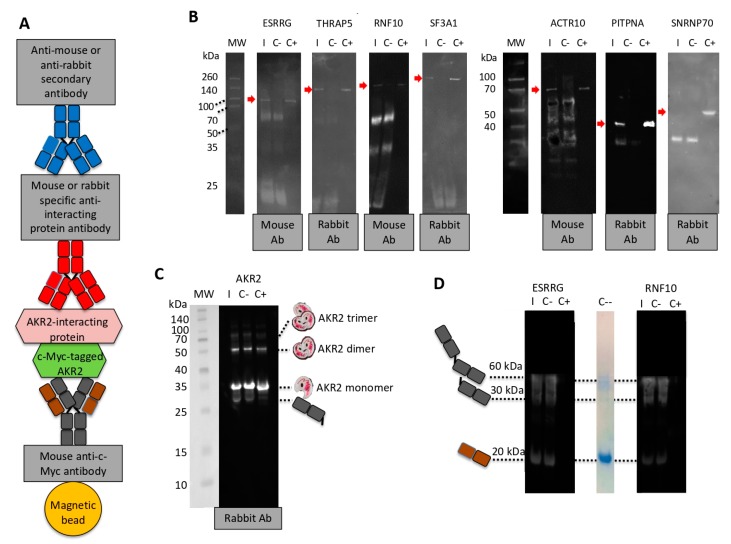
Corroboration of human AKR2–protein interactions with highest confidence. (**A**) Representation of the components of the protein pull-down experiment using c-Myc magnetic beads with Myc-tagged human AKR2. (**B**) Western blot analysis of AKR2–protein interactions. Interacting proteins were incubated with AKR2 and immunopresipitated with c-Myc magnetic beads specific for the AKR2 protein tag (I) or with the c-Myc magnetic beads only as negative control (C-). Recombinant interacting proteins were included as positive control (C+). Mouse or rabbit antibodies specific for each protein were used as primary antibodies and then anti-mouse or anti-rabbit secondary antibodies were used to identify the presence of the interacting proteins (red arrow). The origin of the primary antibody is shown. (**C**) Corroboration of human AKR2–AKR2 interaction using protein pull-down with c-Myc magnetic beads with human Myc-tagged AKR2. The experiment was conducted as described in (**B**). The origin of the primary antibody and predicted size for AKR2 monomer, dimer, and trimer together with bands corresponding to fragments of the mouse anti-c-Myc antibody attached to magnetic beads are shown. (**D**) In order to identify unrelated proteins eluted from the c-Myc magnetic beads, a similar experiment was conducted with selected proteins but incubating only with the secondary antibody. A lane of SDS-PAGE stained with Bio-Safe Coomassie Stain corresponding to c-Myc magnetic beads alone eluted using Laemmli sample buffer was included (C--). The bands corresponding to fragments of the mouse anti-c-Myc antibody attached to magnetic beads are shown.

Finally, AKR2–protein interactions were confirmed in vitro with recombinant proteins for identified prey and including the SNRNP70 protein with score F (experimentally proven technical artifact) as negative control (Figure 4A–D). A protein pull-down approach was used with c-Myc magnetic beads specific for the AKR2 protein tag and followed by immunoprecipitation of AKR2-interacting recombinant proteins with antibodies specific for the interacting proteins (Figure 4A). The results showed that all AKR2-interacting protein bands were identified in the interaction (I) and with a similar size to the positive control (C+), except for the interactions with the c-Myc magnetic beads only (C-) and the non-interacting SNRNP70 negative control (Figure 4B,C). However, other protein bands were observed in both (I) and (C-) lanes at lower molecular weight than the AKR2-interacting proteins (Figure 4B,C). In order to identify these unrelated proteins eluted from the c-Myc magnetic beads, a similar experiment was conducted with selected proteins but incubating only with the secondary antibody. The results suggested that these bands corresponded to fragments of the mouse anti-c-Myc antibody attached to magnetic beads, which was further supported by the fact that these bands were more evident when using mouse-derived anti-interacting protein antibodies and the mouse secondary antibody with higher reactivity to the mouse anti-c-Myc antibody attached to magnetic beads (Figure 4A,D). Finally, this experimental approach provided additional support for AKR2–AKR2 interactions by identifying not only the AKR2 monomer, but also protein dimer and trimer (Figure 4C).

Twenty-four AKR2-protein physical interactions have been described before in human kidney, colon, lung, and mammary epithelial cells [19,20,21,22,23]. Of them, only three proteins were among the identified proteins in human placenta with very high (A; AKR1) or moderate (D; maspardin (SPG21) and E3 ubiquitin-protein ligase LNX (LNX1)) interaction scores (Figure 2B, Table 1, and Data S1). In STRING and NCBI databases of AKR2–protein interactions using Y2H and other alternative interactome models such as IPA, the interactions of AKR2 with AKR1, RNF10, zinc finger protein 862 (ZNF862), LNX1, and SPG21 were identified.

### 3.2. The Sound of the AKR/SUB Coding Sequence Supported Evolutionary Conservation and Functional Protein Interactions

**Figure 5:**
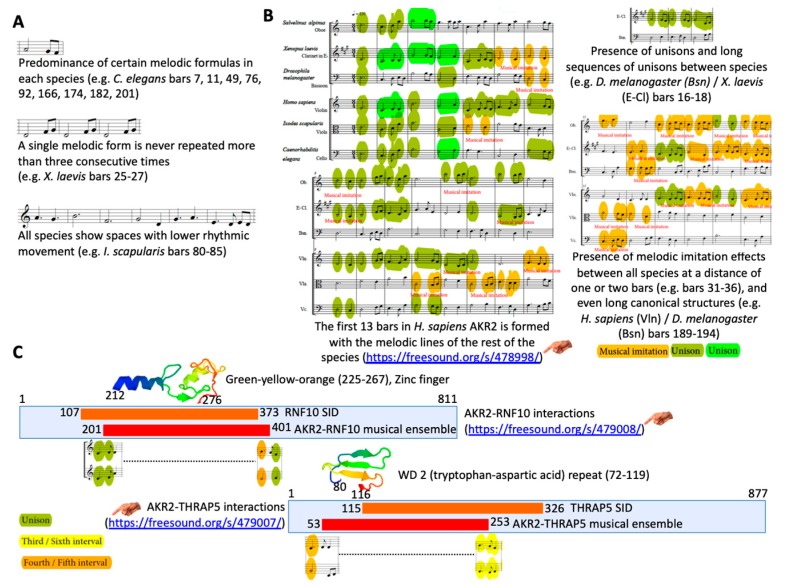
The sound of AKR evolution and protein interactions. (**A**) Examples of regularities observed when comparing AKR/SUB musical scores between different species (*Caenorhabditis elegans akr* NM_058903.6, *Drosophila melanogaster akr* NM_139856.4, *I. scapularis akr/subolesin* AY652654.1, *Xenopus laevis akr2* NM_001092015.1, *Salvelinus alpinus akr2* GQ247760.1, and *Homo sapiens akr2* NM_018064.3; Figure S2). (**B**) Examples of the findings when comparing the six species in a polyphonic context (Figure S3). (**C**) The AKR2-RNF10 and AKR2-THRAP5 but not AKR2-IFN-α1 interactions are manifested in the higher and repeated presence of unison between both sequences (e.g., AKR2-RNF10 bars 232–235, AKR2-THRAP5 bars 83–85), as well as a higher presence of fifth and fourth consonances (e.g., AKR2–RNF10 bars 210–219, AKR2–THRAP5 bars 67–78) (Figure S4). Audio files were uploaded and can be found in Figure S4 (78998 to 479009).

Previously, we proposed an algorithm to characterize genome structural features on the basis of the fact that the non-random organization of the genome must have left a structural imprinting in the sound of the DNA language [8]. This method was based on the communicative strength of music to show the presence of syntactical structures in the DNA language, and some properties of these structures [8]. Herein, we applied an improved version of this method to translate AKR/SUB sequences into musical scores and music (Figures S2 and S3) to provide an alternative and complementary approach to the study of AKR evolutionary relationships and functional protein interactions. As shown previously by genetic approaches [17], musical ensembles supported the fact that AKR in different species are evolutionarily related and structurally conserved (Figure 5A,B and Figure S3). The results showed characteristics of the AKR/SUB proteins evidenced by the predominance of certain melodic formulas in each species, the fact that a single melodic form was never repeated more than three consecutive times, and that all species showed spaces with lower rhythmic movement (Figure 5A). When comparing the AKR/SUB from the six analyzed species (Figure 5A) in a polyphonic context, the results showed the presence of unisons, long sequences of unisons, and melodic imitation effects even having long canonical structures that represent regions of amino acid homology between species (Figure 5B). To use this approach to further characterize the AKR/SUB interactome, the sequences of AKR2-interacting proteins RNF10 and THRAP5 (Table 1) and IFN-α1 as negative control were translated into music (Figure S2) to produce musical ensembles of AKR2–protein interactions by trial and error. The results showed that both AKR2–RNF10 and AKR2–THRAP5 but not AKR2–IFN-α1 musical ensembles (Figure S4) predicted protein interactions coinciding with those found by Y2H (Figure 5C).

### 3.3. AKR2–Protein Interactions Positively and Negatively Regulated the NF-κB Signaling Pathway

**Figure 6:**
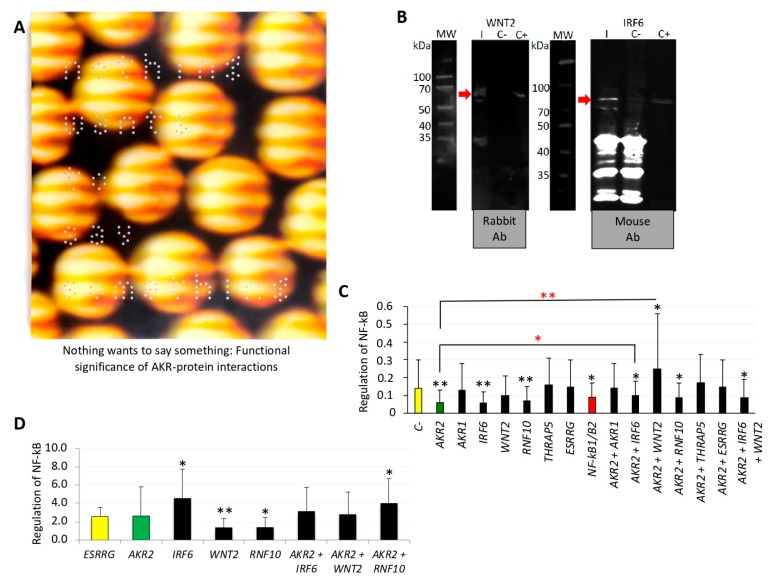
Function of AKR2 interactome in the regulation of NF-κB. (**A**) The piece “Nothing wants to say something” by Raúl Cordero (acrylic, polyester, oil and metallic pigments on canvas, 210 × 190 cm; courtesy of the artist) challenges the view of random AKR–protein interactions and suggests that these interactions are functionally relevant in the regulation of different biological processes. (**B**) Western blot analysis of AKR2–IRF6 and AKR2–WNT2 interactions. Interacting proteins were incubated with AKR2 and immunoprecipitated with c-Myc magnetic beads specific for the AKR2 protein tag (I) or with the c-Myc magnetic beads only as negative control (C-). Recombinant interacting proteins were included as positive control (C+). Antibodies specific for each protein were used to identify the presence of the interacting proteins (red arrow). The origin of the primary antibody is shown. (**C**,**D**) Regulation of NF-κB in response to AKR2 and interacting proteins. (**C**) The NF-κB reporter was used for monitoring the activity of the NF-κB signaling pathway in human placenta-cultured cells after gene knockdown by RNAi. The ratio of firefly luminescence from the NF-κB reporter to *Renilla* luciferase vector control was represented as average + SD and compared between siRNA-treated groups and the siRNA negative control (C-) (black asterisks), and between combined siRNA-treated groups and the *AKR2* siRNA (red asterisks) by Student’s *t*-test with unequal variance and one-way ANOVA with similar results (* *p* < 0.05, ** *p* < 0.005; *n* = 6 biological replicates). (**D**) The NF-κB reporter was used for monitoring the activity of the NF-κB signaling pathway in human placenta cells after transfection of recombinant proteins AKR2, RNF10, WNT2, IRF6, and combinations with AKR2. As a positive control, a FITC-antibody was transfected. Negative control cells were transfected with the ESRRG protein. The firefly to *Renilla* normalized luciferase activity for NF-κB reporter was represented as average + SD and compared between protein-treated groups and the ESRRG control by Student’s *t*-test with unequal variance (* *p* < 0.05, ** *p* < 0.005; *n* = 4 biological replicates).

The next step in the characterization of AKR2–protein interactions was to address the question of how these interactions functionally regulate specific signaling pathways. In the piece “Nothing wants to say something” (Figure 6A), the artist proposed a new approach to address scientific challenges on the basis of “visual vibrations” that somehow should modify the experience and internal chemical processes that occur when looking at a piece of art. According to the artist’s statement, “what you are looking for is also looking for you, highlighting that nothing wants to tell you something and it is only a matter of finding how to perceive the message”. From a scientist’s perspective, this message challenges the view that AKR/SUB–protein interactions occur randomly, and suggests that these interactions are functionally relevant in the regulation of different biological processes as occurs with other regulatory factors [44]. To address this question, the NF-κB signaling pathway was selected due to its role in the IMD immune response biological process [15,16,17,26,28]. We first identified proteins previously described as implicated in the regulation of the evolutionarily conserved NF-κB pathway (Table 1) [45,46,47,48,49,50,51]. Then, the interactions between AKR2-IRF6 and AKR2-WNT2 (D-score moderate confidence of interactions; Table 1) was corroborated in vitro (Figure 6B). The characterization of the AKR2-interacting unrelated proteins with lower molecular weight was addressed before (Figure 4A,D). The genes coding for these proteins were knockdown (Figure 6C and Figure S5) or protein levels increased by protein transfection (Figure 6D) in human placenta cells alone and in combination with AKR2 to characterize their role in the regulation of the NF-κB signaling pathway.

**Figure 7:**
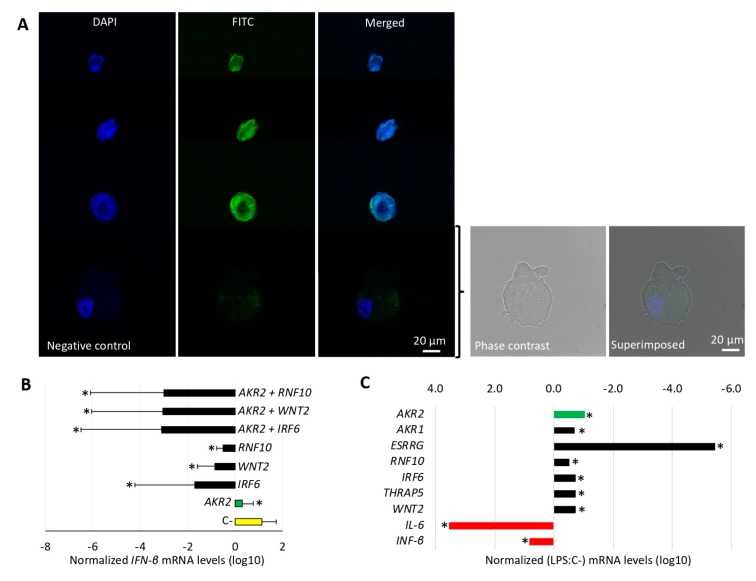
AKR2 interactome differentially regulated the NF-κB pathway. (**A**) Protein transfection was confirmed in FITC-antibody positive control-transfected cells and untreated negative control cells by fluorescence microscopy. Host cell nucleus was stained with DAPI (blue). Image of negative control cells was also collected by phase contrast microscopy and superimposed to FITC/DAPI merged image. Bar: 20 µm. (**B**) The expression of *IFN-β* was characterized in human placenta cells after gene knockdown by qRT-PCR. The *IFN-β* mRNA levels were normalized against human *β-actin*, and normalized Ct values were compared between test siRNA-treated placenta cells and controls treated with non-targeting siRNA (C-) by Student’s *t*-test with unequal variance (* *p* < 0.05; *n* = 6 biological replicates). (**C**) The expression of *AKR2*; genes coding for interacting proteins *AKR1*, *ESRRG*, *RNF10*, *THRAP5*, *IRF6*, and *WNT2*; and regulated genes *IFN-β* and *IL-6* was characterized in human placenta cells treated with lipopolysaccharides (LPS) by qRT-PCR. The mRNA levels were normalized against human *β-actin*, the normalized LPS to PBS-treated control (C-) ratio (LPS: C-) Ct values were calculated, and normalized Ct values were compared between LPS-treated and C-cells by chi^2^ test (* *p* < 0.001; *n* = 6 biological replicates).

The results of gene knockdown experiments provided evidence for the role of AKR2, IRF6, RNF10, and AKR2 interactions with IRF6, RNF10, and IRF6/WNT2 in the positive regulation of NF-κB, whereas AKR2–WNT2 interactions appeared to negatively regulate the NF-κB signaling pathway (Figure 6C). ESRRG alone or in combination with AKR2 did not affect NF-κB regulation (Figure 6C) and was used as a negative control in the protein transfection experiment (Figure 6D). The increase in protein levels corroborated the NF-κB positive regulation by IRF6 and AKR2–RNF10 interactions, whereas transfection of WNT2 and RNF10 proteins resulted in the negative regulation of NF-κB (Figure 6D). Transfection of AKR2 alone did not affect NF-κB regulation (Figure 6D). Discrepancies between results of gene knockdown (Figure 6C) and protein transfection (Figure 6D) experiments may be explained at least in part by the fact that proteins appeared to directly enter cell nucleus after transfection (Figure 7A), which may prevent post-translational modifications required for protein function. In this case, post-translational modifications would have functional implications for AKR2 and RNF10. Additionally, differences between results of *AKR2* gene knockdown and protein transfection may also suggest that AKR2 is necessary but not sufficient to upregulate NF-κB (Figure 6C,D).

The expression of *IFN-β* gene regulated by IRF and NF-κB factors [52,53,54,55,56] and therefore identified as an NF-κB target gene (https://www.bu.edu/NF-κB/gene-resources/target-genes/) was characterized in human placenta cells after gene knockdown (Figure 7B). The knockdown of *AKR2*, *IRF6*, *WNT2*, and *RNF10* alone and in combination with *AKR2* resulted in significant downregulation of *IFN-β* (Figure 7B). This effect was higher for combined *AKR2*-*RNF10*, *AKR2*-*WNT2*, and *AKR2*-*IRF6* gene knockdown with a downregulation of *IFN-β* by 13,400- to 16,750-fold (Figure 7B).

### 3.4. LPS Did Not Activate the NF-κB Pathway Via AKR2–Protein Interactions in Human Placenta Cells

Bacterial LPS binding to Toll-like receptor 4 (TLR4) results in the activation and translocation of NF-κB transcription factor into the nucleus, which activates the expression of target genes such as *IL-6*, *IL-8,* *IFN-β*, and tumor necrosis factor (*TNF*) [57,58] that have been implicated in the response to LPS in placenta cells [59]. However, LPS-mediated TLR4 signaling occurs through the myeloid differentiation primary response gene 88 (MyD88) and signaling adaptor protein TIR domain-containing adaptor-inducing interferon-beta (TRIF), which regulate NF-κB translocation into the nucleus to induce gene transcription [60].

To explore the possible role of AKR2 and the interacting proteins in the NF-κB signaling pathway in response to LPS, human placenta cells were treated with LPS and the expression of selected genes characterized by qRT-PCR (Figure 7C and Figure S6). The results showed that LPS treatment induced downregulation of genes coding for AKR2 and the interacting proteins AKR1, ESRRG, RNF10, IRF6, THRAP5, and WNT2 (Figure 7C). However, the NF-κB-regulated genes *IL-6* and *IFN-β* were induced in LPS-treated placenta cells, thus supporting NF-κB activation in response to LPS (Figure 7C). These results supported the fact that AKR2–protein interactions do not mediate LPS activation of the NF-κB pathway in human placenta cells. In these cells, alternative mechanisms such as those mediated by MyD88 and TRIF or others still to be characterized may be involved in the activation of the NF-κB signaling pathway. Nevertheless, LPS-induced down regulation of AKR2 interacting proteins with negative regulation of NF-kB may be a compensatory mechanism to favor the production of certain NF-kB regulated genes such as IFN-β. The downregulation of genes coding for AKR2 and interacting proteins in response to LPS may constitute a mechanism by which placenta cells alter NF-κB dynamics.

### 3.5. The Characterization of the AKR/SUB Interactome Had Implications for Quantum Vaccinomics

**Figure 8:**
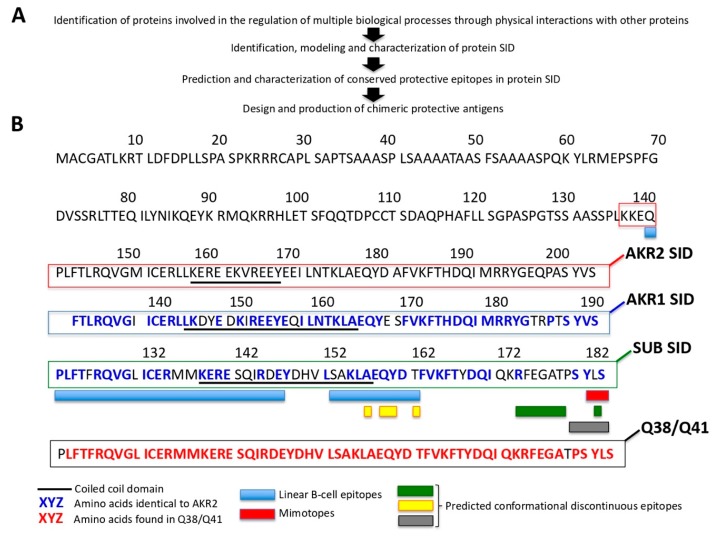
SUB/AKR interactome and possibilities for quantum vaccinomics. (**A**) Pipeline for quantum vaccinomics by focusing on protective epitopes in peptide sequences involved in protein–protein interactions or SID that are particularly relevant for proteins such as SUB/AKR that function through these interactions. (**B**) Alignment of SID amino acid sequences for human AKR2 and AKR1, and *I. scapularis* tick SUB with the corresponding region in the SUB/AKR chimeric Q38 and Q41 protective antigens. The Q38- and Q41-identified and/or predicted protective epitopes are shown.

The proposed pipeline for quantum vaccinomics consisted of the following steps (Figure 8A): (a) characterization of cell interactome and regulome in vector–host–pathogen interactions for the identification of proteins such as AKR/SUB involved in the regulation of multiple biological processes through physical interactions with other proteins; (b) identification, modeling, and characterization of protein SID; (c) prediction and characterization of conserved protective epitopes in protein SID; and (d) design and production of chimeric protective antigens.

**Figure 9:**
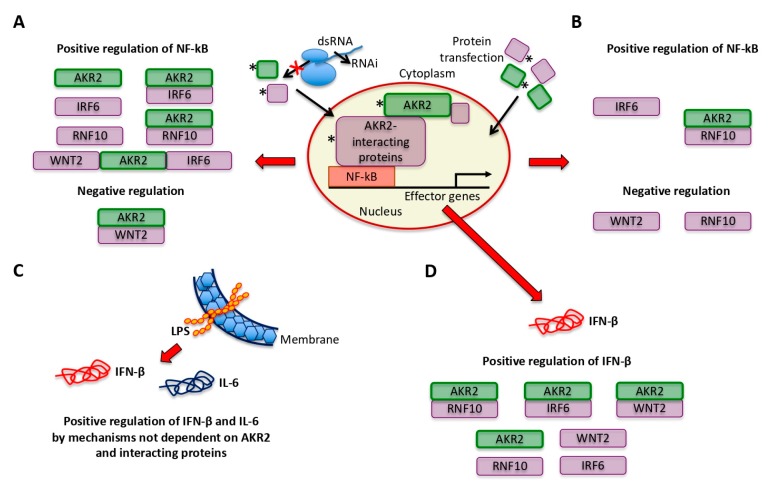
Model for NF-κB regulation by human AKR2 interactome in human placenta cells after (**A**) gene knockdown by RNAi, (**B**) protein transfection, (**C**) *IFN-β* and *IL-6* expression after treatment with LPS, and (**D**) *IFN-β* expression after gene knockdown by RNAi. Predictions are based on results from experiments shown in Figure 6C,D and Figure 7B,C.

As a proof-of-concept, the SID identified here in AKR2 and AKR1, and predicted for tick SUB [17] were aligned and showed more than 60% amino acid sequence identity (Figure 8B). Then, the protective epitopes included into Q38 and Q41 AKR/SUB chimeras [34,38] were mapped and covered more than 75% of the SID (Figure 9B). Considering the protective efficacy of Q38 and Q41-based vaccines [34,35,61], these results supported targeting SID in quantum vaccinomics.

## 4. Discussions

Our combined scientific and artistic perspectives provided a multidisciplinary complementary approach to advance the knowledge of the AKR/SUB model regulatory factor and supported the collaboration between science and art in research (Table 2). It has been proposed that AKR/SUB acts through functional interactions with other proteins and chromatin remodeling [16,17,19,20,21,22,23,24,25,26,27,28], but additional information is needed about the role of AKR/SUB interactome in these processes. Artists did not merely illustrate scientific results but provided their perspectives on the AKR/SUB–protein interactions, which challenged scientists to provide mechanistic and regulatory insights into the functional implications of these interactions.

Phylogenetic or evolutionary trees are the most accepted approach to illustrate the inferred evolutionary relationships between different species on the basis of the similarities and differences in their physical or genetic characteristics. Furthermore, selective evolutionary pressures have driven a non-simple random-based organization of the genome of surviving organisms. Several methods have been applied to the formulation of DNA language and encoded information, showing that there is at least some degree of organization in the nucleotide sequences found in many organisms [8,62,63,64]. Our results advanced the use of the DNA language approach a step forward by characterizing not only evolutionary relationships but also functional protein interactions (Table 2).

The results supported the fact that the multiple interactions between AKR2 and interacting proteins differentially regulate the NF-κB pathway by affecting gene expression controlled by NF-κB in a biological context-dependent manner (Figure 8A–D). In all experiments, except in LPS-treated cells, the results suggested that AKR2 and IRF6 alone and the combination of AKR2–RNF10 positively regulate the NF-κB signaling pathway. Differences between different experiments support the possibility of the differential regulation of the NF-κB pathway in a biological context-dependent manner.

Of the identified AKR2–protein interactions in human placenta cells, five proteins with very high confidence in the interactions representing 9.6% of the identified proteins included AKR1, ACTR10, RNF10, SF3A1, and THRAP5 (Table 1). Of them, AKR1, RNF10, and THRAP5 have been previously described as involved in the regulation of the NF-κB signaling pathway [45,46,47,48,49,50,51]. One of the remaining challenges addressed in this study by using the NF-κB signaling pathway as a model was the characterization of the mechanisms by which AKR2–protein interactions are regulated by cell type-specific models that in turn regulate different biological processes (i.e., immune response or development). It has been proposed that AKR/SUB specifies NF-κB selectivity of innate immune response via chromatin remodeling [25,27]. However, the results of our study provided evidence suggesting that in human placenta cells the differential interaction between AKR2 and the interacting proteins IRF6, RNF10, and WNT2 differentially regulate the NF-κB signaling pathway. The role of AKR dimerization/multimerization in the regulation of these protein interactions is also potentially functionally relevant and requires additional research.

Previously, van Essen et al. [38] proposed that the activation of NF-κB by NF-κB (p65) depends on the availability of suitable secondary transcription factors, a process determined by cell-type and stimulus. Herein, we showed that some of these secondary transcription factors may be AKR2 and interacting proteins, and suggested that AKR2–protein interactions may affect gene expression controlled by NF-κB in a biological context-dependent manner. The fact that *AKR2* gene knockdown but not protein transfection affected NF-κB regulation provided additional evidence that AKR2 needs to interact with other proteins to activate the NF-κB pathway [28]. Additionally, the results supported the role proposed previously for post-translational modifications in the function of AKR/SUB and interacting proteins [17,24].

Quantum vaccinomics will advance the design of more effective and safe vaccines to target some of the challenges posed for ectoparasite control vaccines [65]. The algorithm for quantum vaccinomics proposed here focusses on proteins involved in cell interactome and regulome and functioning through protein–protein interactions for the regulation of multiple biological processes involved in vector-host-pathogen interactions. In this way, vaccination with AKR/SUB protective epitopes in SID will induce an antibody response not only interfering with protein translocation to the nucleus [66], but also blocking AKR/SUB–protein interactions involved in the regulation of multiple biological processes. Vaccines based on Q38 and Q41 AKR/SUB protective epitopes chimeras have proven efficacy for the control of different ectoparasites and infection by vector-borne pathogens [34,35,61]. Nevertheless, future experiments should address the effect of these vaccines on the regulation of various biological processes to further advance the possibility of developing a vaccine for the control of vector infestations and pathogen infection/transmission by multiple species [67].

## 5. Conclusions

The results of this study provided a novel combined scientific and artistic multidisciplinary approach to address challenging questions in molecular biology. The collaboration between scientists and artists provided two main methodological outcomes: (a) the suggestion by visual artists of the scientific characterization of previously unexplored properties of AKR/SUB and (b) the application of an algorithm using musical ensembles based on AKR/SUB and interacting protein sequences as a new method to predict protein–protein interactions (Table 2). The recent review of AKR/SUB functional evolution [17] suggested that these proteins evolved with conserved sequence, structure, and function, and that there is a need to further characterize its function in different conserved biological pathways such as NF-κB. The need to better understand AKR/SUB function was addressed in this study, and although further experiments are required to fully characterize the role of the AKR/SUB interactome in the regulation of the NF-κB pathway, the results in human placenta cells served as a methodological proof-of-concept to advance this research area. The results showed that AKR–AKR interactions result in protein dimerization/multimerization with possible functional implications that require attention. AKR/SUB physically interacts with different proteins simultaneously to regulate various biological processes with cell type differences in AKR2 interactome that are likely associated with the specific biological processes regulated by this protein in each cell type. In this way, multiple interactions between AKR2 and interacting proteins differentially regulate the NF-κB pathway by affecting gene expression controlled by NF-κB in a biological context-dependent manner. In addition to cell type-specific differences in AKR/SUB-protein interactions, species-specific differences have been also described and require further attention [17]. For example, it has been shown in tick cells that SUB regulome affects multiple biological processes that vary in response to infection with the tick-borne pathogen *Anaplasma phagocytophilum* [17]. Therefore, studies in other cells such as lymphocytes or monocytes and promyelocytic leukemia HL-60 model cells are required to evaluate cell type-specific differences in the function of AKR2–protein interactions in NF-κB signaling. Functionally, AKR2–protein interactions in human placenta cells differentially regulate the NF-κB signaling pathway, suggesting that AKR2 interacting proteins might constitute suitable secondary transcription factors for cell and stimulus-specific regulation of NF-κB. As shown previously by genetic approaches, musical ensembles supported the fact that AKR/SUB in different species are evolutionarily related and structurally conserved, and also predicted AKR2–protein interactions coinciding with those found by genetic approaches such as the Y2H screening used here. The methodological approach proposed here for quantum vaccinomics further advances the potential of the vaccinomics pipeline used before for the identification of candidate protective antigens [29,68,69]. Furthermore, if combined with network analysis for the integration of interactomics and regulomics datasets [70] and big data machine learning algorithms to identify candidate protective antigens and epitopes [71], quantum vaccinomics would result in designing chimeric antigens based on protective epitopes in SID from vector- and pathogen-derived regulatory proteins. Vaccines with these chimeric protective antigens would address the possibility of effective and sustainable control of vector-borne diseases by targeting multiple ectoparasite and pathogen species in multiple hosts. The collaboration between scientists and artists from multiple disciplines has a positive impact on advancing research to address scientific challenges.

## Figures and Tables

**Table 1:** Proteins identified with A–D scores as candidates for interactions with AKR2.

Name	Description	Protein/Gene ID	Interaction Score (PBS)
AKR1 ^§^	Akirin1	Q9H9L7	A
ACTR10	Actin related protein 10	Q9NZ32	A
RNF10 ^§^	RING finger protein 10	Q8N5U6	A
SF3A1	Splicing factor 3a subunit 1	Q15459	A
THRAP5 ^§^	Mediator of RNA polymerase II transcription subunit 16	Q9Y2X0	A
AKR2 ^§^	Akirin2	Q53H80	B
ESRRG ^§^	Estrogen related receptor gamma	P62508	B
PITPNA	Phosphatidylinositol transfer protein alpha isoform	Q00169	B
ATF4	Cyclic AMP-dependent transcription factor ATF-4	P18848	D
C12orf10	Chromosome 12 open reading frame 10	Q86UA3	D
CCNE1	G1/S-specific cyclin-E1	P24864	D
CDC23	Cell division cycle protein 23 homolog	Q9UJX2	D
CLIC3	Chloride intracellular channel protein 3	O95833	D
CLTCL1	Clathrin heavy chain 2	P53675	D
CNOT3	CCR4-NOT transcription complex subunit 3	O75175	D
EXPH5	Exophilin 5	Q149M6	D
EZR	Ezrin	P15311	D
FNTA	Protein farnesyltransferase/geranylgeranyltransferase type-1 subunit alpha	P49354	D
HEG1	Protein HEG homolog 1	Q9ULI3	D
HNRPF	Heterogeneous nuclear ribonucleoprotein F	P52597	D
IFT57	Intraflagellar transport protein 57 homolog	Q9NWB7	D
INTS2	Integrator complex subunit 2	Q9H0H0	D
IRF6 ^§^	Interferon regulatory factor 6	O14896	D
KLHL5	Kelch-like 5 isoform 2 variant	Q59HD9	D
LIMS1	LIM and senescent cell antigen-like-containing domain protein 1	P48059	D
LNX1	E3 ubiquitin-protein ligase LNX	Q8TBB1	D
PKM	Pyruvate kinase PKM	P14618	D
SCFD1	Sec1 family domain-containing protein 1	Q8WVM8	D
SERPINE2	Glia-derived nexin	P07093	D
SF3B1	Splicing factor 3B subunit 1	O75533	D
SPG21	Maspardin	Q9NZD8	D
SPTBN1 var 1	Spectrin beta chain, non-erythrocytic 1	Q01082	D
TOPBP1	DNA topoisomerase 2-binding protein 1	Q92547	D
URB2	Unhealthy ribosome biogenesis protein 2 homolog	Q14146	D
USP7	Ubiquitin carboxyl-terminal hydrolase 7	Q93009	D
VPS39	Vam6/Vps39-like protein	Q96JC1	D
WNT2 ^§^	Protein Wnt-2	P09544	D
ZNF862	Zinc finger protein 862	O60290	D
GAREM1 *	GRB2-associated and regulator of MAPK protein1	Q9H706	D
Mitochondrion *	Mitochondrion, complete genome	MF992925	D
RBPMS *	RNA binding protein, mRNA processing factor (RBPMS), on chromosome 8	NG_029534.1	D
BRD7 *	Bromodomain containing 7 (BRD7), on chromosome 16	NG_023418.1	D
LSS *	Lanosterol synthase (LSS), on chromosome 21	NG_011510.1	D
DRC3 *	DRC3 gene, complete cds	AF282168.1	D
AF-6 *	AF-6, complete cds	AB011399.1	D
DAPK1 *	Death-associated protein kinase 1 (DAPK1) gene, complete cds	DQ436495.1	D
EDH17B2 *	17-beta-hydroxysteroid dehydrogenase (EDH17B2) gene, complete cds	U34879.1	D

PBS: A, very high confidence in the interaction; B, high confidence in the interaction; D, moderate confidence in the interaction. ^§^ Proteins previously described as involved in the regulation of the NF-κB signaling pathway. * Entries with gene sequence matches (Data S1).

**Table 2:** Highlights of the collaboration between artists and scientists in this study.

Artist Contribution	Scientist Perspective
**Methodological Approach**	
Three visual artists and a musician were invited to read a simplified version of our recent review on AKR/SUB functional evolution [17] and were challenged with the conserved function of these proteins in the regulation of different biological processes throughout the metazoan.	In response to this challenge, the artists contributed the pieces, musical scores, and interpretations shown in the paper in order to provide their view on this matter. Artists’ contributions served to inspire scientists to discuss and find new perspectives on unexplored characteristics of these proteins with putative functional implications.
**Piece in Figure 1A**	
The artist represents the origins of life, with multiple geometric images that interact to illustrate in different species the conserved function of these proteins in biological processes represented by the sea, paper boat, Picasso’s dove, and a fetus growing in a mother’s womb while opening its eyes to the world.	From a scientist’s perspective, a new and unexplored facet of possible functional relevance of AKR dimerization was proposed in this piece.
**Piece in Figure 2A**	
The artist describes the constant movement of these proteins that translate into the visual rhythm of repetitive interconnected interactions of forms and colors.	From a scientist’s perspective, the possibility that AKR physically interacts with different proteins simultaneously to regulate various biological processes defined by cell-specific AKR–protein interactions was proposed in this piece.
**Piece in Figure 6A**	
The artist proposed that what you are looking for is also looking for you, highlighting that nothing wants to tell you something and it is only a matter of finding how to perceive the message.	From a scientist’s perspective, this message challenges the view that AKR–protein interactions occur randomly and suggests that these interactions are functionally relevant in the regulation of different biological processes as occurs with other regulatory factors.
**Musical Scores in Figure 5 and Figures S2–S4**	
A revised algorithm using musical ensembles was applied to translate the AKR and interacting protein sequences into music.	The results supported that AKR in different species are evolutionarily related and structurally conserved, and confirmed AKR2–protein interactions identified in this study. Therefore, this tool may be used for evolutionary studies and the prediction of protein–protein interactions.

## Supplementary Materials

The following are available online at https://www.mdpi.com/2076-393X/8/1/77/s1: Figure S1: Annotation of the AKR2–protein interacting domains. Figure S2: Translation of AKR and selected interacting protein sequences into musical scores. Figure S3: Multi-species AKR musical ensemble. Figure S4: Musical ensemble of AKR2–protein interactions. Figure S5: Gene knockdown by RNAi in human placenta cells. Figure S6: Gene expression in LPS-treated human placenta cells and controls. Data S1: Results of the ULTImate Y2H screening of human AKR2 bait vs. human placenta RP6 library prey.
