# Expression of Concern: Artigas-Jerónimo et al. A Novel Combined Scientific and Artistic Approach for the Advanced Characterization of Interactomes: The Akirin/Subolesin Model. *Vaccines* 2020, *8*, 77

**DOI:** 10.3390/vaccines8030418

**Published:** 2020-07-25

**Authors:** Vaccines Editorial Office

**Affiliations:** MDPI, St. Alban-Anlage 66, 4052 Basel, Switzerland; vaccines@mdpi.com

The Editorial Office of *Vaccines* has decided to issue the following Expression of Concern about the published paper: A Novel Combined Scientific and Artistic Approach for the Advanced Characterization of Interactomes: The Akirin/Subolesin Model [1], following concerns received from a reader and Editorial Board Members of *Vaccines*, after peer-review and publication.

As a matter of transparency, and upon agreement with all parties involved, these comments and the authors’ responses to these comments are made available below. The “Expression of Concern” was prepared in agreement with the authors. As a publisher, MDPI does not wish to deliberately avoid the above rebuttal, but we hope that by bringing this discussion publicly will raise awareness that the article should be considered carefully.

MDPI remains committed to publishing high-quality peer-reviewed scientific research, in all fields and in all disciplines.

**Comment from Reader**: “I would like to ask for your comment on the attached link to a paper that appears to have been published in the MDPI family of journals (Vaccines). It was highlighted in a recent literature alert as I have an interest in protein-protein interactions. I found some of the wording in the abstract frankly alarming (e.g., “quantum vaccinomics”) and am concerned at the reputational repercussions it may have on the MDPI journal family, and the poor impression it gives of your peer review process [1].
**Comment from Editorial Board Member 1**: “It seems an odd publication for Vaccines as I do not understand the main objective of the study. The primary focus does not seem to be vaccine research. I would not consider this a high impact study. Some wording seems weird and could be due to sensationalism to present the findings as being more impactful. The funding source of the study also seems questionable for a focused manuscript on vaccine science. I would have rejected this manuscript”.
**Comment from Editorial Board Member 2**: “There is no doubt that this manuscript is very unusual for a scientific paper. If I had been the editor, I don’t think I would have published this. However, I don’t see anything that suggests it should be retracted (there are plenty of experiments conducted with statistics, etc.). The phrase “quantum vaccinomics” is also strange, but appears to be an attempt by the authors to establish a new phrase to label a new approach. The authors had used music-based algorithms in the past (reference 8), so there is at least a history of this unconventional approach”.

The authors provided the following responses to these comments:

**Authors’ Response to Reader**: As disclosed in the paper, “The characterization of protein-protein interactions and vaccinomics have been proposed as novel approaches for vaccine development [29]. Considering the structural and functional conservation of AKR/SUB proteins [17], the characterization of the AKR2 interactome may have implications in quantum vaccinomics as a new approach for the development of vaccines for the control of vector infestations and infection/transmission of vector-borne pathogens. In this context, quantum vaccinomics could be focused on cell interactome and regulome for the identification of protective epitopes in peptide sequences involved in protein-protein interactions or selected interacting domains (SID) that are particularly relevant for proteins such as AKR/SUB that function through these interactions” Herein, we proposed a novel methodological approach to advance the characterization of protein–protein interactions for AKR/SUB with a proven vaccine efficacy and thus supporting the possibility to advance in the quantum vaccinomics, one of the main directions in future vaccine development. The results of the paper do support this possibility.
**Authors’ Response to Editorial Board Member 1**: The objectives of the study are clearly disclosed in the paper, and include an algorithm for quantum vaccinomics, which is supported by the results presented in the paper and do have an impact on vaccine research. The funding for the study came from the project “de la Fuente, J. & Villar, M. PIs. Subolesin/Akirin interactome and function in the regulation of immune response in invertebrate vector (tick) and vertebrate (human) cells. Ministerio de Economía, Industria y Competitividad Spanish Research Program, grant BFU2016-79892-P. 2017-2020”, which do include the translational research. In summary, the results of the paper do have relevance for vaccine research and are well supported. Would you have rejected the manuscript before fully understanding the content of the paper and ignoring authors, reviewers and editor arguments?
**Authors’ Response to Editorial Board Member 2**: First focusing on this paper, the editor clearly stated that there is no reason to retract the paper. Quantum vaccinomomics was introduced as translational research of this innovative methodological approach that is well explained and supported by the results of the study. In order to advance vaccine research, it is important to address challenges with novel methodological approaches. In addition to the specific responses above, we would like to highlight that we have never received that kind of comment on an accepted and published paper.
**Comments from the Editor-in-Chief**: The manuscript does not provide methodological proof-of-concept to advance the position of ‘quantum vaccinomics’, which is inadequately defined in the literature or the manuscript. A proof-of-concept is guided by determining if an idea can be turned into a reality, and is meant to determine the feasibility of the idea or to verify that it will function as envisioned. Akirins (AKR) are highly conserved nuclear proteins required for NF-κB-dependent gene expression in drosophila and mice (Nature Immunology volume 9, pages 97–104 (2008). Subolesin (SUB) is an evolutionary conserved molecule in arthropod species that has a role in the regulation of genes involved in immune responses (Parasites & Vectors volume 8, Article number: 132 (2015)). The evolution and function of AKR/SUB are challenging if one evaluates the molecular interactions in a particular cell type, particularly as the ‘interactome’, which is variant and interacts among many proteins but can also have indirect interactions among genes. The interactome and function of these proteins changes based upon different stimuli, so ‘quantum vaccinomics’ is not operable as defined or implied in the manuscript and there is no scientific basis for the association of visual art, music, and function occur with the AKR2 interactome in the regulation of the NF-κB pathway in human placenta cells. Further, the results do not serve to advance this research area. There is no scientific evidence regarding whether musical perspective supports AKR/SUB evolutionary conservation or if it provides new mechanistic insights into the AKR2 interactome. It is not clear if the combined scientific and artistic perspectives result in a multidisciplinary approach for advancing our knowledge on AKR/SUB interactome.

Regards,

Prof. Dr. Ralph Tripp

Editor-in-Chief

**Authors’ Response to Editor-in-Chief**: The high-resolution description of the immune response and protective antigens is important to advance the identification of protective epitopes, the immunological quantum, leading to quantum vaccinology. In our paper, we provided in Section “*3.5. The characterization of the AKR/SUB interactome have implication for quantum vaccinomics*” an approach for quantum vaccinology by using the interactome of proteins with key functions in cell regulome such as Akirin/Subolesin. Then, as a proof-of-concept, the protective epitopes included into Q38 and Q41 AKR/SUB chimeras [*Vaccine* 2013, *31*, 1187–1196] were mapped and covered more than 75% of the interacting domains (SID) (Figure 9B). Considering the protective efficacy of Q38- and Q41-based vaccines [refs. 44,62,63], we proposed that these results support targeting SID in quantum vaccinomics. Regarding the proposed methodological approach combining scientific and artistic approaches, we did not claim any implication in quantum vaccinology, which was a proposed implication of the results of the study. The advance that the collaboration between artists and scientists represented for our study was clearly disclosed in the paper and summarized in “Table 2. Highlights of the collaboration between artists and scientists in this study”. We all agree in that this methodology is challenging and may be questionable by other scientists, but it did not affect the scientific validity of the results presented in the paper. In particular, the proposed musical algorithm provided an unexpected result by not only advancing the study of AKR/SUB evolution through musical syntactical structures (https://freesound.org/people/josedelafuente/sounds/478998 to 479009), but also to produce musical ensembles of AKR2-protein interactions by trial and error, showing protein SID coinciding with those found by Y2H (Section “*3.2. The sound of the AKR/SUB coding sequenced support evolutionary conservation and functional protein interactions*”). Based on the results of the study and as stated in the paper, we consider that the collaboration between scientists and artists provided a multidisciplinary approach for advancing our knowledge on AKR/SUB interactome by two main methodological outcomes: (a) the suggestion by visual artists of the scientific characterization of previously unexplored properties of AKR/SUB and (b) the application of an algorithm using musical ensembles based on AKR/SUB and interacting protein sequences as a new method to predict protein–protein interactions. Finally, our proposal “to publish a response to these comments to show to the scientific community why in our opinion *Vaccines* is not making a well supported decision on this paper” was not a threat but a claim for transparency, as would be now achieved by publishing this expression of concern together with our response. Including the names of the reader and editorial board members raising the concerns included in this document will further contribute to this goal.

In summary, we and other scientists, among those that have viewed (707 views by May 8th), downloaded (779 downloads by May 8th) and cited the paper (*Ticks Tick Borne Dis*. 2020; 101445. doi:10.1016/j.ttbdis.2020.101445), consider that this study has advanced our knowledge of the AKR/SUB interactome and proposed new methodological approaches to address scientific challenges and ways to investigate quantum vaccinology.
