# Supplementary material for: A Novel Combined Scientific and Artistic Approach for the Advanced Characterization of Interactomes: The Akirin/Subolesin Model
Source: Vaccines (Basel). 2020 Feb 8;8(1):77. doi: 10.3390/vaccines8010077 (PMC7157757; doi:10.3390/vaccines8010077)
Supplement: Supplementary file 1 [file vaccines-08-00077-s001.zip › Supplementary Materials.pdf]

Supplementary Materials

A novel combined scientific and artistic approach for the advanced characterization of interactomes: the Akirin/Subolesin model

Sara Artigas-Jerónimo, Juan J. Pastor Comín, Margarita Villar, Marinela Contreras, Pilar Alberdi, Israel León Viera, Leandro Soto, Raúl Cordero, James J. Valdés, Alejandro Cabezas-Cruz, Agustín Estrada-Peña, José de la Fuente*

*Correspondence to: jose_delafuente@yahoo.com

This PDF file includes:

Figures S1 to S6

HYBRIGENICS SERVICES

DomSight: PLA_RP6_hgx4913v1 vs. Human Placenta_RP6 (14 Nov 2017)

(Bait plasmid(s): hgx4913v1_pB27)

Legend	
Bait fragment	
SID fragment	
Pfam or SMART domain	
Transmembrane domain (TMHMM)	
Coiled-coil domain (Ncoils)	
Signal peptide (SignalP)	

SID: Selected Interaction Domain
It is the amino acid sequence shared by all prey fragments matching the same reference protein. SIDs have been found in numerous cases to correspond to an identified structural or functional domain.
Protein Display:
Proteins larger than 1500 aa are shown in sections of 1499 aa each. Only sections containing bait fragments, SIDs or predicted functional and structural domains are represented.

Legend

AKIRIN2 – Homo sapiens – ACCESSION: NM_018064.3 – Gene ID: 55122 (1–203)
- 1 – 203 hgx4913v1
- 157 – 177 Coiled domain

A — ACTR10 – Homo sapiens – ACCESSION: NM_018477.2 – Gene ID: 55860 (1–417)
- 1 – 403 SID
- 12 – 383 Actin family - PF00022
- 13 – 394 Actin family - SM00268

A — AKIRIN1 – Homo sapiens – ACCESSION: NM_024595.2 – Gene ID: 79647 (1–192)
- 132 – 192 SID
- 145 – 165 Coiled domain

B — AKIRIN2 – Homo sapiens – ACCESSION: NM_018064.3 – Gene ID: 55122 (1–203)
- 136 – 203 SID
- 157 – 177 Coiled domain

D — ATF4 – Homo sapiens – ACCESSION: NP_877962.1 – Gene ID: 468 (1–351)
- 1 – 57 SID
- 268 – 288 Coiled domain
- 296 – 344 Coiled domain
- 276 – 340 Basic-leucine zipper domain - SM00338
- 276 – 337 Basic-leucine zipper domain - PF00170

D — C12orf10 – Homo sapiens – ACCESSION: NP_067653.3 – Gene ID: 60314 (1–376)
- 43 – 258 SID
- 1 – 21 Signal peptide domain
- 47 – 366 Metal-dependent protein hydrolase - PF03690

D — CCNE1 – Homo sapiens – ACCESSION: NM_001322262.1 – Gene ID: 898 (1–395)
- 40 – 227 SID
- 100 – 226 Cyclin, N-terminal - PF00134
- 135 – 220 Cyclin-like - SM00385
- 229 – 351 Cyclin, C-terminal domain - SM01332
- 230 – 350 Cyclin, C-terminal domain - PF02984

D — CDC23 – Homo sapiens – ACCESSION: NM_004661.3 – Gene ID: 8697 (1–597)
- 2 – 469 SID
- 1 – 21 Signal peptide domain
- 33 – 149 Cdc23 - PF04049
- 169 – 202 Tetratricopeptide repeat - SM00028
- 263 – 296 Tetratricopeptide repeat - SM00028
- 331 – 364 Tetratricopeptide repeat - SM00028
- 332 – 363 Tetratricopeptide repeat - PF13181
- 365 – 398 Tetratricopeptide repeat - SM00028
- 372 – 409 TPR repeat - PF13414
- 399 – 432 Tetratricopeptide repeat - SM00028
- 433 – 466 Tetratricopeptide repeat - SM00028
- 510 – 543 Tetratricopeptide repeat - SM00028

D — CLIC3 – Homo sapiens – ACCESSION: NM_004669.2 – Gene ID: 9022 (1–236)
- 56 – 223 SID
- 21 – 86 Glutathione S-transferase, N-terminal - PF13417
- 126 – 207 Glutathione S-transferase, C-terminal do - PF13410

D — CLTCL1 – Homo sapiens – ACCESSION: NM_007098.3 – Gene ID: 8218 (1–1499)
- 1457 – 1499 SID
- 19 – 55 Clathrin, heavy chain, propeller repeat - PF01394
- 150 – 187 Clathrin, heavy chain, propeller repeat - PF01394
- 198 – 234 Clathrin, heavy chain, propeller repeat - PF01394
- 296 – 330 Clathrin, heavy chain, propeller repeat - PF01394
- 331 – 354 Clathrin, heavy chain, linker, core moti - PF09268
- 356 – 421 Clathrin-H-link - PF13838
- 537 – 679 Clathrin, heavy chain/VPS, 7-fold repeat - SM00299
- 542 – 678 Clathrin, heavy chain/VPS, 7-fold repeat - PF00637
- 686 – 828 Clathrin, heavy chain/VPS, 7-fold repeat - SM00299
- 689 – 826 Clathrin, heavy chain/VPS, 7-fold repeat - PF00637
- 833 – 972 Clathrin, heavy chain/VPS, 7-fold repeat - SM00299
- 842 – 968 Clathrin, heavy chain/VPS, 7-fold repeat - PF00637
- 979 – 1119 Clathrin, heavy chain/VPS, 7-fold repeat - PF00637
- 979 – 1124 Clathrin, heavy chain/VPS, 7-fold repeat - SM00299
- 1128 – 1269 Clathrin, heavy chain/VPS, 7-fold repeat - SM00299
- 1132 – 1266 Clathrin, heavy chain/VPS, 7-fold repeat - PF00637
- 1274 – 1420 Clathrin, heavy chain/VPS, 7-fold repeat - SM00299
- 1277 – 1417 Clathrin, heavy chain/VPS, 7-fold repeat - PF00637
- 1423 – 1499 Clathrin, heavy chain/VPS, 7-fold repeat - SM00299
- 1425 – 1499 Clathrin, heavy chain/VPS, 7-fold repeat - PF00637

(1500–1640)
- 1500 – 1640 SID
- 1500 – 1582 Clathrin, heavy chain/VPS, 7-fold repeat - SM00299
- 1500 – 1565 Clathrin, heavy chain/VPS, 7-fold repeat - PF00637

D — CNOT3 – Homo sapiens – ACCESSION: NM_014516.3 – Gene ID: 4849 (1–753)
- 594 – 722 SID
- 38 – 61 Coiled domain
- 132 – 152 Coiled domain
- 3 – 232 CCR4-Not complex component, Not N-termin - PF04065
- 623 – 747 NOT2/NOT3/NOT5 - PF04153

B — ESRRG – Homo sapiens – ACCESSION: NM_001438.3 – Gene ID: 2104 (1–458)
- 168 – 458 SID
- 125 – 196 Zinc finger, nuclear hormone receptor-ty - SM00399
- 127 – 195 Zinc finger, nuclear hormone receptor-ty - PF00105
- 266 – 440 Nuclear hormone receptor, ligand-binding - PF00104
- 270 – 428 Nuclear hormone receptor, ligand-binding - SM00430

D — EXPH5 – Homo sapiens – ACCESSION: NM_015065.2 – Gene ID: 23086 (1–1499)
- 1 – 327 SID
- 14 – 45 Coiled domain

(1500–1989)

D — EZR – Homo sapiens – ACCESSION: NM_003379.4 – Gene ID: 7430 (1–586)
- 269 – 471 SID
- 302 – 462 Coiled domain
- 524 – 544 Coiled domain
- 1 – 206 Band 4.1 domain - SM00295
- 10 – 69 FERM, N-terminal - PF09379
- 93 – 206 FERM central domain - PF00373
- 210 – 298 FERM, C-terminal PH-like domain - PF09380
- 210 – 299 FERM, C-terminal PH-like domain - SM01196
- 338 – 586 Ezrin/radixin/moesin, C-terminal - PF00769

D — FNTA – Homo sapiens – ACCESSION: NM_002027.2 – Gene ID: 2339 (1–379)
- 1 – 353 SID
- 320 – 354 Coiled domain
- 117 – 142 Protein prenyltransferase, alpha subunit - PF01239
- 151 – 178 Protein prenyltransferase, alpha subunit - PF01239
- 185 – 211 Protein prenyltransferase, alpha subunit - PF01239
- 219 – 246 Protein prenyltransferase, alpha subunit - PF01239
- 259 – 285 Protein prenyltransferase, alpha subunit - PF01239

D — HEG1 – Homo sapiens – ACCESSION: NM_020733.1 – Gene ID: 57493 (1–1381)
- 876 – 1188 SID
- 1248 – 1270 Transmembrane domain
- 1 – 29 Signal peptide domain
- 985 – 1023 EGF-like calcium-binding domain - SM00179
- 988 – 1023 EGF-like domain - SM00181
- 989 – 1020 EGF-like domain - PF00008
- 1025 – 1063 EGF-like calcium-binding domain - SM00179
- 1025 – 1056 EGF-like calcium-binding domain - PF07645
- 1028 – 1063 EGF-like domain - SM00181
- 1183 – 1231 EGF-like domain - SM00181

E — HMG20A – Homo sapiens – ACCESSION: NP_001291433.1 – Gene ID: 10363 (1–347)
- 34 – 347 SID
- 153 – 173 Coiled domain
- 229 – 270 Coiled domain
- 102 – 172 High mobility group box domain - SM00398
- 103 – 170 High mobility group box domain - PF00505

D — HNRPF – Homo sapiens – ACCESSION: NM_001098208.1 – Gene ID: 3185 (1–415)
- 190 – 415 SID
- 12 – 86 RNA recognition motif domain - SM00360
- 112 – 184 RNA recognition motif domain - SM00360
- 114 – 181 RNA recognition motif domain - PF00076
- 255 – 290 Zinc finger, CHHC-type - PF08080
- 290 – 360 RNA recognition motif domain - SM00360
- 293 – 356 RNA recognition motif domain - PF00076

D — IFT57 – Homo sapiens – ACCESSION: NM_018010.3 – Gene ID: 55081 (1–429)
- 66 – 331 SID
- 304 – 331 Coiled domain
- 339 – 366 Coiled domain
- 44 – 401 Intra-flagellar transport protein 57 - PF10498

DomSight: PLA_RP6_hgx4913v1 vs. Human Placenta_RP6 (14 Nov 2017)

(Bait plasmid(s): hgx4913v1_pB27)

Legend

- *Bait fragment*
- *SID fragment*
- *Pfam or SMART domain*
- *Transmembrane domain (TMHMM)*
- *Coiled-coil domain (Ncoils)*
- *Signal peptide (SignalP)*

SID: Selected Interaction Domain
It is the amino acid sequence shared by all prey fragments matching the same reference protein. SIDs have been found in numerous cases to correspond to an identified structural or functional domain.
Protein Display:
Proteins larger than 1500 aa are shown in sections of 1499 aa each. Only sections containing bait fragments, SIDs or predicted functional and structural domains are represented.

Legend

D — INTS2, Homo sapiens, ACCESSION: NM_001351695.1, Gene ID: 57508 (1–1196)
- 670 1196 SID
- 26 1129 Integrator complex subunit 2 - PF14750

D — IRF6, Homo sapiens, ACCESSION: NM_001206696.1, Gene ID: 3664 (1–372)
- 105 362 SID
- 128 312 Interferon regulatory factor-3 - SM01243
- 129 311 Interferon regulatory factor-3 - PF10401

D — KLHL5, Homo sapiens, ACCESSION: NM_001171654.1, Gene ID: 51088 (1–568)
- 1 174 SID
- 24 129 BTB/POZ domain - PF00651
- 33 130 BTB/POZ domain - SM00225
- 135 236 BTB/Kelch-associated - SM00875
- 135 234 BTB/Kelch-associated - PF07707
- 280 314 Kelch repeat type 1 - PF01344
- 281 327 Kelch repeat type 1 - SM00612
- 316 361 Kelch repeat type 1 - PF01344
- 328 374 Kelch repeat type 1 - SM00612
- 364 408 Kelch repeat type 1 - PF01344
- 375 421 Kelch repeat type 1 - SM00612
- 410 455 Kelch repeat type 1 - PF01344
- 422 468 Kelch repeat type 1 - SM00612
- 457 508 Kelch repeat type 1 - PF01344
- 469 521 Kelch repeat type 1 - SM00612
- 513 554 Kelch repeat type 1 - PF01344
- 522 568 Kelch repeat type 1 - SM00612

E — KPNA4, Homo sapiens, ACCESSION: NM_002268.4, Gene ID: 3840 (1–521)
- 1 448 SID
- 11 93 Importin-alpha, importin-beta-binding do - PF01749
- 103 144 Armadillo - SM00185
- 104 144 Armadillo - PF00514
- 146 184 Armadillo - PF00514
- 146 186 Armadillo - SM00185
- 188 229 Armadillo - SM00185
- 189 229 Armadillo - PF00514
- 232 271 Armadillo - SM00185
- 233 270 Armadillo - PF00514
- 273 313 Armadillo - SM00185
- 274 312 Armadillo - PF00514
- 315 355 Armadillo - PF00514
- 315 355 Armadillo - SM00185
- 357 396 Armadillo - PF00514
- 357 397 Armadillo - SM00185
- 400 440 Armadillo - SM00185
- 402 438 Armadillo - PF00514
- 451 498 Atypical Arm repeat - PF16186

D — LIMS1, Homo sapiens, ACCESSION: NM_001193485.2, Gene ID: 3987 (1–387)
- 22 343 SID
- 71 124 Zinc finger, LIM-type - SM00132
- 72 127 Zinc finger, LIM-type - PF00412
- 132 183 Zinc finger, LIM-type - SM00132
- 133 187 Zinc finger, LIM-type - PF00412
- 196 246 Zinc finger, LIM-type - SM00132
- 197 248 Zinc finger, LIM-type - PF00412
- 254 305 Zinc finger, LIM-type - SM00132
- 255 309 Zinc finger, LIM-type - PF00412
- 313 365 Zinc finger, LIM-type - SM00132
- 314 368 Zinc finger, LIM-type - PF00412

D — LNX1, Homo sapiens, ACCESSION: NP_001119800.1, Gene ID: 84708 (1–728)
- 180 610 SID
- 41 78 Zinc finger, RING-type - SM00184
- 41 77 Zinc finger, C3HC4 RING-type - PF00097
- 276 356 PDZ domain - PF00595
- 284 359 PDZ domain - SM00228
- 382 460 PDZ domain - PF00595
- 391 464 PDZ domain - SM00228
- 508 588 PDZ domain - PF00595
- 516 593 PDZ domain - SM00228
- 639 720 PDZ domain - PF00595
- 647 724 PDZ domain - SM00228

B — PITPNA, Homo sapiens, ACCESSION: NM_006224.3, Gene ID: 5306 (1–270)
- 156 270 SID
- 240 260 Coiled domain
- 3 251 Phosphatidylinositol transfer protein - PF02121

D — PKM, Homo sapiens, ACCESSION: NP_002645.3, Gene ID: 5315 (1–531)
- 324 531 SID
- 43 394 Pyruvate kinase, barrel - PF00224
- 410 528 Pyruvate kinase, C-terminal - PF02887

E — PSAP, Homo sapiens, ACCESSION: NP_001035930.1, Gene ID: 5660 (1–527)
- 189 512 SID
- 1 16 Signal peptide domain
- 138 158 Coiled domain
- 21 54 Saposin A-type domain - SM00162
- 23 54 Saposin A-type domain - PF02199
- 61 138 Saposin B type domain - SM00741
- 62 98 Saposin-like type B, region 1 - PF05184
- 106 138 Saposin B type, region 2 - PF03489
- 196 234 Saposin-like type B, region 1 - PF05184
- 196 274 Saposin B type domain - SM00741
- 239 274 Saposin B type, region 2 - PF03489
- 316 391 Saposin B type domain - SM00741
- 317 353 Saposin-like type B, region 1 - PF05184
- 359 391 Saposin B type, region 2 - PF03489
- 410 485 Saposin B type domain - SM00741
- 411 447 Saposin-like type B, region 1 - PF05184
- 452 485 Saposin B type, region 2 - PF03489
- 494 527 Saposin A-type domain - SM00162
- 496 527 Saposin A-type domain - PF02199

A — RNF10, Homo sapiens, ACCESSION: NM_014868.4, Gene ID: 9921 (1–811)
- 107 373 SID
- 224 266 Zinc finger, RING-type - PF13639
- 225 266 Zinc finger, RING-type - SM00184

D — SCFD1, Homo sapiens, ACCESSION: NP_057190.2, Gene ID: 23256 (1–642)
- 146 367 SID
- 338 358 Coiled domain
- 44 635 Sec1-like protein - PF00995

D — SERPINE2, Homo sapiens, ACCESSION: NM_006216.3, Gene ID: 5270 (1–398)
- 270 398 SID
- 1 19 Signal peptide domain
- 34 398 Serpin domain - PF00079
- 36 398 Serpin domain - SM00093

A — SF3A1, Homo sapiens, ACCESSION: NM_005868.1, Gene ID: 10291 (1–793)
- 154 297 SID
- 250 270 Coiled domain
- 50 101 SWAP/Surp - PF01805
- 50 103 SWAP/Surp - SM00648
- 164 217 SWAP/Surp - SM00648
- 164 215 SWAP/Surp - PF01805
- 233 472 Splicing factor 3A subunit 1 - PF12230
- 715 786 Ubiquitin domain - SM00213
- 722 788 Ubiquitin domain - PF00240

D — SF3B1, Homo sapiens, ACCESSION: NP_036565.2, Gene ID: 23451 (1–1304)
- 566 862 SID
- 1 28 Coiled domain
- 847 874 Coiled domain
- 330 451 Splicing factor 3B subunit 1 - PF08920

E — SNF8, Homo sapiens, ACCESSION: NP_009172.2, Gene ID: 11267 (1–258)
- 1 208 SID
- 33 53 Coiled domain
- 6 225 EAP30 - PF04157

F — SNRNP70 var2, Homo sapiens, ACCESSION: NM_001301069.1, Gene ID: 6625 (1–428)
- 29 159 SID
- 280 300 Coiled domain
- 3 94 U1 small nuclear ribonucleoprotein of 70 - PF12220
- 104 177 RNA recognition motif domain - SM00360
- 105 174 RNA recognition motif domain - PF00076

D — SPG21, Homo sapiens, ACCESSION: NP_001121362.1, Gene ID: 51324 (1–281)
- 1 281 SID

DomSight: PLA_RP6_hgx4913v1 vs. Human Placenta_RP6 (14 Nov 2017)

(Bait plasmid(s): hgx4913v1_pB27)

Legend	
Bait fragment	*SID: Selected Interaction Domain*
SID fragment	*It is the amino acid sequence shared by all prey fragments matching the same reference protein. SIDs have been found in numerous cases to correspond to an identified structural or functional domain.*
Pfam or SMART domain	*Protein Display:*
Transmembrane domain (TMHMM)	*Proteins larger than 1500 aa are shown in sections of 1499 aa each. Only sections containing bait fragments, SIDs or predicted functional and structural domains are represented.*
Coiled-coil domain (Ncoils)	
Signal peptide (SignalP)	

Legend

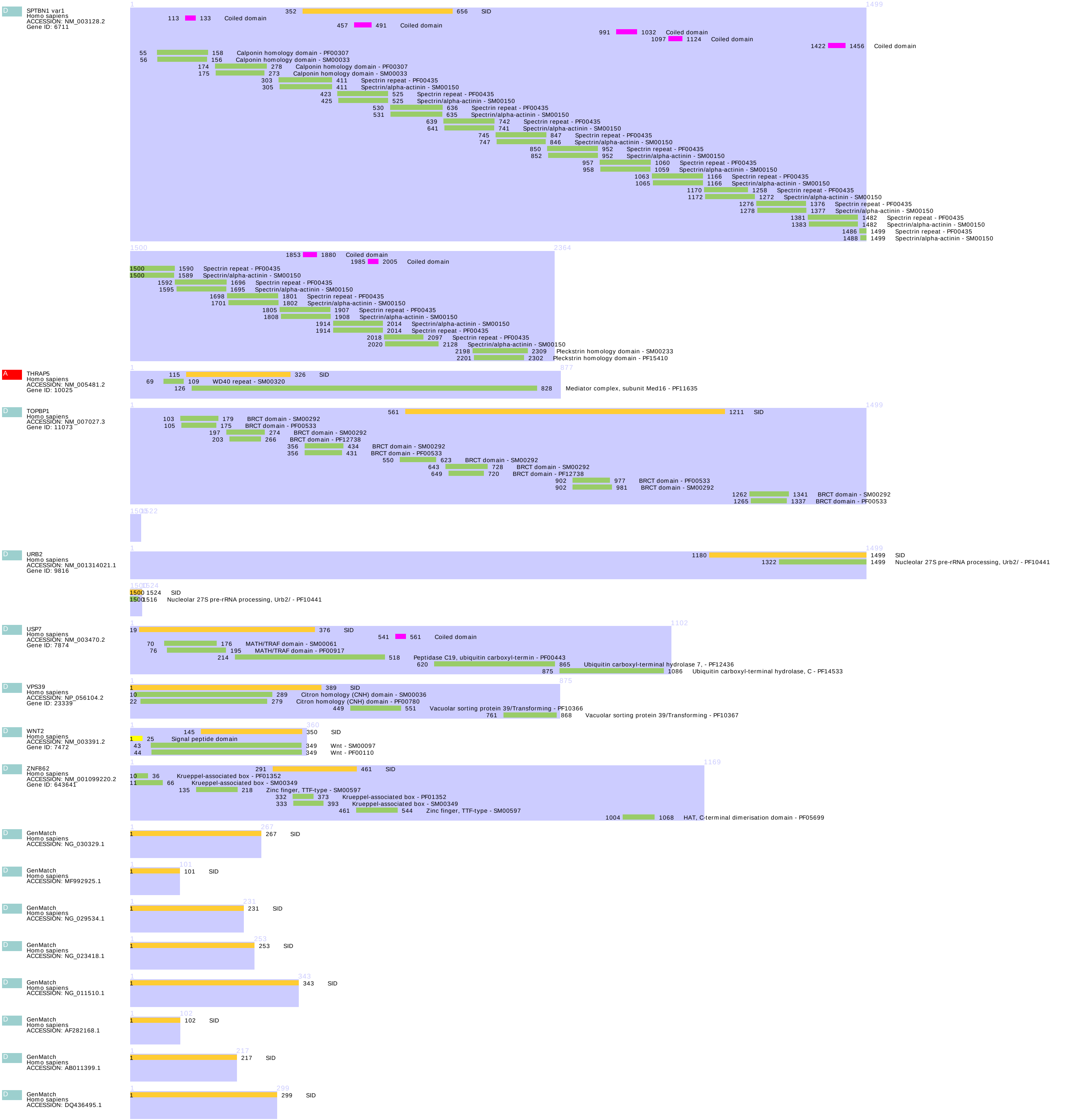

HYBRIGENICS SERVICES

DomSight: PLA_RP6_hgx4913v1 vs. Human Placenta_RP6 (14 Nov 2017)

(Bait plasmid(s): hgx4913v1_pB27)

Bait fragment
SID fragment
Pfam or SMART domain
Transmembrane domain (TMHMM)
Coiled-coil domain (Ncoils)
Signal peptide (SignalP)

SID: Selected Interaction Domain
It is the amino acid sequence shared by all prey fragments matching the same reference protein. SIDs have been found in numerous cases to correspond to an identified structural or functional domain.
Protein Display:
Proteins larger than 1500 aa are shown in sections of 1499 aa each. Only sections containing bait fragments, SIDs or predicted functional and structural domains are represented.

Legend

D GenMatch
Homo sapiens
ACCESSION: U34879.1

1 181
1 181 SID

Figure S1. Annotation of the AKR2-protein interacting domains. The annotations were done using DomSight (Hybrigenics Services) to compare the bait fragment and the selected interacting domain (SID) of the prey proteins with the functional and structural domains generated by Pfam, SMART, TMHMM, SignalP, and COILS algorithms.

>NM_058903.6 *Caenorhabditis elegans* Akirin

```
ATG GCT TGC GGA CTC GCA CTG AAA AGA CCT CTG CAA CAC GAG TAT GAA TCT TTT TTA
 M   A   C   G   L   A   L   K   R   P   L   Q   H   E   Y   E   S   F   L
 M  DRL  T  SL  LS  DR  LSF RD  RFS MRL LSF MF  LT  FSR LSTD FS  SL  FL LLS

ACT GAT GAG ACA TAC AAC GGA GAA GCA AAG CGA GCC AGA ACG CAA TGC CCT CCT TTC
 T   D   E   T   Y   N   G   E   A   K   R   A   R   T   Q   C   P   P   F
FML DTD FSR FM  LS  RM  SL  FS  DR  RDR  R DRMR RFS FMR MF   T  MRL MRL  F

CGT GCT CAA ATG GGA ACT ATT GCC  GCT ACA CTG CCG TCA ACC AGC ACA TTT GCT CAG
 R   A   Q   M   G   T   I   A    A   T   L   P   S   T   S   T   F   A   Q
RTL DRR  MF  M  SL  FML TDF DRMR DRL  FM LSF MRR  S FMMR SDR FM   FL DRL MFR

AAG TTC AAA GAA CAG GAG GAA AGC GTC TTC CAA GCA GCT ACA CTT ATG ACT CGT TTG
 K   F   K   E   Q   E   E   S   V   F   Q   A   A   T   L   M   T   R   L
RDR  F  RD  FS  MFR FSR FS  SDR TLS  F  MF  DR  DRL FM  LF   M  FML RTL LSF

TCT CGA AAT CAG CTC AAA ACA TAT CTC AGT TCG GAA GTG AAA AAC CTG CGC AAG CGA
 S   R   N   Q   L   K   T   Y   L   S   S   E   V   K   N   L   R   K   R
SL   R RMTD MFR  LS  RD  FM LSTD LS STL  SR  FS TLSF RD  RM LSF RDR RDR  R

AAA GCC ATT CCA CGC AGC AAT GAC TTT GAT GAT GAT GGA GAT CAA AGA GGA GAC GGA
 K   A   I   P   R   S   N   D   F   D   D   D   G   D   Q   R   G   D   G
RD DRMR TDF MR  RDR SDR RMTD D   FL DTD DTD DTD SL  DTD  MF  RFS SL  D   SL

TGC AGC TCA AAC TAC TCA AAA GCT TAC AGA GCT CCT TCT TCT CCC AAG TCT GGA TCG
 C   S   S   N   Y   S   K   A   Y   R   A   P   S   S   P   K   S   G   S
 T  SDR  S  RM  LS   S  RD  DRL LS  RFS DRL MRL SL  SL MRMR RDR  SL  SL  SR

GAT TCC GAG GGT GAA GCA CCA TCA ACA TCT GTG ACT GAT CGT TCC AGC GCC AAG AGG
 D   S   E   G   E   A   P   S   T   S   V   T   D   R   S   S   A   K   R
DTD SMR FSR SLTL FS DR  MR   S  FM  SL TLSF FML DTD RTL SMR SDR DRMR RDR RSL

GAA TTT ACA ATG GCA AAT GTT CAA ATG ATA TGC GAA CGT CTG CTC AAA CAA CAA GAA
 E   F   T   M   A   N   V   Q   M   I   C   E   R   L   L   K   Q   Q   E
FS  FL  FM   M  DR RMTD TLF MF   M  TD   T  FS  RTL LSF  LS  RD  MF  MF  FS

ATC CGT CTG AGA AAT GAG TTC GAA ATG GTT CTG ACT AAG AAG CTT GAT GAG CAA CAC
 I   R   L   R   N   E   F   E   M   V   L   T   K   K   L   D   E   Q   H
TDS RTL LSF RFS RMTD FSR F  FS   M  TLF LSF FML RDR RDR  LF DTD FSR  MF  LT
```

```
CAG CAA TAC GTC CAA TTT GCT GCC GAG CAG CTG AAC TCC AAA TGT GTG AGC ACT
 Q   Q   Y   V   Q   F   A   A   E   Q   L   N   S   K   C   V   S   T
MFR MF  LS TLS  MF  FL  DRL DRMR FSR MFR LSF RM SMR RD TTL TLSF SDR FML

GGG   GAT GAC TAT TCC TAT TCG TAC CTC TCC TAA
 G     D   D   Y   S   Y   S   Y   L   S
SLSL  DTD  D LSTD SMR LSTD SR LS  LS  SMR STOP
```

Caenorhabditis elegans Akirin

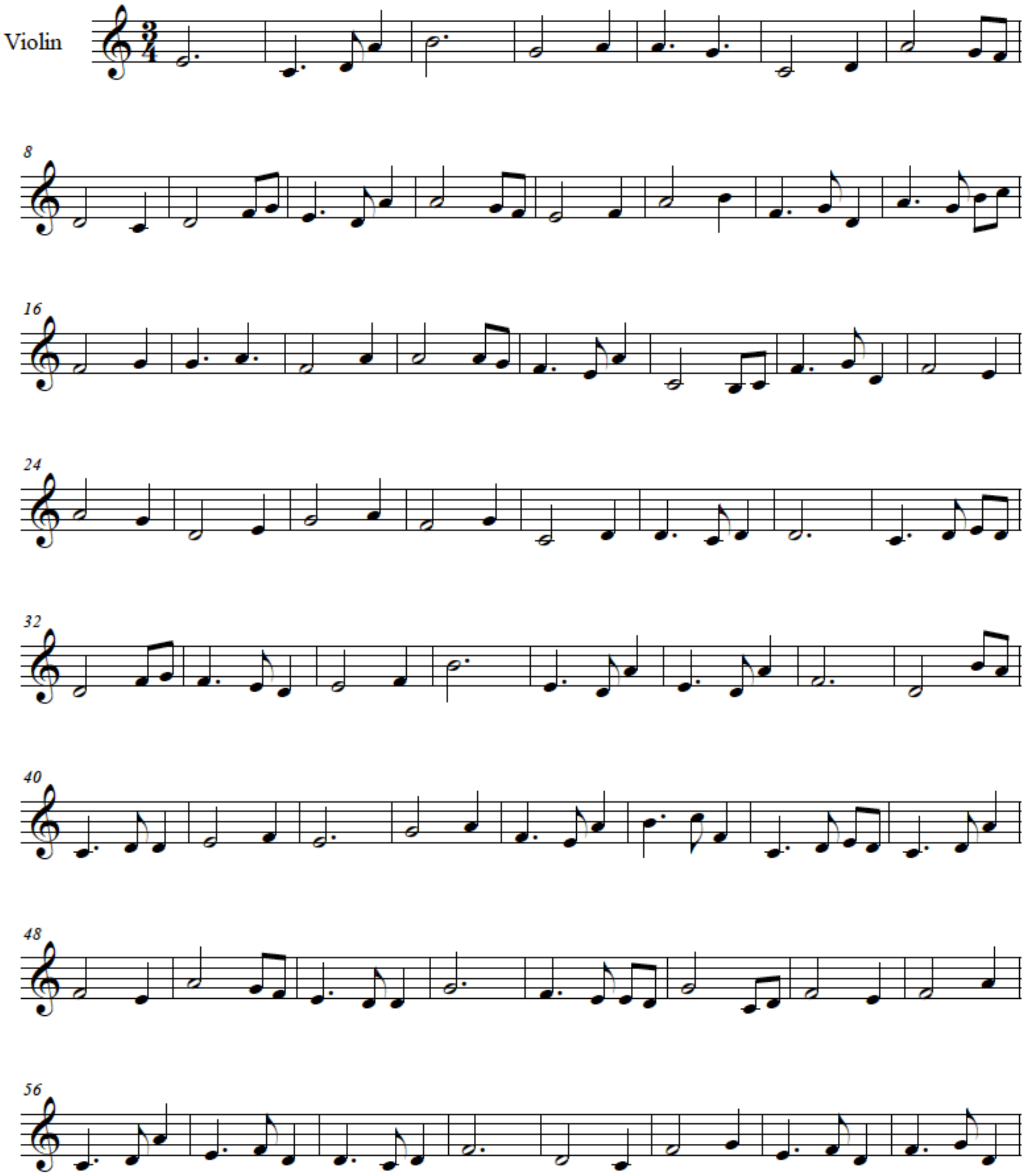

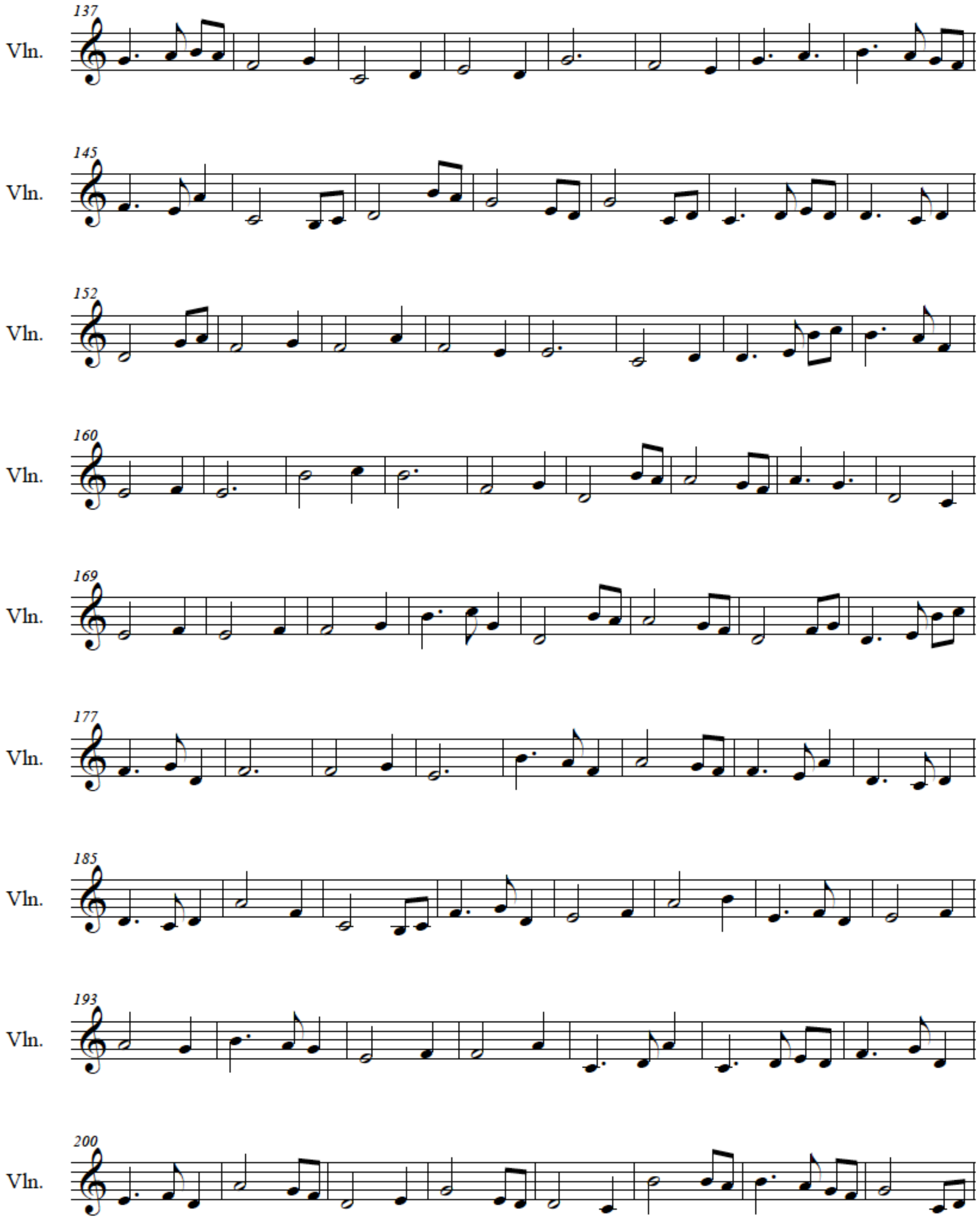
137
Vln.
145
Vln.
152
Vln.
160
Vln.
169
Vln.
177
Vln.
185
Vln.
193
Vln.
200
Vln.

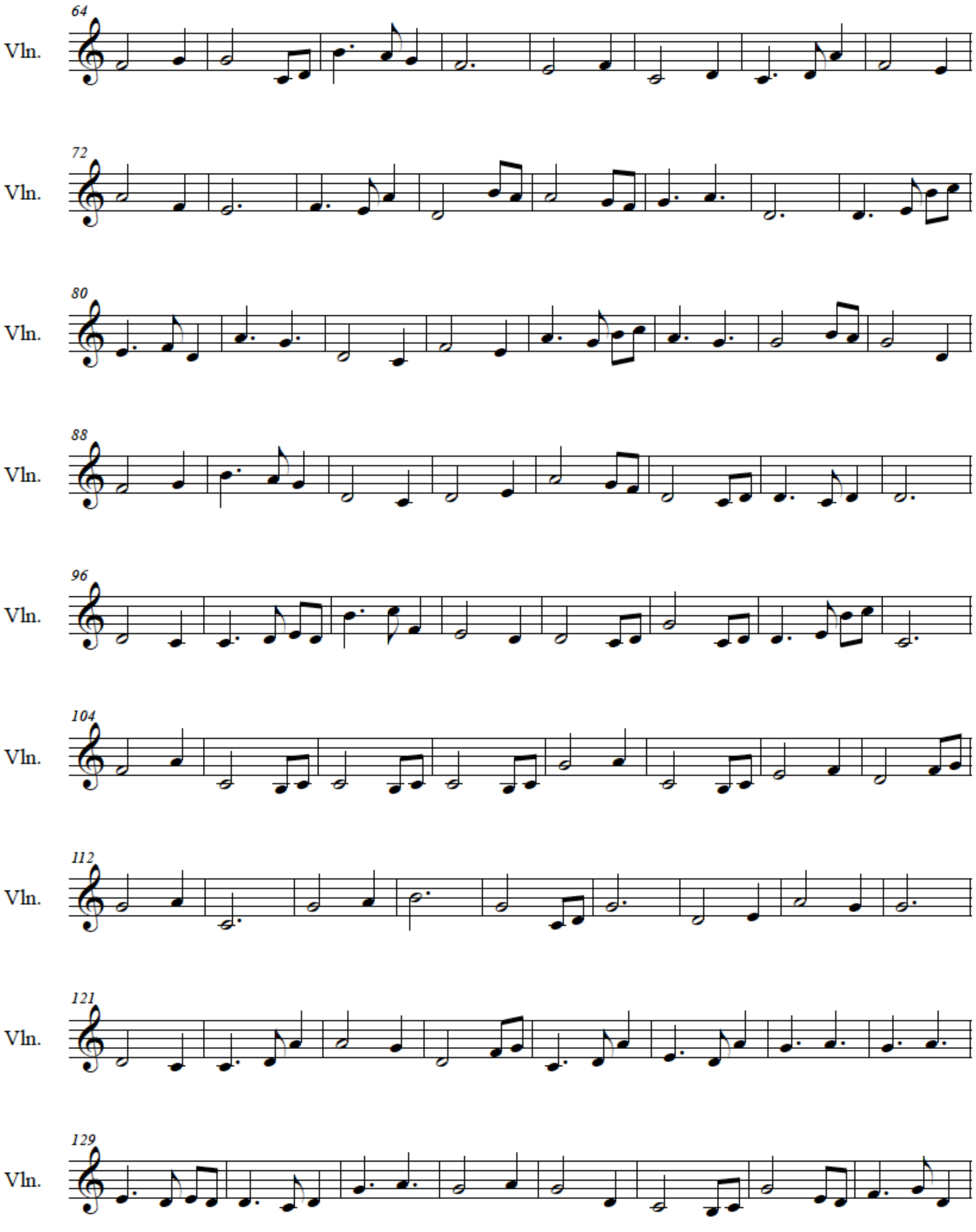
64
Vln.
72
Vln.
80
Vln.
88
Vln.
96
Vln.
104
Vln.
112
Vln.
121
Vln.
129
Vln.

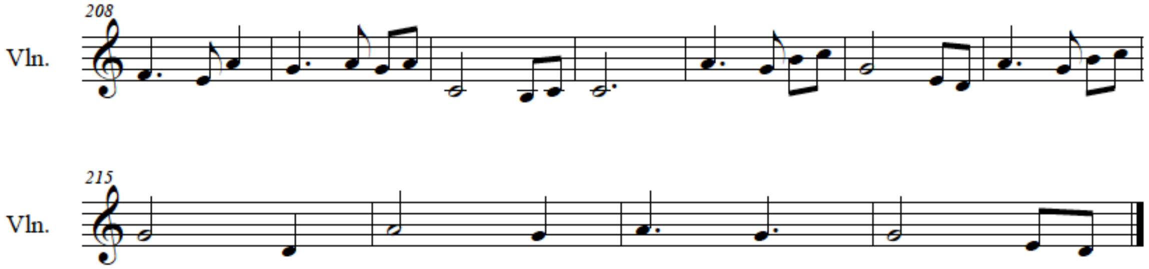
208
Vln.
215
Vln.

>NM_139856.4 *Drosophila melanogaster* Akirin

```
ATG GCC TGT GCA ACC CTG AAA CGA GCC CTA GAC TGG GAG TCG ATG AAC CAG CGG CCT
 M   A   C   A   T   L   K   R   A   L   D   W   E   S   M   N   Q   R   P
 M DRMR TTL DR FMMR LSF RD   R DRMR  L   D  SF  FSR  SR  M  RM  MFR RSL MRL

CCG AAG CGC CGG CGT TGC AAT CCC TTT GGC CAG GCT GGG  AGC AAT GCA GGT CCA GCG
 P   K   R   R   R   C   N   P   F   G   Q   A   G    S   N   A   G   P   A
MRR RDR RDR RSL RTL T RMTD MRMR FL SLDR MFR DRL SLSL SDR RMTD DR SLTL MR DRR

TCG CCA TCC CGC GAC GGT CCC AGC ACC TCG GCT GGT CTG CCC CAC ACG CCC  AGC AAC
 S   P   S   R   D   G   P   S   T   S   A   G   L   P   H   T   P    S   N
SR MR SMR RDR  D SLTL MRMR SDR FMMR SR DRL SLTL LSF MRMR LT FMR MRMR SDR  RM

CGA TTC GCC AAG GAT AGC ACC GAA CCT AGT CCG TTC AGT GAG TCG TCG CTG GCC AAA
 R   F   A   K   D   S   T   E   P   S   P   F   S   E   S   S   L   A   K
 R   F DRMR RDR DTD SDR FMMR FS MRL STL MRR  F  STL FSR SR  SR  LSF DRMR RD

ATG TCA CCA GAC AAA ATG GCC GAG AGC TTG TGC AAT GAG ATC AAG AGA CTG CAC AAG
 M   S   P   D   K   M   A   E   S   L   C   N   E   I   K   R   L   H   K
 M   S  MR   D  RD   M DRMR FSR SDR LSF  T RMTD FSR TDS RDR RFS LSF LT  RDR

CGC AAA CAG CTG CCG ATC ACT TCG TCG GCC TTG GAA CGC ATG CAG GAT TCG GAG TCC
 R   K   Q   L   P   I   T   S   S   A   L   E   R   M   Q   D   S   E   S
RDR RD  MFR LSF MRR TDS FML SR  SR DRMR LSF FS  RDR  M  MFR DTD SR  FSR SMR

AGC GGA TCG GAG ATG GGT CCA GAG AGT CCG CGC CGC CCG GAC AGT CCA CAG AAC CTG
 S   G   S   E   M   G   P   E   S   P   R   R   P   D   S   P   Q   N   L
SDR SL  SR  FSR  M SLTL MR  FSR STL MRR RDR RDR MRR  D  STL MR  MFR RM  LSF

ATG CGC CAC GGC GAA AAG GCC CTG TTC ACG TTC AAG CAG GTG CAG CTC ATT TGC GAG
 M   R   H   G   E   K   A   L   F   T   F   K   Q   V   Q   L   I   C   E
 M  RDR LT  SLDR FS RDR DRMR LSF F  FMR  F  RDR MFR TLSF MFR LS TDF  T  FSR

AGC ATG ATC AAG GAG CGC GAG AAT CAG CTA AGG GAG CGC TAC GAG TCC GTG CTG ACC
 S   M   I   K   E   R   E   N   Q   L   R   E   R   Y   E   S   V   L   T
SDR M TDS RDR FSR RDR FSR RMTD MFR L  RSL FSR RDR  LS FSR SMR TLSF LSF FMMR

ACC  AAG CTG GCC GAG CAG TAC GAT GCC TTT GTC AAG TTC ACA TAT  GAT CAG ATA CAG
 T    K   L   A   E   Q   Y   D   A   F   V   K   F   T   Y    D   Q   I   Q
FMMR RDR LSF DRMR FSR MFR LS DTD DRMR FL TLS RDR  F   FM LSTD DTD MFR  TD MFR
```

```
CGT CGC TAC GAG  GCA GCG CCT AGC TAC CTG TCG TAA
 R   R  Y    E    A   A   P   S   Y   L   S
RTL RDR LS  FSR   DR DRR MRL SDR  LS LSF  SR STOP
```

Drosophila melanogaster Akirin

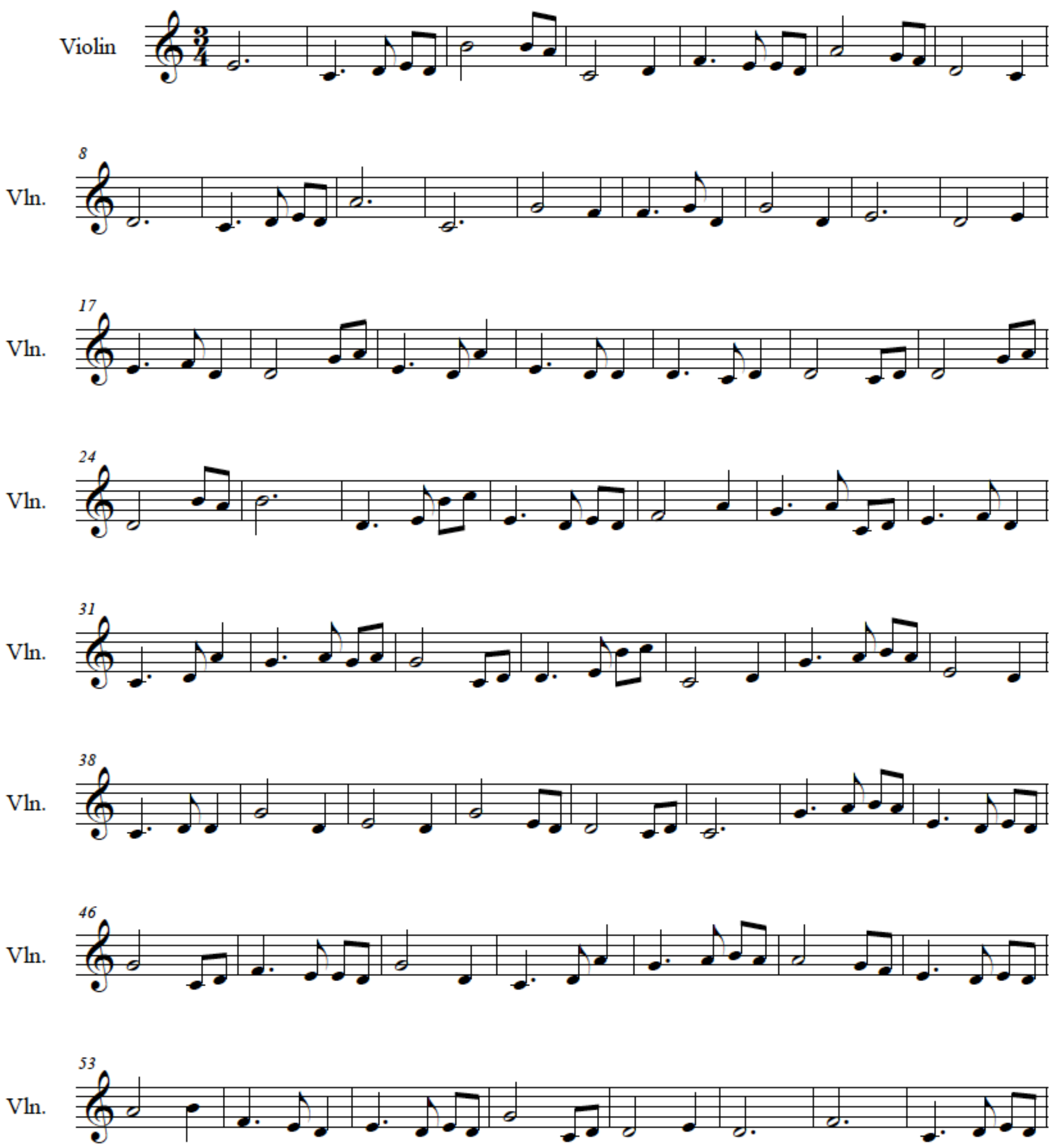

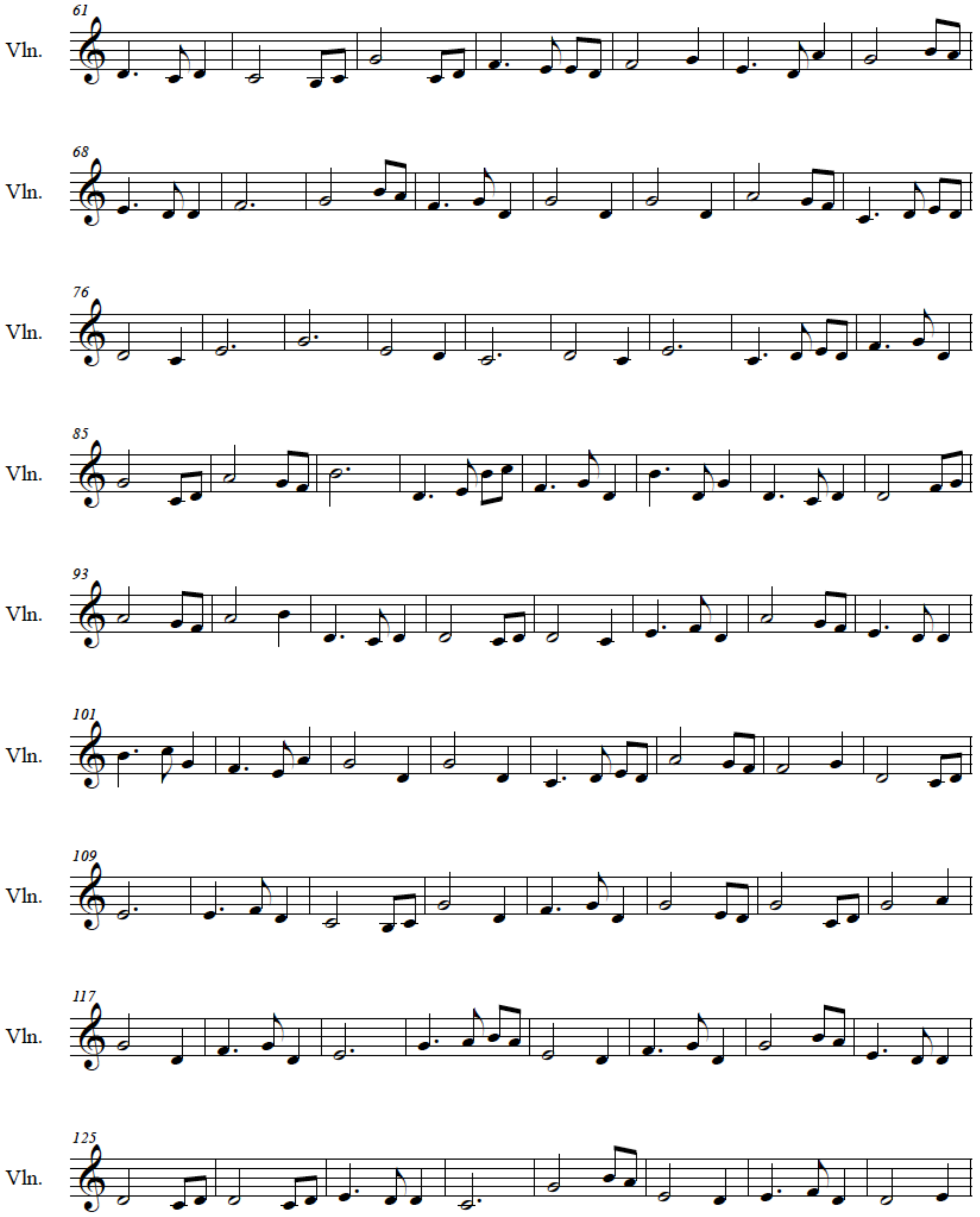
61
Vln.
68
Vln.
76
Vln.
85
Vln.
93
Vln.
101
Vln.
109
Vln.
117
Vln.
125
Vln.

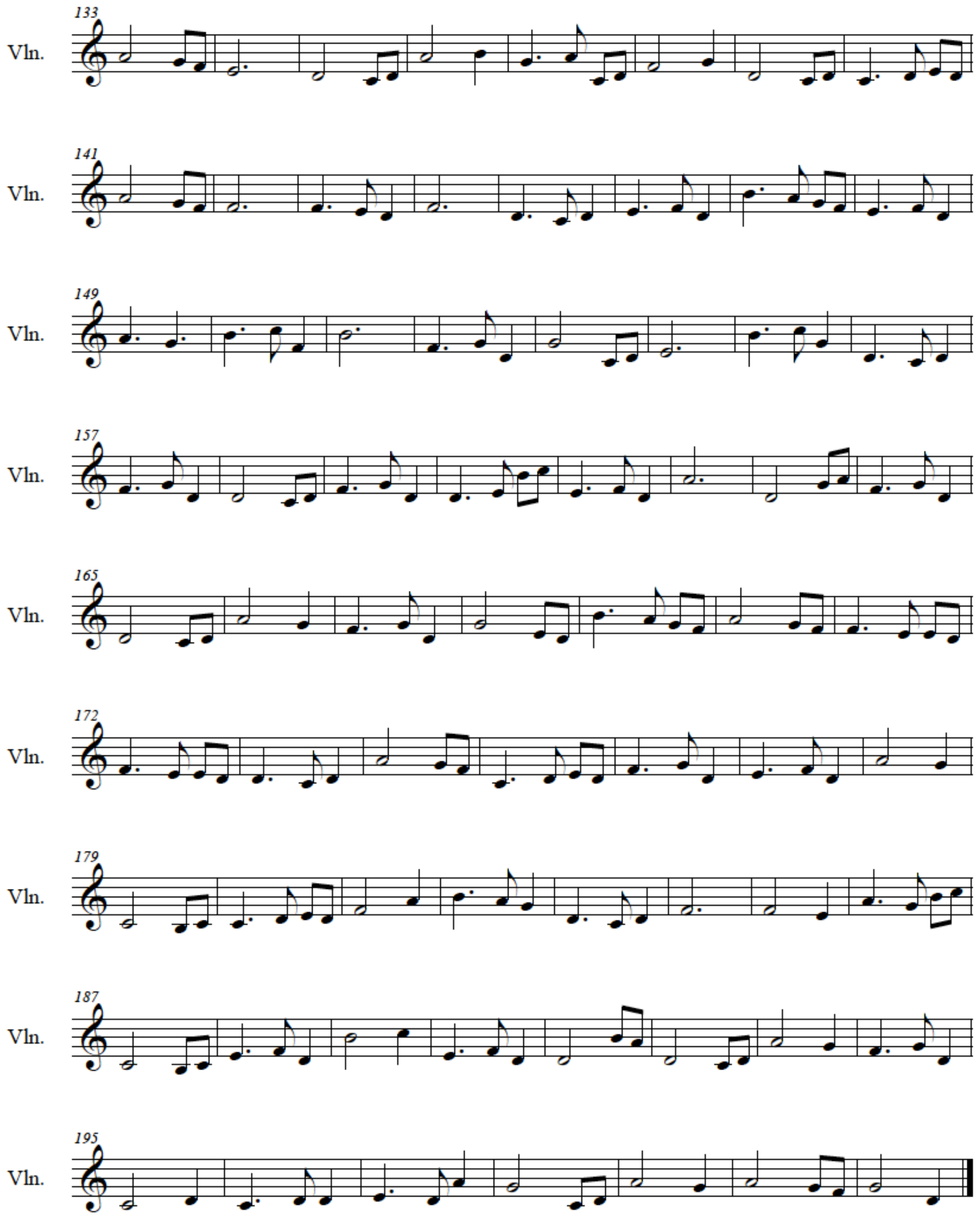
133
Vln.
141
Vln.
149
Vln.
157
Vln.
165
Vln.
172
Vln.
179
Vln.
187
Vln.
195
Vln.

>AY652654.1 *Ixodes scapularis* Akirin (Subolesin)

```
ATG GCT TGC GCA ACA TTA AAG CGA ACA CAC GAT TGG GAT CCG CTG CAT AGT CCA AAC
 M   A   C   A   T   L   K   R   T   H   D   W   D   P   L   H   S   P   N
 M  DRL  T   DR FM  LLS RDR  R   FM LT  DTD  SF DTD MRR LSF LTM STL MR  RM

GGA AGA TCG CCC AAA CGA CGG AGA TGT ATG CCT TTG TCG GTC ACA CAA GCA GCG ACT
 G   R   S   P   K   R   R   R   C   M   P   L   S   V   T   Q   A   A   T
SL  RFS  SR MRMR RD  R  RSL RFS TTL  M  MRL LSF SR  TLS FM  MF  DR  DRR FML

CCC CCA ACA AGG GCA CAC CAA ATC AAC CCT TCA CCC TTC GGT GAA GTG CCA CCG AAA
 P   P   T   R   A   H   Q   I   N   P   S   P   F   G   E   V   P   P   K
MRMR MR FM  RSL DR  LT  MF  TDS RM  MRL  S  MRMR F  SLTL FS TLSF MR MRR  RD

TTA ACT TCA GAG GAG ATA GCG GCC AAC ATT CGG GAG GAA ATG CGA CGT CTG CAG CGG
 L   T   S   E   E   I   A   A   N   I   R   E   E   M   R   R   L   Q   R
LLS FML  S  FSR FSR TD  DRR DRMR RM TDF RSL FSR FS   M   R  RTL LSF MFR RSL

CGC AAG CAG CTC TGC TTC TCG TCT CCC  CTG GAG TCG GGC TCC CCG TCG GCG ACT CCC
 R   K   Q   L   C   F   S   S   P    L   E   S   G   S   P   S   A   T   P
RDR RDR MFR  LS  T   F  SR    SL MRMR LSF FSR SR SLDR SMR MRR  SR DRR FML MRMR

CCT GCG GCC GAT TGC GGA CCA GCC TCC CCC ACG GGC CTG TCC CCC GGG  GGC  CTG CTG
 P   A   A   D   C  G  P   A   S   P    T   G   L   S   P    G    G   L   L
MRL DRR DRMR DTD T SL MR DRMR SMR MRMR FMR SLDR LSF SMR MRMR SLSL SLDR LSFLSF

TCG CCC GTG CGC AGG GAC CAA CCC CTC TTC ACC TTC CGC CAG GTG GGG CTC ATC TGC
 S   P   V   R   R   D   Q   P   L   F   T   F   R   Q   V   G   L   I   C
SR MRMR TLSF RDR RSL D  MF MRMR  LS  F FMMR F  RDR MFR TLSF SLSL LS TDS  T

GAG CGC ATG ATG AAG GAG CGC GAG AGC CAG ATA CGC GAC GAG TAC GAC CAC GTT CTG
 E   R   M   M   K   E   R   E   S   Q   I   R   D   E   Y   D   H   V   L
FSR RDR  M   M  RDR FSR RDR FSR SDR MFR TD  RDR  D  FSR LS   D  LT  TLF LSF

TCT GCC AAG CTG GCA GAG CAG TAC GAC ACA TTT GTC AAG TTT ACG TAC GAC CAA ATT
 S   A   K   L   A   E   Q   Y   D   T   F   V   K   F   T   Y   D   Q   I
SL DRMR RDR LSF  DR FSR MFR LS   D  FM  FL  TLS RDR FL  FMR LS   D  MF  TDF

CAG AAG CGG TTT GAG GGT GCC ACT CCA AGC TAT TTG TCA TAA
 Q   K   R   F   E   G   A   T   P   S   Y   L   S
MFR RDR RSL FL FSR SLTL DRMR FML MR SDR LST DLSF S  STOP
```

***Ixodes scapularis* Akirin (Subolesin)**

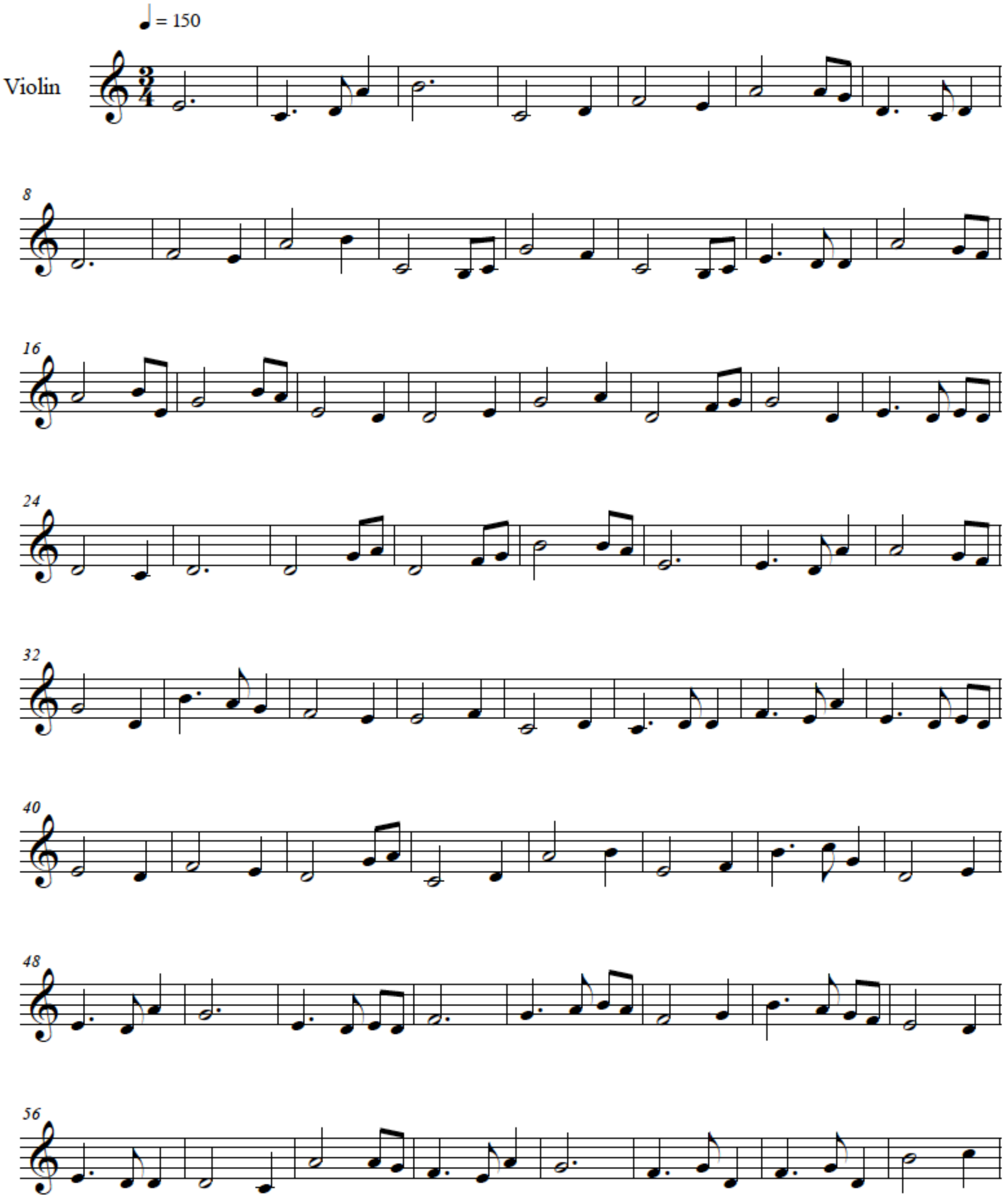

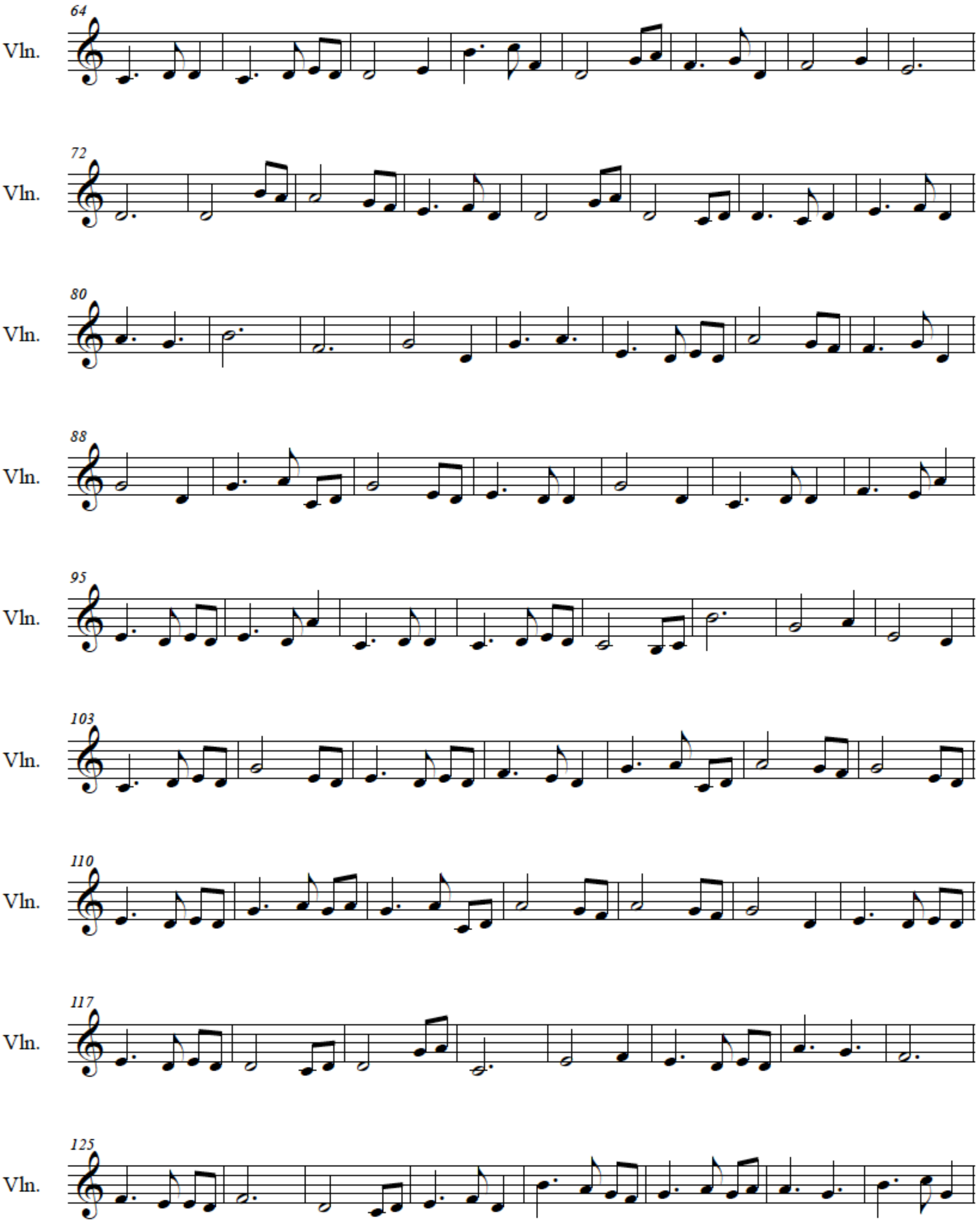
64
Vln.
72
Vln.
80
Vln.
88
Vln.
95
Vln.
103
Vln.
110
Vln.
117
Vln.
125
Vln.

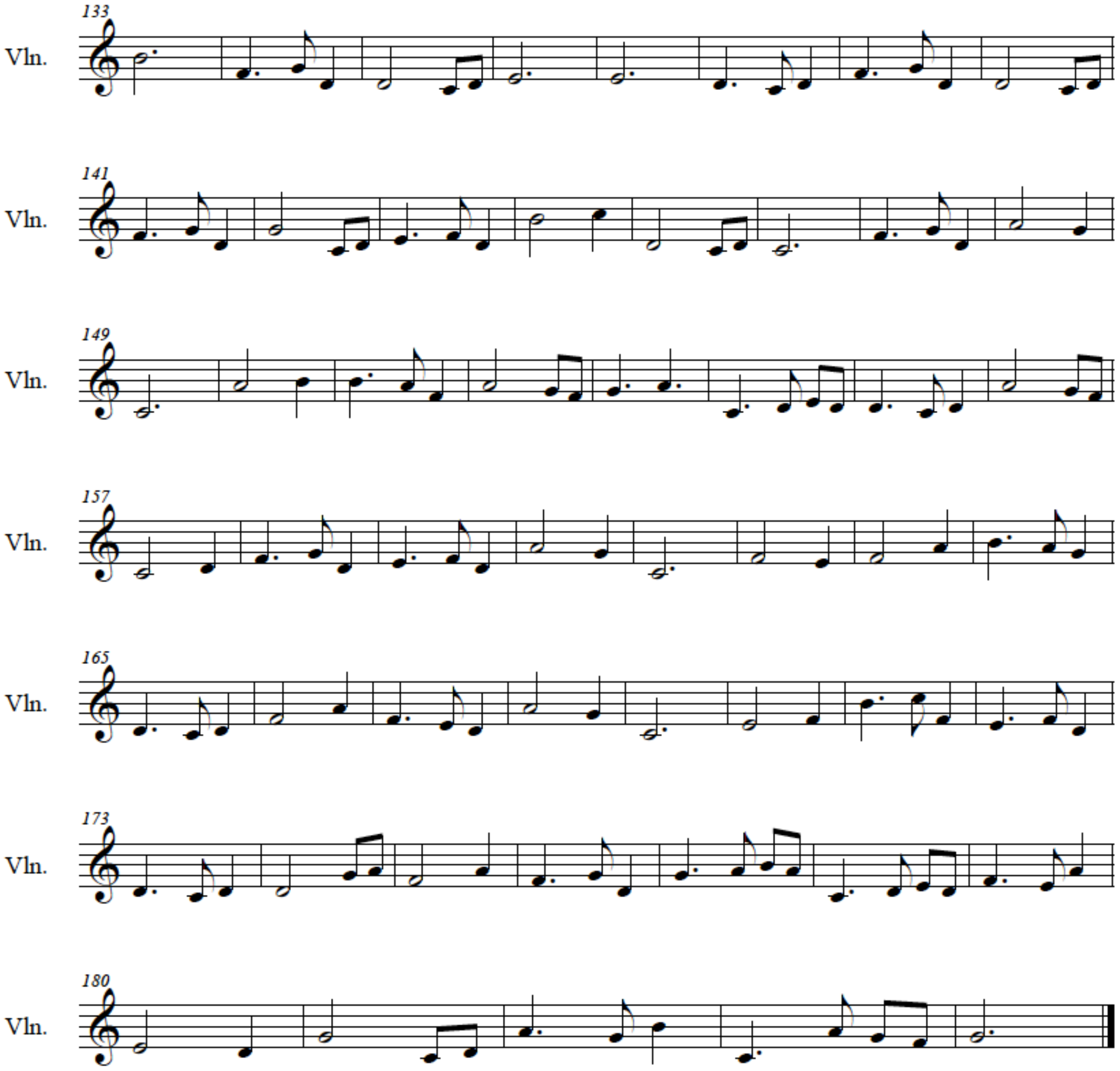
133
Vln.
141
Vln.
149
Vln.
157
Vln.
165
Vln.
173
Vln.
180
Vln.

>NM_001092015.1 *Xenopus laevis* Akirin2

```
ATG GCG TGT GGA GCC ACA CTT AAA AGG ACT ATC GAA TTC GAT CCT CTG TTG AGC CCA
 M   A   C   G   A   T   L   K   R   T   I   E   F   D   P   L   L   S   P
 M  DRR TTL SL DRMR FM  LF  RD  RSL FML TDS  FS  F  DTD MRL LSF LSF SDR  MR

GCA GCG TCT CCC AAG AGA AGA AGA TGC GCC CCC CTC TCT CCC TCG GGG  CCC TCC CCA
 A   A   S   P   K   R   R   R   C   A   P   L   S   P   S   G    P   S   P
DR  DRR SL MRMR RDR RFS RFS RFS  T DRMR MRMR LS  SL MRMR SR SLSL MRMR SMR MR

CAG  AAA TAC CTT CGC TTG GAA CCT TCA CCG TTC GGG GAG GTG TCC CCT CGT CTT ACT
 Q    K   Y   L   R   L   E   P   S   P   F   G   E   V   S   P   R   L   T
MFR  RD  LS  LF  RDR LSF FS  MRL  S  MRR  F SLSL FSR TLSF SMR MRL RTL LF FML

GCA GAG CAA ATC CTT TAT AAC ATT AAA CAA GAG TAT AAA CGA ATG CAA AAG CGA AGA
 A   E   Q   I   L   Y   N   I   K   Q   E   Y   K   R   M   Q   K   R   R
DR  FSR MF  TDS LF LSTD RM  TDF  RD  MF FSR LSTD RD  R   M  MF  RDR  R  RFS

CAT TTA GAA AGC AGC TTC CAA CCA ACA GAC CCC TGC TGC TCC AGC GAG GGC CAG CCA
 H   L   E   S   S   F   Q   P   T   D   P   C   C   S   S   E   G   Q   P
LTM LLS FS  SDR SDR  F  MF  MR  FM   D MRMR  T   T  SMR SDR FSR SLDR MFR MR

CAG ACT TTC ATC CCA TCT GGG CCG ACT TTA CCA GGC ACA TCA GCT ACA TCT CCA TTA
 Q   T   F   I   P   S   G   P   T   L   P   G   T   S   A   T   S   P   L
MFR FML  F  TDS MR  SL SLSL MRR FML LLS MR SLDR  FM  S  DRL  FM SL  MR  LLS

AGA AAG GAG CAG CCA TTG TTT TCA TTA AGG CAA GTA GGC ATG ATA TGT GAA CGA CTG
 R   K   E   Q   P   L   F   S   L   R   Q   V   G   M   I   C   E   R   L
RFS RDR FSR MFR MR  LSF FL   S  LLS RSL MF  TL SLDR  M  TD  TTL FS   R  LSF

CTT AAA GAA CGC GAG GAT AAT GTC CGT GAG GAA TAT GAA GAA ATA TTG ACC ACA AAA
 L   K   E   R   E   D   N   V   R   E   E   Y   E   E   I   L   T   T   K
LF  RD  FS  RDR FSR DTD RMTD TLS RTL FSR FS LSTD FS  FS  TD  LSF FMMR FM RD

CTC GCA GAA CAA TAT GAT GCT TTT GTG AAA TTC ACA CAT GAT CAG ATA ATG CGA CGA
 L   A   E   Q   Y   D   A   F   V   K   F   T   H   D   Q   I   M   R   R
LS  DR  FS  MF LSTD DTD DRL FL TLSF RD   F  FM  LTM DTD MFR TD   M   R   R

TTT GGA GAA CAG CCA GCT AGC TAC GTT TCA TGA
 F   G   E   Q   P   A   S   Y   V   S
FL   SL FS  MFR  MR DRL SDR LS  TLF  S  STOP
```

Xenopus laevis Akirin2

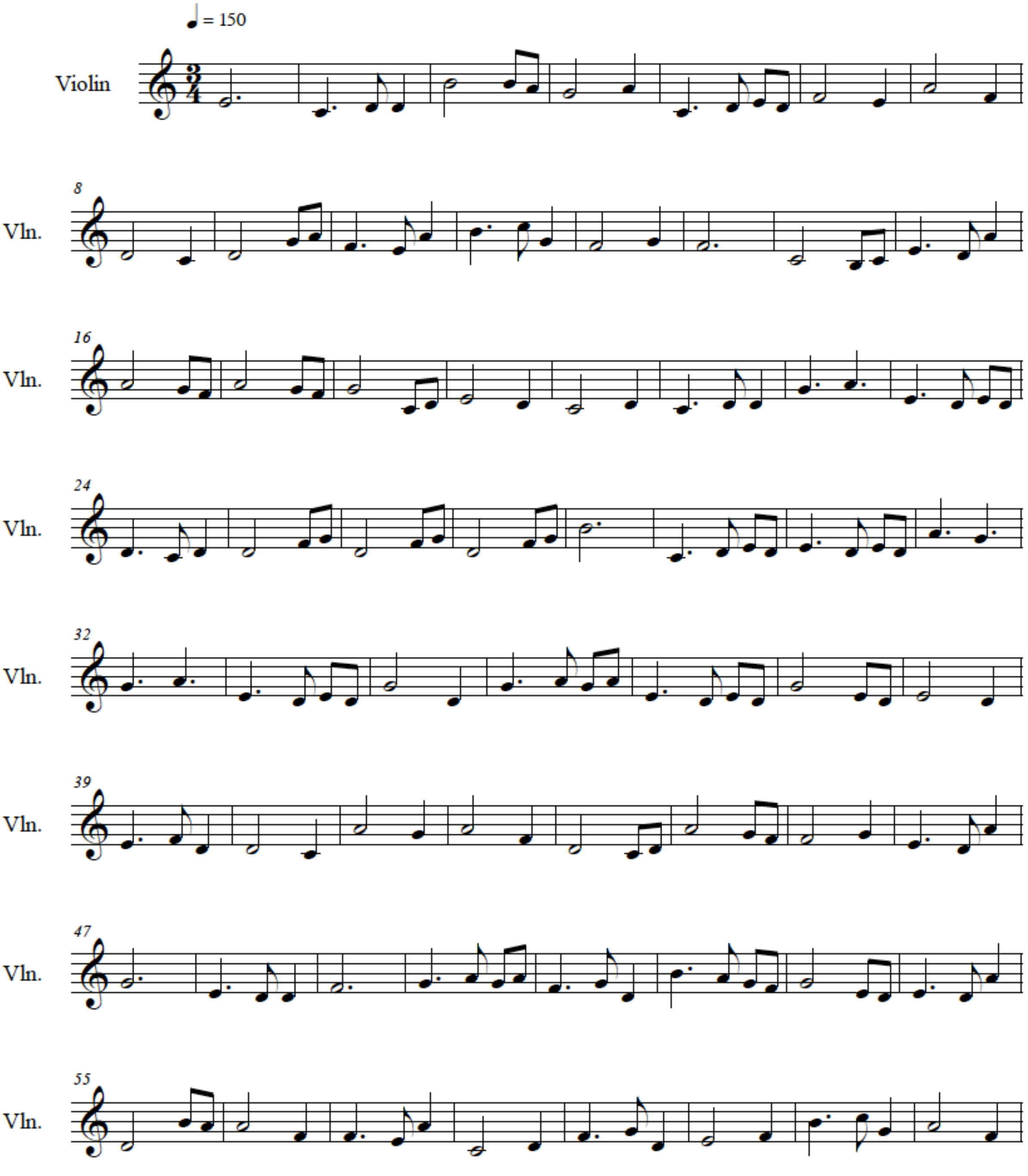

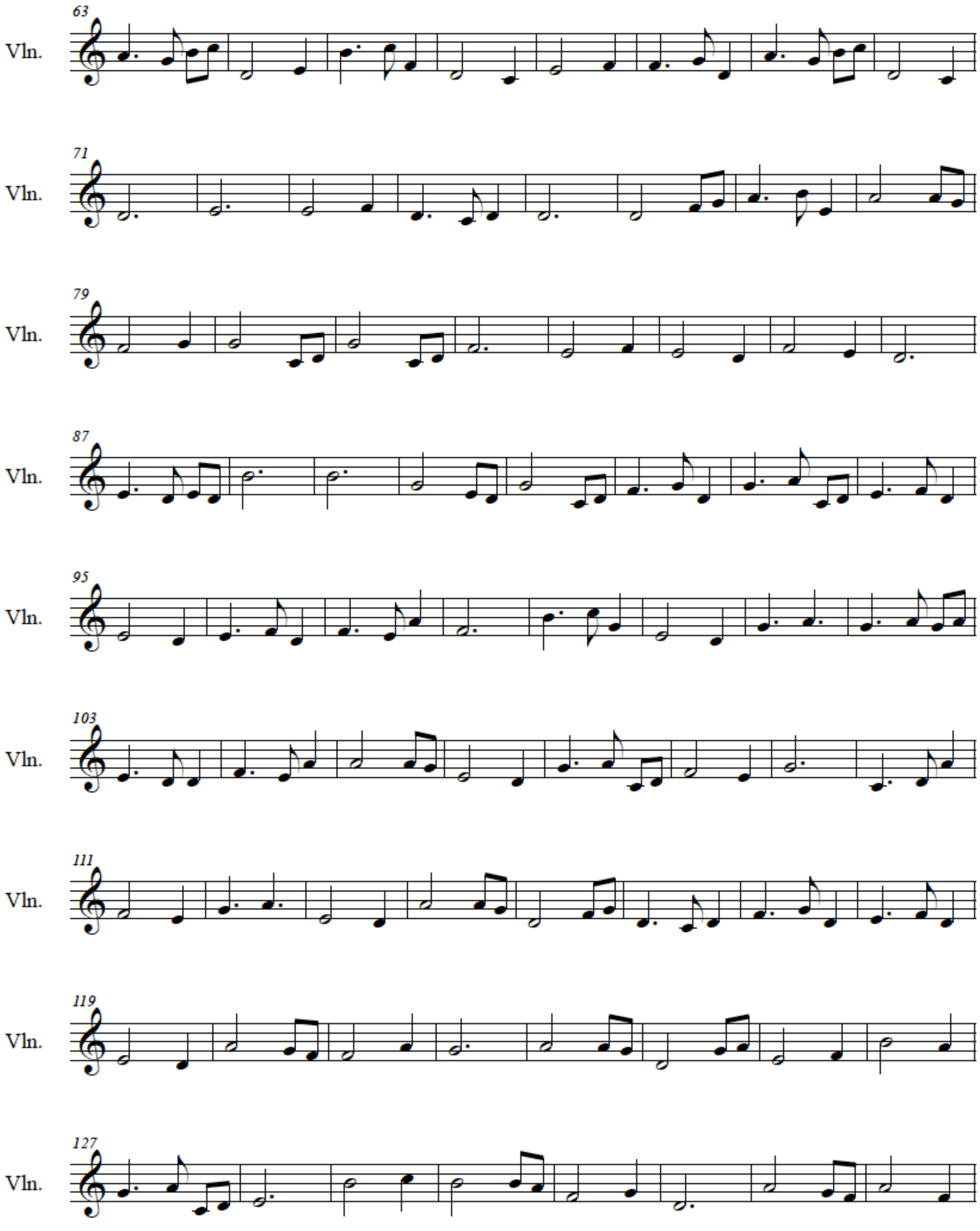
63
Vln.
71
Vln.
79
Vln.
87
Vln.
95
Vln.
103
Vln.
111
Vln.
119
Vln.
127
Vln.

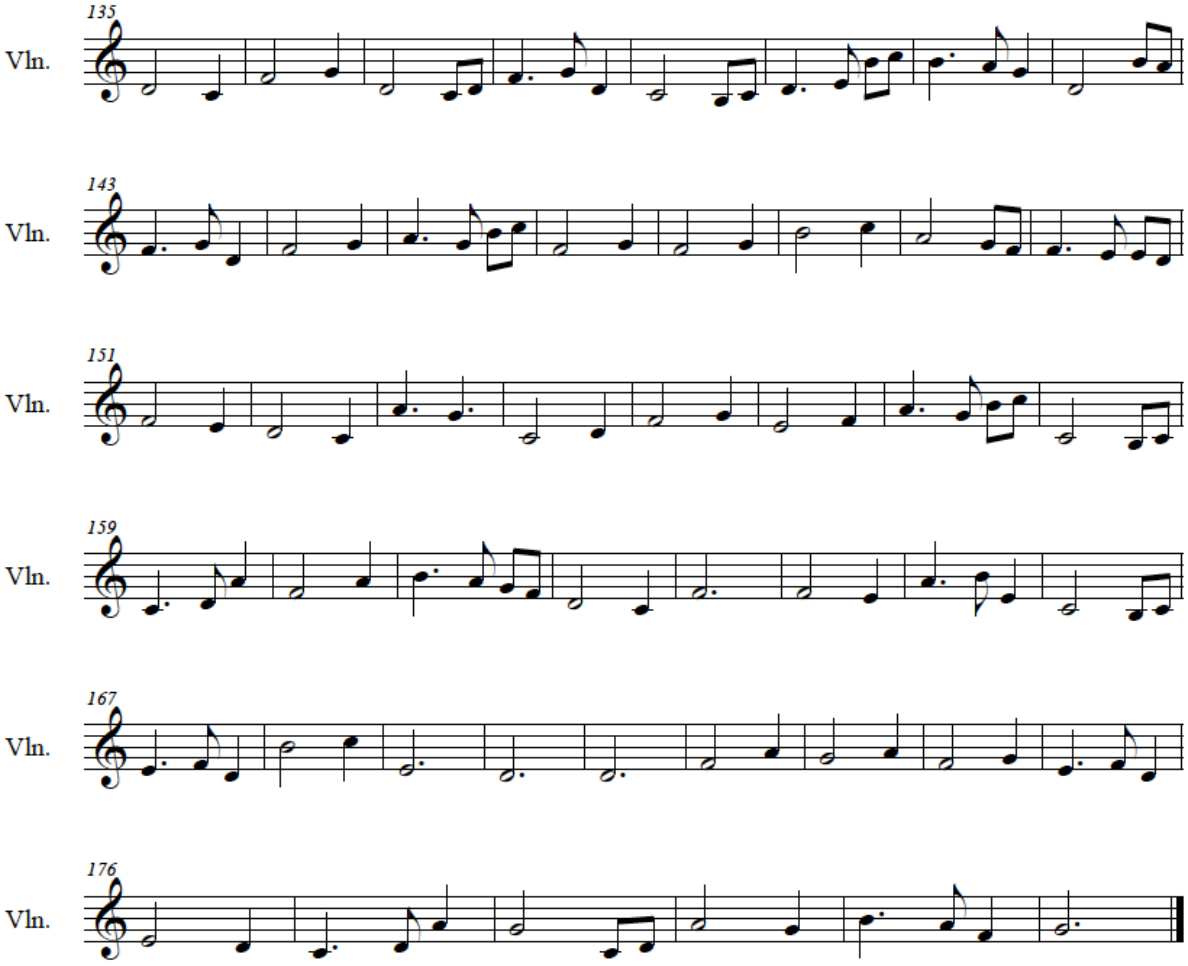
135
Vln.
143
Vln.
151
Vln.
159
Vln.
167
Vln.
176
Vln.

>GQ247760.1 *Salvelinus alpinus* Akirin2

```
ATG GCT TGT GGG GCT ACT TTG AAA AGG ACT ATG GAT TTC GAT CCA TTG ATG AAC CAG
 M   A   C   G   A   T   L   K   R   T   M   D   F   D   P   L   M   N   Q
 M DRL TTL SLSL DRL FML LSF RD  RSL FML  M  DTD  F  DTD MR  LSF  M  RM  MFR

GCG TCC CCC AAA AGG AGG AGG TGC ACC CCA ATG TCT CAA GCC GCC TCG ACC TCA TCA
 A   S   P   K   R   R   R   C   T   P   M   S   Q   A   A   S   T   S   S
DRR SMR MRMR RD RSL RSL RSL  T FMMR MR   M  SL  MF DRMR DRMR SR FMMR S   S

CCC CAG AAA TAT  CTG CGT ATG GAG CCC TCA CCA TTT GGA GAA GTG TCG TCC AGA CTC
 P   Q   K   Y    L   R   M   E   P   S   P  F   G   E   V   S   S   R   L
MRMR MFR RD LSTD LSF RTL  M  FSR MRMR S  MR  FL  SL  FS TLSF SR  SMR RFS  LS

ACA ACG GAG CAA ATC CTG CAC AAT ATC AAG CAA GAG TAC AAG CGG ATG CAG AAG CGA
 T   T   E   Q   I   L   H   N   I   K   Q   E   Y   K   R   M   Q   K   R
FM  FMR FSR MF  TDS LSF LT RMTD TDS RDR MF  FSR LS  RDR RSL  M  MFR RDR  R

AGG CAC CTG GAG AAC AGC TTT CAG CAG ACC GAG GGC TGT  TGT CCC  CTG GAG TCA CAA
 R   H   L   E   N   S   F   Q   Q   T   E   G   C    C   P    L   E   S   Q
RSL LT LSF FSR RM  SDR FL  MFR MFR FMMR FSR SLDR TTL TTL MRMR LSF FSR  S   MF

CCC  CAT AGC TCC ATC CTT AAT GGA TCC AGT CTG CCA GGA ACA TCT TCT GGT  GCC
ATC
 P    H   S   S   I   L   N   G   S   S   L   P   G   T   S   S   G    A    I
MRMR LTM SDR SMR TDS LF RMTD SL SMR STL LSF  MR  SL  FM  SL  SL SLTL DRMR TDS

TCC CCA TCT AGA AAA GAG CAA CCT CTA TTT ACC TTG AGA CAG GTT GGG ATG ATC TGT
 S   P   S   R   K   E   Q   P   L   F   T   L   R   Q   V   G   M   I   C
SMR MR  SL  RFS RD  FSR MF  MRL  L  FL FMMR LSF RFS MFR TLF SLSL M  TDS TTL

GAA CGA CTA CTT AAA GAA CGA GAG GAG AAG ATA AGG GAA GAA TAT GAC GAA ATA TTG
 E   R   L   L   K   E   R   E   E   K   I   R   E   E   Y   D   E   I   L
FS   R   L  LF  RD  FS   R  FSR FSR RDR TD  RSL FS  FS LSTD  D  FS  TD  LSF

ACA ACA AAA CTA GCA GAG CAA TAT GAT GCT TTC GTT AAG TTC ACT CAC GAT CAG CTA
 T   T   K   L   A   E   Q   Y   D   A   F   V   K   F   T   H   D   Q   L
FM  FM  RD   L  DR  FSR MF LSTD DTD DRL  F  TLF RDR  F  FML LT  DTD MFR  L

ATG CGG CGA TTT GGA GAG CAA CCT GCT AGC TAT  GTT  TCC TGA
 M   R   R   F   G   E   Q   P   A   S   Y    V    S
 M  RSL  R  FL  SL  FSR MF  MRL DRL SDR LSTD TLF  SMR STOP
```

***Salvelinus alpinus* Akirin2**

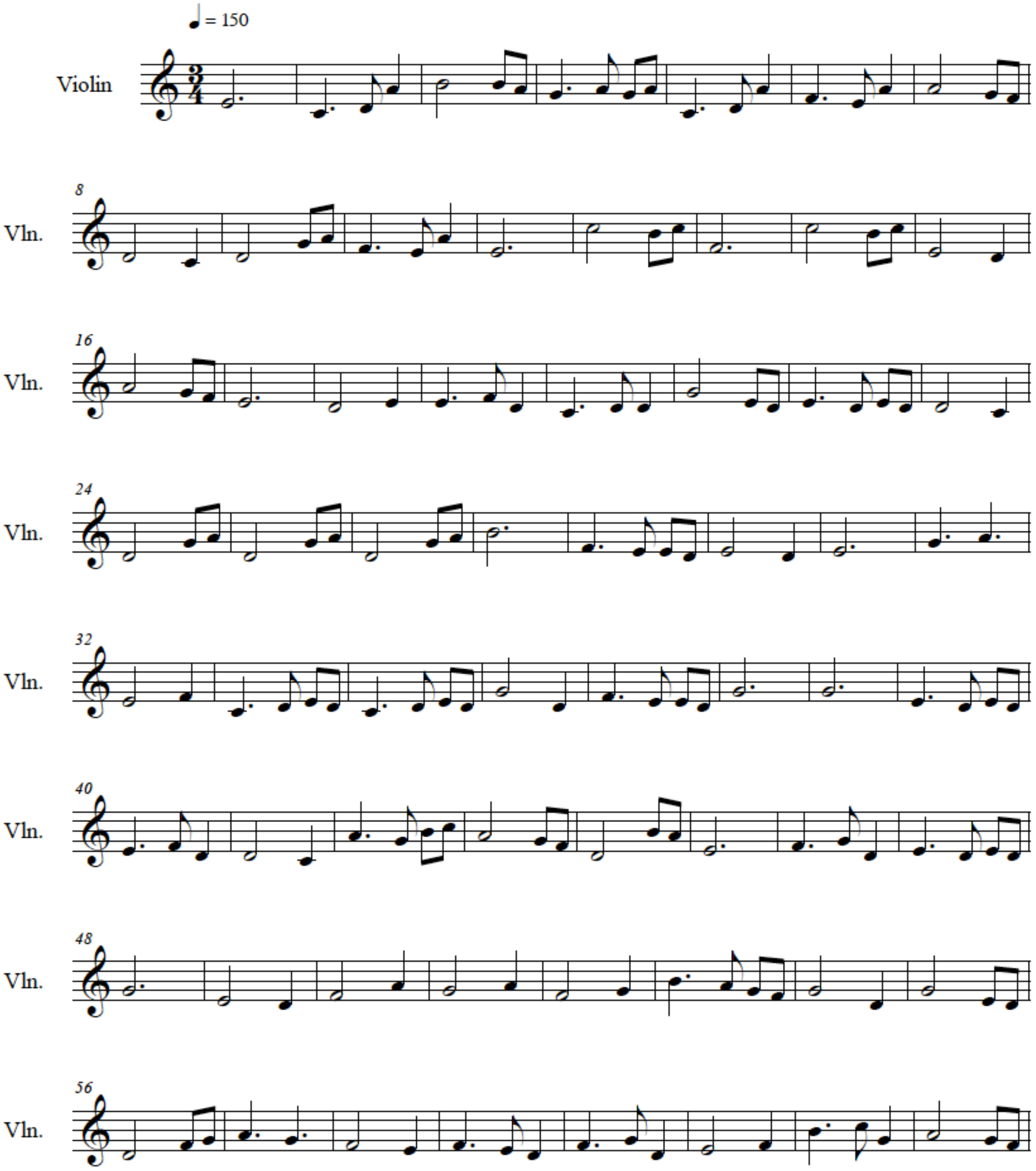

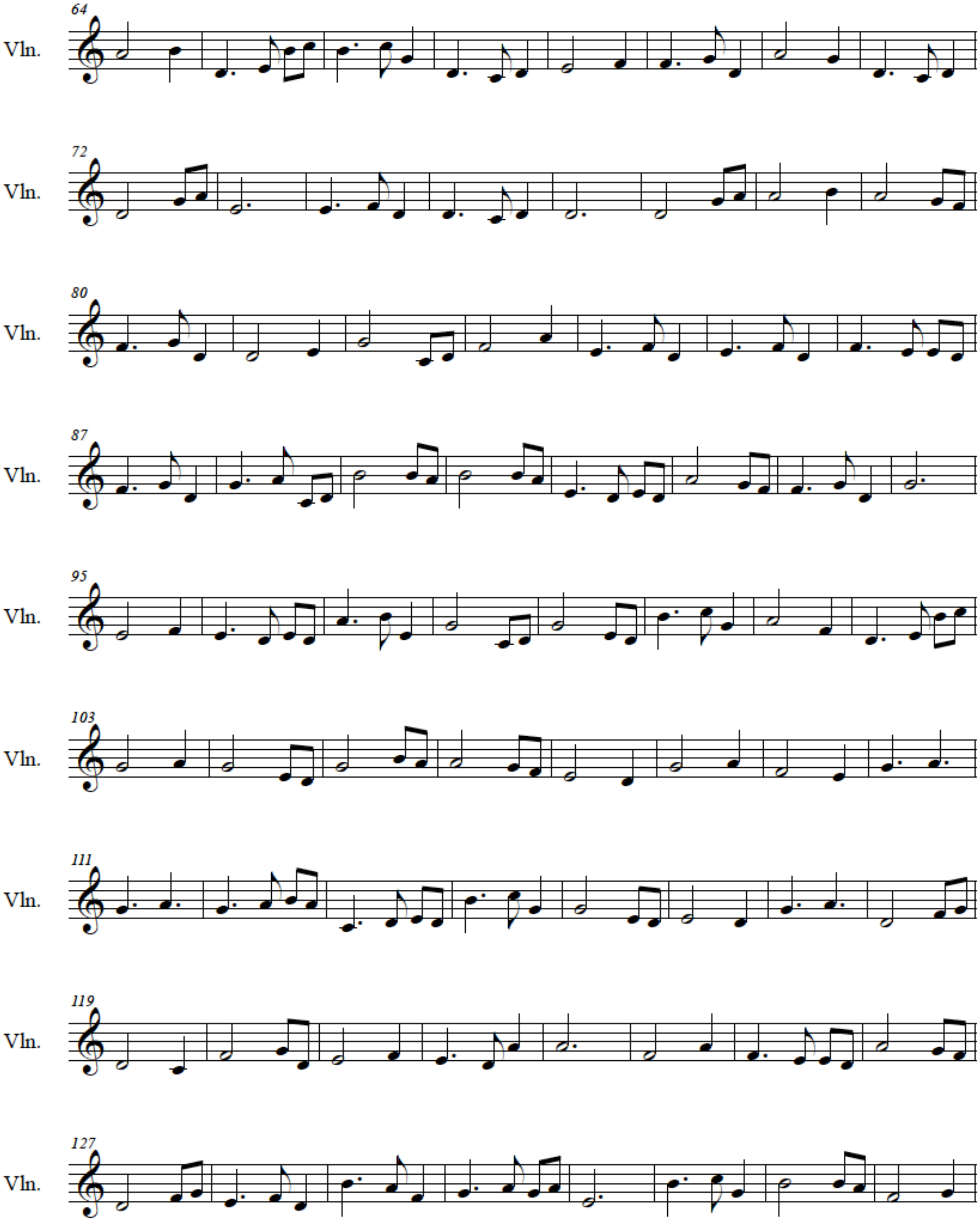
64
Vln.
72
Vln.
80
Vln.
87
Vln.
95
Vln.
103
Vln.
111
Vln.
119
Vln.
127
Vln.

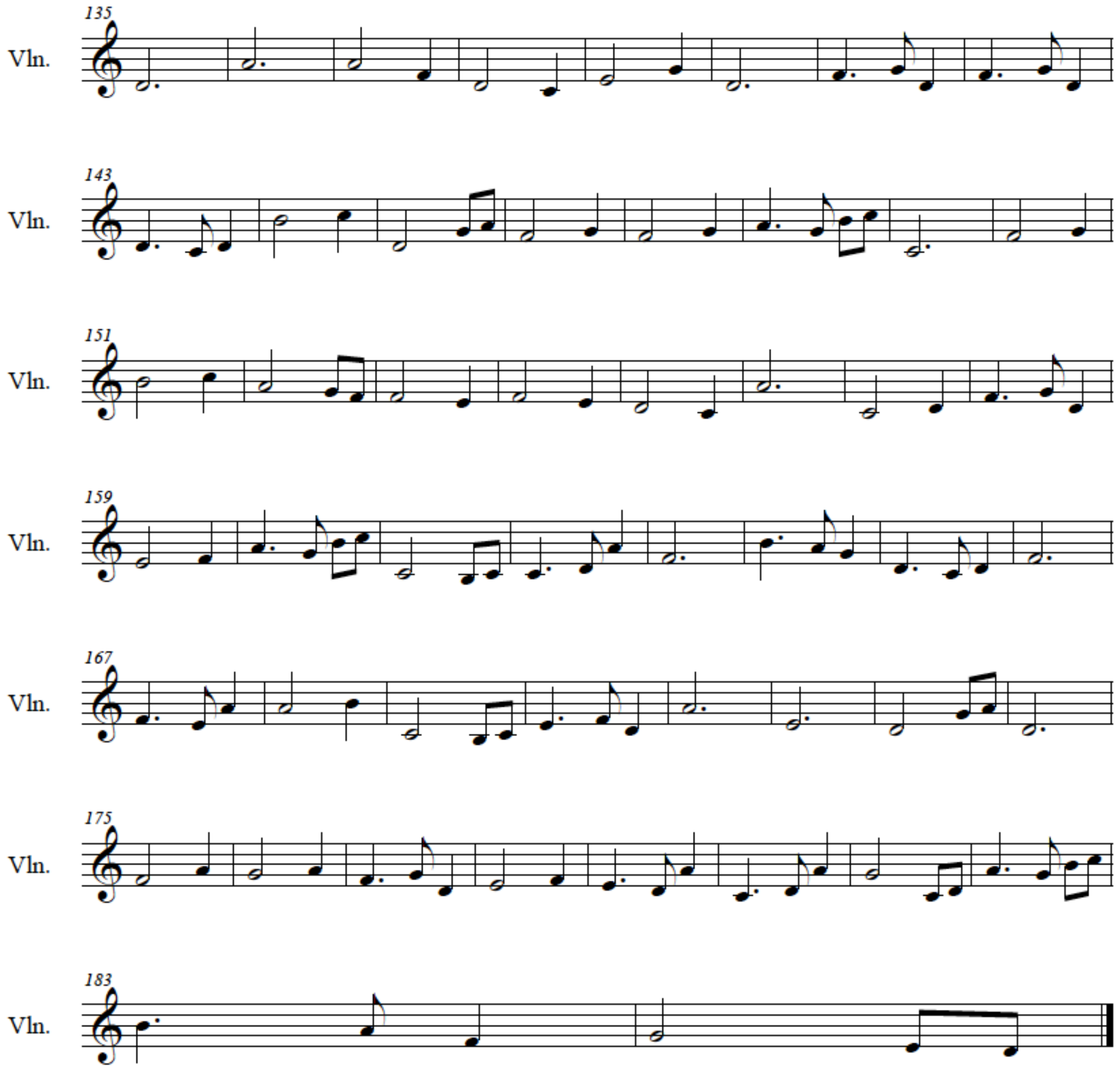
135
Vln.
143
Vln.
151
Vln.
159
Vln.
167
Vln.
175
Vln.
183
Vln.

>NM_018064.3 *Homo sapiens* Akirin2

```
ATG GCG TGC GGA GCC ACT CTG AAA AGG ACT CTG GAT TTC GAC CCG CTG TTG AGC CCG
 M   A   C   G   A   T   L   K   R   T   L   D   F   D   P   L   L   S   P
 M  DRR  T  SL DRMR FML LSF RD  RSL FML LSF DTD  F   D  MRR LSF LSF SDR MRR

GCG TCC CCG AAG CGC AGG CGA TGT GCG CCA TTG TCG GCG CCC ACC  TCG  GCC GCT GCC
 A   S   P   K   R   R   R   C   A   P   L   S   A   P   T    S    A   A   A
DRR SMR MRR RDR RDR RSL  R  TTL DRR  MR LSF SR  DRR MRMR FMMR SR DRMR DRL
DRMR

TCC CCG TTG TCG GCG GCC GCG GCC  ACC  GCC  GCC TCC TTC TCC GCT GCG GCC  GCC TCG
 S   P   L   S  A    A   A   A    T    A    A   S   F   S   A   A   A    A   S
SMR MRR LSF SR DRR DRMR DRR DRMR FMMR DRMR DRMR SMR F  SMR DRL DRR DRMR DRMR SR

CCG CAG AAG TAT CTC CGA ATG GAG CCA TCC CCC TTC GGC GAC GTC TCC TCC CGC CTC
 P   Q   K   Y   L   R   M   E   P   S   P   F   G   D   V   S   S   R   L
MRR MFR RDR LSTD LS  R   M  FSR MR  SMR MRMR F SLDR  D  TLS SMR SMR RDR  LS

ACC ACA GAA CAA ATT CTG TAC AAC ATA AAA CAA GAG TAT AAA CGA ATG CAG AAG AGA
 T   T   E   Q   I   L   Y   N   I   K   Q   E   Y   K   R   M   Q   K   R
FMMR FM FS  MF  TDF LSF LS  RM  TD  RD  MF  FSR LSTD RD  R   M  MFR RDR RFS

AGA CAT TTA GAA ACG AGT TTC CAA CAG ACA GAT CCG TGT TGT ACT TCT GAT GCA CAG
 R   H   L   E   T   S   F   Q   Q   T   D   P   C   C   T   S   D   A   Q
RFS LTM LLS FS  FMR STL  F  MF  MFR FM  DTD MRR TTL TTL FML SL  DTD DR  MFR

CCA CAT GCA TTT CTC CTC AGT GGA CCA GCT TCA CCA GGG ACT TCA TCT GCA GCA TCC
 P   H   A   F   L   L   S   G   P   A   S   P   G   T   S   S   A   A   S
MR  LTM DR  FL  LS  LS  STL SL  MR  DRL  S  MR SLDR FML  S  SL  DR  DR  SMR

TCA CCA TTA AAA AAA GAA CAG CCC TTA TTT ACT CTA CGG CAG GTT GGG ATG ATC TGT
 S   P   L   K   K   E   Q   P   L   F   T   L   R   Q   V   G   M   I   C
 S MR  LLS RD  RD  FS  MFR MRMR LLS FL  FML  L  RDR MFR TLF SLSL M  TDS TTL

GAA CGT TTG TTG AAA GAA CGT GAA GAG AAA GTT CGA GAA GAA TAT GAA GAA ATA TTG
 E   R   L   L   K   E   R   E   E   K   V   R   E   E   Y   E   E   I   L
FS  RTL LSF LSF RD  FS  RTL FS  FSR RD  TLF  R  FS  FS LSTD FS  FS  TD  LSF

AAC ACA AAA CTT GCA GAA CAA TAT GAT GCG TTT GTG AAG TTT ACG CAT GAT CAA ATA
 N   T   K   L   A   E   Q   Y   D   A   F   V   K   F   T   H   D   Q   I
RM  FM  RD  LF  DR  FS  MF LSTD DTD DRR FL TLSF RDR FL  FMR LTM DTD  MF  TD
```

```
ATG CGA CGA TAT GGA  GAA  CAG CCT GCT AGC TAT GTT TCA TGA
 M   R   R   Y   G    E    Q   P   A   S   Y   V   S
 M   R   R LSTD  SL    FS  MFR MRL DRL SDR LSTD TLF S  STOP
```

Homo sapiens **Akirin2**

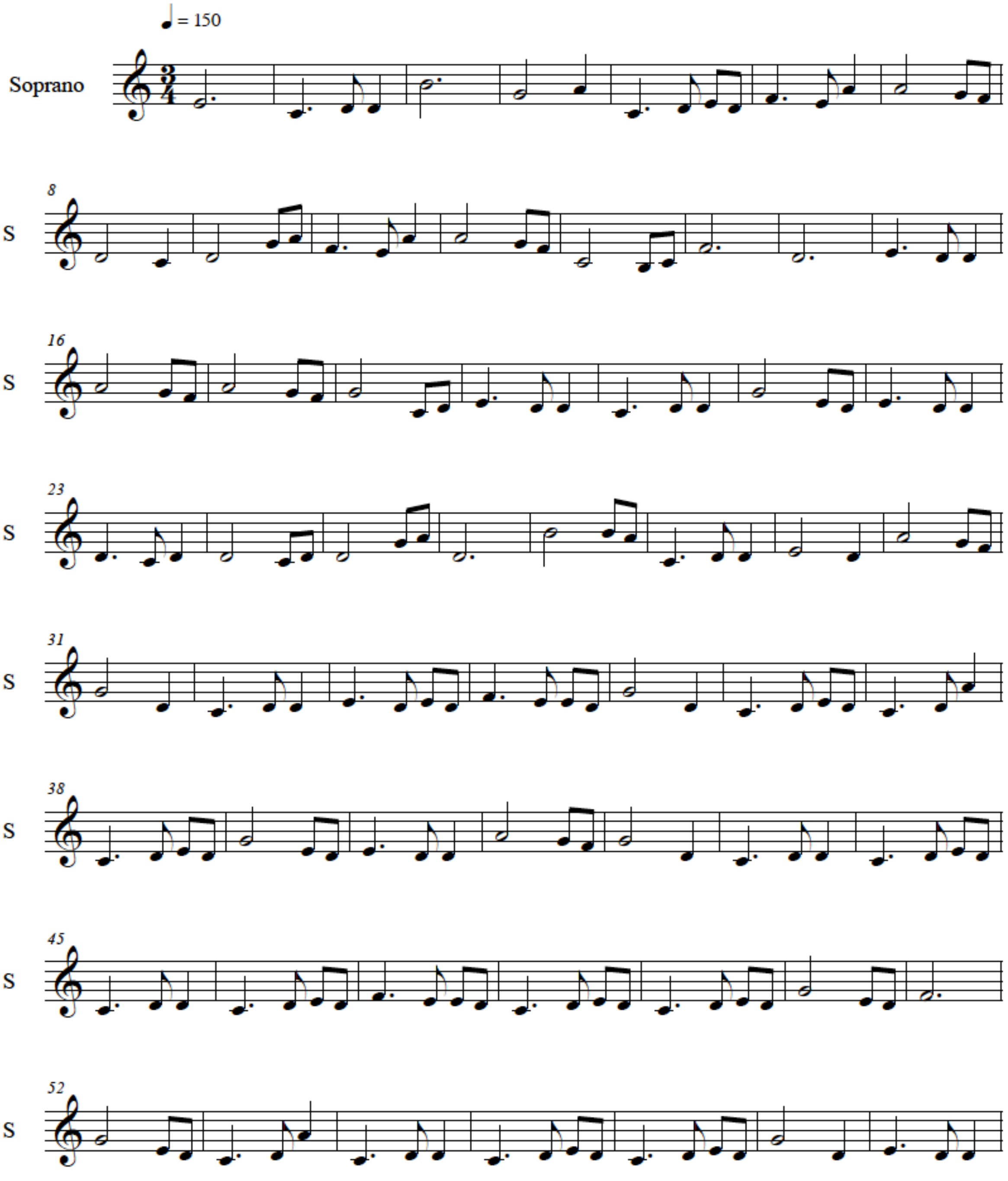

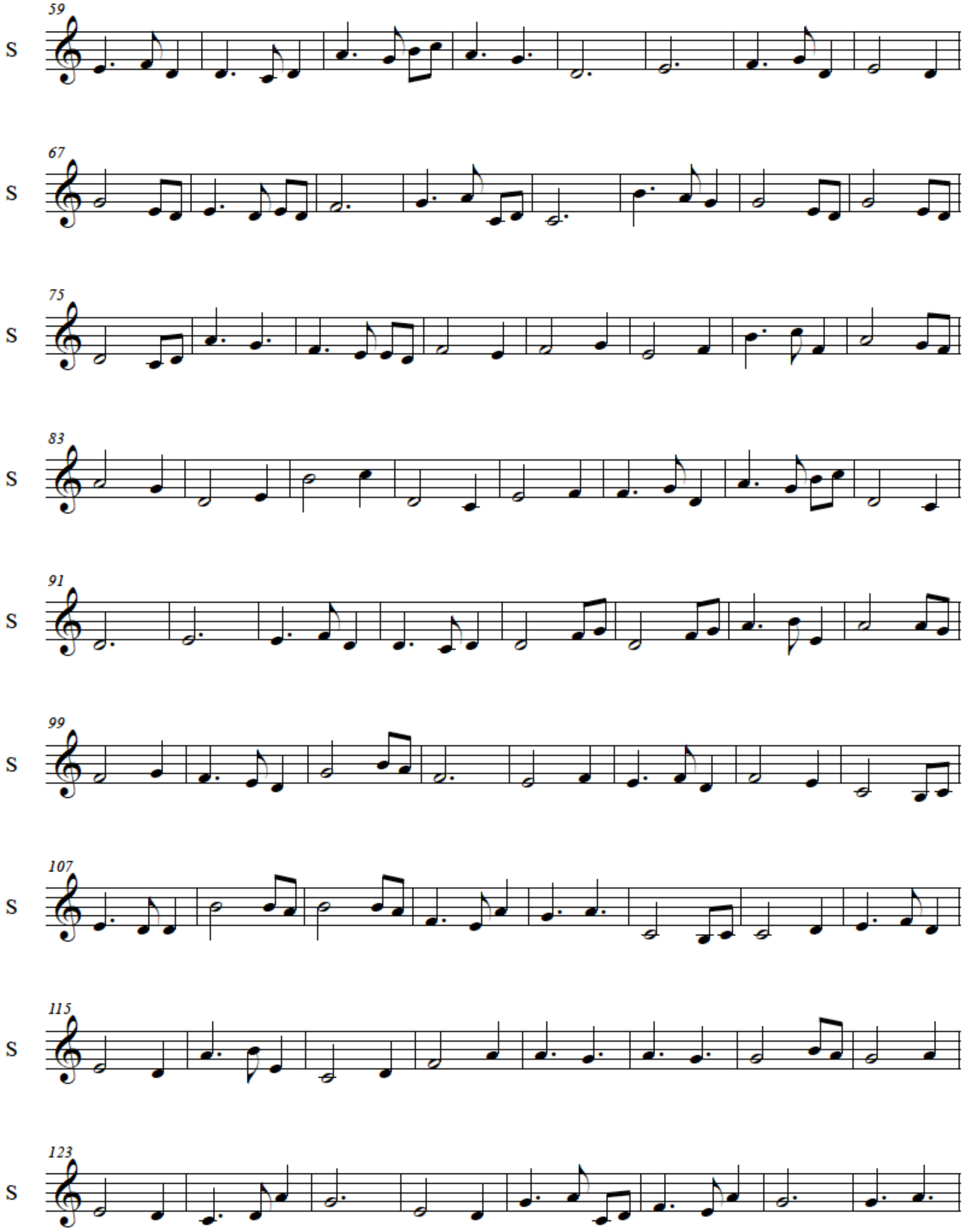
59
S
67
S
75
S
83
S
91
S
99
S
107
S
115
S
123
S

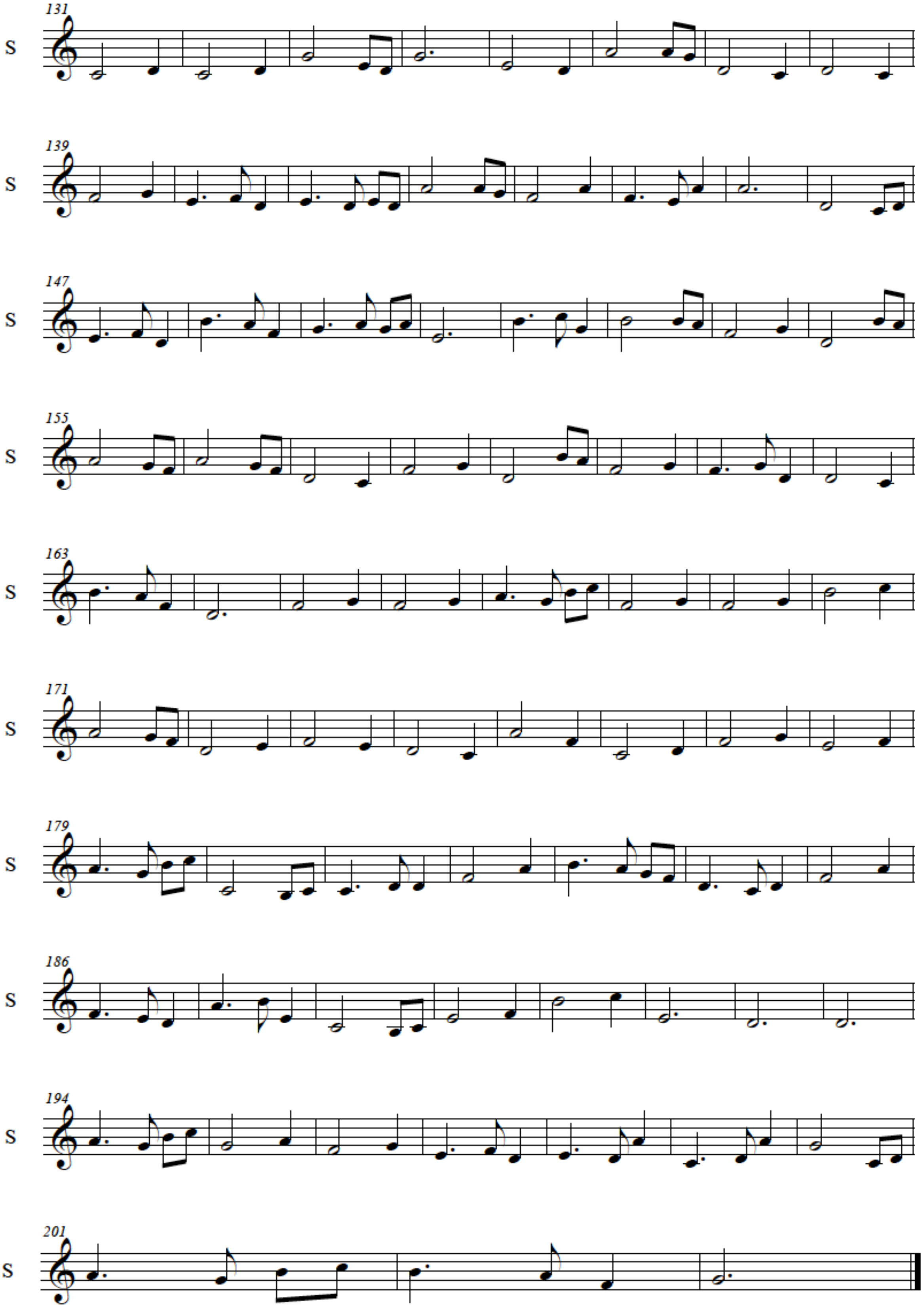
131
S
139
S
147
S
155
S
163
S
171
S
179
S
186
S
194
S
201
S

>NM_014868.4 *Homo sapiens* ring finger protein 10 (RNF10)

ATG CCG CTG AGC TCC CCC AAC GCC GCC GCC ACC GCC TCC GAC ATG GAC AAG AAC AGC
M P L S S P N A A A T A S D M D K N S
M MRR LSF SDR SMR MRMR RM DRMR DRMR DRMR FMMR DRMR SMR D M D RDR RM SDR

GGC TCC AAC AGC TCC TCC GCC TCT TCG GGC AGC AGC AAA GGG CAA CAG CCG CCC CGC
G S N S S S A S S G S S K G Q Q P P R
SLDR SMR RM SDR SMR SMR DRMR SL SR SLDR SDR SDR RD SLSL MF MFR MRR MRMR RDR

TCC GCC TCG GCG GGG CCA GCC GGC GAG TCT AAA CCC AAG AGC GAT GGA AAG AAC TCC
S A S A G P A G E S K P K S D G K N S
SMR DRMR SR DRR SLSL MR DRMR SLDR FSR SL RD MRMR RDR SDR DTD SL RDR RM SMR

AGT GGA TCC AAG CGT TAT AAT CGC AAA CGT GAA CTT TCC TAC CCC AAA AAT GAA AGT
S G S K R Y N R K R E L S Y P K N E S
STL SL SMR RDR RTL LSTD RMTD RDR RD RTL FS LF SMR LS MRMR RD RMTD FS STL

TTT AAC AAC CAG TCC CGT CGC TCC AGT TCA CAG AAA AGC AAG ACT TTT AAC AAG ATG
F N N Q S R R S S S Q K S K T F N K M
FL RM RM MFR SMR RTL RDR SMR STL S MFR RD SDR RDR FML FL RM RDR M

CCT CCT CAA AGG GGC GGC GGC AGC AGC AAA CTC TTT AGC TCT TCT TTT AAT GGT GGA
P P Q R G G G S S K L F S S S F N G G
MRL MRL MF RSL SLDR SLDR SLDR SDR SDR RD LS FL SDR SL SL FL RMTD SLTL SL

AGA CGA GAT GAG GTA GCA GAG GCT CAA CGG GCA GAG TTT AGC CCT GCC CAG TTC TCT
R R D E V A E A Q R A E F S P A Q F S
RFS R DTD FSR TL DR FSR DRL MF RSL DR FSR FL SDR MRL DRMR MFR F SL

GGT CCT AAG AAG ATC AAC CTG AAC CAC TTG TTG AAT TTC ACT TTT GAA CCC CGT GGC
G P K K I N L N H L L N F T F E P R G
SLTL MRL RDR RDR TDS RM LSF RM LT LSF LSF RMTD F FML FL FS MRMR RTL SLDR

CAG ACG GGT CAC TTT GAA GGC AGT GGA CAT GGT AGC TGG GGA AAG AGG AAC AAG TGG
Q T G H F E G S G H G S W G K R N K W
MFR FMR SLTL LT FL FS SLDR STL SL LTM SLTL SDR SF SL RDR RSL RM RDR SF

GGA CAT AAG CCT TTT AAC AAG GAA CTC TTT TTA CAG GCC AAC TGC CAA TTT GTG GTG
G H K P F N K E L F L Q A N C Q F V V
SL LTM RDR MRL FL RM RDR FS LS FL LLS MFR DRMR RM T MF FL TLSF TLSF

```
TCT GAA GAC CAA GAC TAC ACA GCT CAT TTT GCT GAT CCT GAT ACA TTA GTT AAC TGG
 S   E   D   Q   D   Y   T   A   H   F   A   D   P   D   T   L   V   N   W
SL  FS   D   MF  D  LS  FM  DRL LTM  FL DRL DTD MRL DTD FM  LLS TLF  RM  SF

GAC TTT GTG GAA CAA GTG  CGC ATT TGT AGC CAT GAA GTG CCA TCT TGC CCA ATA TGC
 D   F   V   E   Q   V    R   I   C   S   H   E   V   P   S   C   P   I   C
 D   FL TLSF FS  MF TLSF RDR TDF TTL SDR LTM FS TLSF MR  SL   T  MR  TD   T

CTC TAT CCA  CCT ACT GCA GCC AAG ATA ACC  CGT TGT GGA CAC ATC TTC TGC TGG GCA
 L   Y   P    P   T   A   A   K   I   N    R   C   G   H   I   F   C   W   A
LS LSTD  MR  MRL FML DR DRMR RDR  TD  RM  RTL TTL SL  LT  TDS  F   T  SF  DR

TGC ATC CTG CAC TAT CTT TCA CTG AGT GAG AAG ACG TGG AGT AAA TGT CCC ATC TGT
 C   I   L   H   Y   L   S   L   S   E   K   T   W   S   K   C   P   I   C
 T  TDS LSF LT LSTD  LF  S  LSF STL FSR RDR FMR SF  STL RD  TTL MRMR TDS TTL

TAC AGT TCT GTG CAT AAG AAG GAT CTC AAG AGT GTT GTT GCC ACA GAG TCA CAT CAG
 Y   S   S   V   H   K   K   D   L   K   S   V   V   A   T   E   S   H   Q
LS  STL SL TLSF LTM RDR RDR DTD LS  RDR STL TLF TLF DRMR FM FSR  S  LTM MFR

TAT  GTT GTT GGT GAT ACC  ATT ACG ATG CAG CTG ATG AAG AGG GAG AAA GGG GTG  TTG
 Y    V   V   V   D   T    I   T   M   Q   L   M   K   R   E   K   G   V    L
LSTD TLF TLF TLF DTD FMMR TDF FMR  M  MFR LSF  M  RDR RSL FSR RD SLSL TLSF LSF

GTG  GCT TTG CCC AAA TCC AAA TGG ATG AAT GTA GAC CAT CCC  ATT CAT CTA GGA GAT
 V    A   L   P   K   S   K   W   M   N   V   D   H   P    I   H   L   G   D
TLSF DRL LSF MRMR RD SMR RD   SF  M RMTD TL   D  LTM MRMR TDF LTM  L  SL  DTD

GAA CAG CAC AGC CAG TAC TCC AAG TTG CTG CTG GCC TCT AAG GAG CAG GTG  CTG CAC
 E   Q   H   S   Q   Y   S   K   L   L   L   A   S   K   E   Q   V    L   H
FS  MFR LT  SDR MFR  LS SMR RDR LSF LSF LSF DRMR SL RDR FSR MFR TLSF LSF LT

CGG GTA GTT CTG GAG GAG AAA GTA GCA CTA GAG CAG CAG CTG GCA GAG GAG AAG CAC
 R   V   V   L   E   E   K   V   A   L   E   Q   Q   L   A   E   E   K   H
RSL TL  TLF LSF FSR FSR RD  TL  DR   L  FSR MFR MFR LSF DR  FSR FSR RDR  LT

ACT CCC  GAG TCC TGC TTT ATT GAG GCA GCT ATC CAG GAG CTC AAG ACT CGG GAA GAG
 T   P    E   S   C   F   I   E   A   A   I   Q   E   L   K   T   R   E   E
FML MRMR FSR SMR  T  FL  TDF FSR DR  DRL TDS MFR FSR LS  RDR FML RSL FS  FSR
```

GCT CTG TCG GGA TTG GCC GGA AGC AGA AGG GAG GTC ACT GGT GTT GTG GCT GCT CTG
 A   L   S   G   L   A   G   S   R   R   E   V   T   G   V   V   A   A   L
DRL LSF SR SL LSF DRMR SL SDR RFS RSL FSR TLS FML SLTL TLF TLSF DRL DRL LSF

GAA CAA CTG GTG CTG ATG GCT CCC TTG GCG AAG GAG TCT GTT TTT CAA CCC AGG AAG
 E   Q   L   V   L   M   A   P   L   A   K   E   S   V   F   Q   P   R   K
FS   MF LSF TLSF LSF M DRL MRMR LSF DRR RDR FSR SL TLF FL MF MRMR RSL RDR

GGT GTG CTG GAG TAT CTG TCT GCC TTC GAT GAA GAA ACC ACG GAA GTT TGT TCT CTG
 G   V   L   E   Y   L   S   A   F   D   E   E   T   T   E   V   C   S   L
SLTL TLSF LSF FSR LSTD LSF SL DRMR F DTD FS FS FMMR FMR FS TLF TTL SL LSF

GAC ACT CCT TCT AGA CCT CTT GCT CTC CCT CTG GTA GAA GAG GAG GAA GCA GTG TCT
 D   T   P   S   R   P   L   A   L   P   L   V   E   E   E   E   A   V   S
 D FML MRL SL RFS MRL LF DRL LS MRL LSF TL FS FSR FSR FS DR TLSF SL

GAA CCA GAG CCT GAG GGG TTG CCA GAG GCC TGT GAT GAC TTG GAG TTA GCA GAT GAC
 E   P   E   P   E   G   L   P   E   A   C   D   D   L   E   L   A   D   D
FS   MR FSR MRL FSR SLSL LSF MR FSR DRMR TTL DTD D LSF FSR LLS DR DTD D

AAT CTT AAA GAG GGG ACC ATT TGC ACT GAG TCC AGC CAG CAG GAA CCC ATC ACC AAG
 N   L   K   E   G   T   I   C   T   E   S   S   Q   Q   E   P   I   T   K
RMTD LF RD FSR SLSL FMMR TDF T FML FSR SMR SDR MFR MFR FS MRMR TDS FMMR RDR

TCA GGC TTC ACA CGC CTC AGC AGC TCT CCT TGT TAC TAC TTT TAC CAA GCG GAA GAT
 S   G   F   T   R   L   S   S   S   P   C   Y   Y   F   Y   Q   A   E   D
 S SLDR F FM RDR LS SDR SDR SL MRL TTL LS LS FL LS MF DRR FS DTD

GGA CAG CAT ATG TTC CTG CAC CCT GTG AAT GTG CGC TGC CTC GTG CGG GAG TAC GGC
 G   Q   H   M   F   L   H   P   V   N   V   R   C   L   V   R   E   Y   G
SL MFR LTM M   F LSF LT MRL TLSF RMTD TLSF RDR T LS TLSF RSL FSR LS SLDR

AGC CTG GAG AGG AGC CCC GAG AAG ATC TCA GCA ACT GTG GTG GAG ATT GCT GGC TAC
 S   L   E   R   S   P   E   K   I   S   A   T   V   V   E   I   A   G   Y
SDR LSF FSR RSL SDR MRMR FSR RDR TDS S DR FML TLSF TLSF FSR TDF DRL SLDR LS

TCC ATG TCT GAG GAT GTT CGA CAG CGT CAC AGA TAT CTC TCT CAC TTG CCA CTC ACC
 S   M   S   E   D   V   R   Q   R   H   R   Y   L   S   H   L   P   L   T
SMR M SL FSR DTD TLF R MFR RTL LT RFS LSTD LS SL LT LSF MR LS FMMR

```
TGT GAG TTC AGC ATC TGT GAA CTG GCT TTG CAA CCT CCT GTG  GTC TCT AAG GAA ACC
 C   E   F   S   I   C   E   L   A   L   Q   P   P   V    V   S   K   E   T
TTL FSR  F  SDR TDS TTL FS  LSF DRL LSF MF  MRL MRL TLSF TLS SL  RDR  FS FMMR

CTA GAG ATG TTC TCA GAT GAC ATT GAG AAG AGG AAA CGT CAG CGC CAA AAG AAG GCT
 L   E   M   F   S   D   D   I   E   K   R   K   R   Q   R   Q   K   K   A
 L  FSR  M   F   S  DTD  D  TDF FSR RDR RSL RD  RTL MFR RDR MF  RDR RDR DRL

CGG GAG GAA CGC CGC CGA GAG CGC AGG ATT GAG ATA GAG GAG AAC AAG AAA CAG GGC
 R   E   E   R   R   R   E   R   R   I   E   I   E   E   N   K   K   Q   G
RSL FSR FS  RDR RDR  R  DSR RDR RSL TDF FSR TD  FSR FSR RM  RDR RD  MFR SLDR

AAG TAC CCA GAA GTC CAC ATT CCC CTC GAG AAT CTA CAG CAG TTT CCT GCC TTC AAT
 K   Y   P   E   V   H   I   P   L   E   N   L   Q   Q   F   P   A   F   N
RDR LS  MR  FS  TLS  LT TDF MRMR LS FSR RMTD L  MFR MFR  FL MRL DRMR F RMTD

TCT TAT ACC TGC TCC TCT GAT TCT GCT TTG GGT  CCC  ACC  AGC ACC  GAG GGC  CAT GGG
 S   Y   T   C   S   S   D   S   A   L   G    P    T    S   T    E   G    H   G
SL LSTD FMMR T  SMR  SL DTD SL  DRL LSF SLTL MRMR FMMR SDR FMMR FSR SLDR LTM SLSL

GCC CTC TCC ATT TCT CCT CTC AGC AGA AGT CCA GGT TCC CAT GCA GAC TTT CTG CTG
 A   L   S   I   S   P   L   S   R   S   P   G   S   H   A   D   F   L   L
DRMR LS SMR TDF SL  MRL  LS SDR RFS STL MR SLTL SMR LTM  DR  D   FL LSF LSF

ACC  CCT CTG TCA CCC ACT GCC  AGT CAG GGC  AGT CCC TCA TTC TGC GTT GGG  AGT CTG
 T    P   L   S   P   T   A    S   Q   G    S   P   S   F   C   V   G    S   L
FMMR MRL LSF  S MRMR FML DRMR STL MFR SLDR STL MRMR S   F   T  TLF SLSL STL LSF

GAA GAA GAC TCT CCC TTC CCT TCC TTT GCC CAG ATG CTG AGG GTT GGA AAA GCA AAA
 E   E   D   S   P   F   P   S   F   A   Q   M   L   R   V   G   K   A   K
 FS  FS  D  SL MRMR  F  MRL SMR FL DRMR MFR  M  LSF RSL TLF  SL  RD  DR  RD

GCA GAT GTG TGG CCC AAA ACT GCT CCA AAG AAA GAT GAG AAC AGC TTA GTT CCT CCT
 A   D   V   W   P   K   T   A   P   K   K   D   E   N   S   L   V   P   P
DR  DTD TLSF SF MRMR RD FML DRL  MR RDR  RD DTD FSR RM  SDR LLS TLF MRL MRL

GCC  CCT GTG GAC AGC GAC GGG GAG AGT GAT AAT TCA GAC CGT GTT CCT GTG  CCC  AGT
 A    P   V   D   S   D   G   E   S   D   N   S   D   R   V   P   V    P    S
DRMR MRL TLSF D  SDR  D SLSL FSR STL DTD RMTD S   D  RTL TLF MRL TLSF MRMR STL
```

```
TTT CAA AAT TCC TTC AGC CAA GCT ATT GAA GCA GCC  TTC ATG AAA CTG GAC ACA CCA
 F   Q   N   S   F   S   Q   A   I   E   A   A    F   M   K   L   D   T   P
FL  MF RMTD SMR  F  SDR MF  DRL TDF  FS  DR DRMR  F   M   RD LSF  D   FM  MR

GCT ACT TCA GAT CCC  CTC TCT GAA  GAG AAA GGA GGA  AAG AAA AGA AAA AAA CAG AAA
 A   T   S   D   P    L   S   E    E   K   G   G    K   K   R   K   K   Q   K
DRL FML  S  DTD MRMR  LS  SL  FS  FSR  RD  SL  SL  RDR  RD RTL  RD  RD MFR  RD

CAG AAG CTC CTG TTC AGC ACC TCA GTC GTC CAC ACC AAG TGA
 Q   K   L   L   F   S   T   S   V   V   H   T   K
MFR RDR  LS LSF  F  SDR FMMR S  TLS TLS LT FMMR RDR STOP
```

Homo sapiens **ring finger protein 10 (RNF10)**

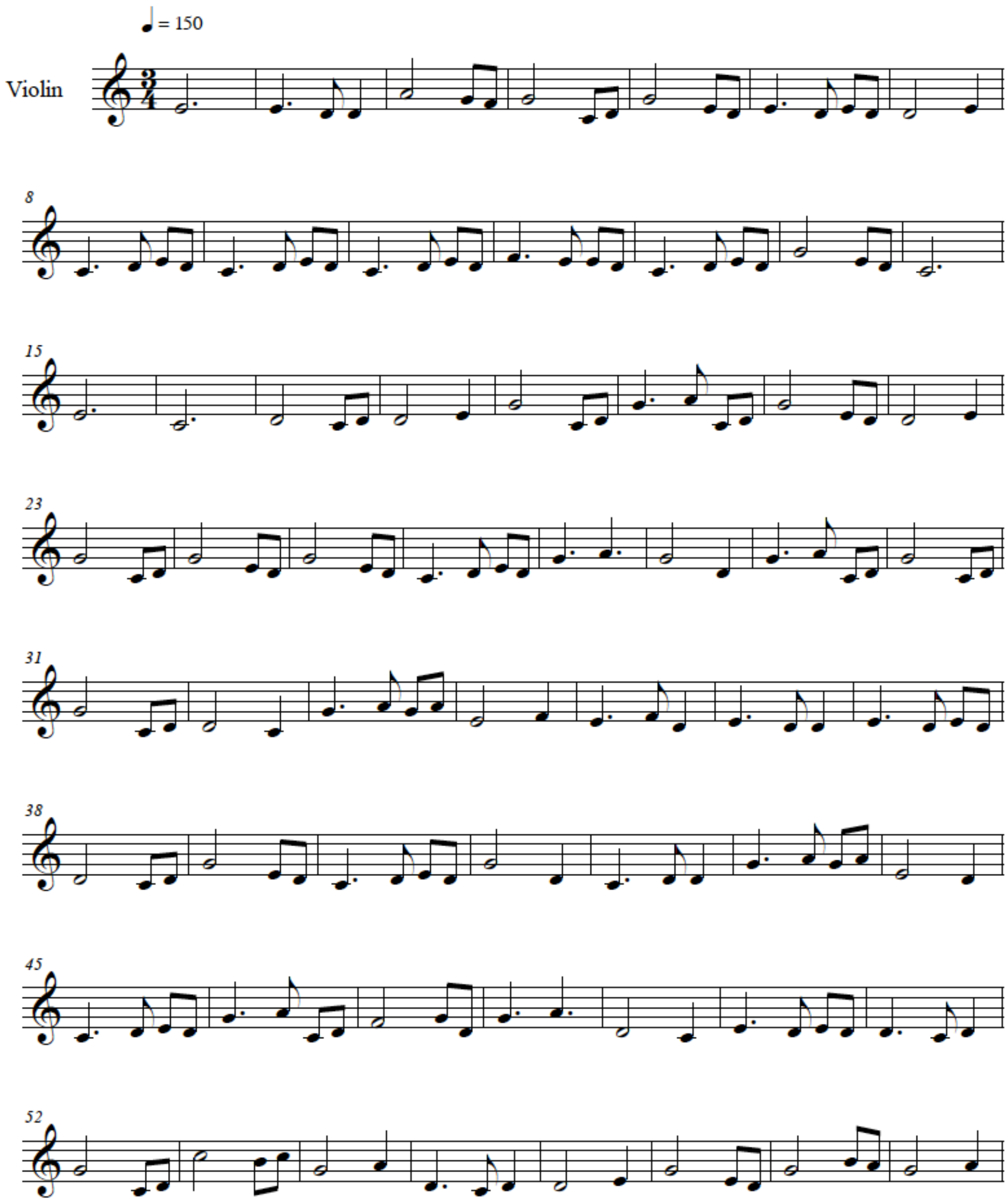

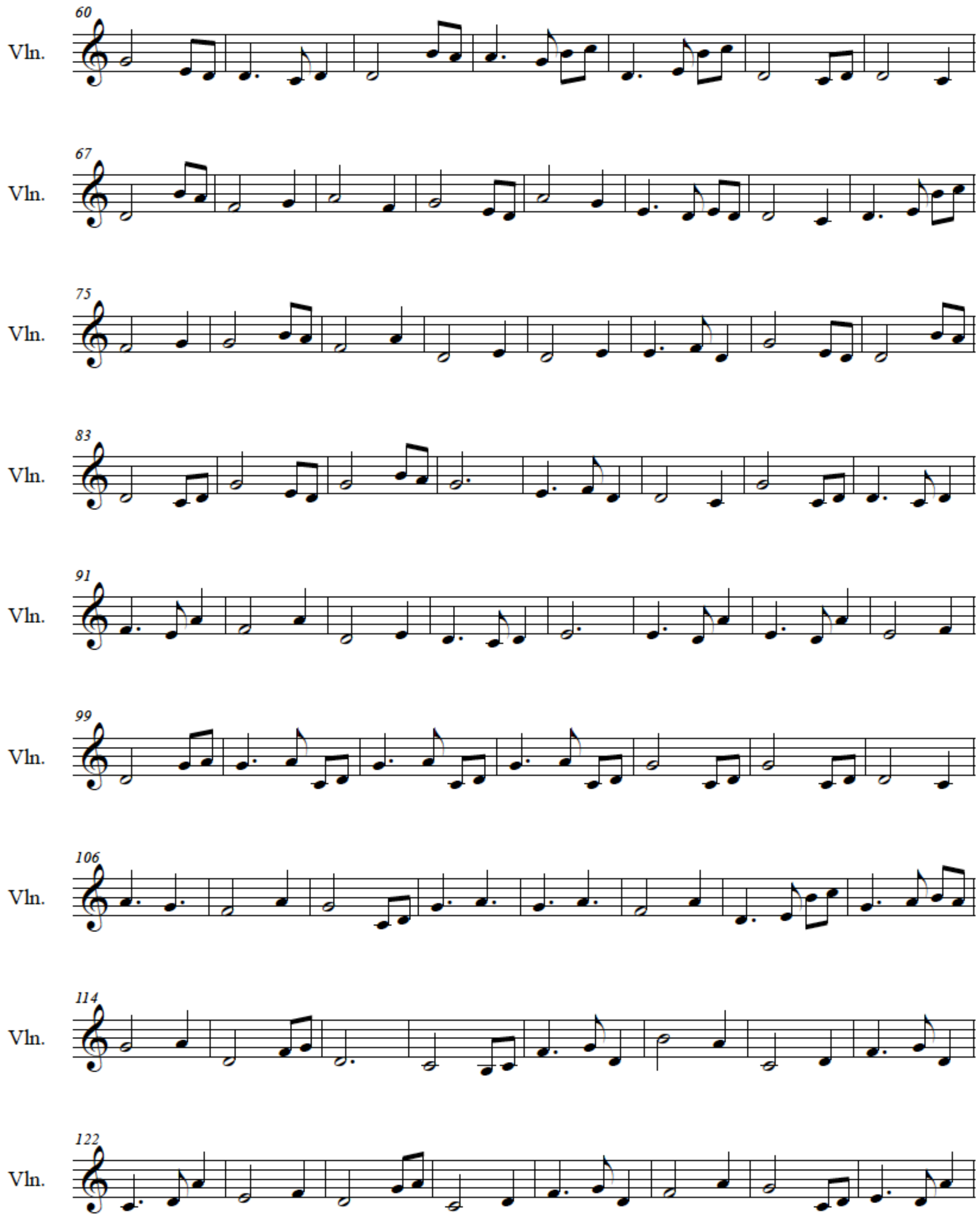
60
Vln.
67
Vln.
75
Vln.
83
Vln.
91
Vln.
99
Vln.
106
Vln.
114
Vln.
122
Vln.

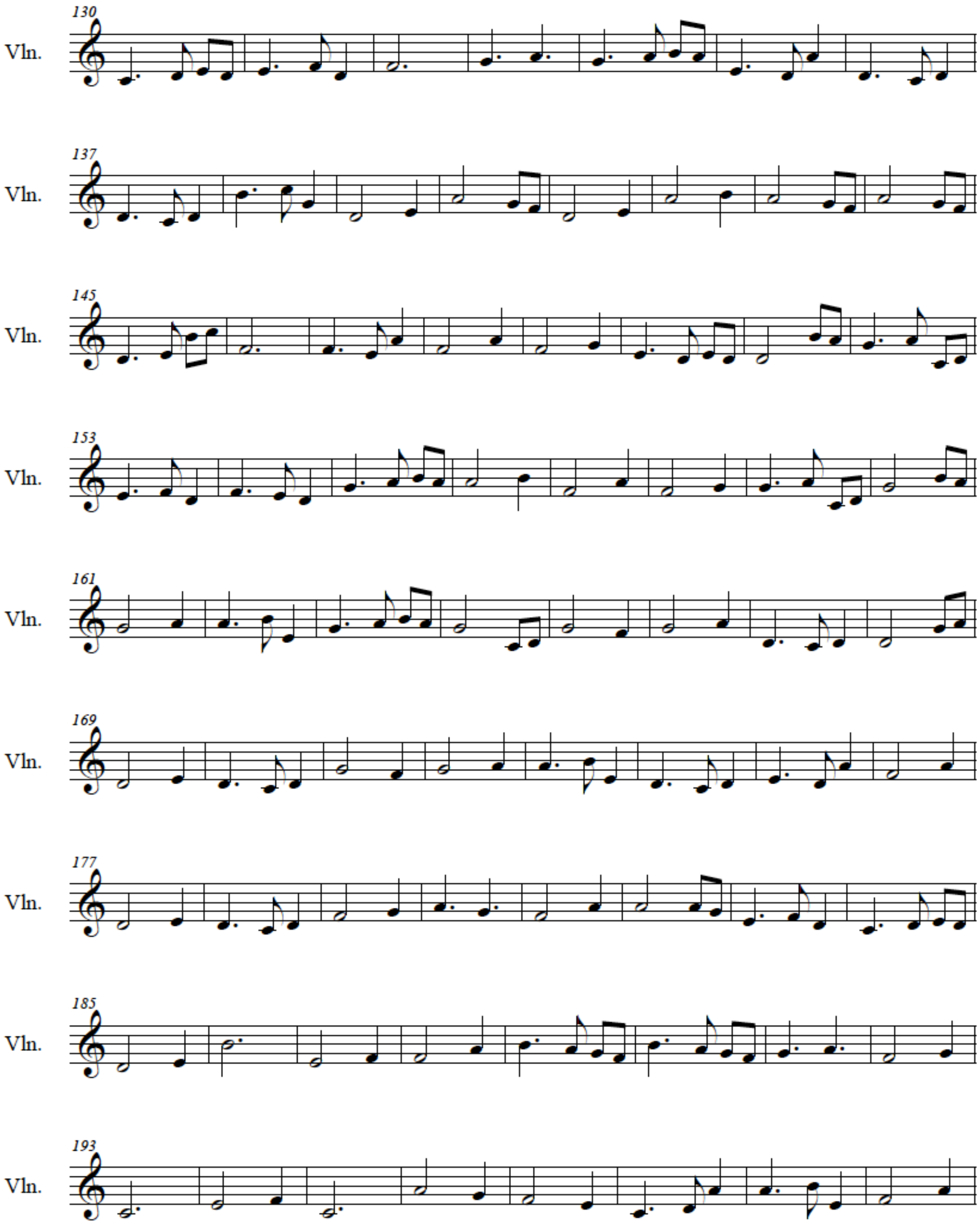
130
Vln.
137
Vln.
145
Vln.
153
Vln.
161
Vln.
169
Vln.
177
Vln.
185
Vln.
193
Vln.

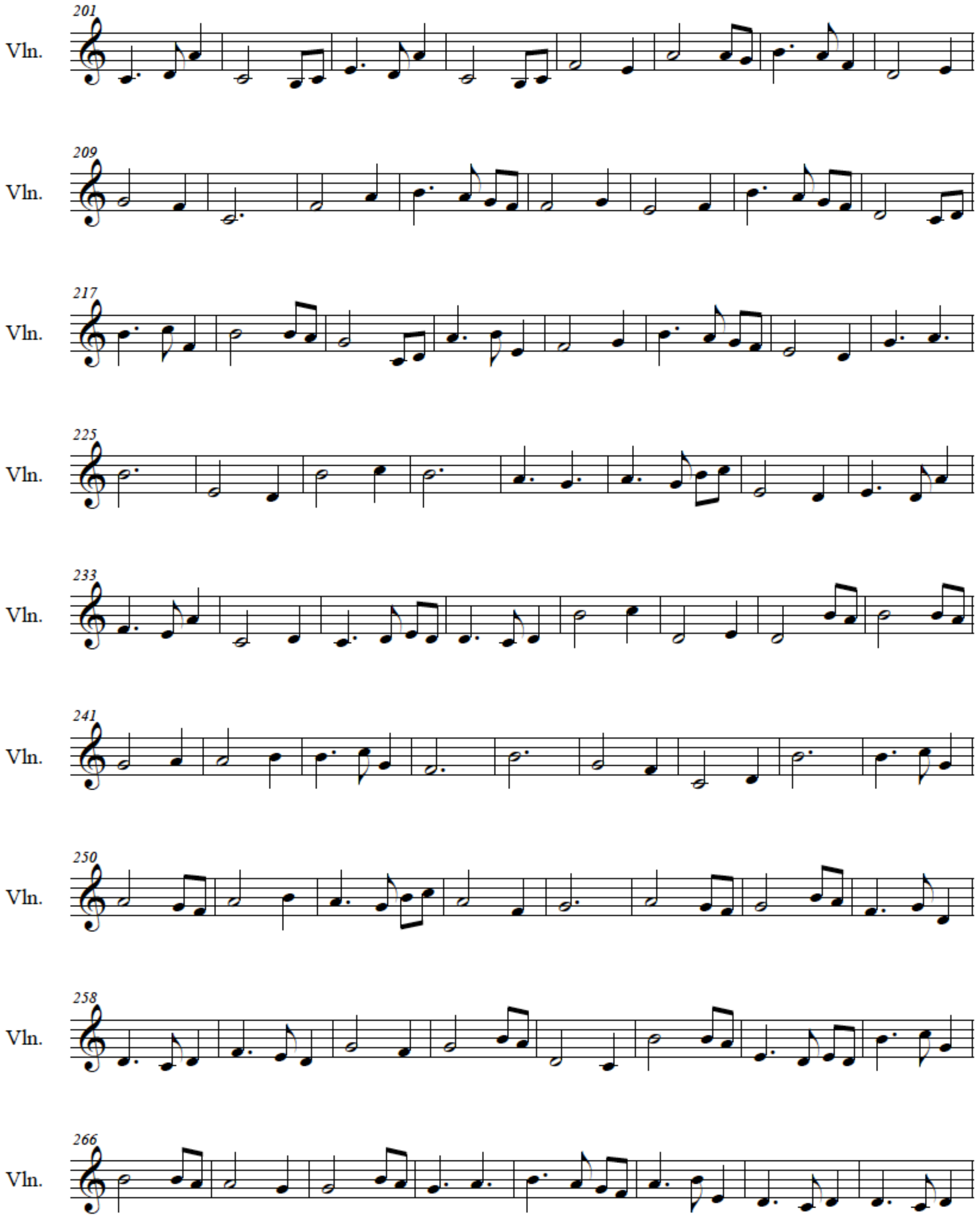
201
Vln.
209
Vln.
217
Vln.
225
Vln.
233
Vln.
241
Vln.
250
Vln.
258
Vln.
266
Vln.

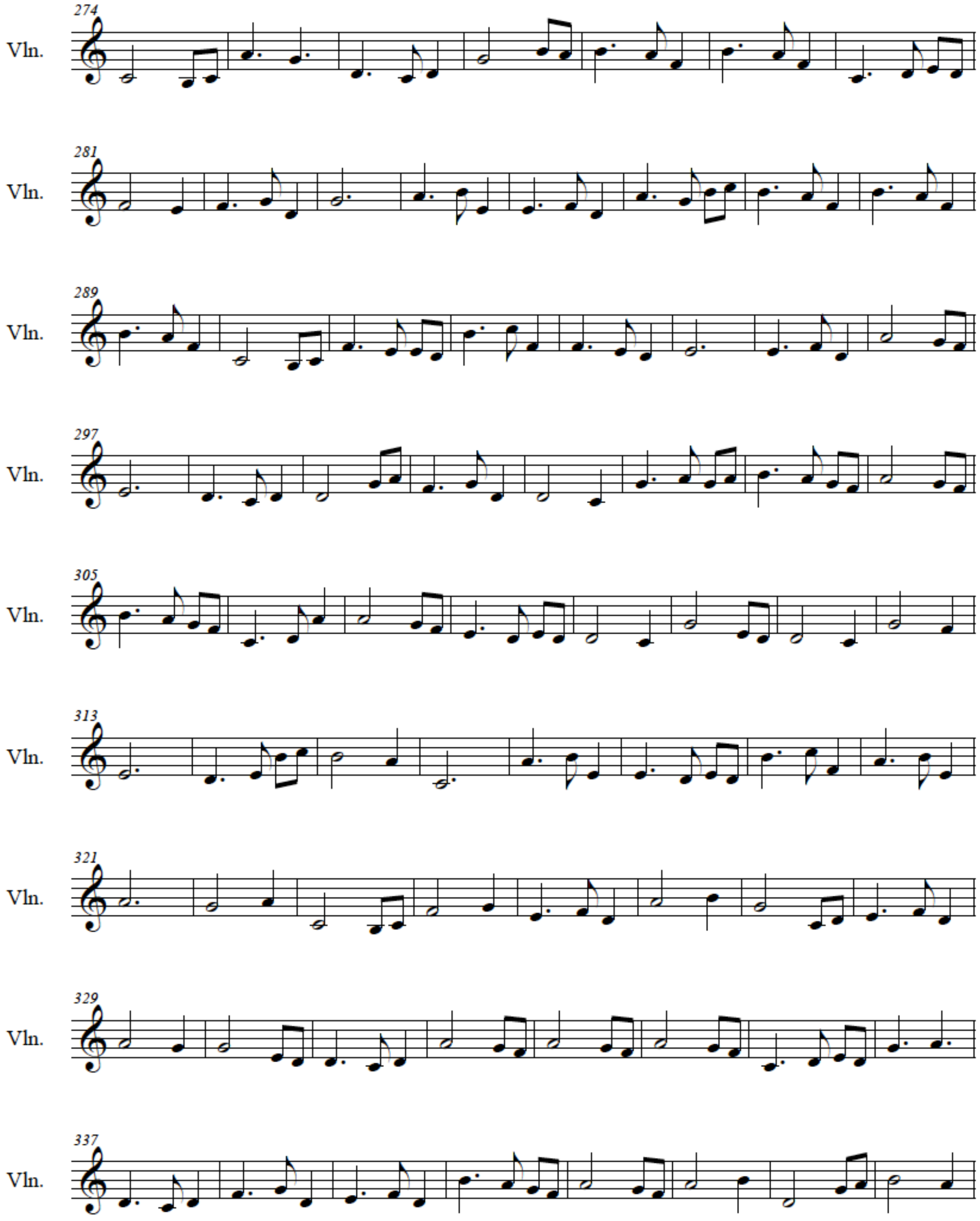
274
Vln.
281
Vln.
289
Vln.
297
Vln.
305
Vln.
313
Vln.
321
Vln.
329
Vln.
337
Vln.

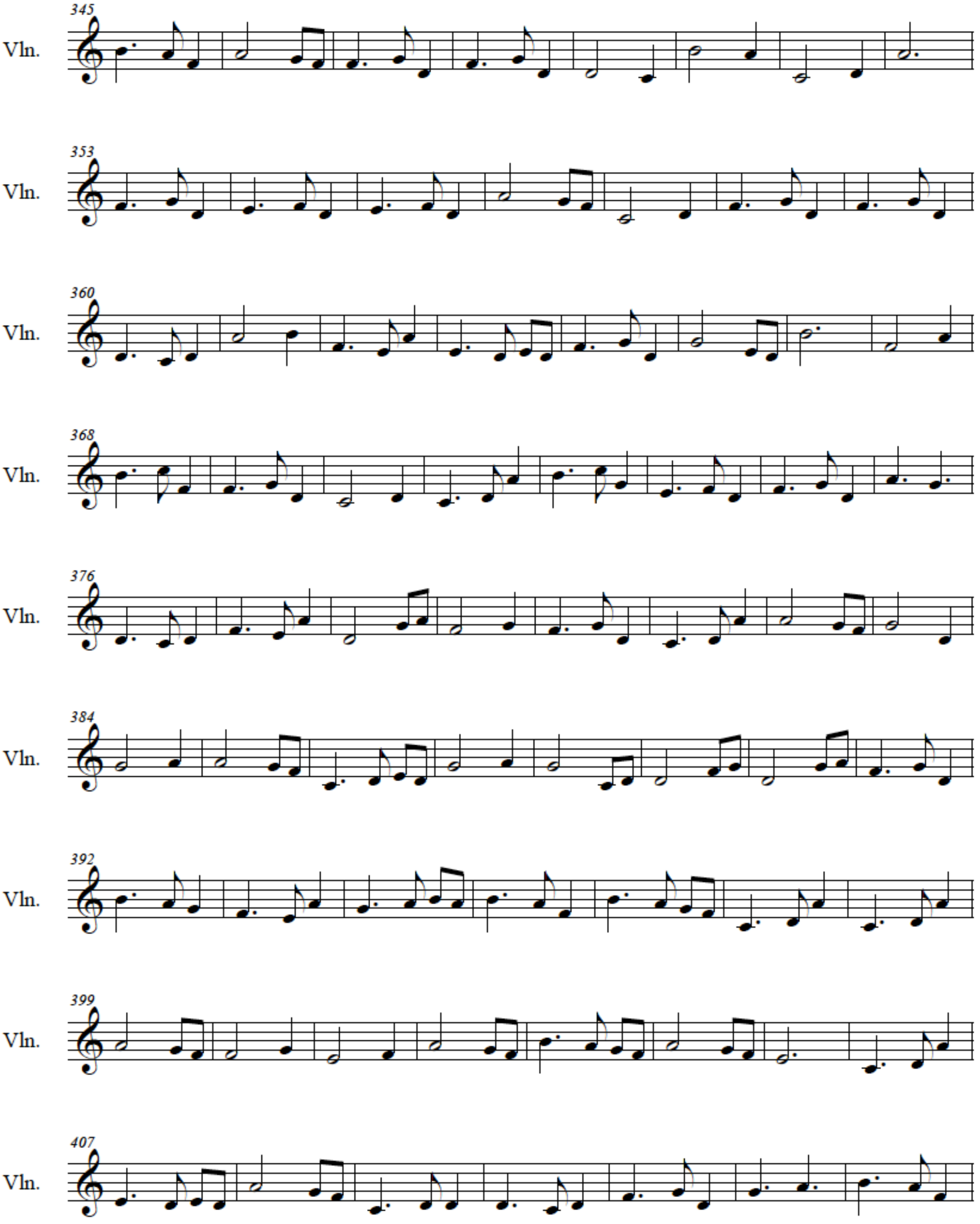
345
Vln.
353
Vln.
360
Vln.
368
Vln.
376
Vln.
384
Vln.
392
Vln.
399
Vln.
407
Vln.

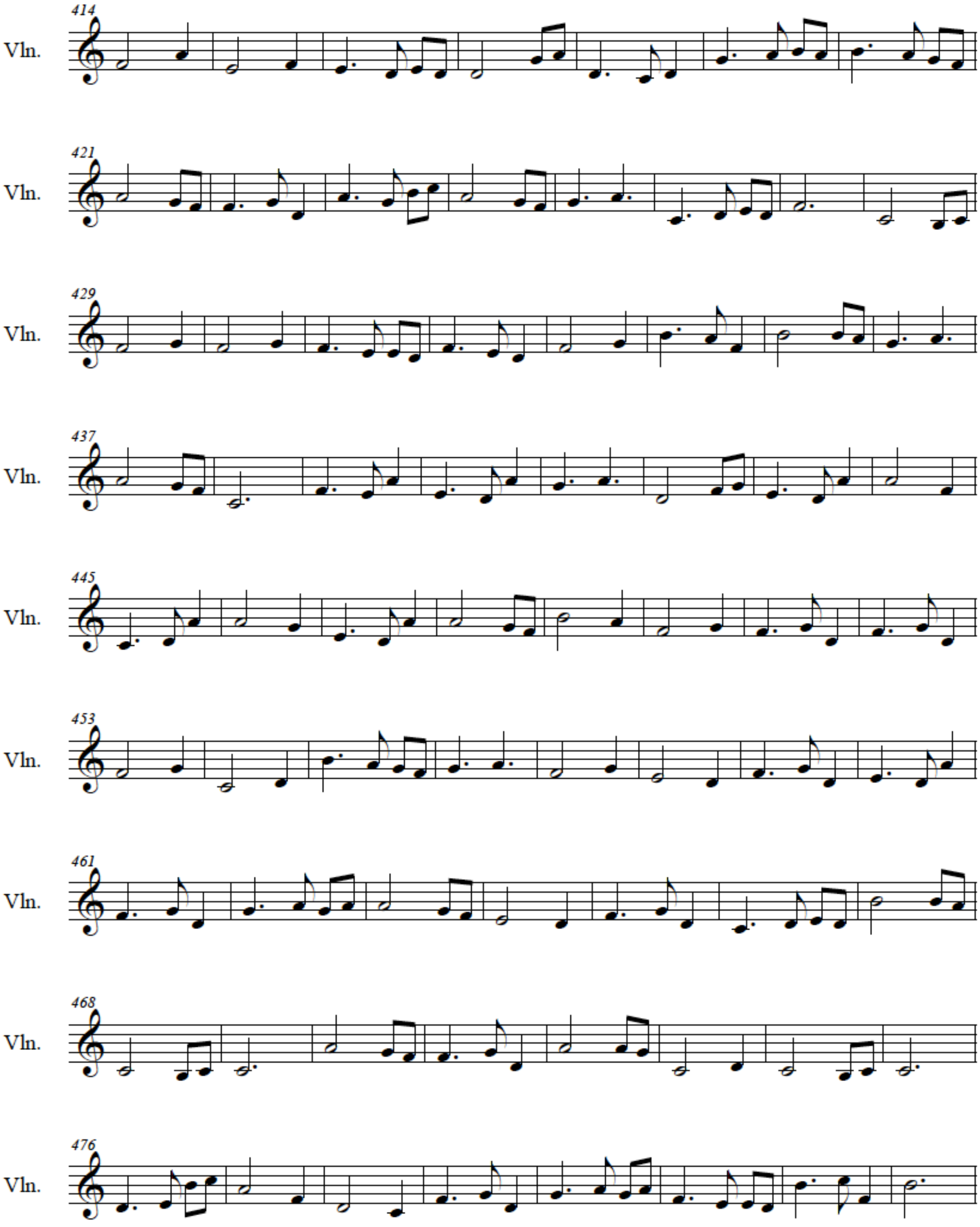
414
Vln.
421
Vln.
429
Vln.
437
Vln.
445
Vln.
453
Vln.
461
Vln.
468
Vln.
476
Vln.

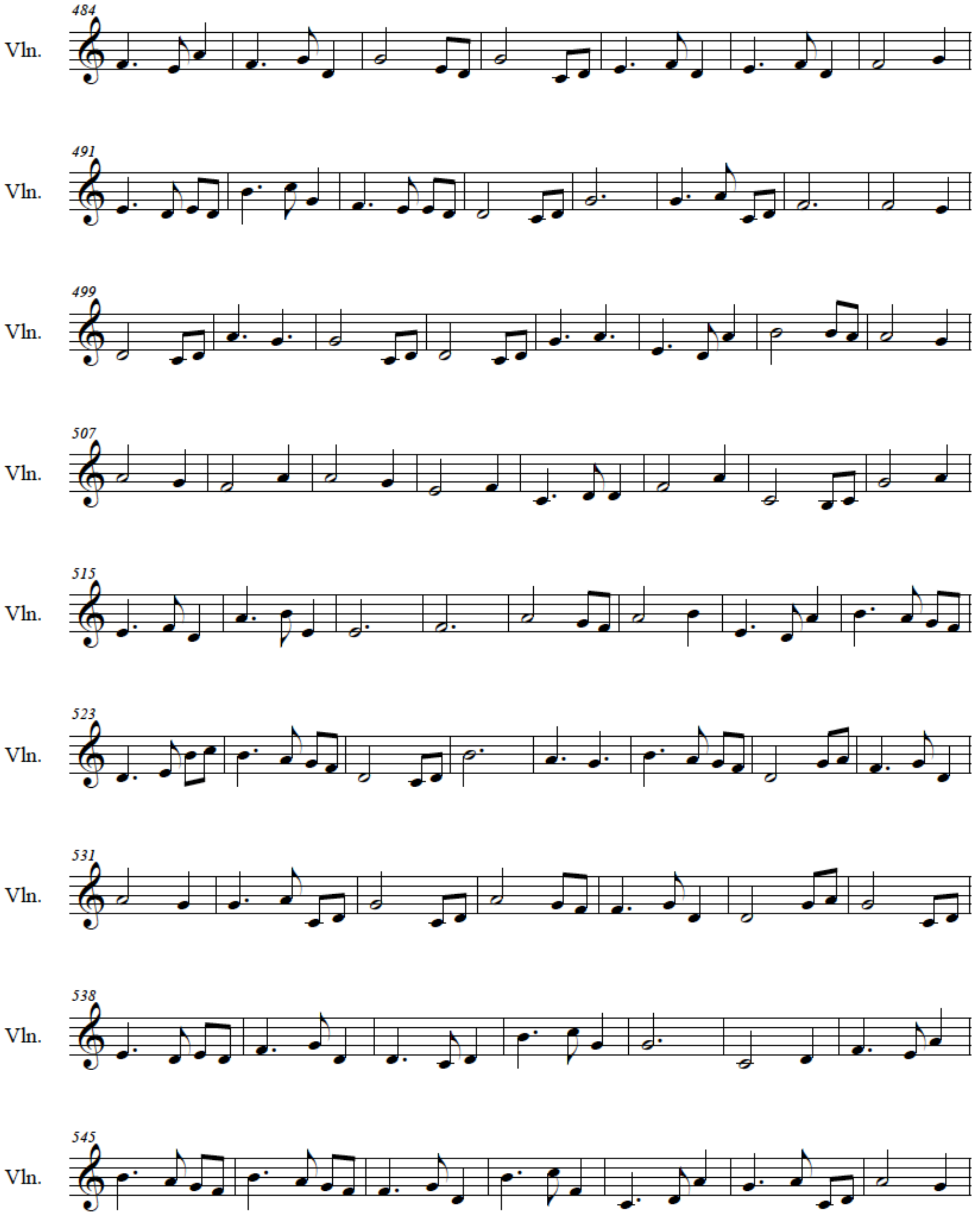
484
Vln.
491
Vln.
499
Vln.
507
Vln.
515
Vln.
523
Vln.
531
Vln.
538
Vln.
545
Vln.

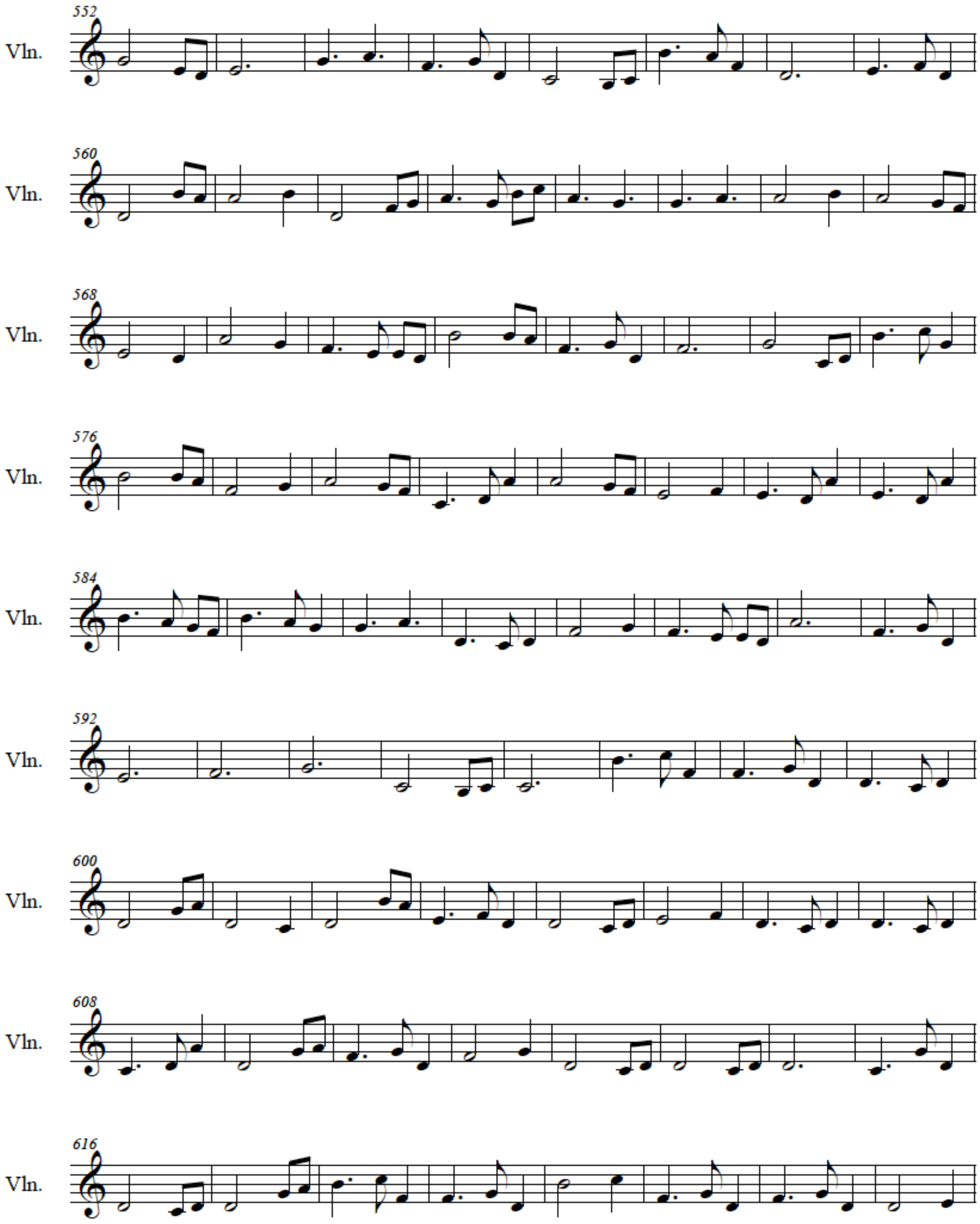
552
Vln.
560
Vln.
568
Vln.
576
Vln.
584
Vln.
592
Vln.
600
Vln.
608
Vln.
616
Vln.

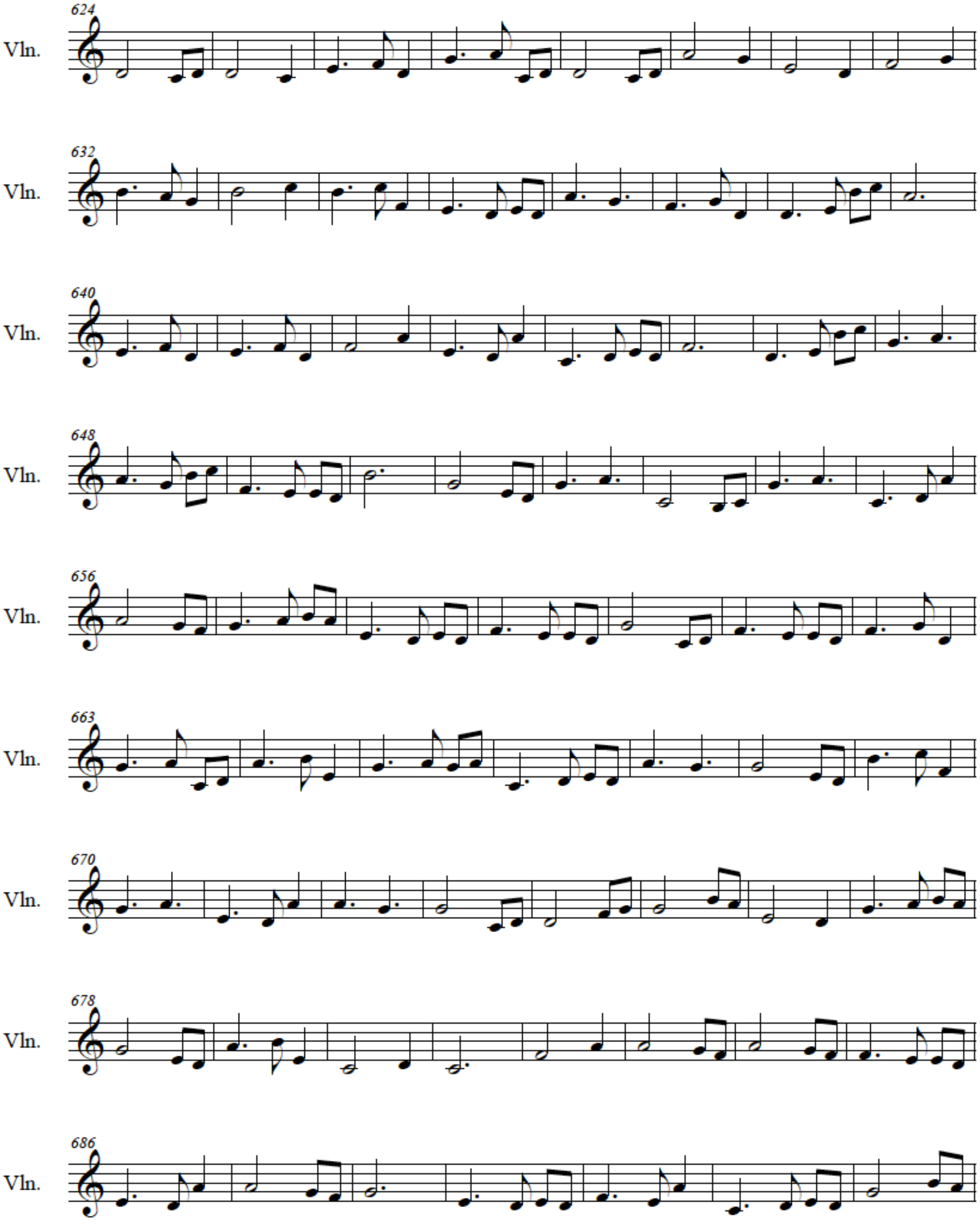
624
Vln.
632
Vln.
640
Vln.
648
Vln.
656
Vln.
663
Vln.
670
Vln.
678
Vln.
686
Vln.

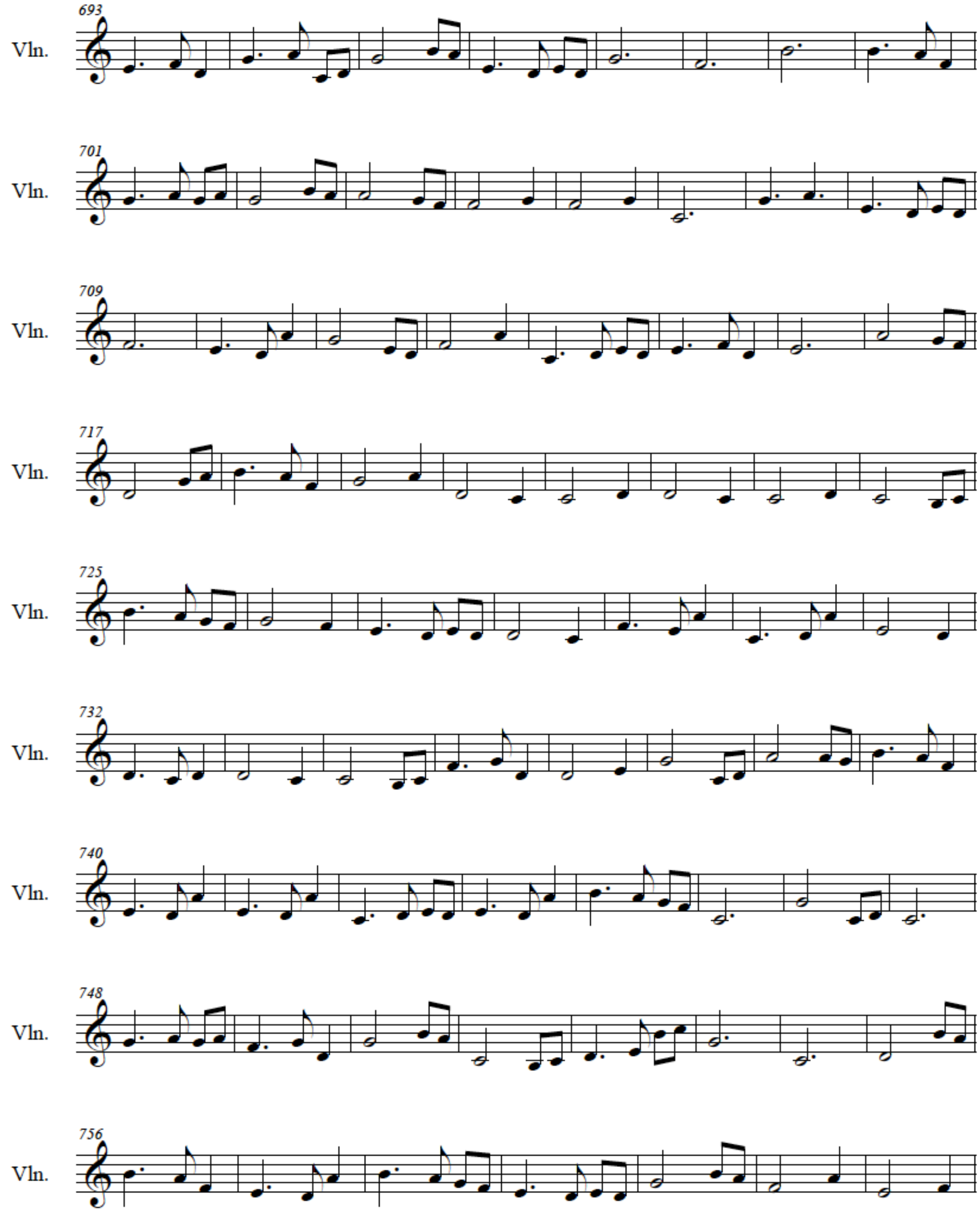
693
Vln.
701
Vln.
709
Vln.
717
Vln.
725
Vln.
732
Vln.
740
Vln.
748
Vln.
756
Vln.

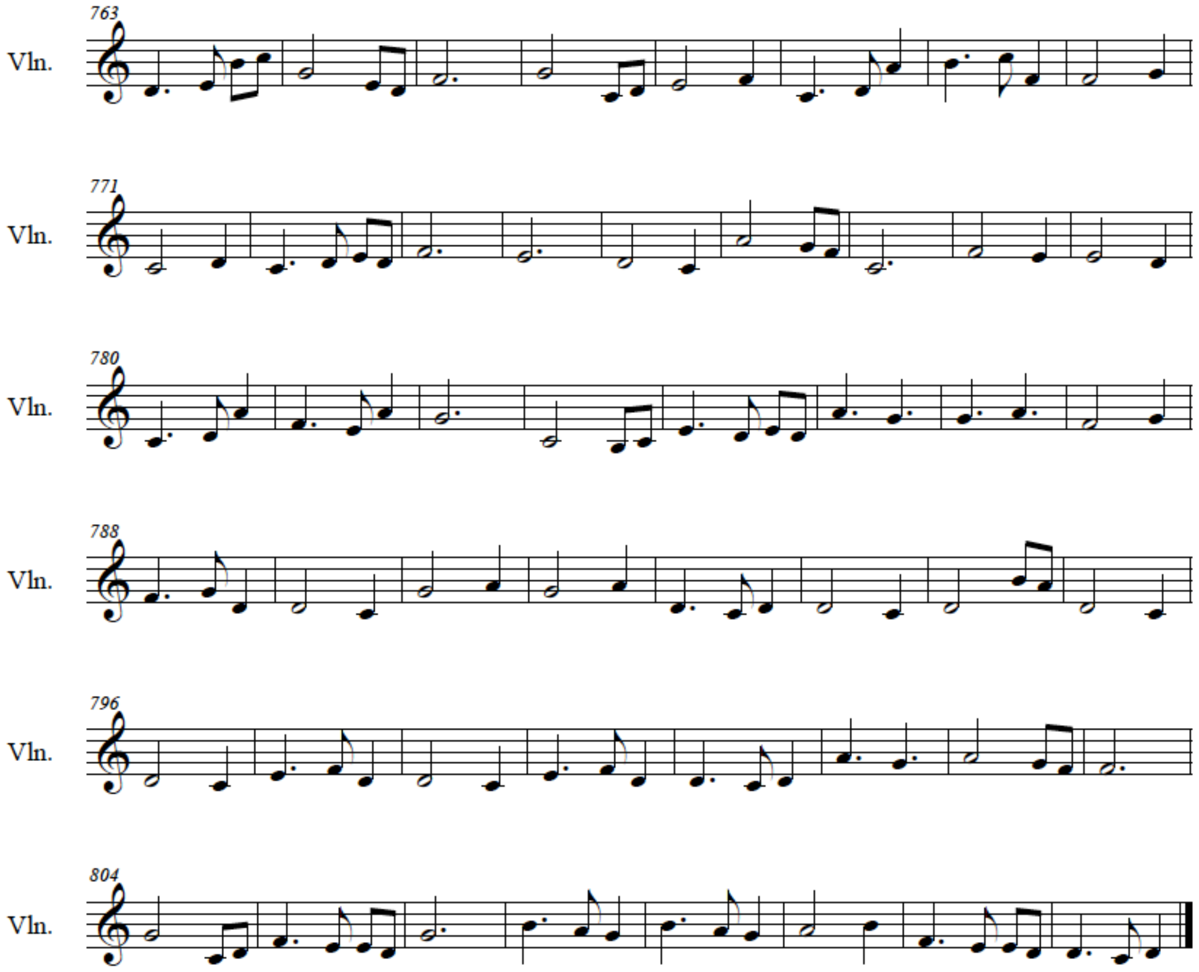
763
Vln.
771
Vln.
780
Vln.
788
Vln.
796
Vln.
804
Vln.

>NM_005481.2 *Homo sapiens* mediator complex subunit 16 (THRAP5)

```
ATG TGT GAT TTG CGG CGG CCA GCG GCA GGT GGG ATG ATG GAC TTG GCC TAC GTC TGT
 M   C   D   L   R   R   P   A   A   G   G   M   M   D   L   A   Y   V   C
 M  TTL DTD LSF RSL RSL MR  DRR DR SLTL SLSL M   M   D  LSF DRMR LS TLS TTL

GAG TGG GAG AAA TGG TCC AAG AGC ACC CAC TGC CCA TCG GTG  CCC  CTG GCC TGC GCC
 E   W   E   K   W   S   K   S   T   H   C   P   S   V    P    L   A   C   A
FSR SF  FSR  RD  SF SMR RDR SDR FMMR LT  T   MR  SR TLSF MRMR LSR DRMR T  DRMR

TGG TCC TGC CGA AAT CTC ATC GCC TTC ACC ATG GAC CTG CGC AGC GAT GAC CAG GAC
 W   S   C   R   N   L   I   A   F   T   M   D   L   R   S   D   D   Q   D
SF  SMR  T   R RMTD  LS TDS DRMR F FMMR  M   D  LSF RDR SDR DTD  D  MFR  D

CTG ACC CGC ATG ATC CAC  ATC CTG GAC ACG GAG CAC CCC  TGG GAC CTG CAC TCG ATC
 L   T   R   M   I   H    I   L   D   T   E   H   P    W   D   L   H   S   I
LSF FMMR RDR M  TDS  LT  TDS LSF  D FMR FSR  LT  MRMR  SF  D  LSF LT  SR  TDS

CCC TCA GAG CAC CAC GAG GCC A TC ACC TGC CTG GAG TGG GAC CAG TCA GGC  TCC CGG
 P   S   E   H   H   E   A    I   T   C   L   E   W   D   Q   S   G    S   R
MRMR S  FSR LT  LT  FSR DRMR TDS FMMR T  LSF FSR SF   D  MFR  S  SLDR SMR RSL

CTC CTG TCA GCA GAT GCC GAC GGG CAG ATC AAG TGC TGG AGC ATG GCG GAC CAC CTG
 L   L   S   A   D   A   D   G   Q   I   K   C   W   S   M   A   D   H   L
LS  LSF  S  DR  DTD DRMR D SLSL MFR TDS RDR  T   SF SDR  M  DRR  D  LT  LSF

GCT AAT  AGC TGG GAG AGC TCA GTG GGC  AGC CTA GTG GAG GGG GAC CCC  ATT GTG  GCC
 A   N    S   W   E   S   S   V   G    S   L   V   E   G   D   P    I   V    A
DRL RMTD SDR SF  FSR SDR  S TLSF SLDR SDR  L TLSF FSR SLSL D  MRMR TDF TLSF DRMR

CTG TCC TGG CTG CAC AAT GGT  GTG AAA CTG GCC  CTG CAC GTG GAG AAG TCG GGC  GCC
 L   S   W   L   H   N   G    V   K   L   A    L   H   V   E   K   S   G    A
LSF SMR SF  LSF LT RMTD SLTL TLSF RD LSF DRMR LSF LT TLSF FSR RDR  SR SLDR DRMR

TCC AGC TTC GGG GAG AAG TTC TCC CGA GTC AAG TTC TCA CCG TCG CTC ACG CTG TTC
 S   S   F   G   E   K   F   S   R   V   K   F   S   P   S   L   T   L   F
SMR SDR  F SLSL FSR RDR  F  SMR  R  TLS RDR  F   S  MRR SR  LS  FMR LSF  F

GGC  GGC  AAG CCC ATG GAG GGC TGG ATC GCG GTG  ACG GTC AGC GGC  CTG GTC ACC  GTG
 G    G    K   P   M   E   G   W   I   A   V    T   V   S   G    L   V   T    V
SLDR SLDR RDR MRMR M  FSR SLDR SF TDS DRR TLSF FMR TLS SDR SLDR LSF TLS FMMR TLSF
```

TCC CTG CTG AAG CCC AGC GGG CAG GTG CTG ACG TCC ACC GAG AGC CTG TGC CGG CTG
S L L K P S G Q V L T S T E S L C R L
SMR LSF LSF RDR MRMR SDR SLSL MFR TLSF LSF FMR SMR FMMR FSR SDR LSF T RSL LSF

CGC GGC CGC GTG GCC CTG GCC GAC ATC GCC TTC ACC GGC GGC GGC AAC ATC GTG GTG
R G R V A L A D I A F T G G G N I V V
RDR SLDR RDR TLSF DRMR LSF DRMR D TDS DRMR F FMMR SLDR SLDR SLDR RM TDS TLSF TLSF

GCC ACG GCG GAC GGC AGC AGC GCG TCG CCC GTG CAG TTC TAC AAG GTG TGC GTG AGC
A T A D G S S A S P V Q F Y K V C V S
DRMR FMR DRR D SLDR SDR SDR DRR SR MRMR TLSF MFR F LS RDR TLSF T TLSF SDR

GTG GTG AGC GAG AAG TGC CGT ATC GAC ACG GAG ATC CTG CCC TCC CTG TTC ATG CGC
V V S E K C R I D T E I L P S L F M R
TLSF TLSF SDR FSR RDR T RTL TDS D FMR FSR TDS LSF MRMR SMR LSF F M RDR

TGC ACC ACC GAC CTC AAC CGC AAG GAC AAG TTT CCC GCC ATC ACC CAC CTC AAG TTC
C T T D L N R K D K F P A I T H L K F
T FMMR FMMR D LS RM RDR RDR D RDR FL MRMR DRMR TDS FMMR LT LS RDR F

CTG GCC CGG GAC ATG TCG GAG CAG GTG CTT TTG TGC GCG TCC AGC CAG ACC AGC AGC
L A R D M S E Q V L L C A S S Q T S S
LSF DRMR RSL D M SR FSR MFR TLSF LF LSF T DRR SMR SDR MFR FMMR SDR SDR

ATC GTG GAG TGC TGG TCC CTG CGC AAG GAG GGA CTC CCC GTG AAC AAC ATC TTC CAG
I V E C W S L R K E G L P V N N I F Q
TDS TLSF FSR T SF SMR LSF RDR RDR FSR SL LS MRMR TLSF RM RM TDS F MFR

CAG ATC TCC CCC GTG GTT GGC GAC AAA CAG CCC ACA ATT CTC AAA TGG CGG ATC CTA
Q I S P V V G D K Q P T I L K W R I L
MFR TDS SMR MRMR TLSF TLF SLDR D RD MFR MRMR FM TDF LS RD SF RSL TDS L

TCG GCC ACC AAC GAT CTG GAC CGT GTG TCG GCC GTG GCG CTG CCC AAG CTG CCC ATC
S A T N D L D R V S A V A L P K L P I
SR DRMR FMMR RM DTD LSF D RTL TLSF SR DRMR TLSF DRR LSF MRMR RDR LSF MRMR TDS

TCG CTC ACC AAC ACC GAC CTC AAG GTG GCC AGC GAC ACA CAG TTC TAC CCT GGC CTC
S L T N T D L K V A S D T Q F Y P G L
SR LS FMMR RM FMMR D LS RDR TLSF DRMR SDR D FM MFR F LS MRL SLDR LS

```
GGG  CTG GCC  CTG GCC TTC CAC GAC GGC AGC GTC CAC ATC GTG CAC CGG CTC TCA CTG
 G    L   A    L   A   F   H   D   G   S   V   H   I   V   H   R   L   S   L
SLSL LSF DRMR LSF DRMR F   LT  D SLDR SDR TLS  LT TDS TLSF LT RSL  LS  S  LSF

CAG ACC ATG GCC GTC TTC TAC AGC TCC GCG GCC  CCG AGG CCT GTG  GAT GAG CCG GCC
 Q   T   M   A   V   F   Y   S   S   A   A    P   R   P   V    D   E   P   A
MFR FMMR M DRMR TLS  F   LS SDR SMR DRR DRMR MRR RSL MRL TLSF DTD FSR MRR DRMR

ATG AAG CGC CCC  CGC  ACC GCG GGC  CCC  GCC  GTC CAC TTA AAG GCT ATG CAG CTA TCG
 M   K   R   P    R    T   A   G    P    A    V   H   L   K   A   M   Q   L   S
 M  RDR RDR MRMR RDR FMMR DRR SLDR MRMR DRMR TLS  LT LLS RDR DRL  M  MFR  L   SR

TGG ACG TCA CTG GCC  CTG GTG  GGG  ATT GAC AGC CAC GGG  AAG CTG AGC GTG CTC CGC
 W   T   S   L   A    L   V    G    I   D   S   H   G    K   L   S   V   L   R
SF  FMR  S  LSF DRMR LSF TLSF SLSL TDF  D  SDR  LT SLSL RDR LSF SDR TLSF LS RDR

CTC TCA CCT TCC ATG GGC CAC CCG CTG GAG GTG  GGG  CTG GCG CTG CGG CAC CTG CTC
 L   S   P   S   M   G   H   P   L   E   V    G    L   A   L   R   H   L   L
 LS  S  MRL SMR  M SLDR  LT MRR LSF FSR TLSF SLSL LSF DRR LSF RSL  LT LSF  LS

TTC CTG CTG GAG TAC TGC ATG GTG ACC  GGC TAC  GAC TGG TGG  GAC ATC CTG CTG CAC
 F   L   L   E   Y   C   M   V   T    G   Y    D   W   W    D   I   L   L   H
 F  LSF LSF FSR  LS  T   M TLSF FMMR SLDR LS   D   SF  SF   D  TDS LSF LSF  LT

GTG  CAG CCC  AGT ATG GTA CAG AGC CTG GTG  GAG AAG CTG CAC GAG GAG TAC ACG CGC
 V    Q   P    S   M   V   Q   S   L   V    E   K   L   H   E   E   Y   T   R
TLSF MFR MRMR STL  M   TL MFR SDR LSF TLSF FSR RDR LSF LT  FSR FSR LS FMR  RDR

CAG ACC  GCT GCC  CTG CAG CAG GTC CTC TCC ACC  CGG ATC CTG GCC ATG AAG GCC TCG
 Q   T    A   A    L   Q   Q   V   L   S   T    R   I   L   A   M   K   A   S
MFR FMMR DRL DRMR LSF MFR MFR TLS  LS SMR FMMR RSL TDS LSF DRMR M  RDR DRMR SR

CTC TGC AAG CTG TCG CCC TGC ACG GTG  ACC  CGC GTG TGC GAC TAC CAC ACC  AAG CTC
 L   C   K   L   S   P   C   T   V    T    R   V   C   D   Y   H   T    K   L
LS   T  RDR LSF SR MRMR  T  FMR TLSF FMMR RDR TLSF T   D   LS  LT FMMR RDR  LS

TTC CTC ATC GCC  ATC AGC TCC ACC  CTG AAG TCG CTG CTG CGC CCC CAC TTT CTC AAC
 F   L   I   A    I   S   S   T    L   K   S   L   L   R   P   H   F   L   N
 F   LS TDS DRMR TDS SDR SMR FMMR_LSF RDR  SR LSF LSF RDR MRMR LT  FL  LS  RM
```

```
ACG CCT GAC AAG AGC CCC  GGC GAC CGG CTG ACC  GAG ATC TGC ACC AAG ATC  ACC GAC
 T   P   D   K   S   P    G   D   R   L   T    E   I   C   T   K   I    T   D
FMR MRL  D  RDR SDR MRMR SLDR D  RSL LSF FMMR FSR TDS  T FMMR RDDR TDS FMMR D

GTC GAC ATT GAC AAG GTC ATG ATC AAC CTC AAG ACG GAG GAA TTT GTG  CTG GAC ATG
 V   D   I   D   K   V   M   I   N   L   K   T   E   E   F   V    L   D   M
TLS  D  TDF  D  RDR TLS  M  TDS  RM  LS RDR FMR FSR  FS  FL TLSF LSF  D   M

AAC ACA CTG CAG GCG CTG CAG CAG CTC TTG CAG TGG GTG  GGC GAC TTC GTG CTG TAC
 N   T   L   Q   A   L   Q   Q   L   L   Q   W   V    G   D   F   V   L   Y
RM  FM  LSF MFR DRR LSF MFR MFR  LS LSF MFR  SF TLSF SLDR D   F TLSF LSF  LS

CTG CTG GCC  AGC CTA CCC AAC  CAG GGT  TCC CTG CTG AGG CCG GGC CAC AGC TTT CTG
 L   L   A    S   L   P   N    Q   G    S   L   L   R   P   G   H   S   F   L
LSF LSF DRMR SDR  L  MRMR RM  MFR SLTL SMR LSF LSF RSL MRR SLDR LT SDR  FL LSF

CGG GAC GGC ACC TCG CTG GGC ATG CTT CGG GAA TTG ATG GTG GTC ATC CGC ATC TGG
 R   D   G   T   S   L   G   M   L   R   E   L   M   V   V   I   R   I   W
RSL  D SLDR FMMR SR LSF SLDR M   LF RSL  FS LSF  M TLSF TLS TDS RDR TDS  SF

GGC CTT CTG AAG CCC  AGC TGC CTG CCC  GTG  TAT  ACG GCC  ACC TCG GAT ACC CAG GAC
 G   L   L   K   P    S   C   L   P    V    Y    T   A    T   S   D   T   Q   D
SLDR LF LSF RDR MRMR SDR  T  LSF MRMR TLSF LSTD FMR DRMR FMMR SR DTD FMMR MFR D

AGC ATG TCC CTG CTC TTC CGC CTG CTC ACC AAG CTC TGG ATC TGC TGT CGC GAT GAG
 S   M   S   L   L   F   R   L   L   T   K   L   W   I   C   C   R   D   E
SDR  M  SMR LSF  LS  F  RDR LSF LS FMMR RDR  LS  SR TDS  T  TTL RDR DTD FSR

GGC CCA GCG AGC GAG CCG GAC GAG GCG CTG GTG G AT GAA TGC TGC CTG CTG CCC  AGC
 G   P   A   S   E   P   D   E   A   L   V    D   E   C   C   L   L   P    S
SLDR MR DRR SDR FSR MRR  D  FSR DRR LSF TLSF DTD FS   T   T  LSF LSF MRMR_SDR

CAG CTG CTT ATC CCC  AGC CTG GAC TGG CTG CCA GCC  AGC GAC GGC CTG GTT AGC CGC
 Q   L   L   I   P    S   L   D   W   L   P   A    S   D   G   L   V   S   R
MFR LSF  LF TDS MRMR SDR LSF  D   SF LSF  MR DRMR SDR  D SLDR LSF TLF SDR RDR

CTG CAG CCC  AAG CAG CCC CTT CGT CTG CAG TTT GGC CGG GCG CCC  ACG CTG CCT GGC
 L   Q   P    K   Q   P   L   R   L   Q   F   G   R   A   P    T   L   P   G
LSF MFR MRMR RDR MFR MRMR LF RTL LSF MFR FL SLDR RSL DRR MRMR FMR LSF MRL SLDR
```

AGT GCT GCC ACC CTG CAG CTC GAC GGC CTC GCC AGG GCC CCA GGC CAG CCC AAG ATC
 S   A   A   T   L   Q   L   D   G   L   A   R   A   P   G   Q   P   K   I
STL DRL DRMR FMMR LSF MFR LS D SLDR LS DRMR RSL DRMR MR SLDR MFR MRMR RDR TDS

GAC CAC CTG CGG AGG CTG CAC CTT GGC GCT TGC CCC ACG GAG GAA TGC AAG GCC TGC
 D   H   L   R   R   L   H   L   G   A   C   P   T   E   E   C   K   A   C
 D   LT LSF RSL RSL LSF LT LF SLDR DRL T MRMR FMR FSR FS T RDR DRMR T

ACC AGG TGC GGC TGT GTC ACC ATG CTC AAG TCG CCC AAC AGA ACC ACG GCG GTG AAG
 T   R   C   G   C   V   T   M   L   K   S   P   N   R   T   T   A   V   K
FMMR RSL T SLDR TTL TLS FMMR M   LS RDR SR MRMR RM RFS FMMR FMR DRR TLSF RDR

CAG TGG GAG CAG CGC TGG ATC AAG AAC TGC CTG GCT GTT GAA GGC CGT GGG CCG GAC
 Q   W   E   Q   R   W   I   K   N   C   L   A   V   E   G   R   G   P   D
MFR SF FSR MFR RDR SF TDS RDR RM T LSF DRL TLF FS SLDR RTL SLSL MRR D

GCC TGC GTG ACC AGC AGA GCT TCT GAG GAA GCC CCT GCC TTT GTC CAG CTG GGC CCG
 A   C   V   T   S   R   A   S   E   E   A   P   A   F   V   Q   L   G   P
DRMR T TLSF FMMR SDR RFS DRL SL FSR FS DRMR MRL DRMR FL TLS MFR LSF SLDR MRR

CAG TCC ACA CAC CAC TCT CCC AGG ACC CCC AGA TCC CTG GAC CAT CTG CAT CCA GAG
 Q   S   T   H   H   S   P   R   T   P   R   S   L   D   H   L   H   P   E
MFR SMR FM LT LT SL MRMR RSL FMMR MRMR RFS SMR LSF D LTM LSF LTM MR FSR

GAC CGT CCG TGA
 D   R   P
 D RTL MRR STOP

Homo sapiens mediator complex subunit 16 (THRAP5)

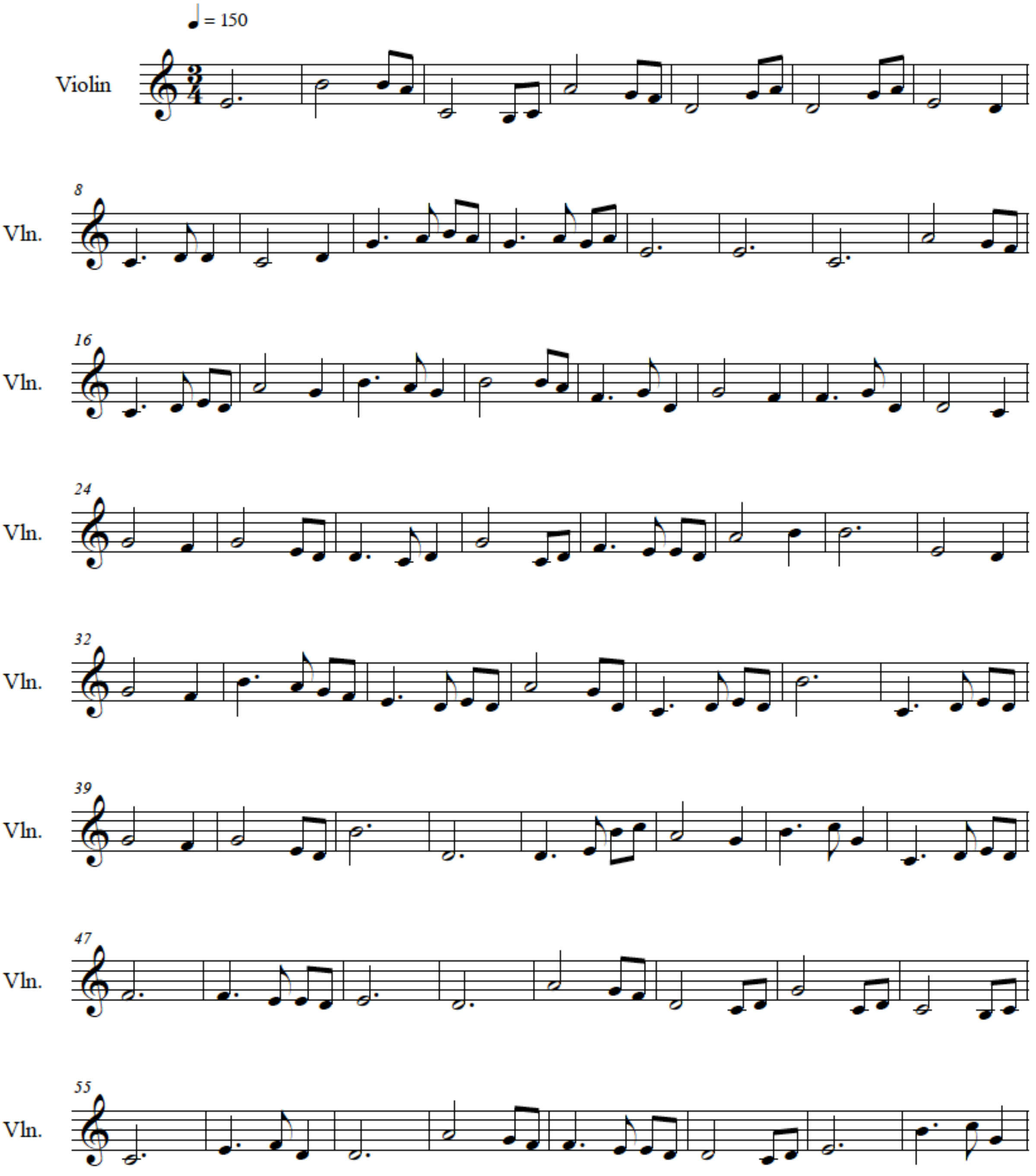

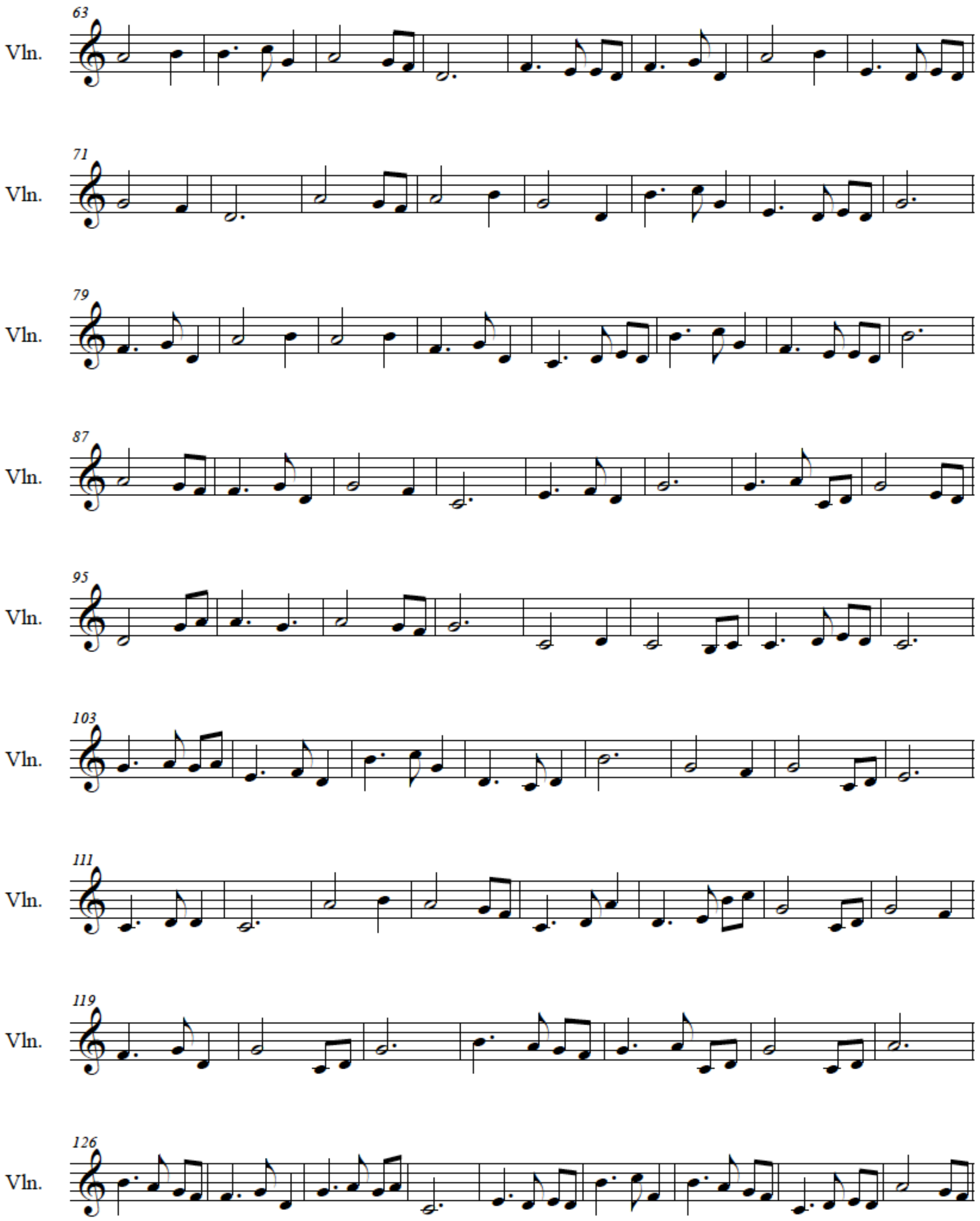
63
Vln.
71
Vln.
79
Vln.
87
Vln.
95
Vln.
103
Vln.
111
Vln.
119
Vln.
126
Vln.

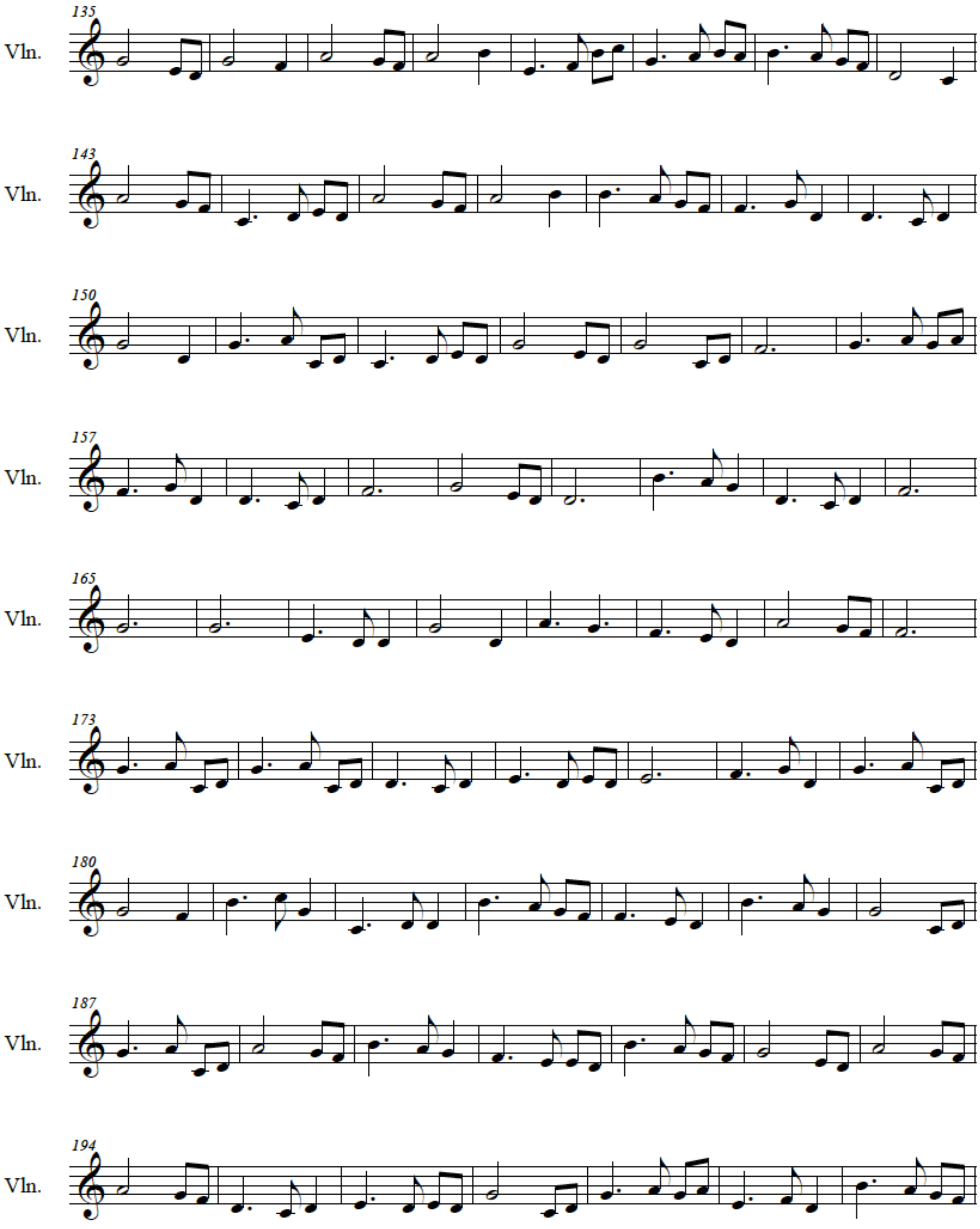
135
Vln.
143
Vln.
150
Vln.
157
Vln.
165
Vln.
173
Vln.
180
Vln.
187
Vln.
194
Vln.

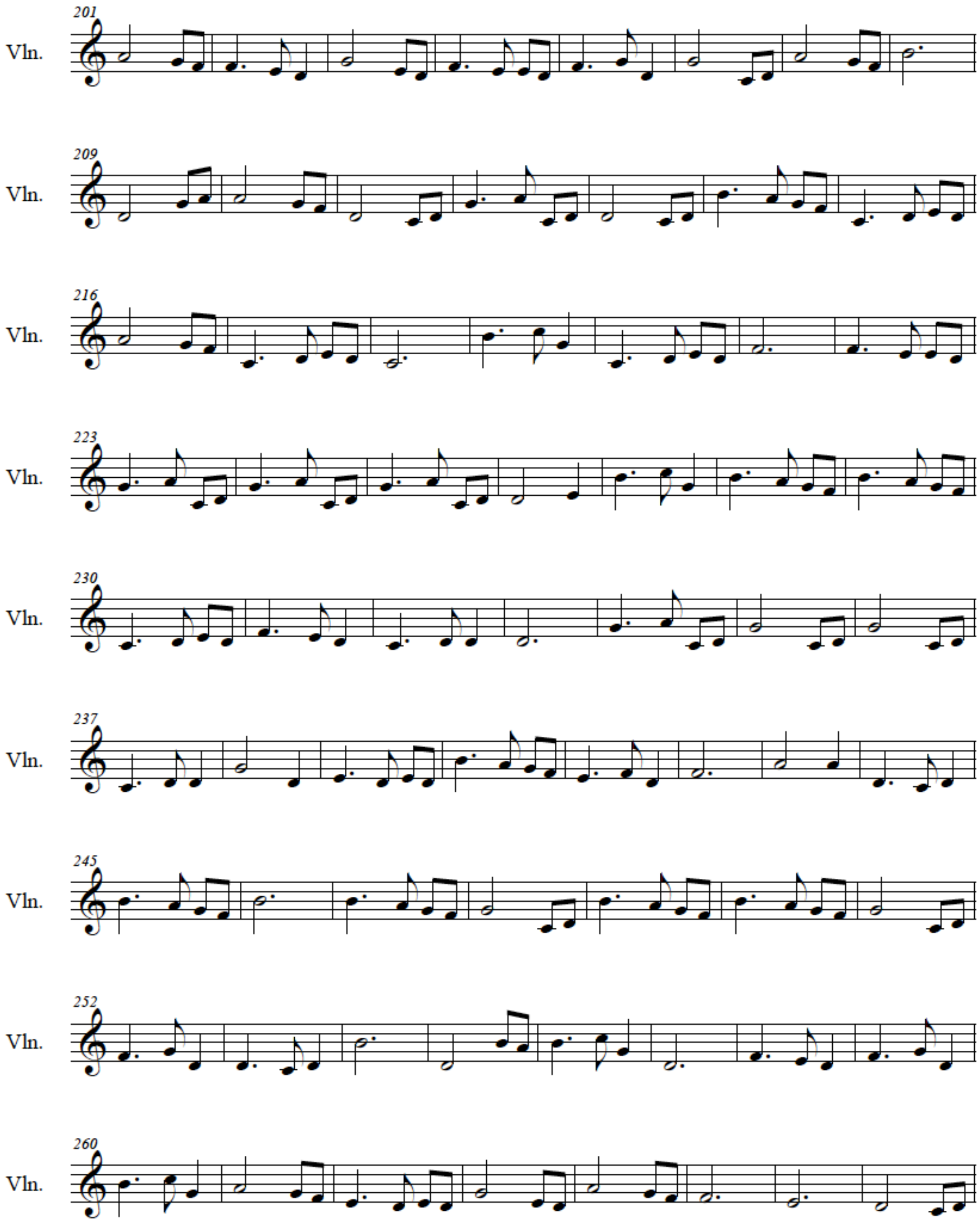
201
Vln.
209
Vln.
216
Vln.
223
Vln.
230
Vln.
237
Vln.
245
Vln.
252
Vln.
260
Vln.

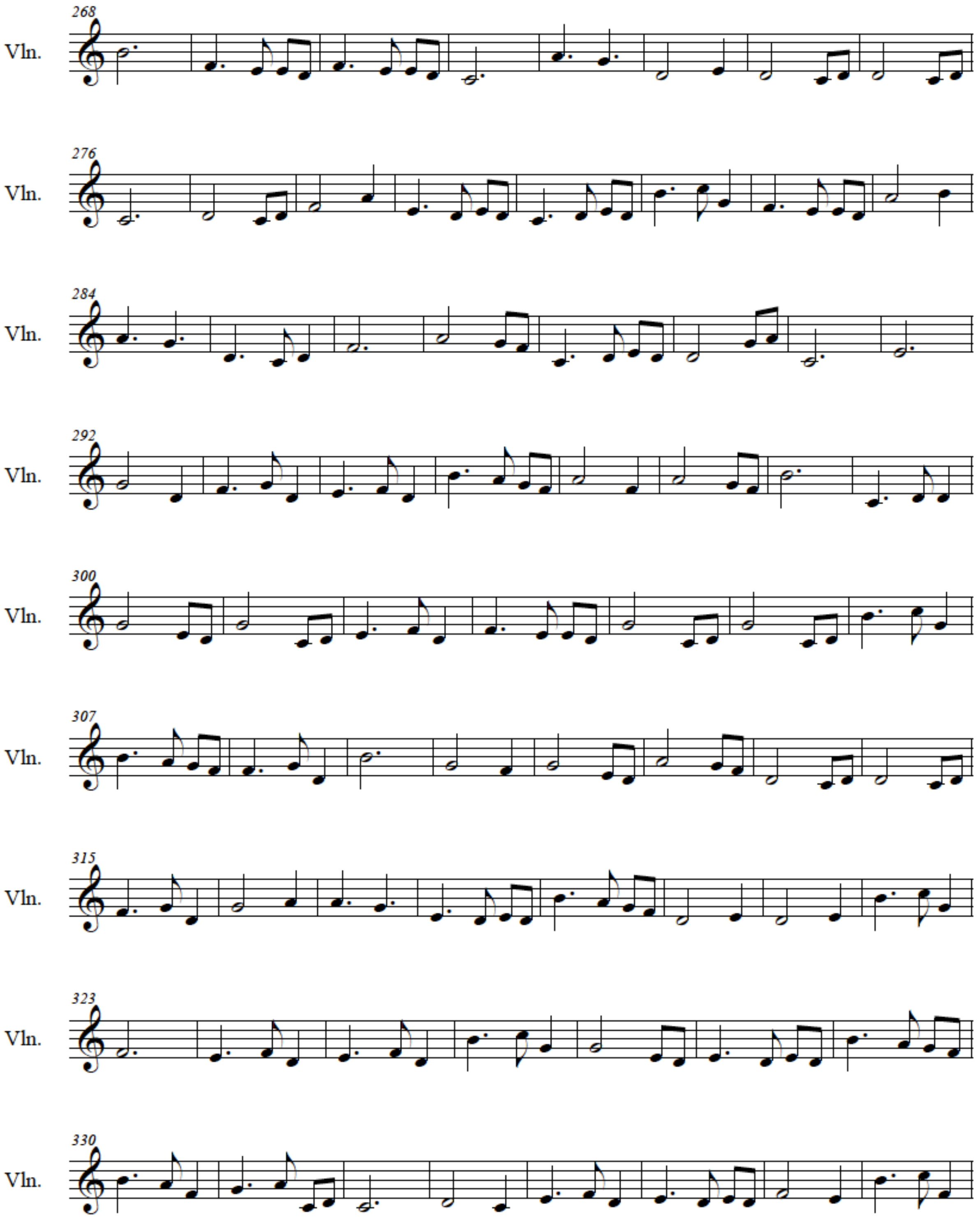
268
Vln.
276
Vln.
284
Vln.
292
Vln.
300
Vln.
307
Vln.
315
Vln.
323
Vln.
330
Vln.

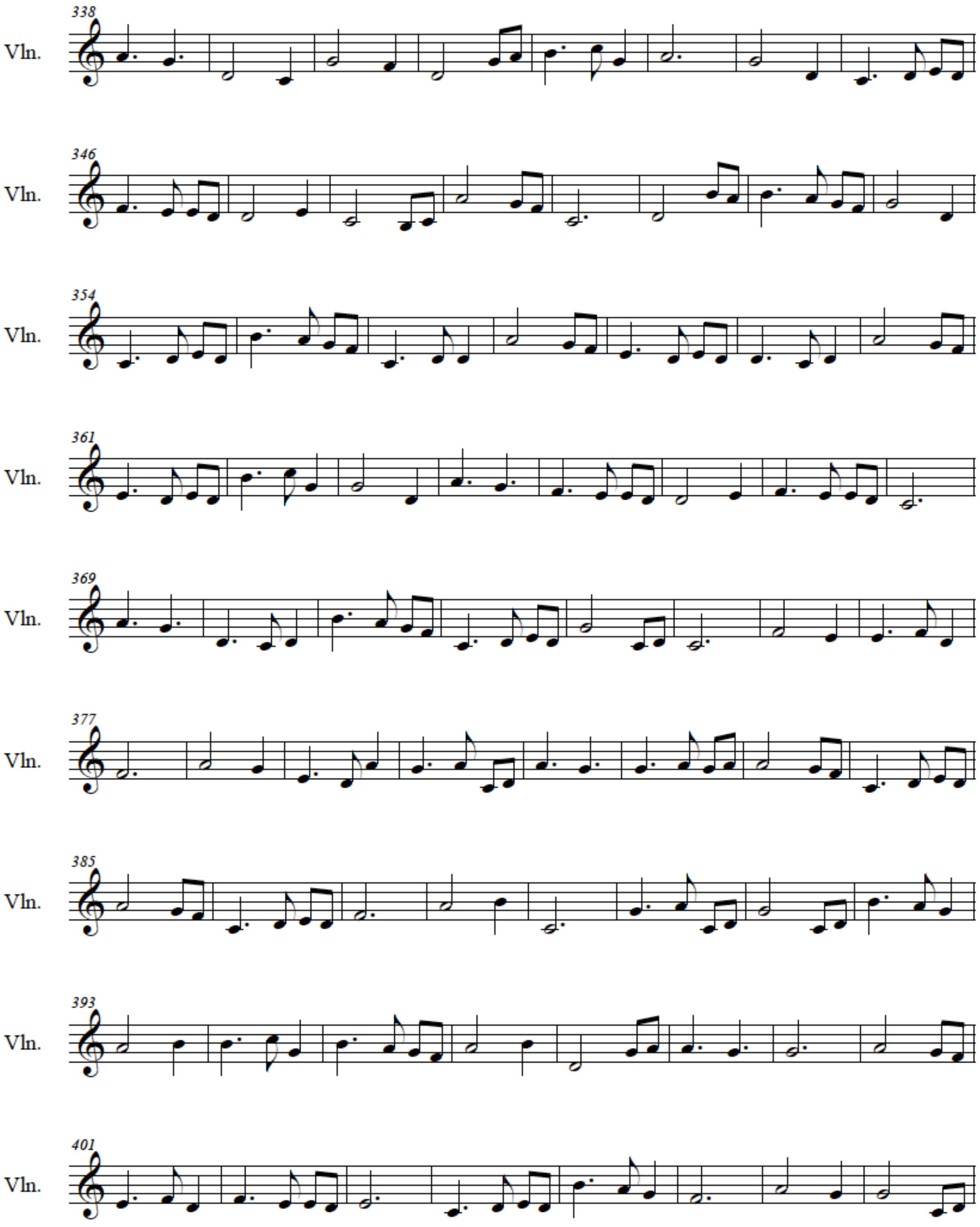
338
Vln.
346
Vln.
354
Vln.
361
Vln.
369
Vln.
377
Vln.
385
Vln.
393
Vln.
401
Vln.

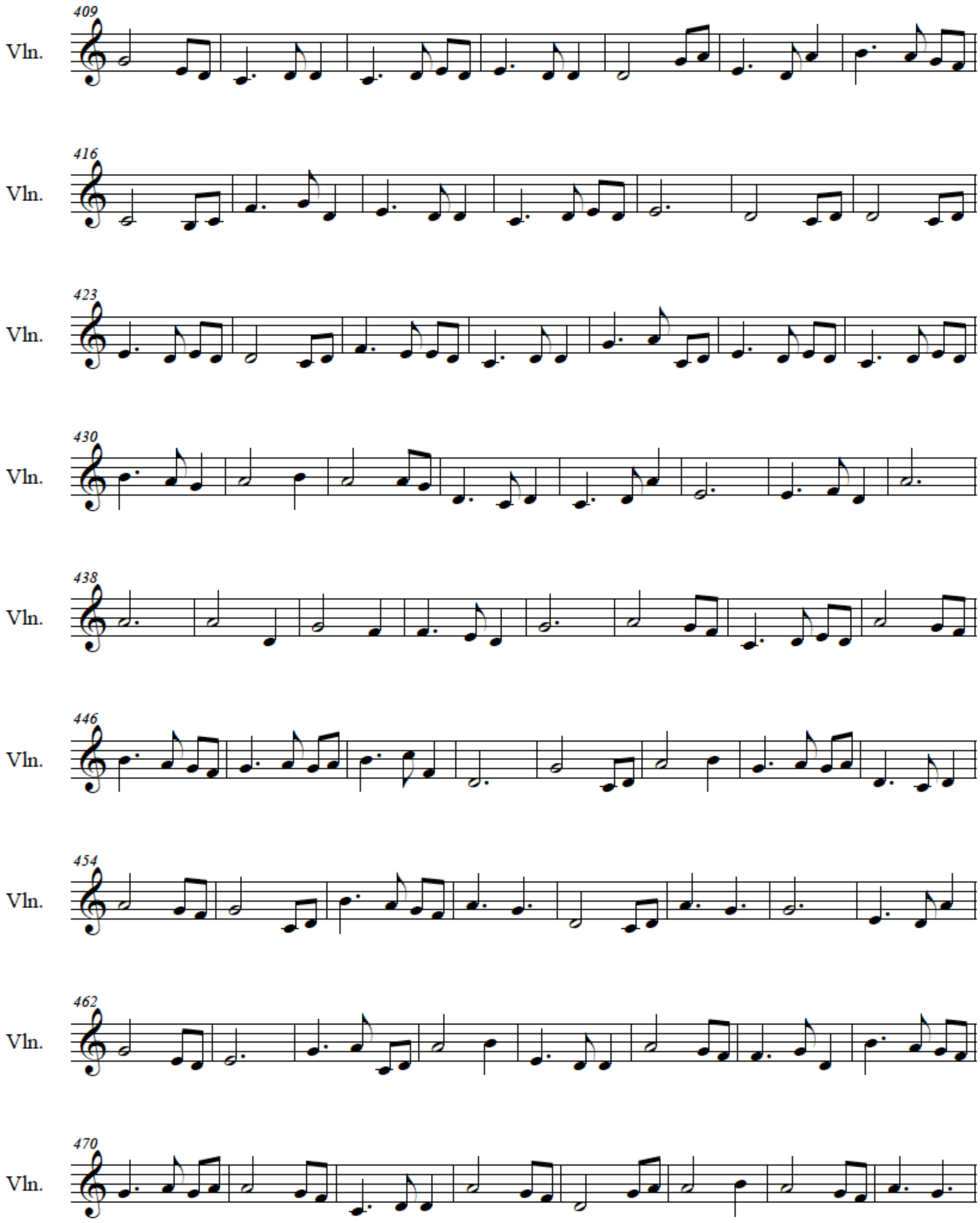
409
Vln.
416
Vln.
423
Vln.
430
Vln.
438
Vln.
446
Vln.
454
Vln.
462
Vln.
470
Vln.

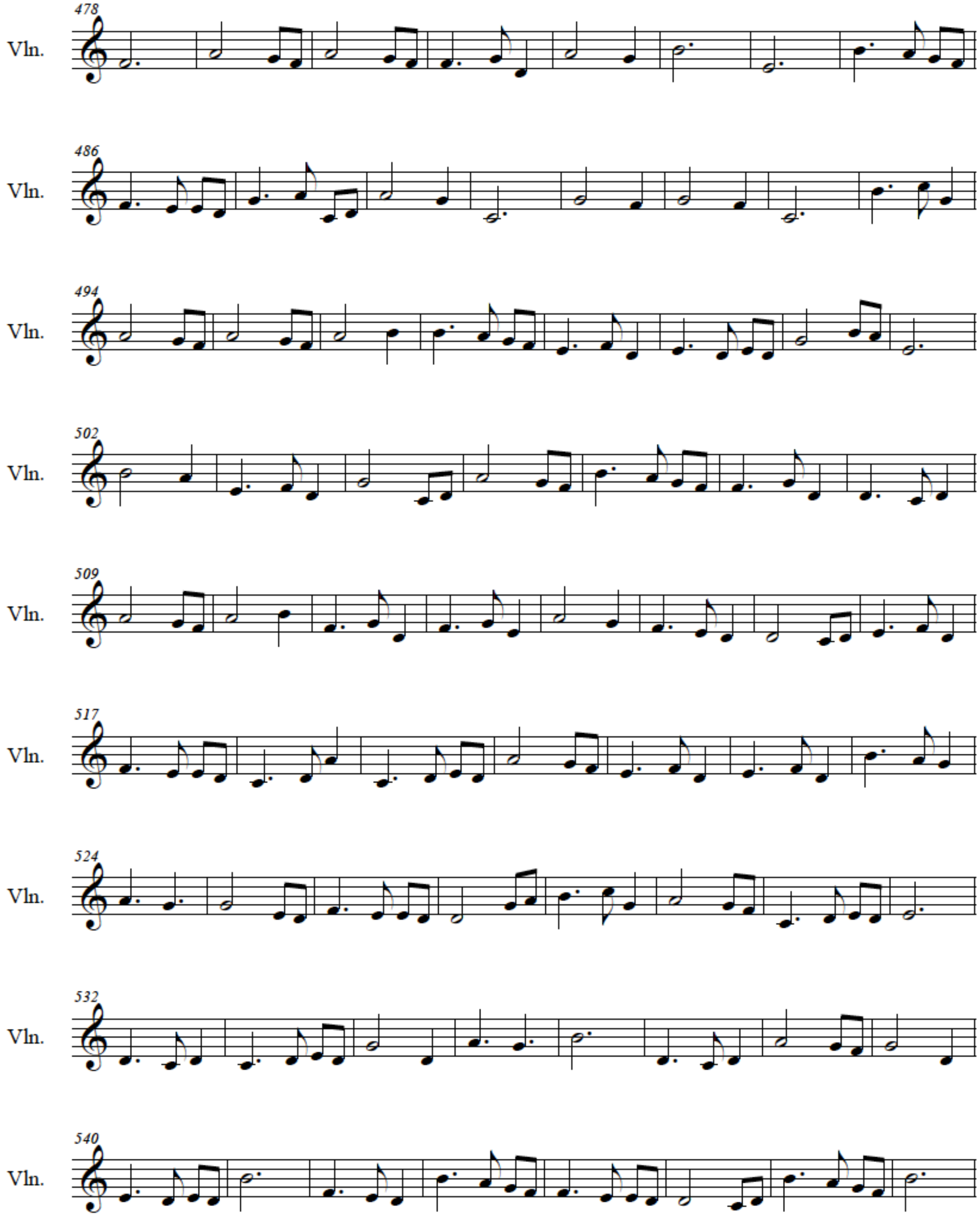
478
Vln.
486
Vln.
494
Vln.
502
Vln.
509
Vln.
517
Vln.
524
Vln.
532
Vln.
540
Vln.

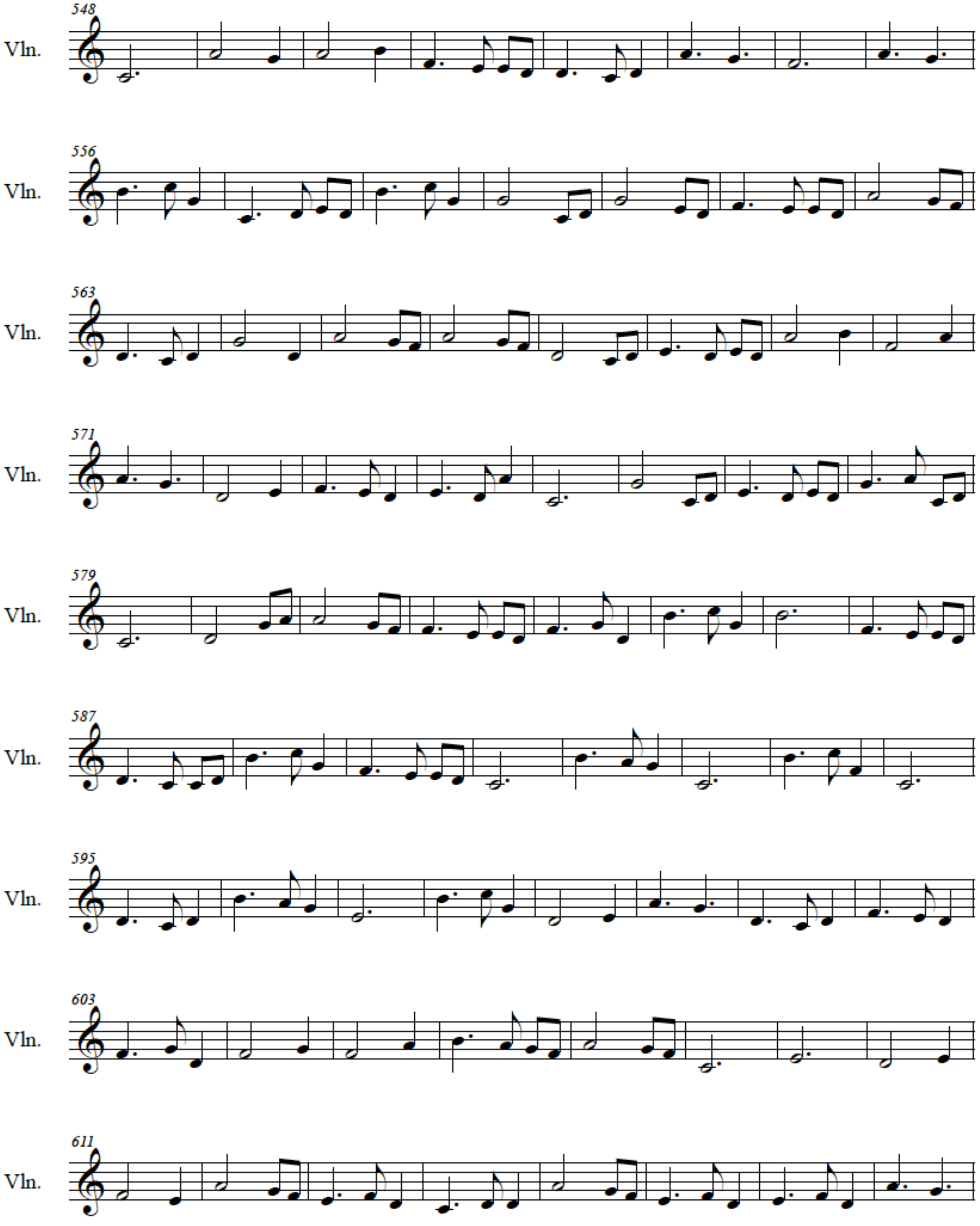
548
Vln.
556
Vln.
563
Vln.
571
Vln.
579
Vln.
587
Vln.
595
Vln.
603
Vln.
611
Vln.

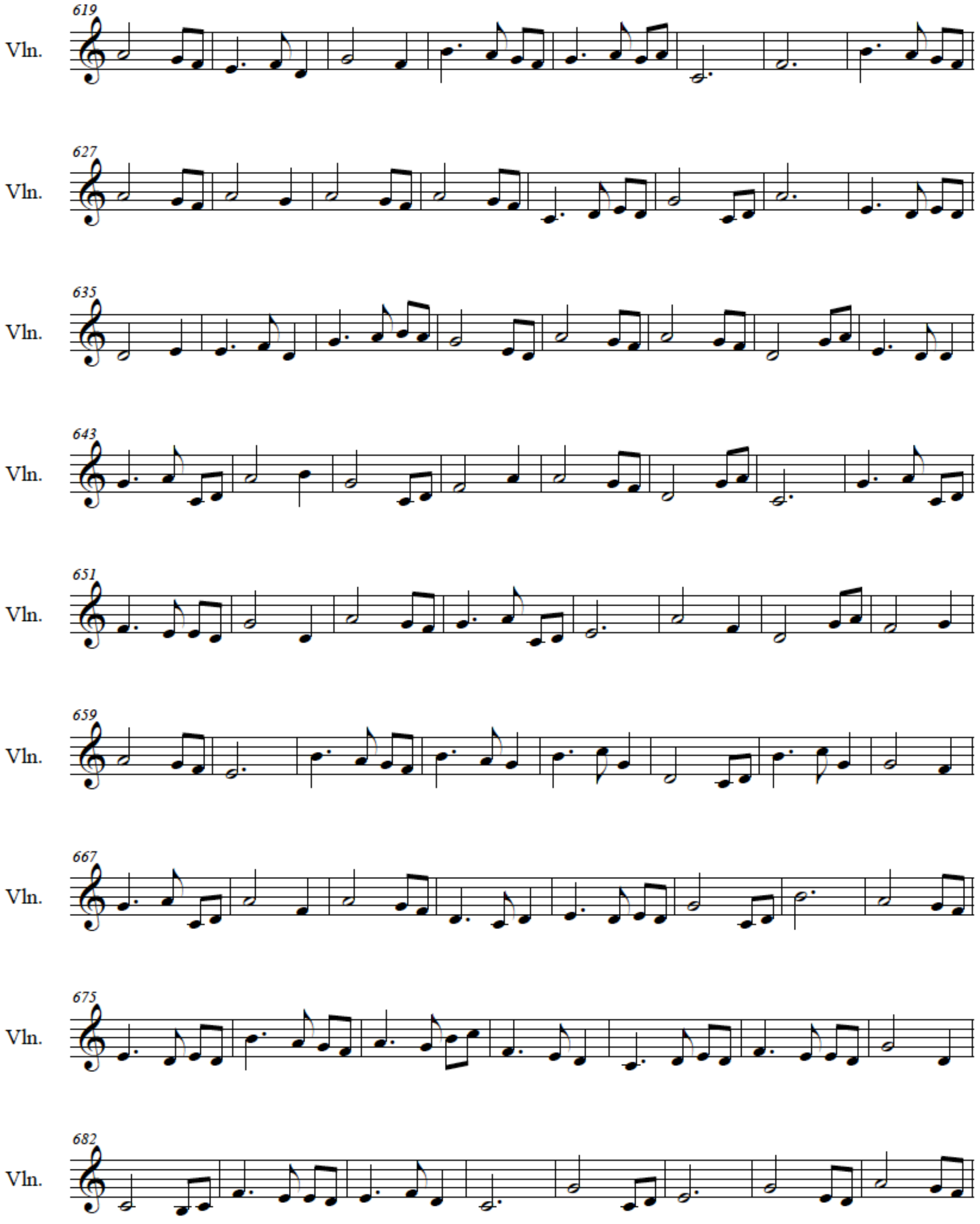
619
Vln.
627
Vln.
635
Vln.
643
Vln.
651
Vln.
659
Vln.
667
Vln.
675
Vln.
682
Vln.

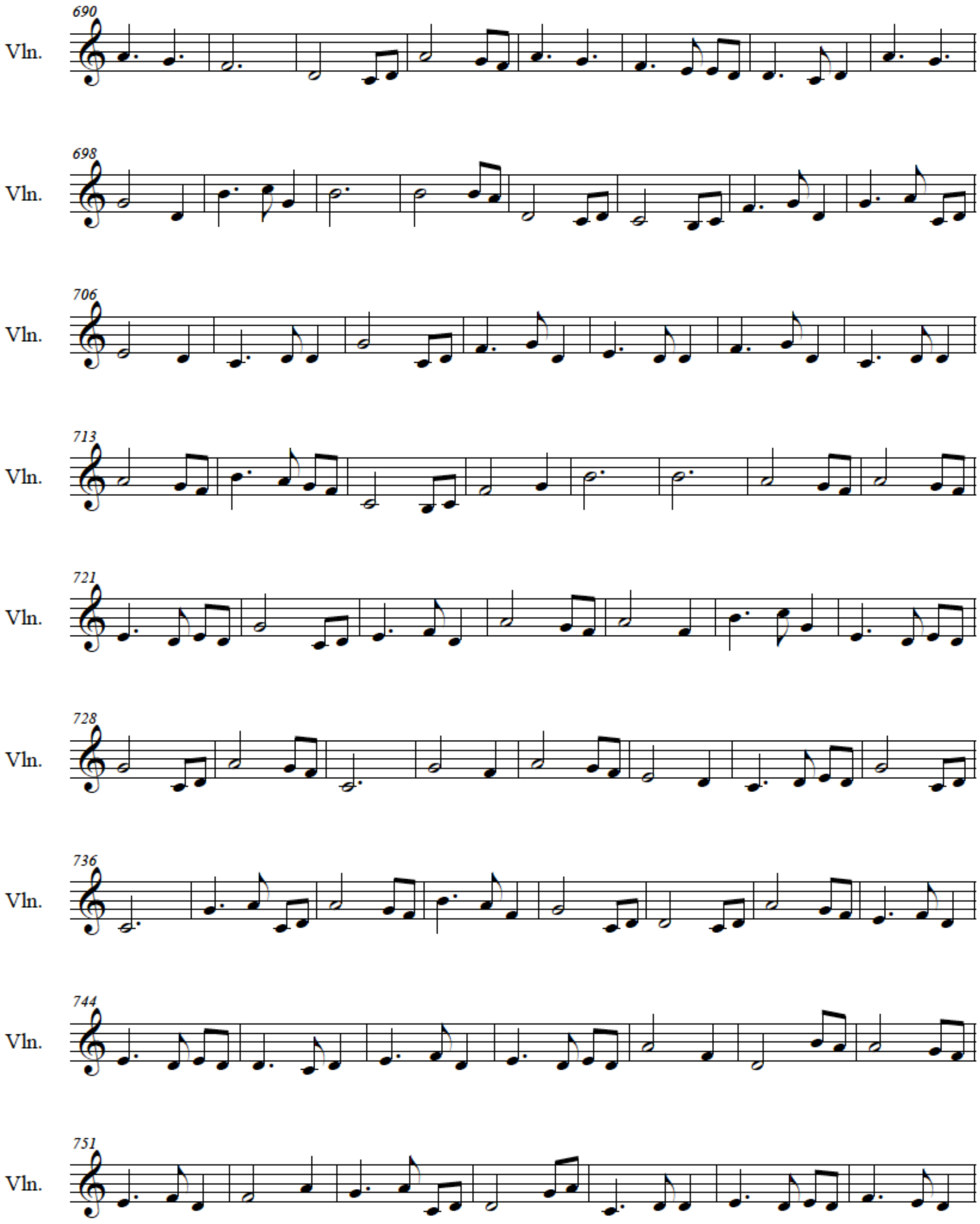
690
Vln.
698
Vln.
706
Vln.
713
Vln.
721
Vln.
728
Vln.
736
Vln.
744
Vln.
751
Vln.

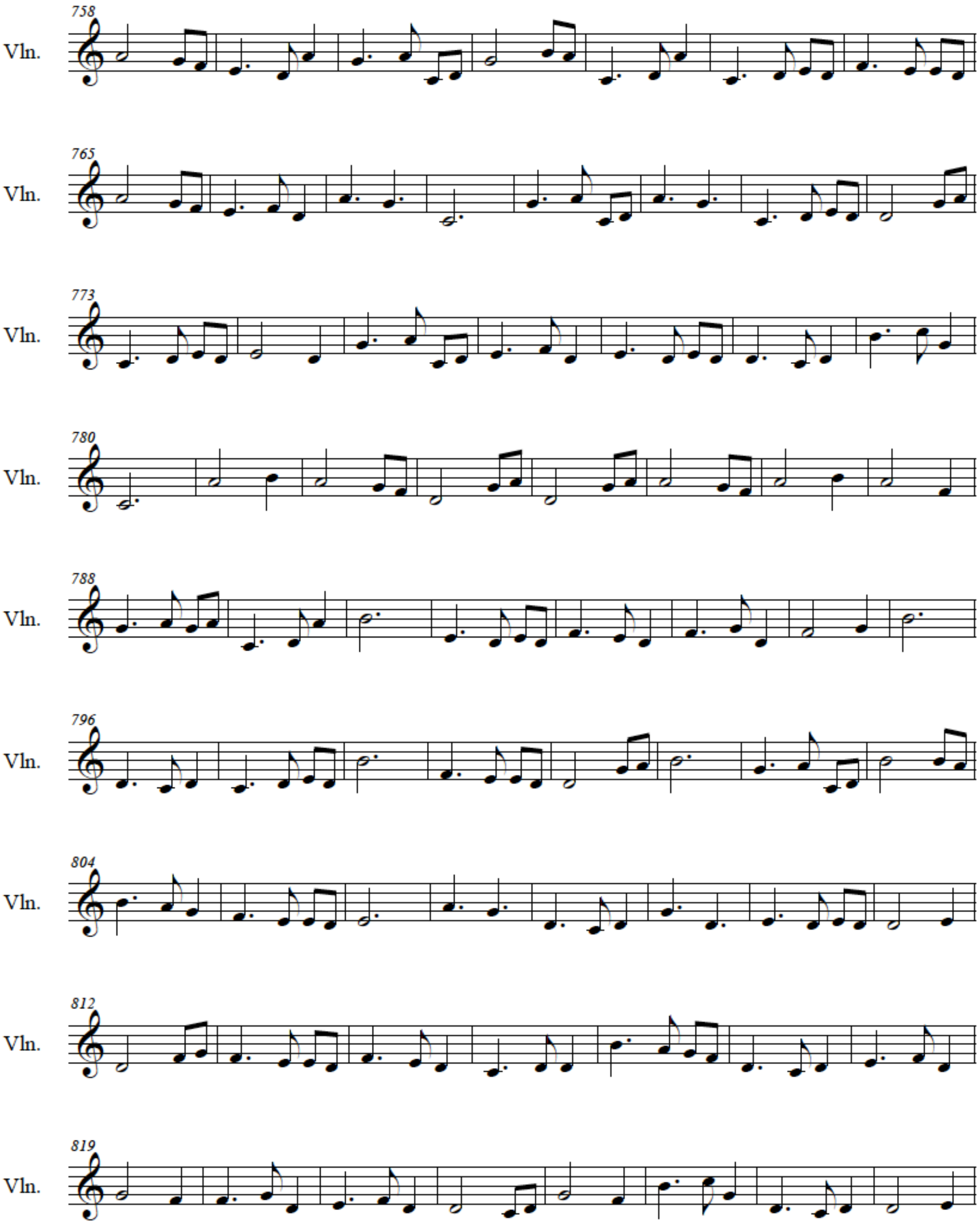
758
Vln.
765
Vln.
773
Vln.
780
Vln.
788
Vln.
796
Vln.
804
Vln.
812
Vln.
819
Vln.

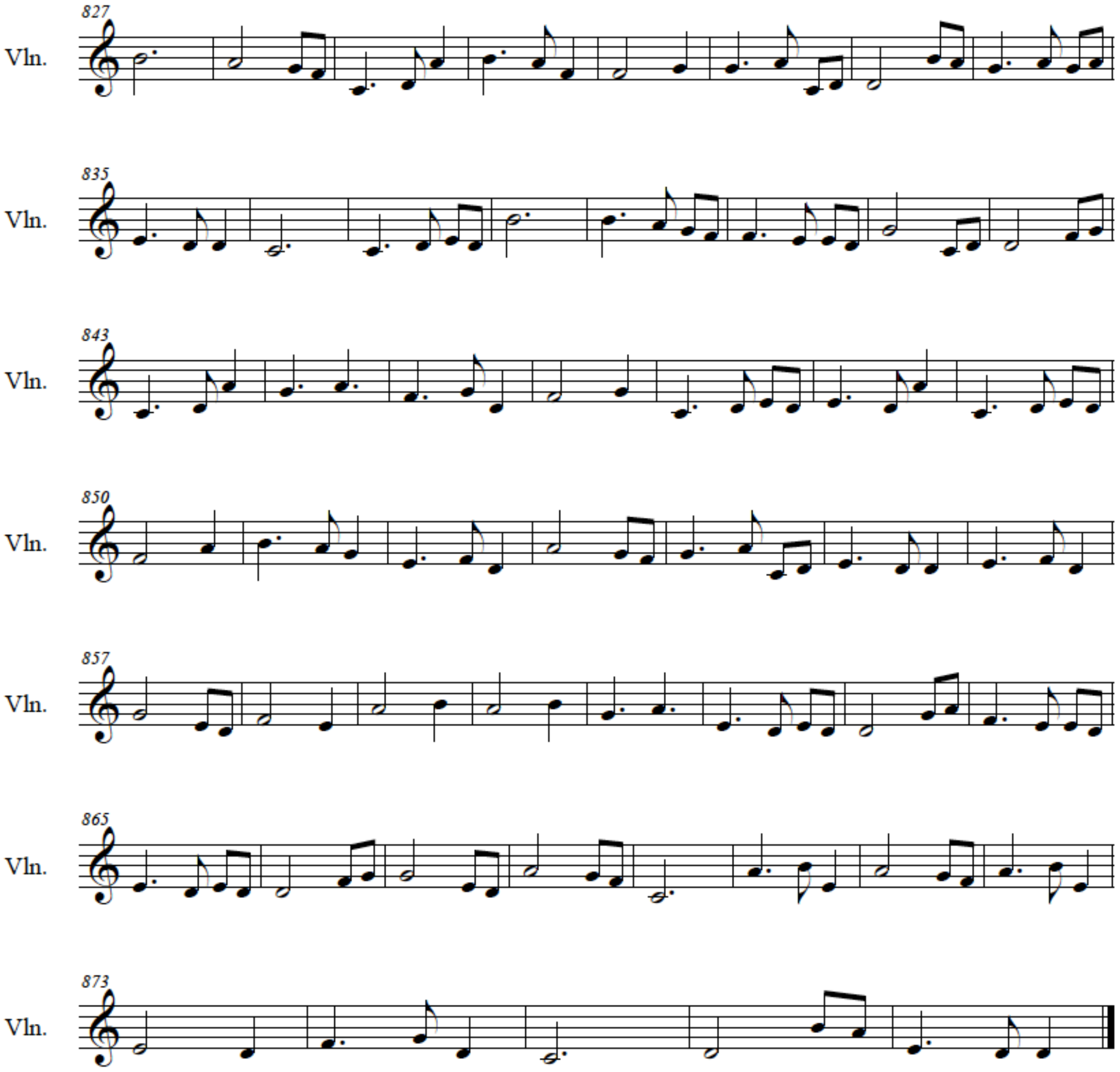
827
Vln.
835
Vln.
843
Vln.
850
Vln.
857
Vln.
865
Vln.
873
Vln.

>V00537 Homo sapiens Interferon-alpha1

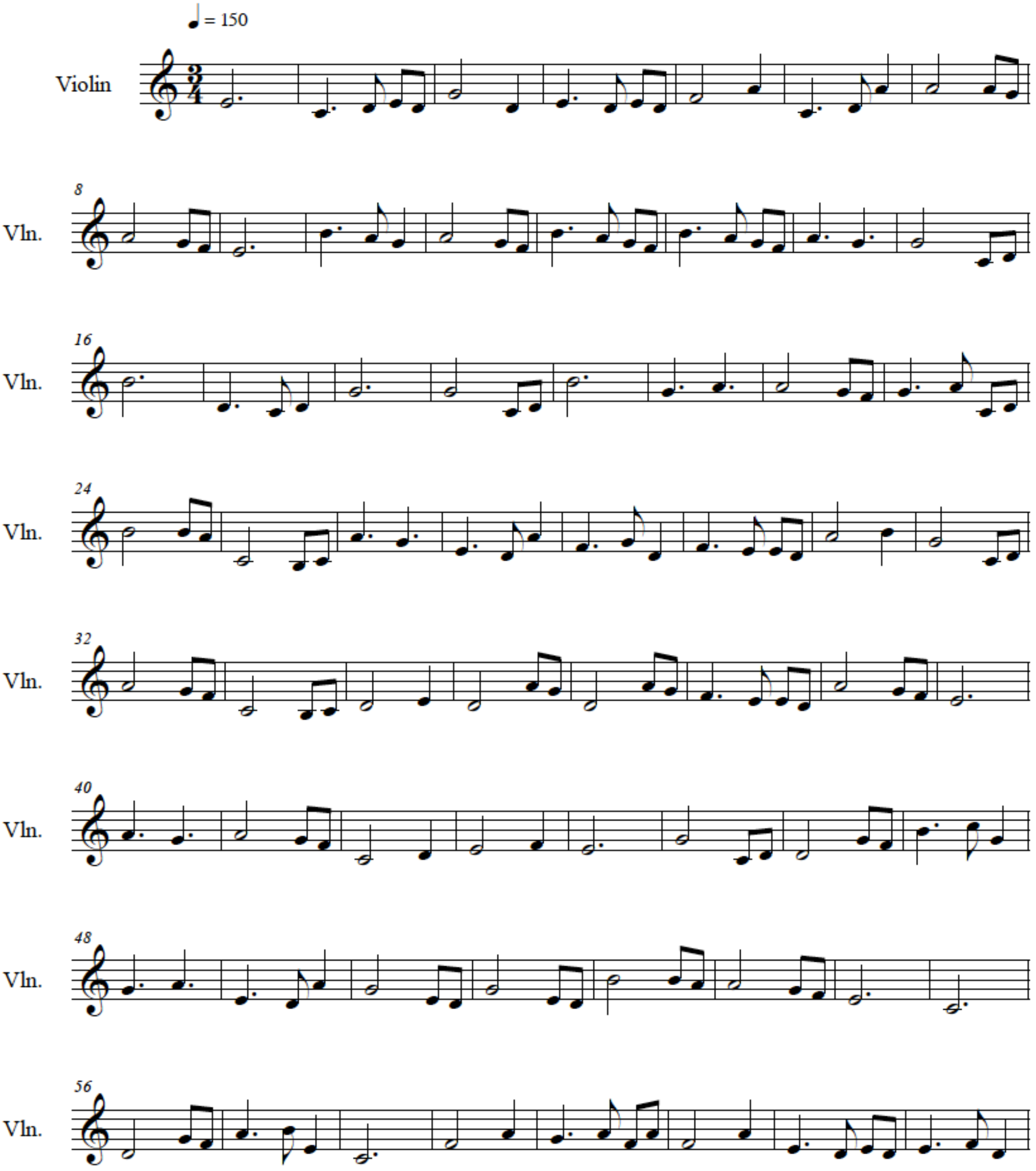

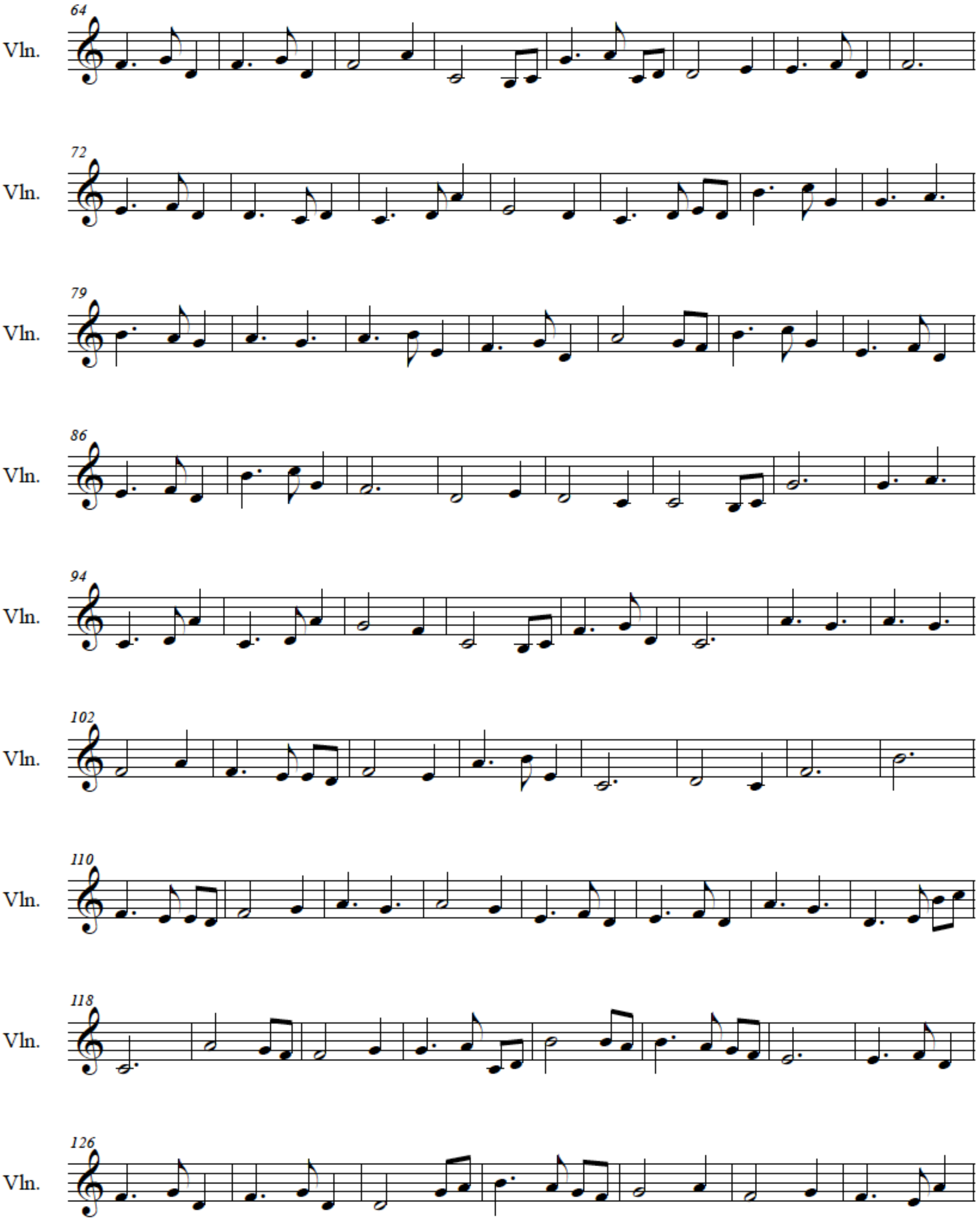
64
Vln.
72
Vln.
79
Vln.
86
Vln.
94
Vln.
102
Vln.
110
Vln.
118
Vln.
126
Vln.

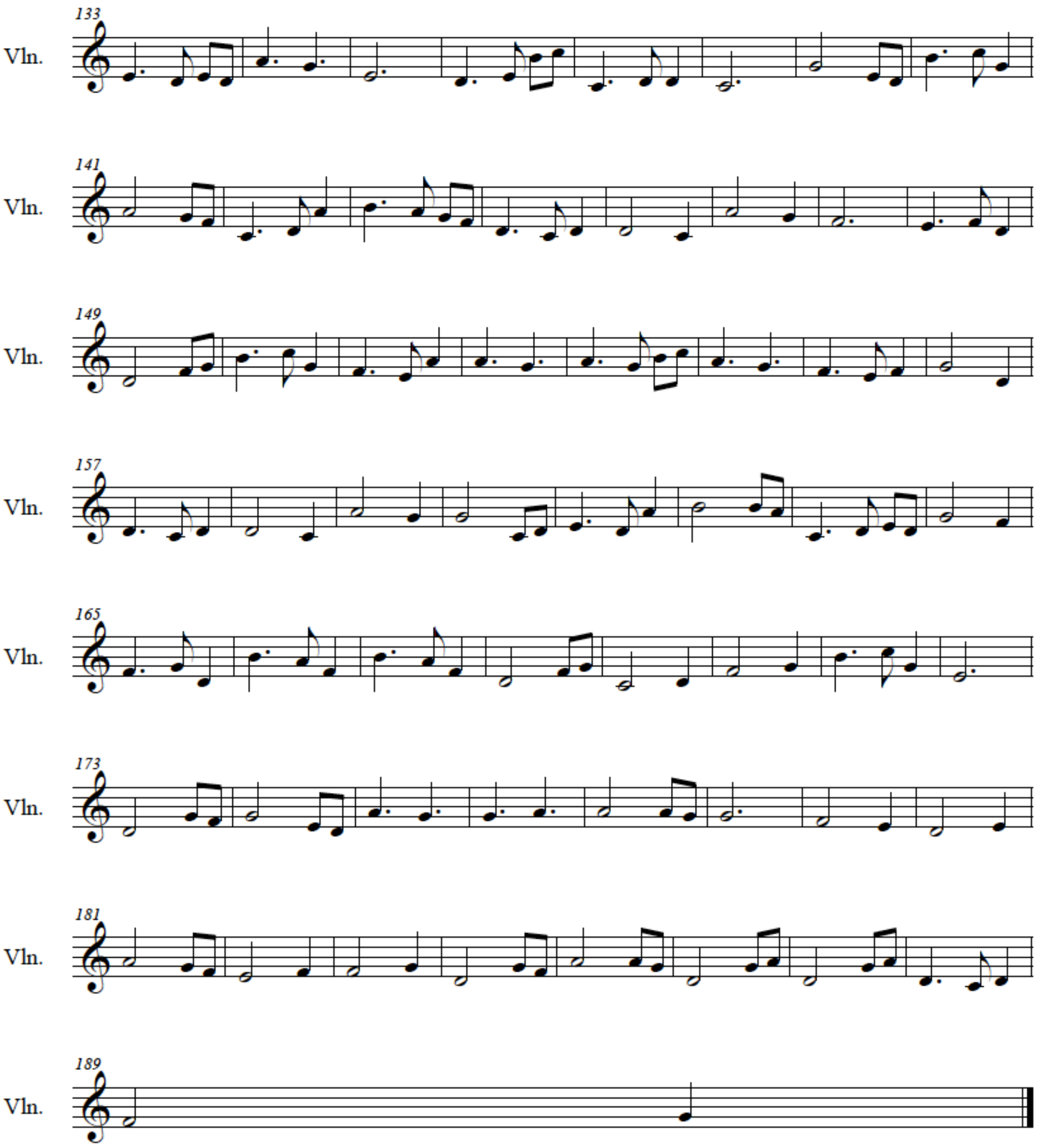

Fig. S2. Translation of AKR and selected interacting protein sequences into musical scores. The algorithm used for translation was previously reported (*8*), which also contains translation data for human IFN-a1. Modifications for this algorithm are described in Materials and Methods.

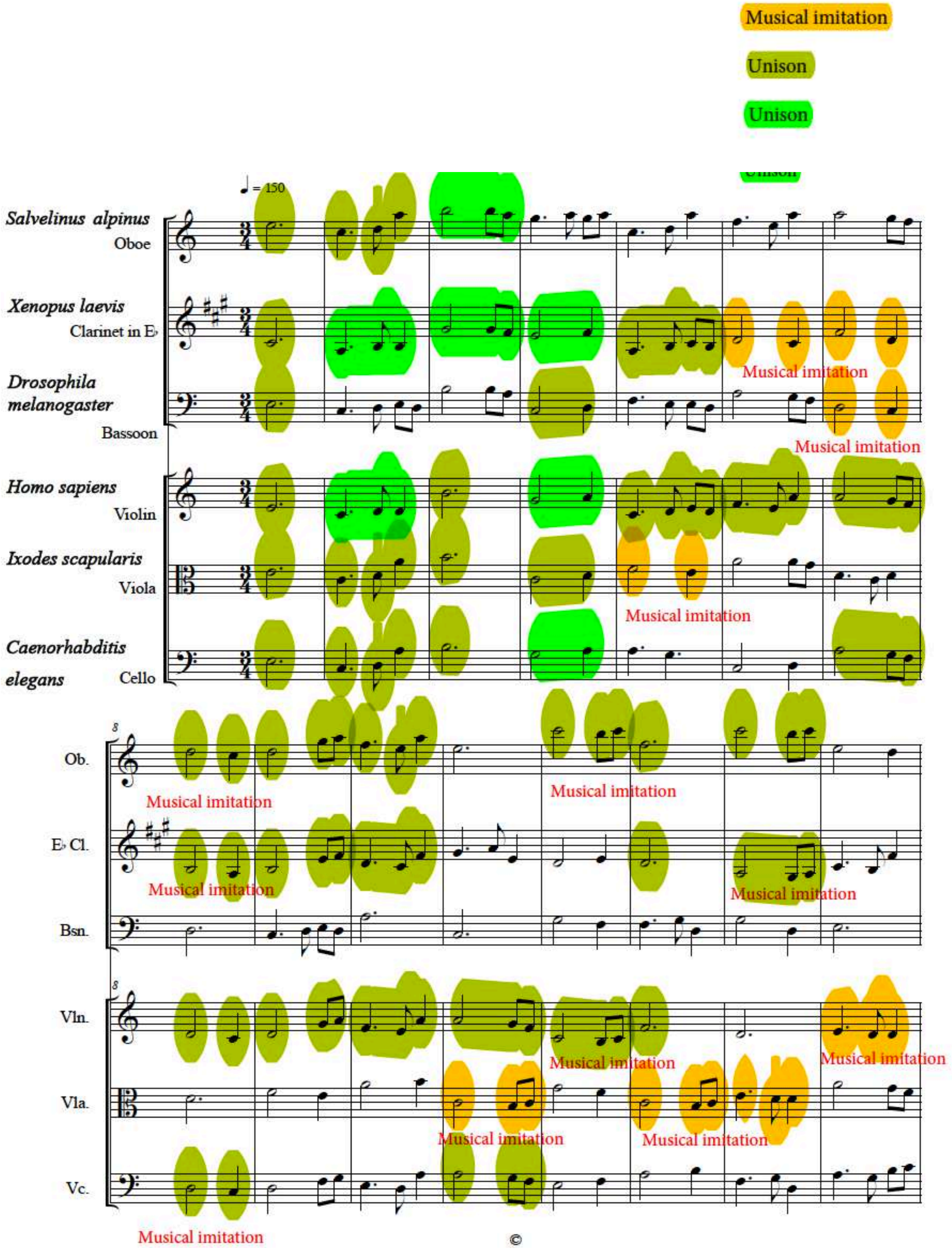
Musical imitation
Unison
Unison
♩ = 150
Salvelinus alpinus
Oboe
Xenopus laevis
Clarinet in E♭
Drosophila melanogaster
Bassoon
Homo sapiens
Violin
Ixodes scapularis
Viola
Caenorhabditis elegans
Cello
Ob.
E♭ Cl.
Bsn.
Vln.
Vla.
Vc.
Musical imitation
©

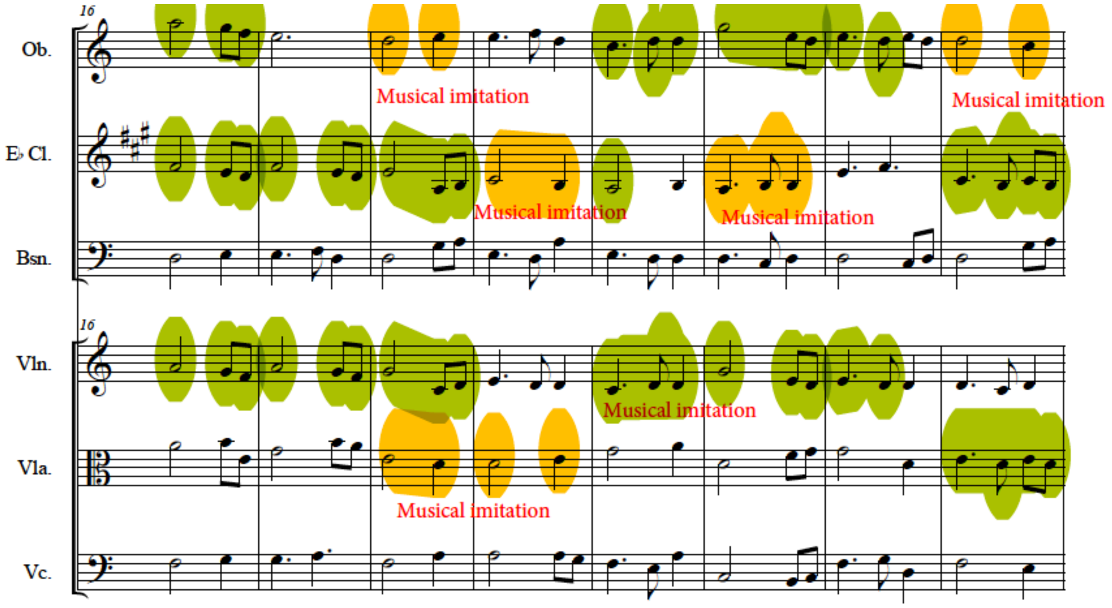
16
Ob.
E♭ Cl.
Bsn.
16
Vln.
Vla.
Vc.
Musical imitation
Musical imitation
Musical imitation
Musical imitation
Musical imitation
Musical imitation

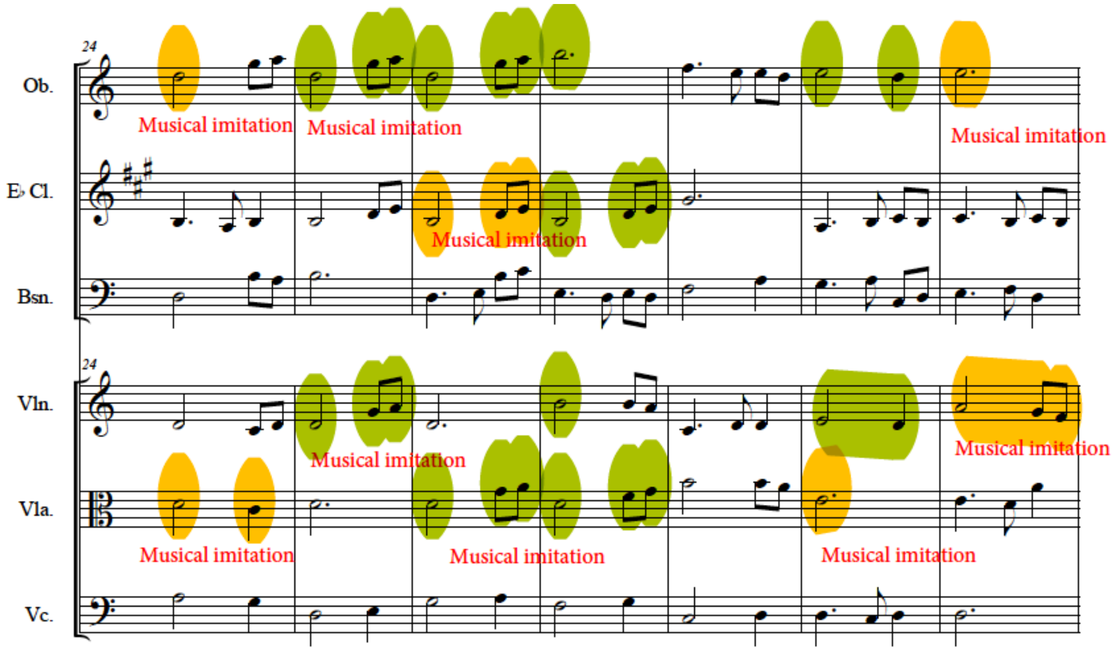
24
Ob.
E♭ Cl.
Bsn.
24
Vln.
Vla.
Vc.
Musical imitation
Musical imitation
Musical imitation
Musical imitation
Musical imitation
Musical imitation
Musical imitation
Musical imitation

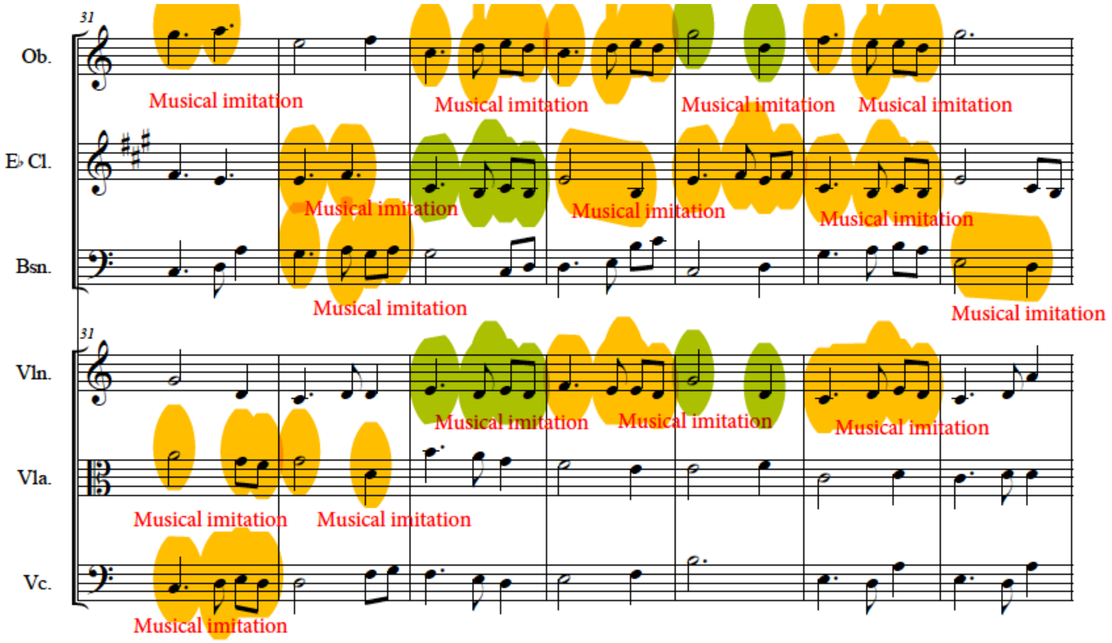
31
Ob.
Musical imitation
Musical imitation
Musical imitation
Musical imitation
E♭ Cl.
Musical imitation
Musical imitation
Musical imitation
Bsn.
Musical imitation
Musical imitation
31
Vln.
Musical imitation
Musical imitation
Musical imitation
Vla.
Musical imitation
Musical imitation
Vc.
Musical imitation

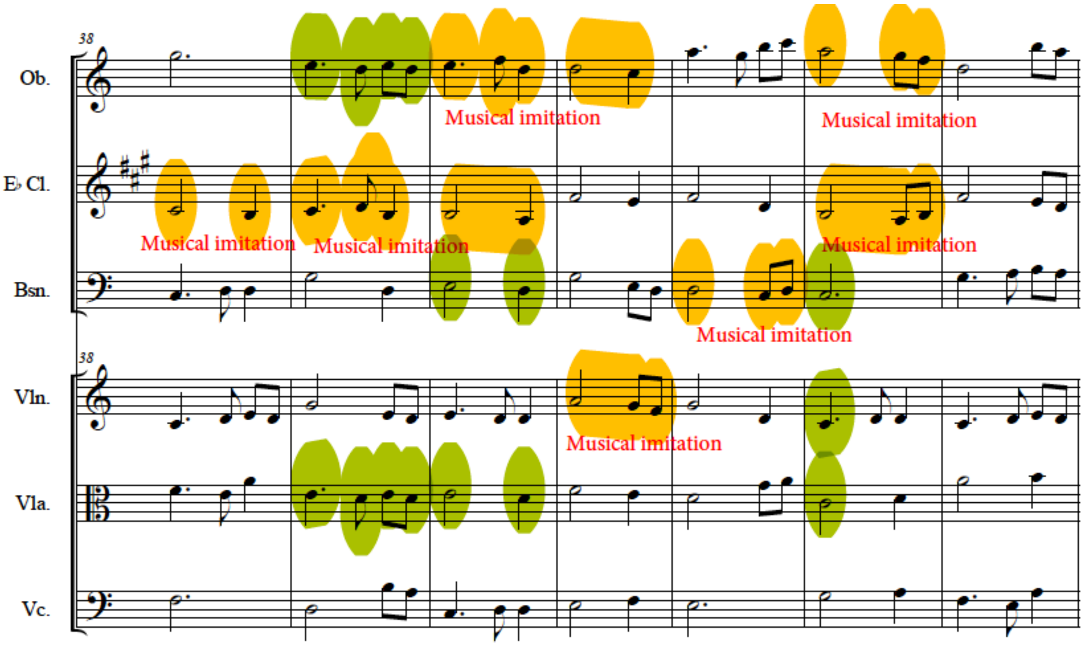
38
Ob.
Musical imitation
Musical imitation
E♭ Cl.
Musical imitation
Musical imitation
Musical imitation
Bsn.
Musical imitation
38
Vln.
Musical imitation
Vla.
Vc.

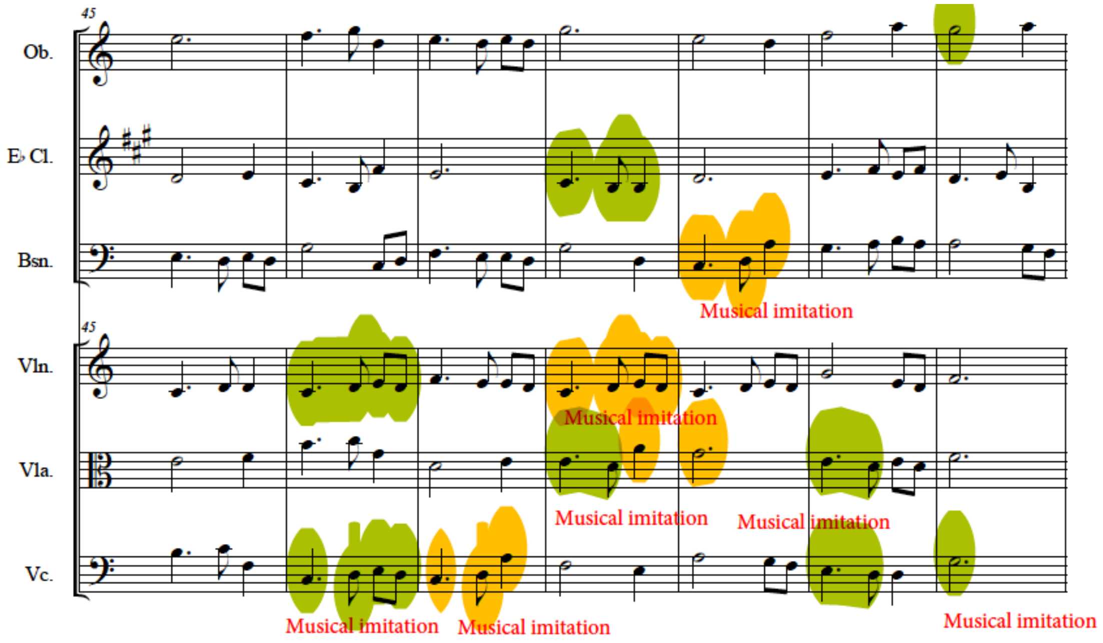
45
Ob.
E♭ Cl.
Bsn.
Musical imitation
45
Vln.
Musical imitation
Vla.
Musical imitation
Musical imitation
Vc.
Musical imitation
Musical imitation
Musical imitation

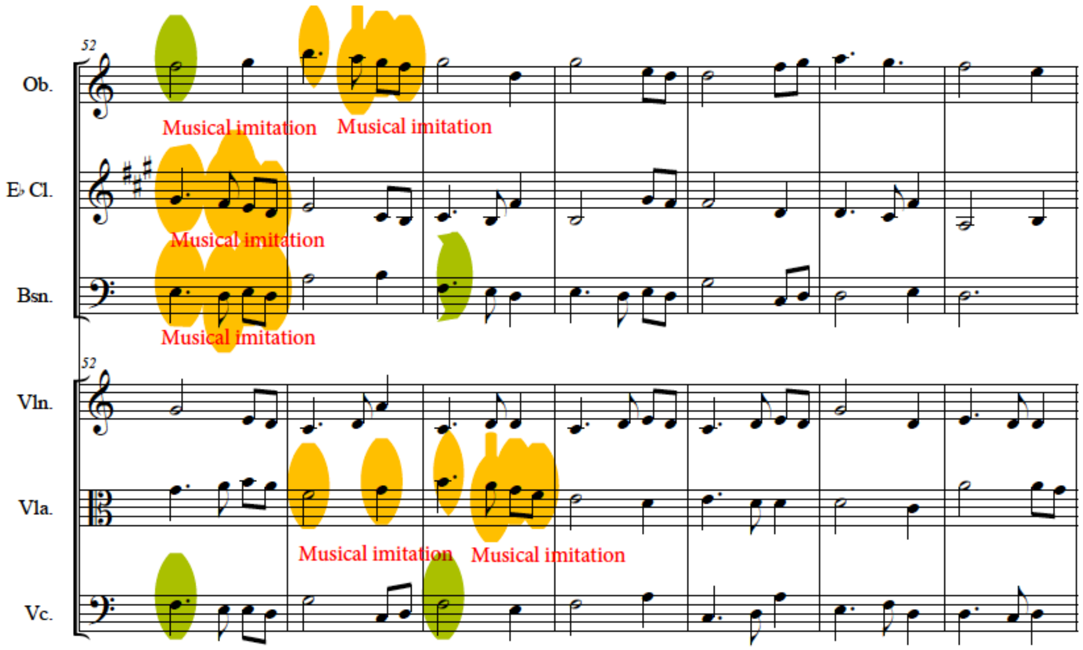
52
Ob.
Musical imitation
Musical imitation
E♭ Cl.
Musical imitation
Bsn.
Musical imitation
52
Vln.
Vla.
Musical imitation
Musical imitation
Vc.

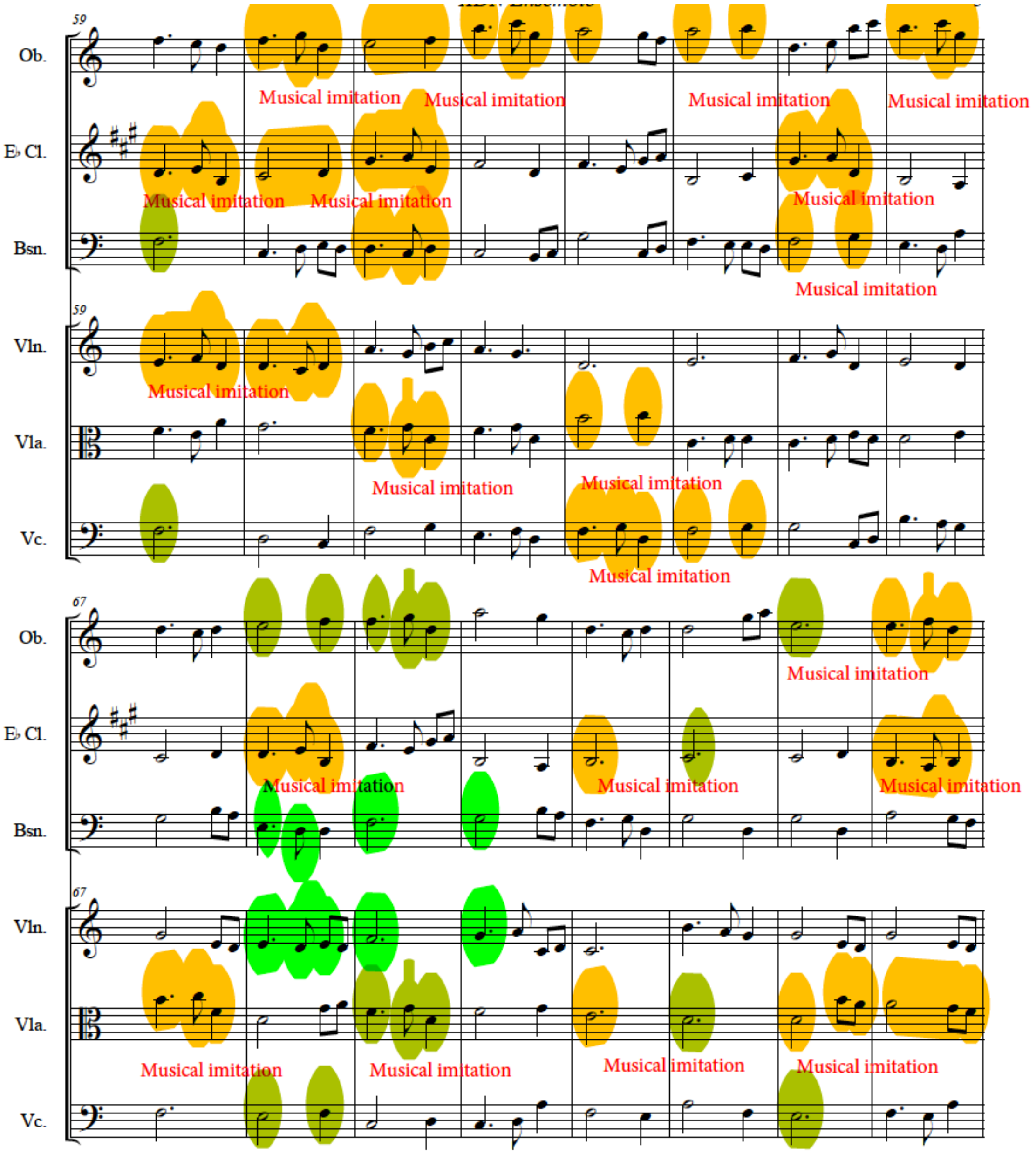
59
Ob.
Musical imitation
Musical imitation
Musical imitation
Musical imitation
E♭ Cl.
Musical imitation
Musical imitation
Musical imitation
Bsn.
Musical imitation
59
Vln.
Musical imitation
Vla.
Musical imitation
Musical imitation
Vc.
Musical imitation
67
Ob.
Musical imitation
E♭ Cl.
Musical imitation
Musical imitation
Musical imitation
Bsn.
67
Vln.
Vla.
Musical imitation
Musical imitation
Musical imitation
Musical imitation
Vc.

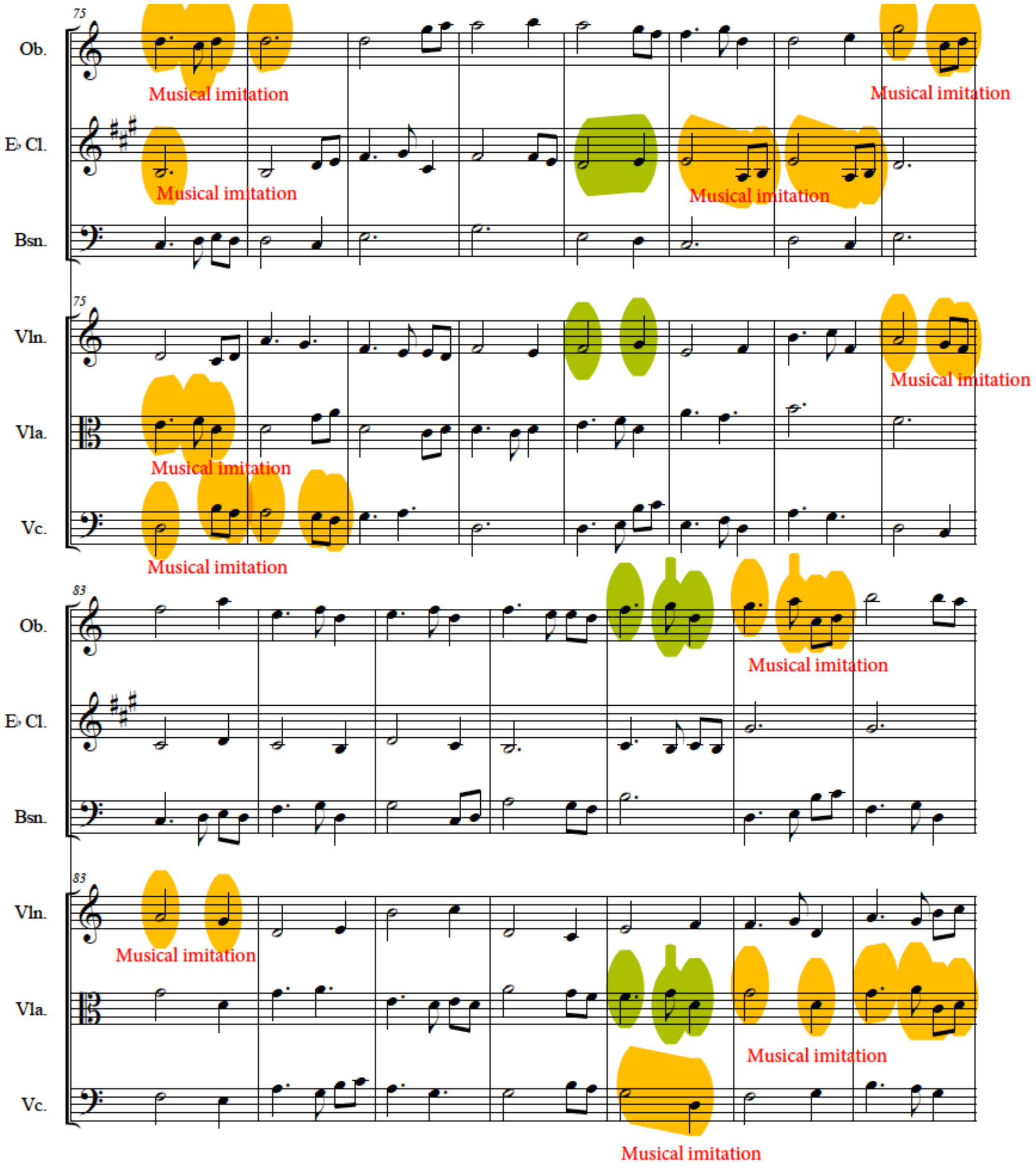
75
Ob.
Musical imitation
Musical imitation
E♭ Cl.
Musical imitation
Musical imitation
Bsn.
75
Vln.
Musical imitation
Vla.
Musical imitation
Vc.
Musical imitation
83
Ob.
Musical imitation
E♭ Cl.
Bsn.
83
Vln.
Musical imitation
Vla.
Musical imitation
Vc.
Musical imitation

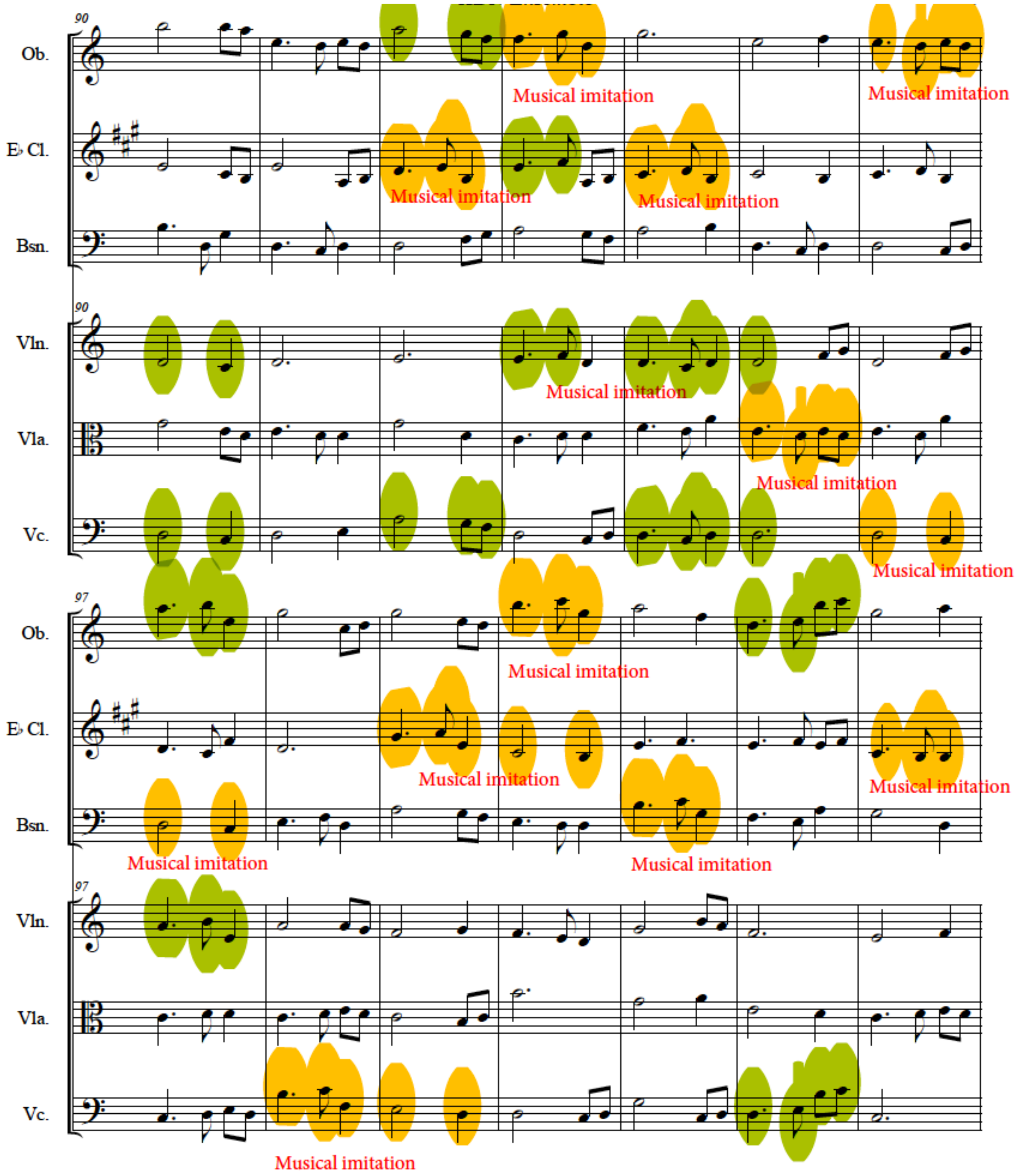
90
Ob.
E♭ Cl.
Bsn.
Vln.
Vla.
Vc.
Musical imitation
Musical imitation
Musical imitation
Musical imitation
Musical imitation
Musical imitation
Musical imitation
97
Musical imitation
Musical imitation
Musical imitation
Musical imitation
Musical imitation
Musical imitation

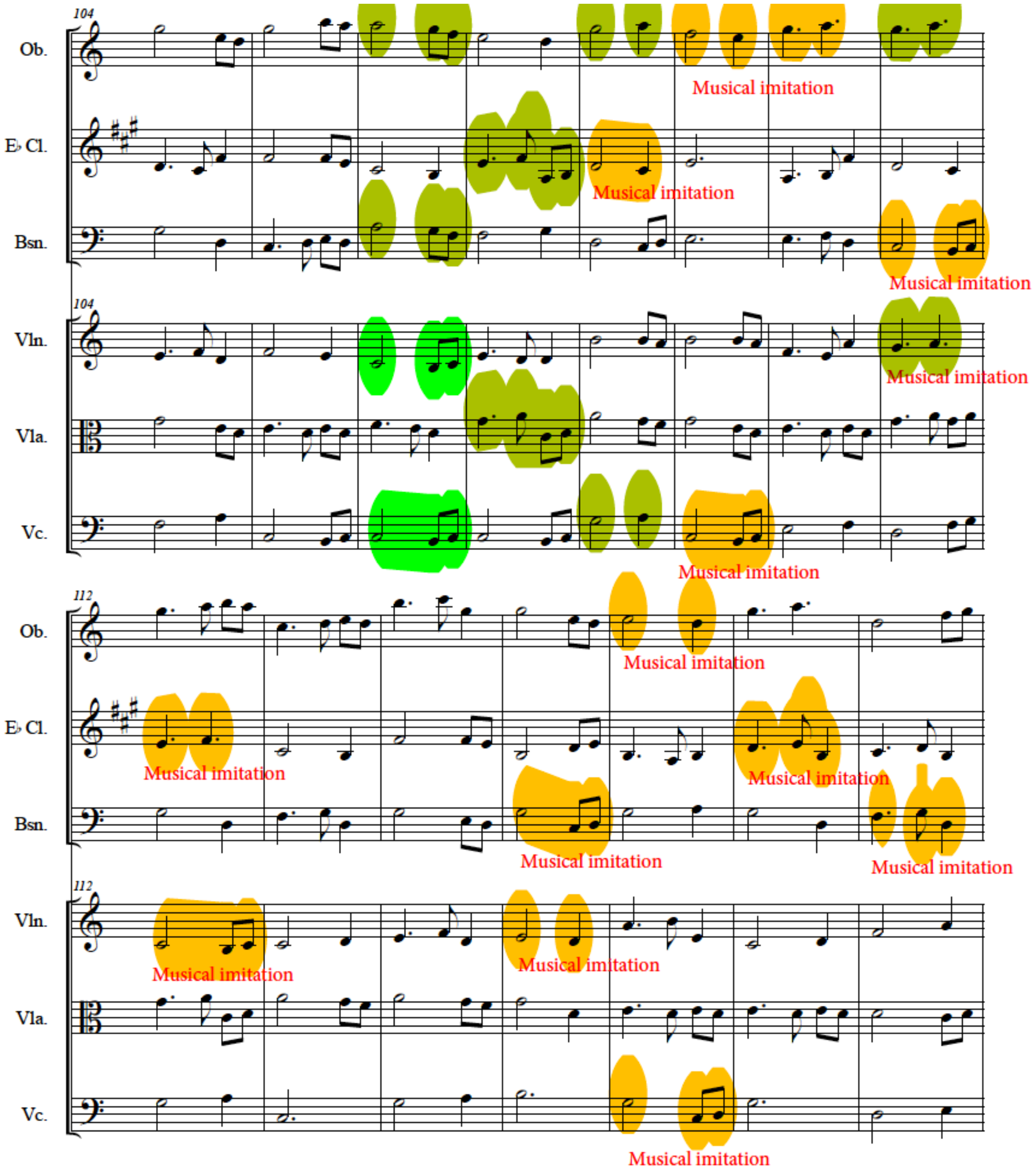
Ob.
E♭ Cl.
Bsn.
Vln.
Vla.
Vc.
104
112
Musical imitation

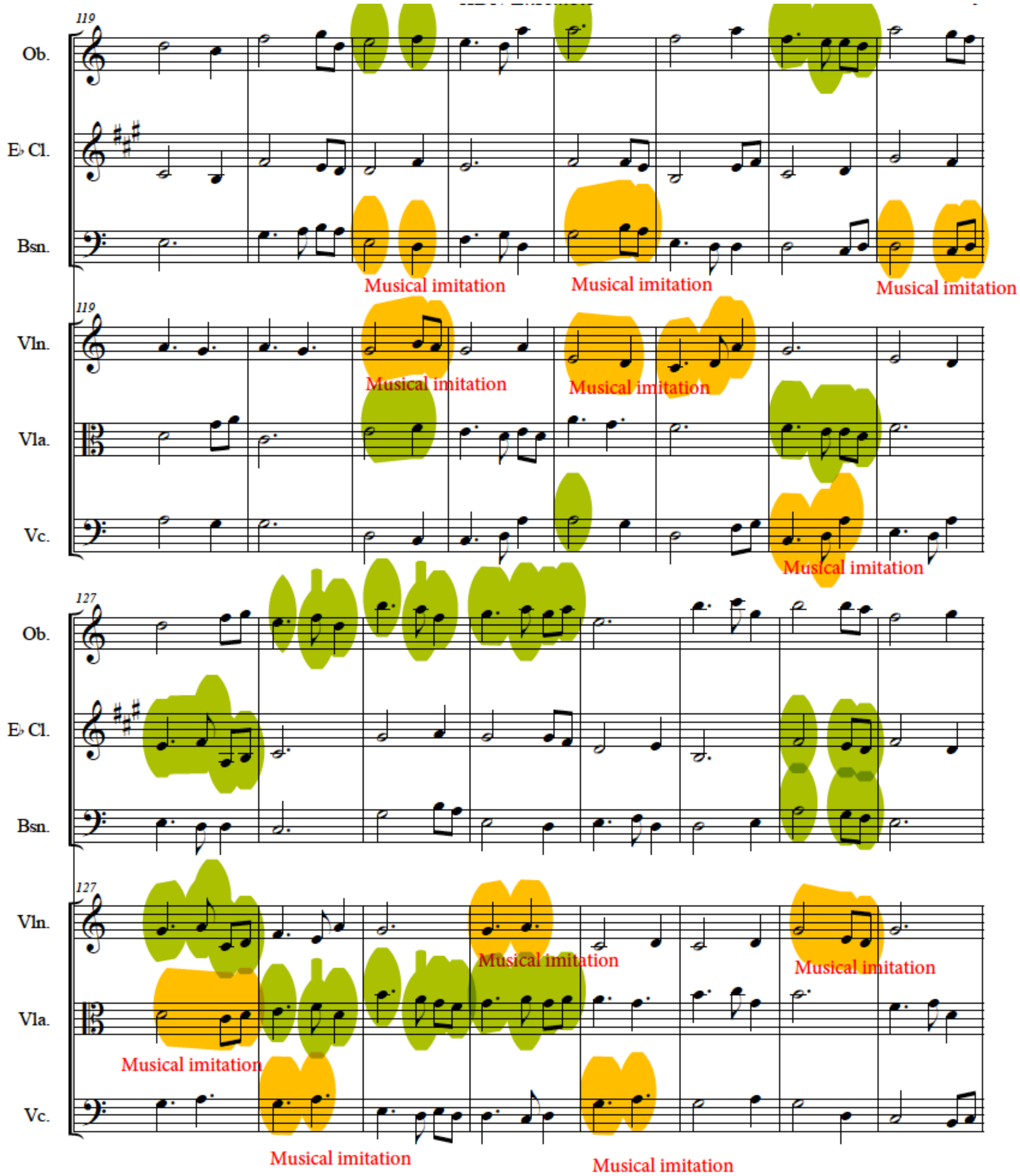
119
Ob.
E♭ Cl.
Bsn.
Musical imitation
Musical imitation
Musical imitation
119
Vln.
Musical imitation
Musical imitation
Vla.
Vc.
Musical imitation
127
Ob.
E♭ Cl.
Bsn.
127
Vln.
Musical imitation
Musical imitation
Vla.
Musical imitation
Vc.
Musical imitation
Musical imitation

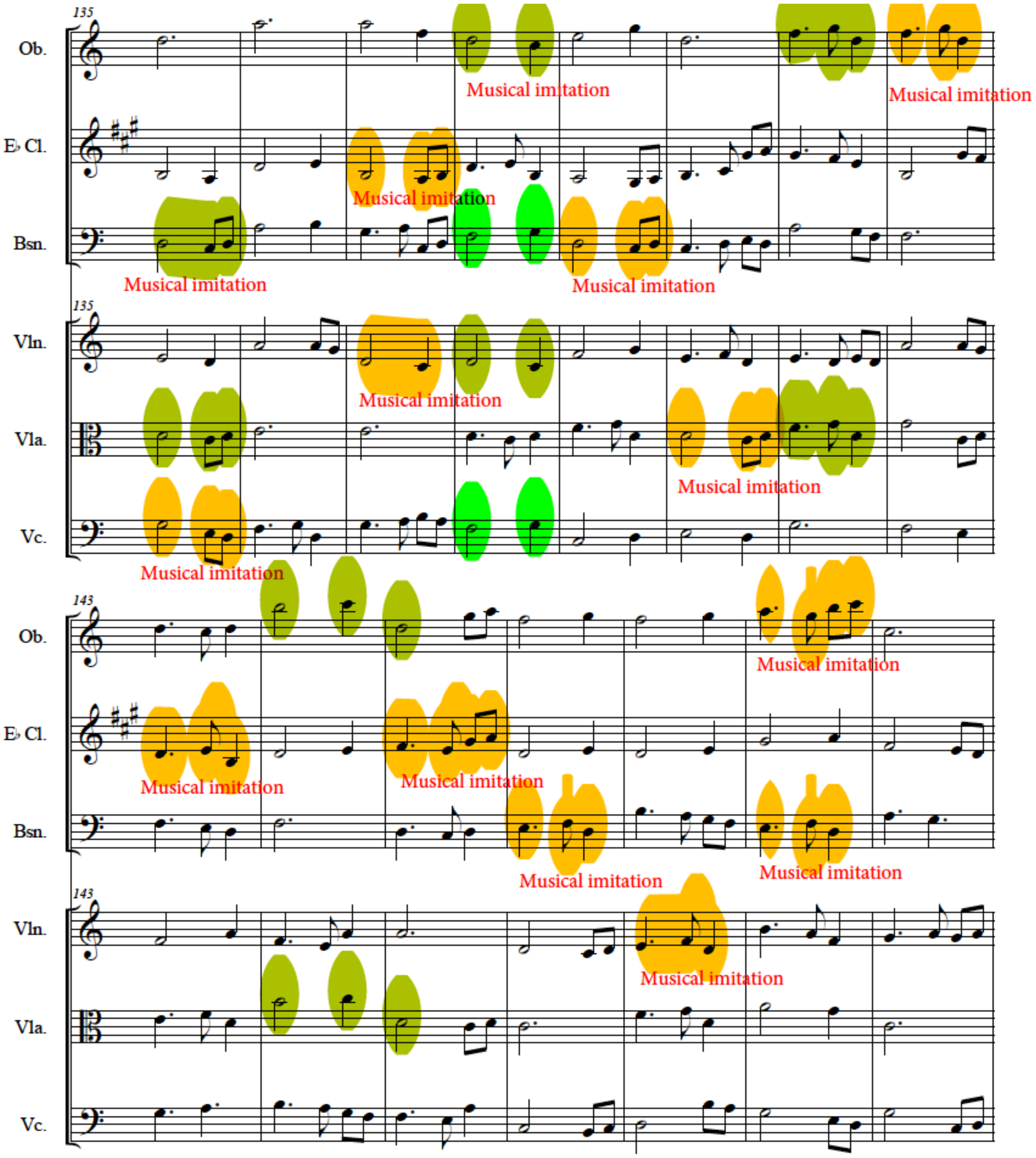
135
Ob.
Musical imitation
Musical imitation
E♭ Cl.
Musical imitation
Bsn.
Musical imitation
Musical imitation
135
Vln.
Musical imitation
Vla.
Musical imitation
Vc.
Musical imitation
143
Ob.
Musical imitation
E♭ Cl.
Musical imitation
Musical imitation
Bsn.
Musical imitation
Musical imitation
143
Vln.
Musical imitation
Vla.
Vc.

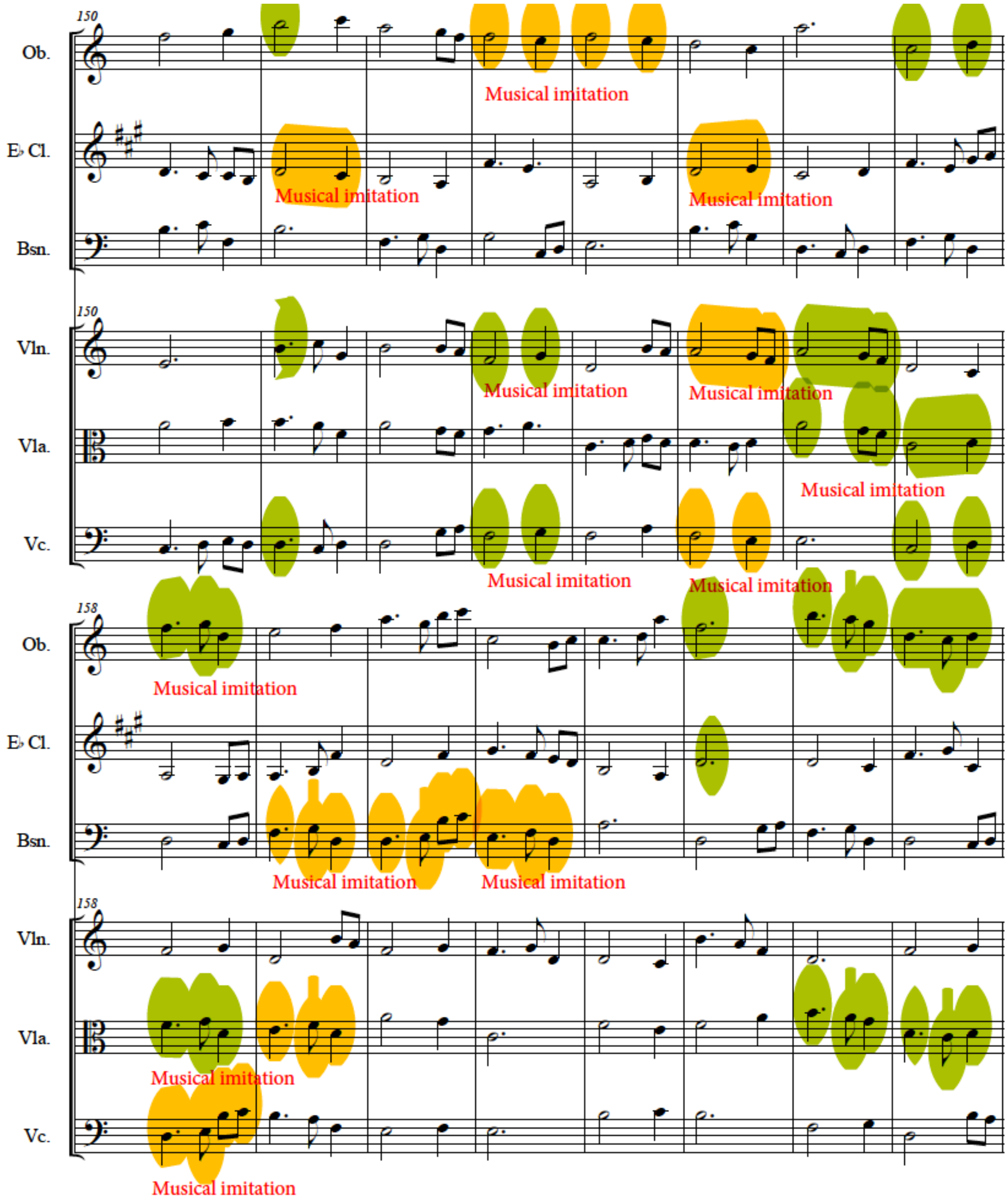
150
Ob.
Musical imitation
E♭ Cl.
Musical imitation
Musical imitation
Bsn.
150
Vln.
Musical imitation
Musical imitation
Vla.
Musical imitation
Vc.
Musical imitation
Musical imitation
158
Ob.
Musical imitation
E♭ Cl.
Bsn.
Musical imitation
Musical imitation
158
Vln.
Vla.
Musical imitation
Vc.
Musical imitation

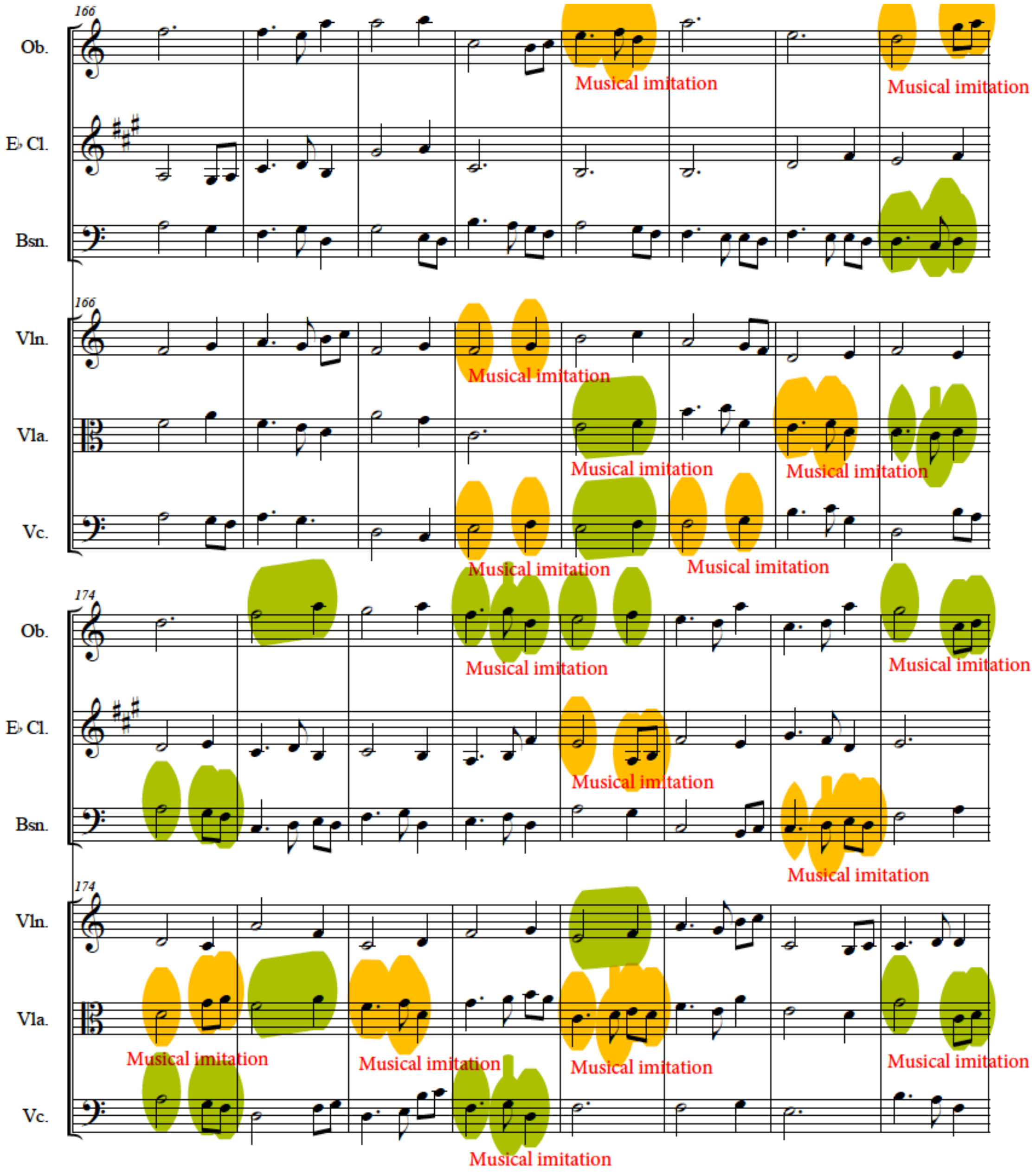
166
Ob.
E♭ Cl.
Bsn.
Vln.
Vla.
Vc.
Musical imitation
174
Ob.
E♭ Cl.
Bsn.
Vln.
Vla.
Vc.
Musical imitation

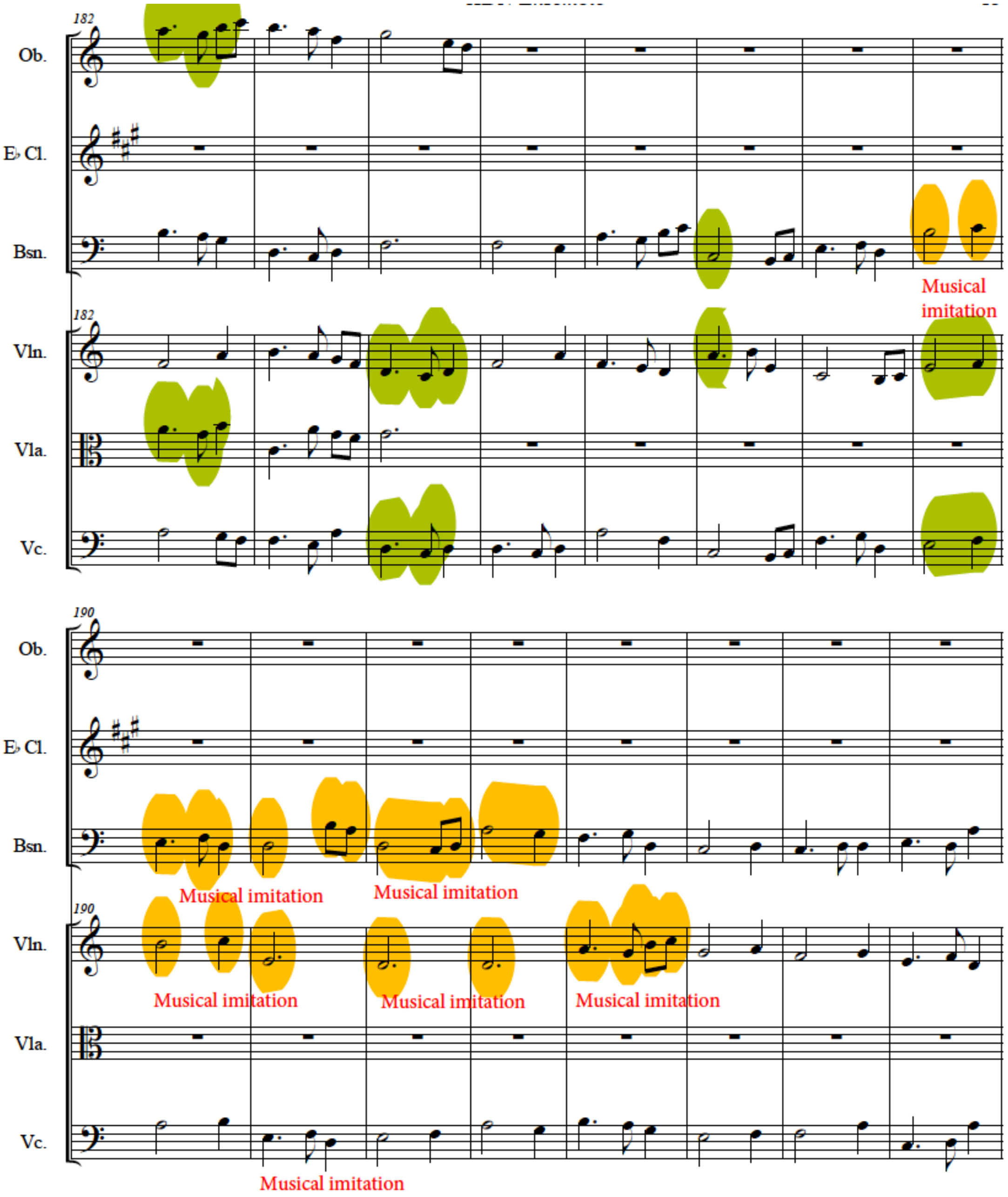
182
Ob.
E♭ Cl.
Bsn.
Musical imitation
182
Vln.
Vla.
Vc.
190
Ob.
E♭ Cl.
Bsn.
Musical imitation
Musical imitation
190
Vln.
Musical imitation
Musical imitation
Musical imitation
Vla.
Vc.
Musical imitation

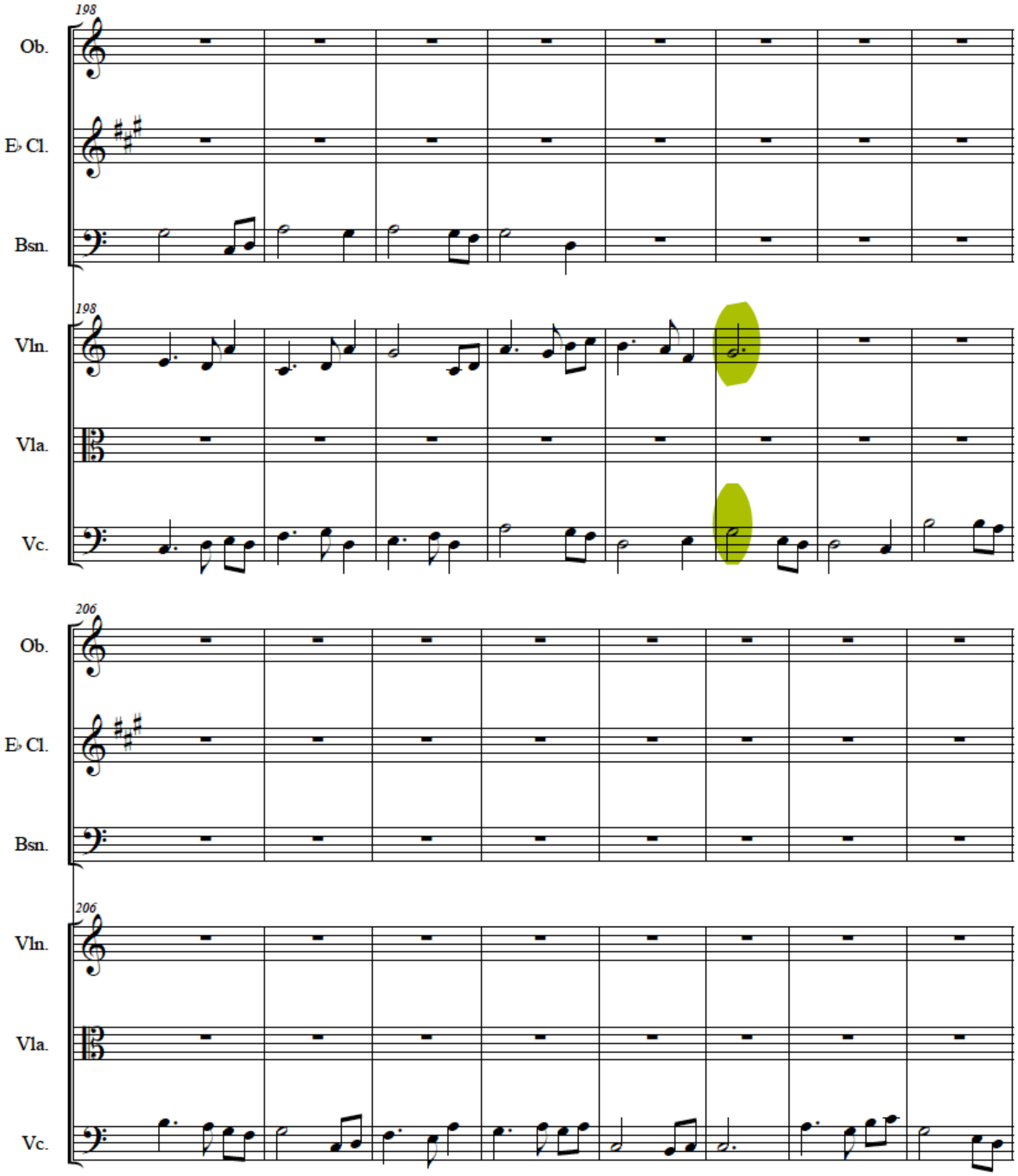
198
Ob.
E♭ Cl.
Bsn.
198
Vln.
Vla.
Vc.
206
Ob.
E♭ Cl.
Bsn.
206
Vln.
Vla.
Vc.

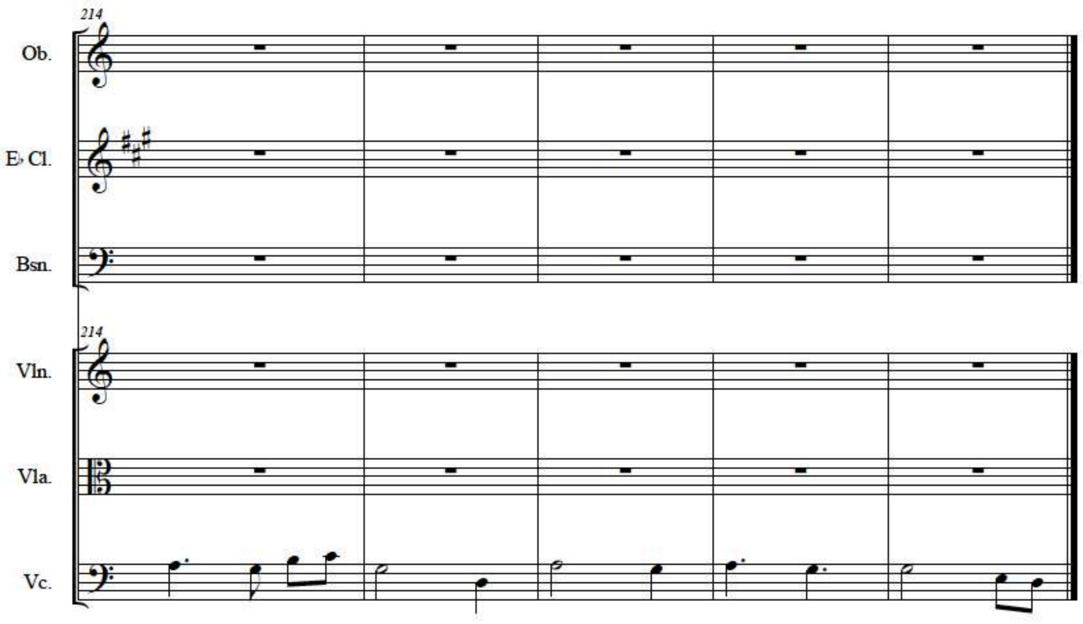

Fig. S3. Multi species AKR musical ensemble. Musical scores for *C. elegans* AKR, *D. melanogaster* AKR, *I. scapularis* AKR (SUB), *X. laevis* AKR2, *S. alpinus* AKR2 and *H. sapiens* AKR2 (Supplementary information, Fig. S1) were ensemble and analyzed for musical patterns.

Unison

Third / Sixth interval

Fourth / Fifth interval

AKR2-RNF10

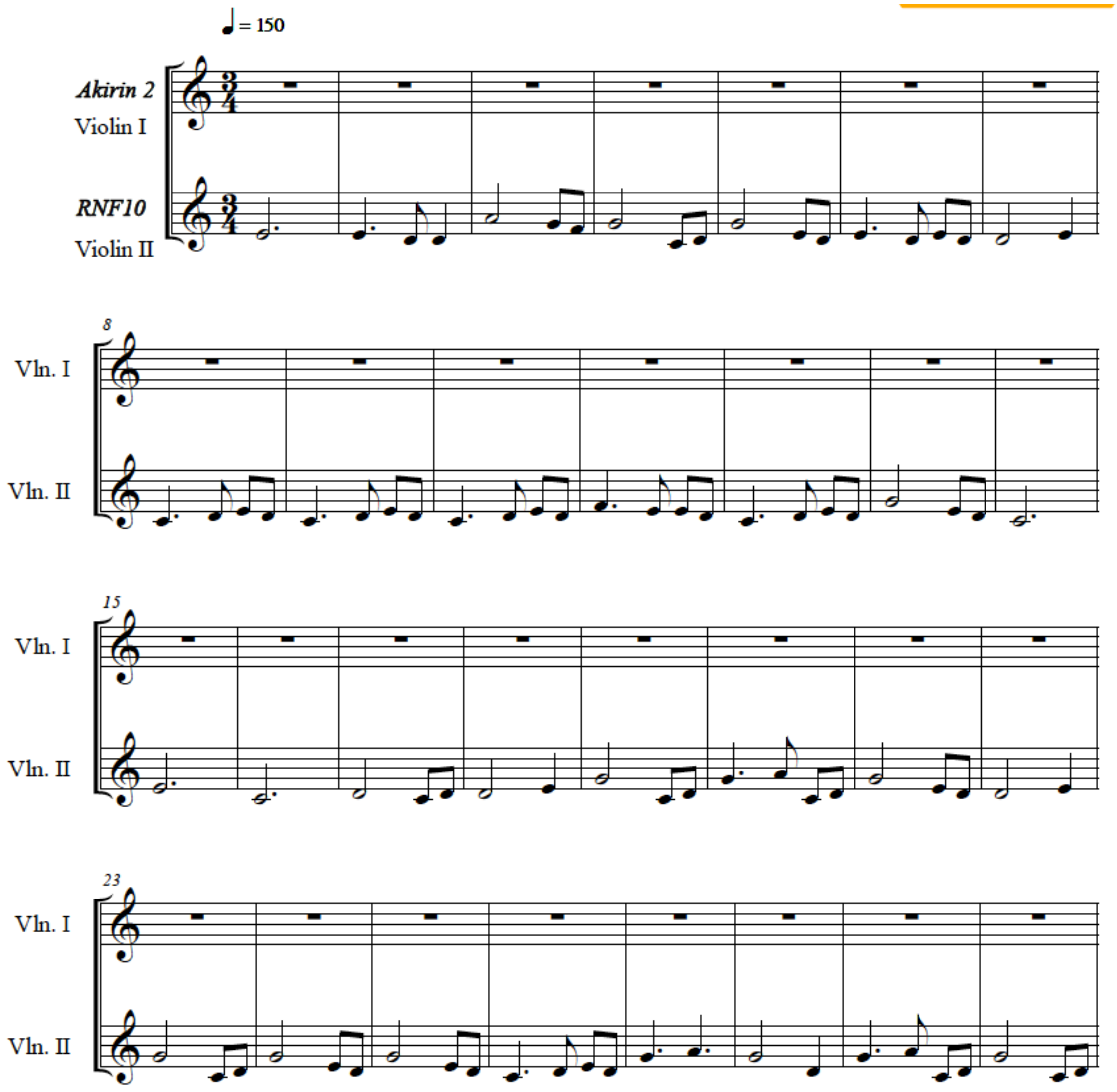

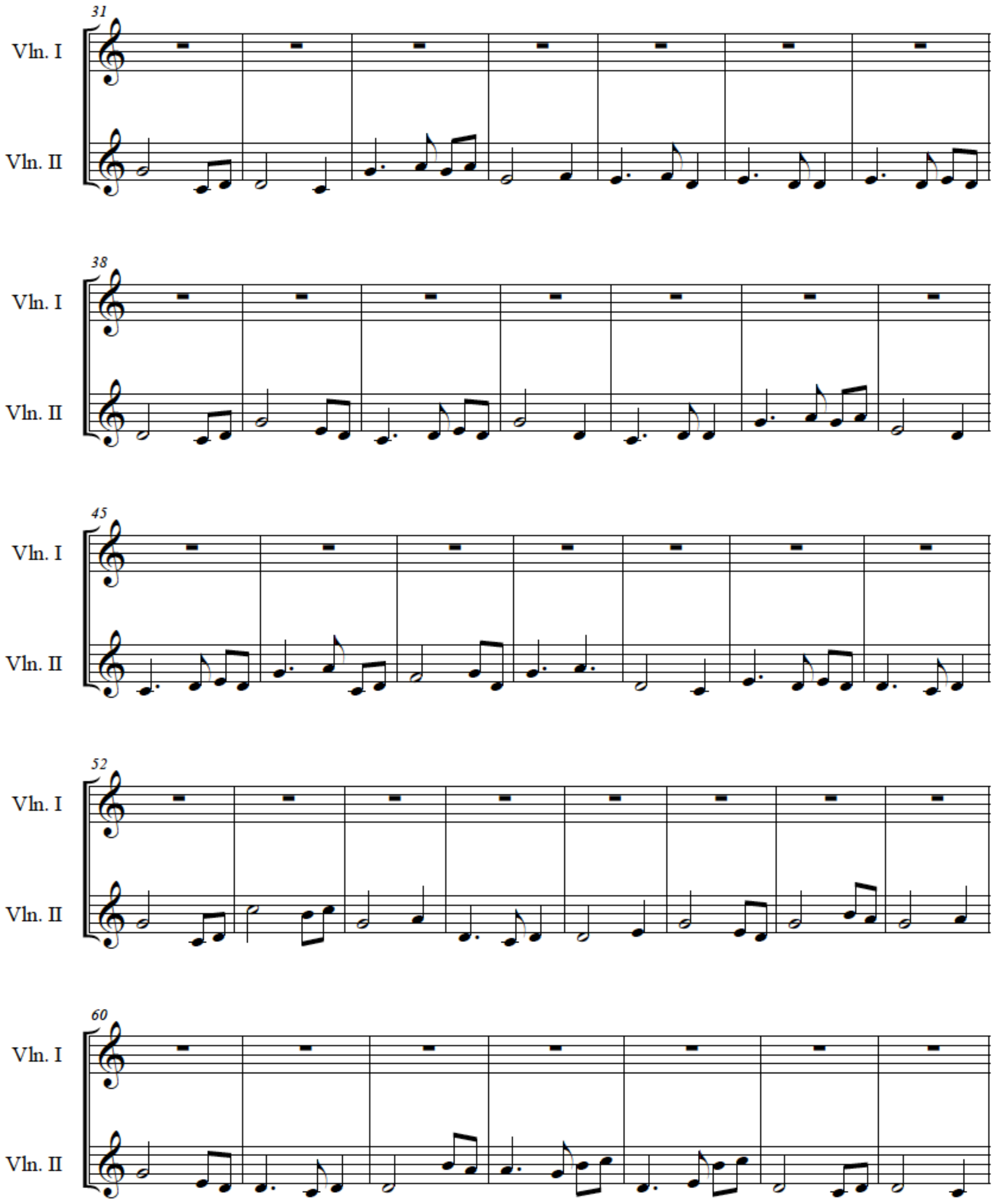
31
Vln. I
Vln. II
38
Vln. I
Vln. II
45
Vln. I
Vln. II
52
Vln. I
Vln. II
60
Vln. I
Vln. II

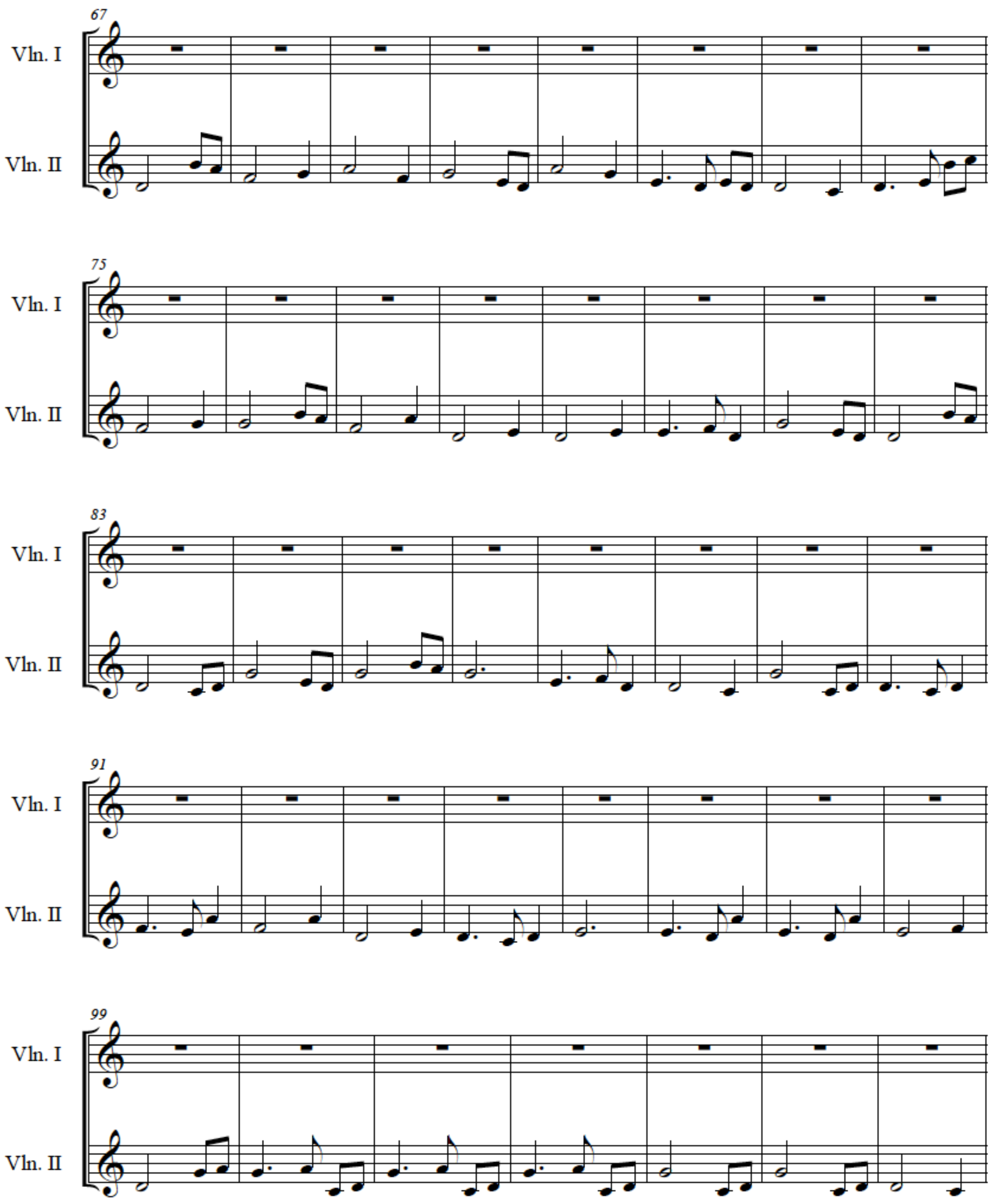
67
Vln. I
Vln. II
75
Vln. I
Vln. II
83
Vln. I
Vln. II
91
Vln. I
Vln. II
99
Vln. I
Vln. II

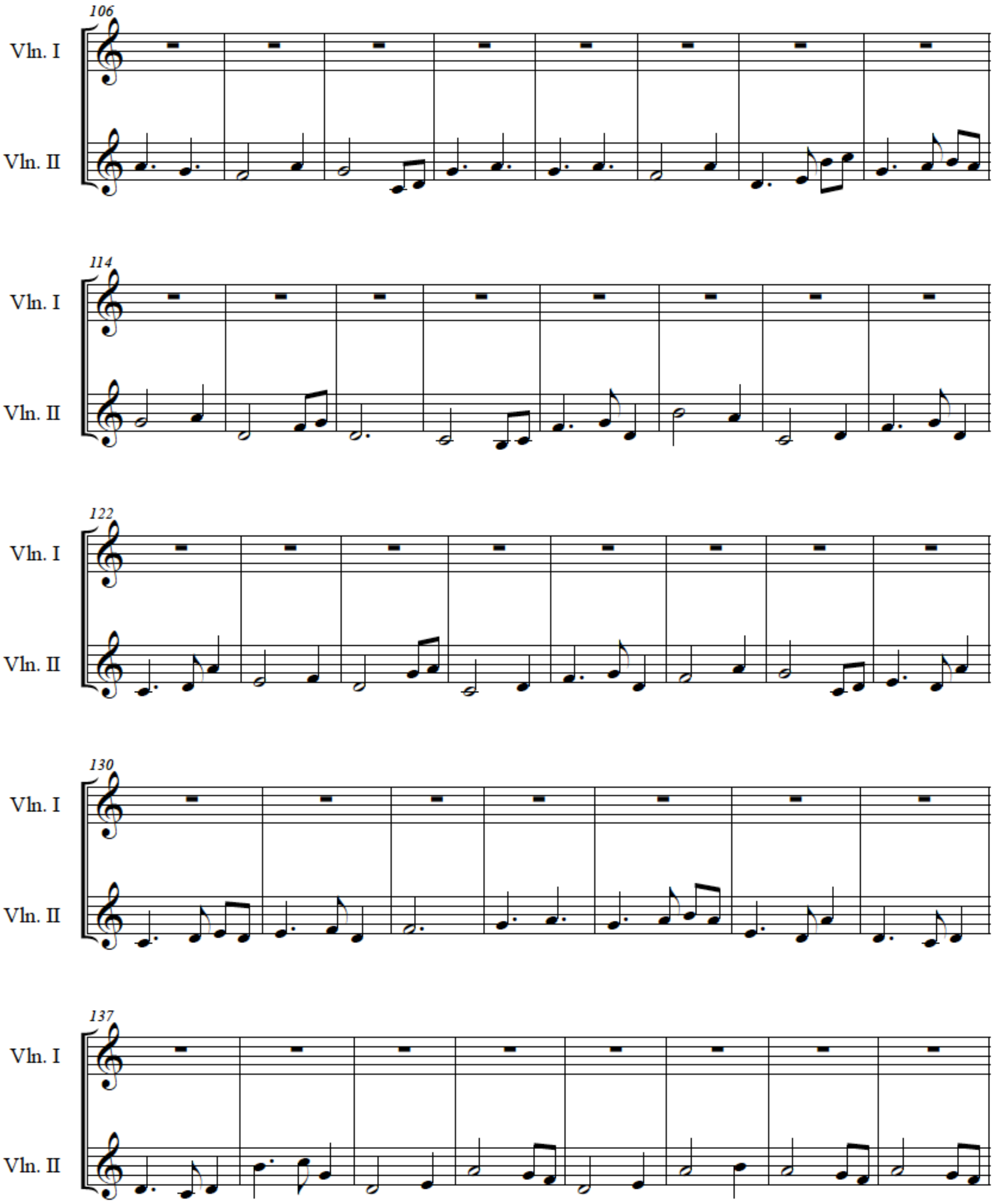
106
Vln. I
Vln. II
114
Vln. I
Vln. II
122
Vln. I
Vln. II
130
Vln. I
Vln. II
137
Vln. I
Vln. II

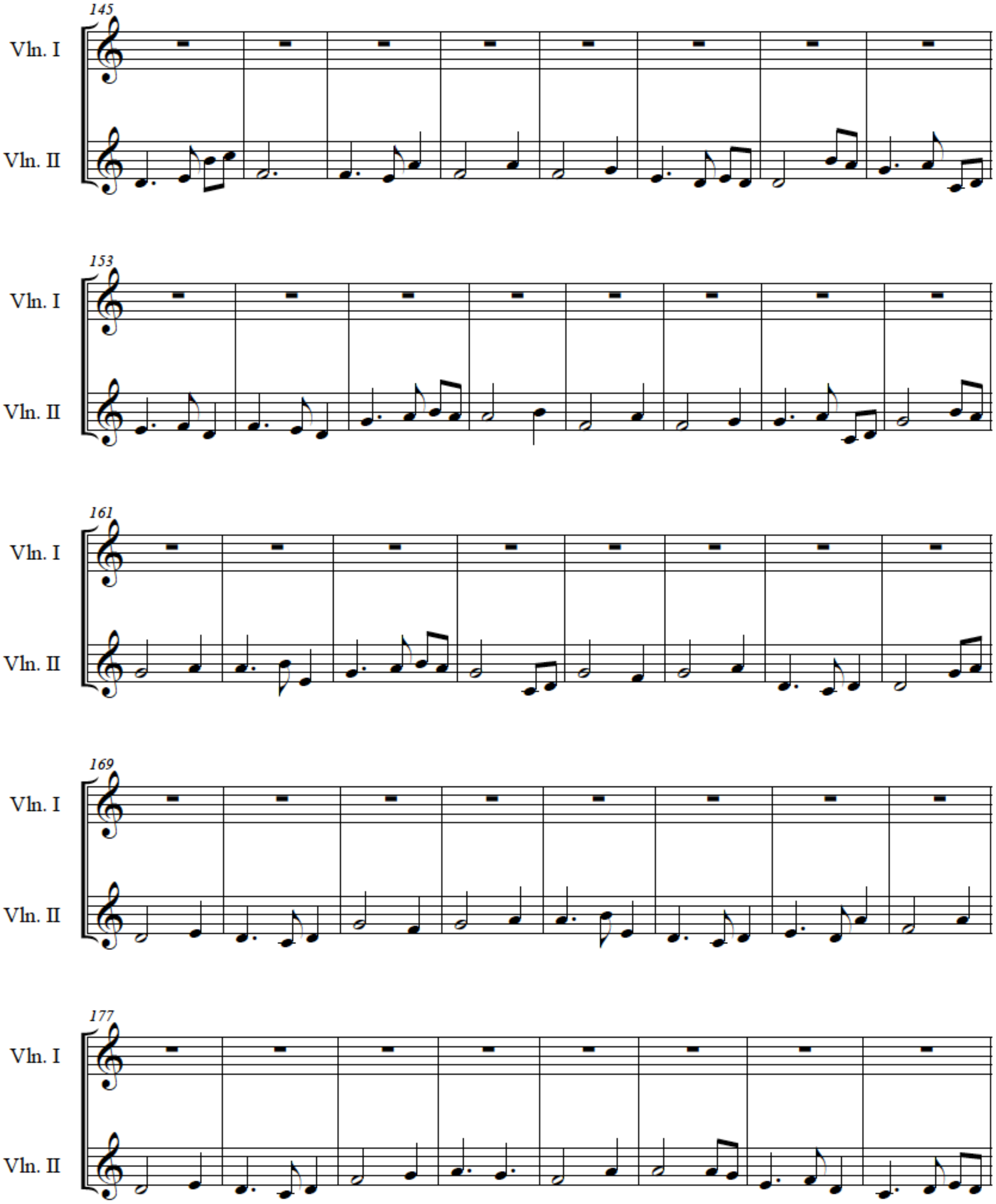
145
Vln. I
Vln. II
153
Vln. I
Vln. II
161
Vln. I
Vln. II
169
Vln. I
Vln. II
177
Vln. I
Vln. II

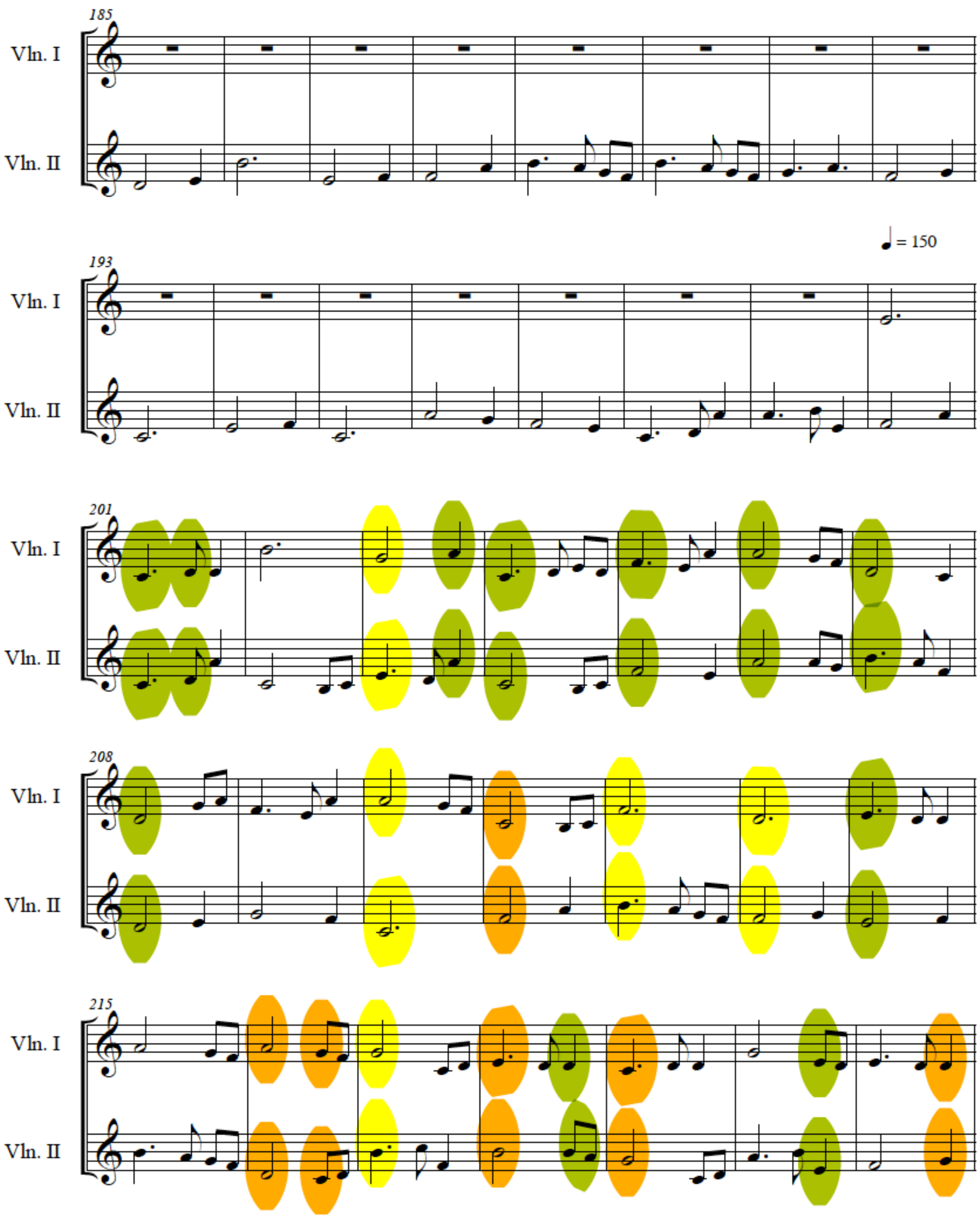
185
Vln. I
Vln. II
193
♩ = 150
201
208
215

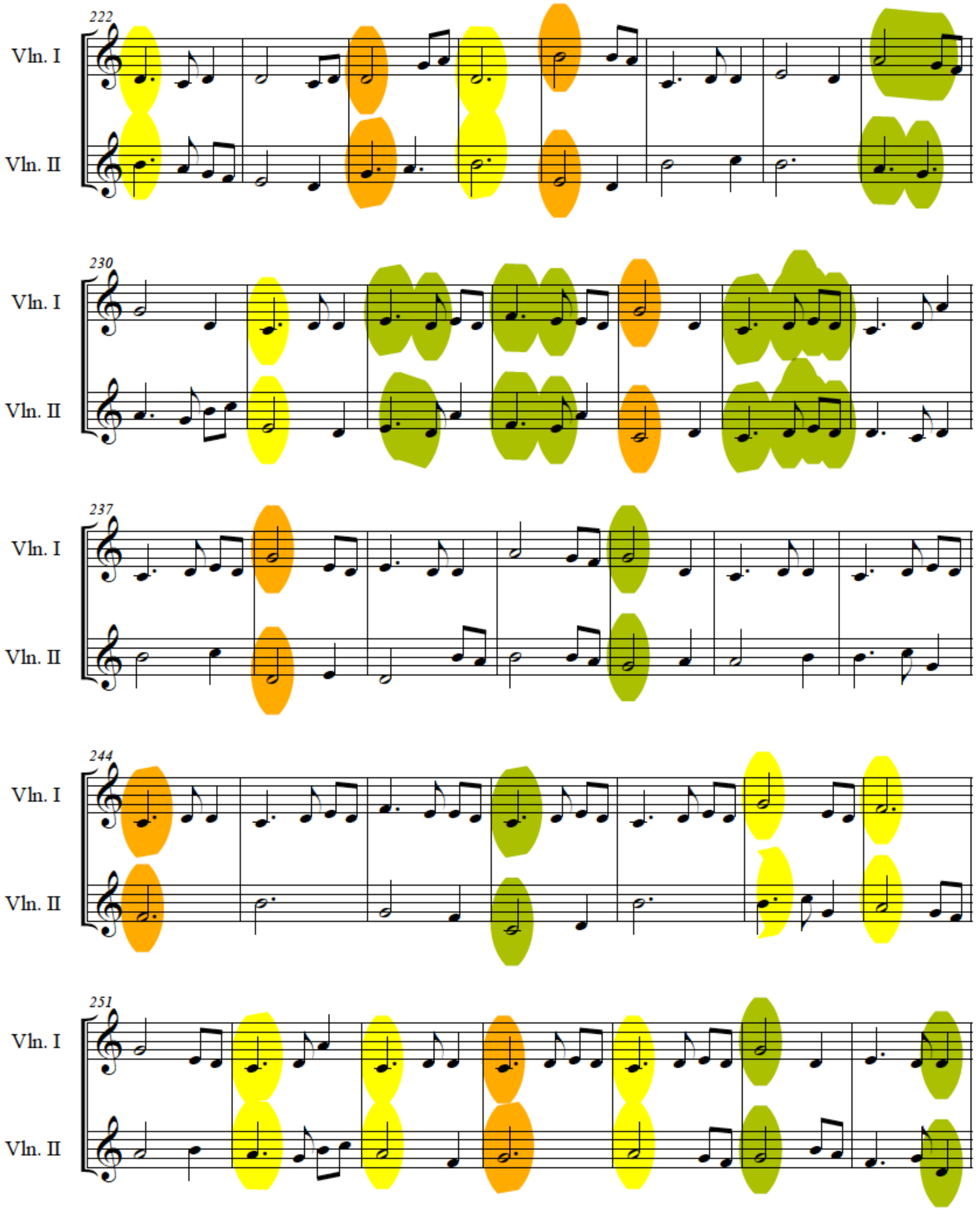
222
Vln. I
Vln. II
230
Vln. I
Vln. II
237
Vln. I
Vln. II
244
Vln. I
Vln. II
251
Vln. I
Vln. II

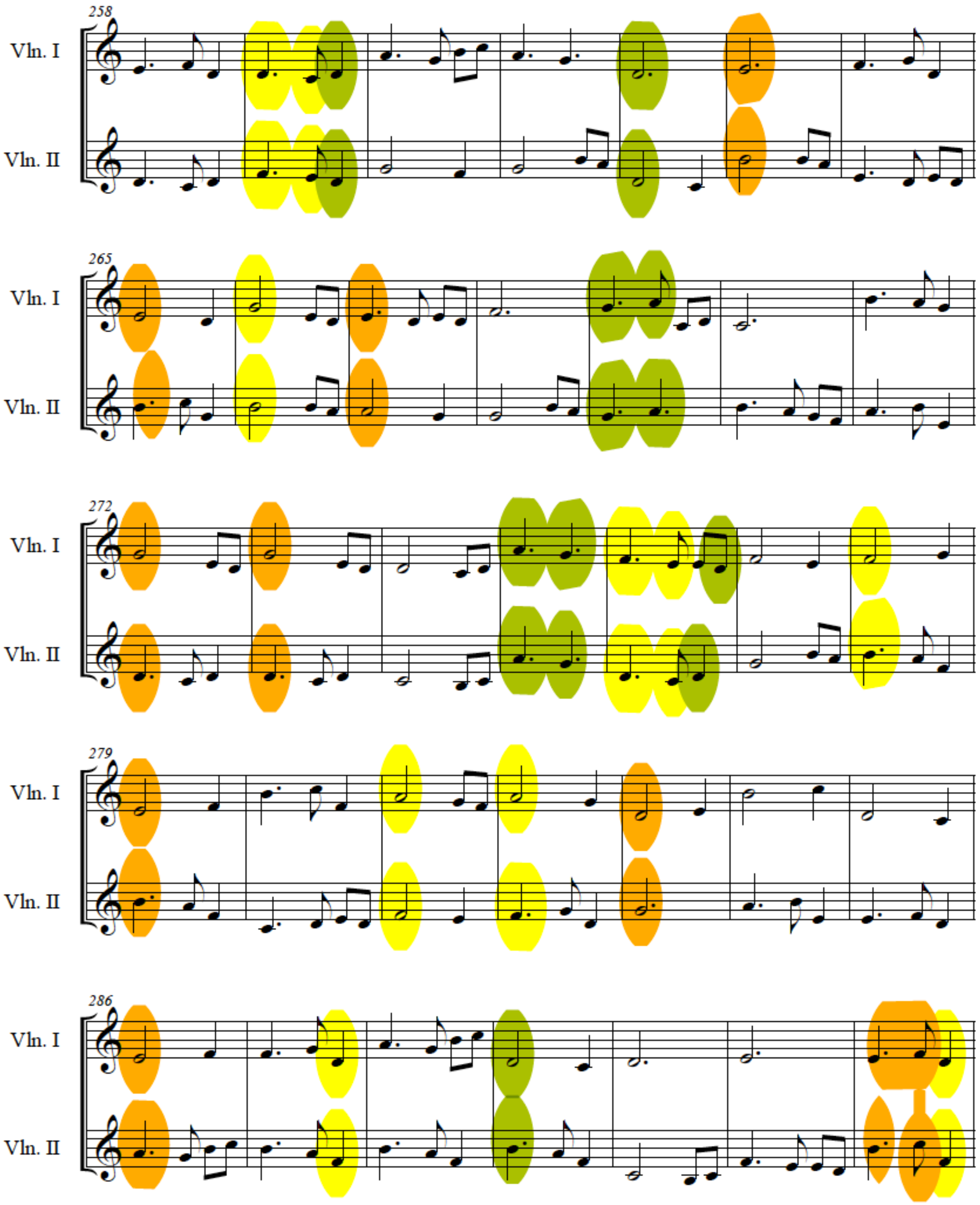
258
Vln. I
Vln. II
265
Vln. I
Vln. II
272
Vln. I
Vln. II
279
Vln. I
Vln. II
286
Vln. I
Vln. II

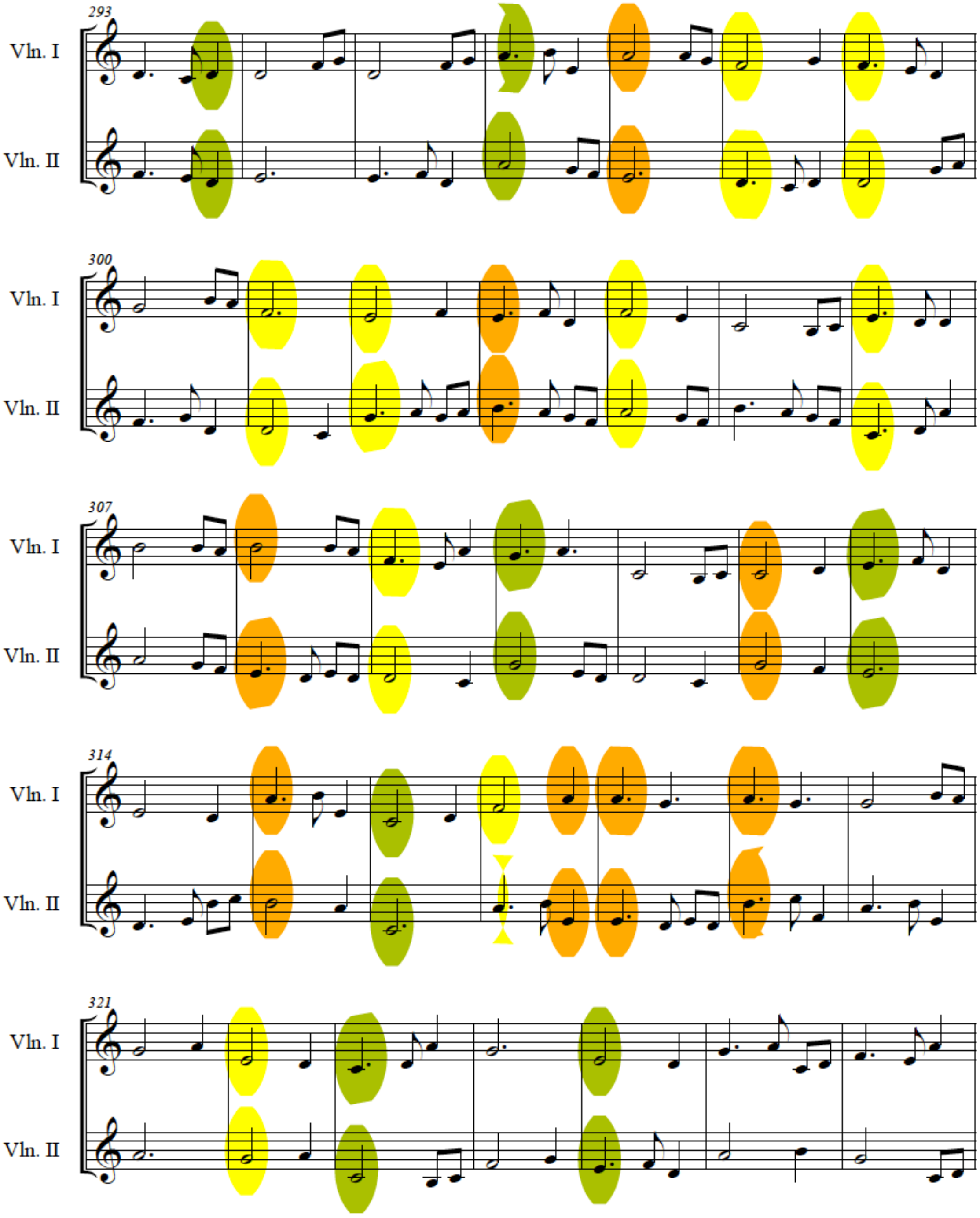
293
Vln. I
Vln. II
300
Vln. I
Vln. II
307
Vln. I
Vln. II
314
Vln. I
Vln. II
321
Vln. I
Vln. II

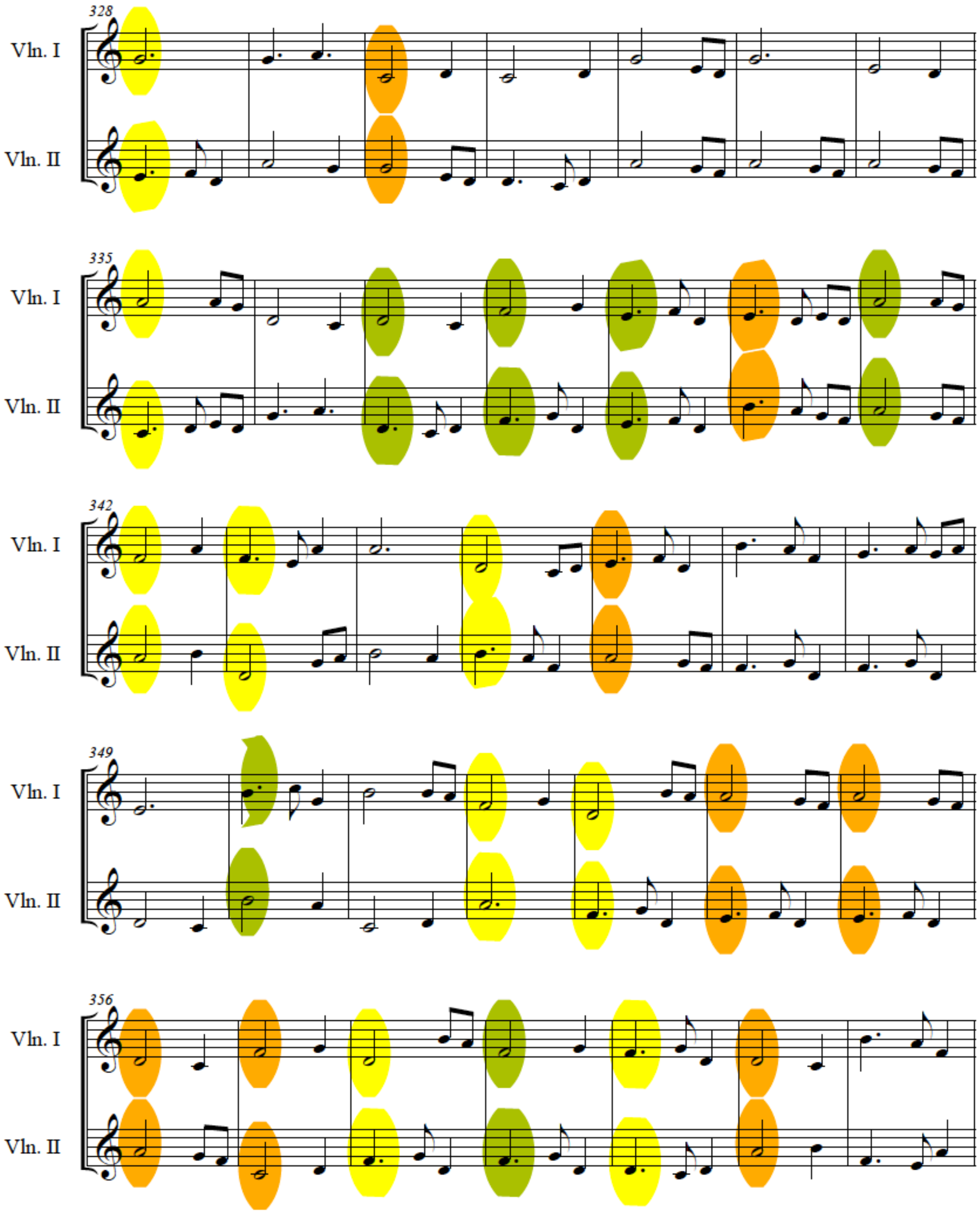
328
Vln. I
Vln. II
335
Vln. I
Vln. II
342
Vln. I
Vln. II
349
Vln. I
Vln. II
356
Vln. I
Vln. II

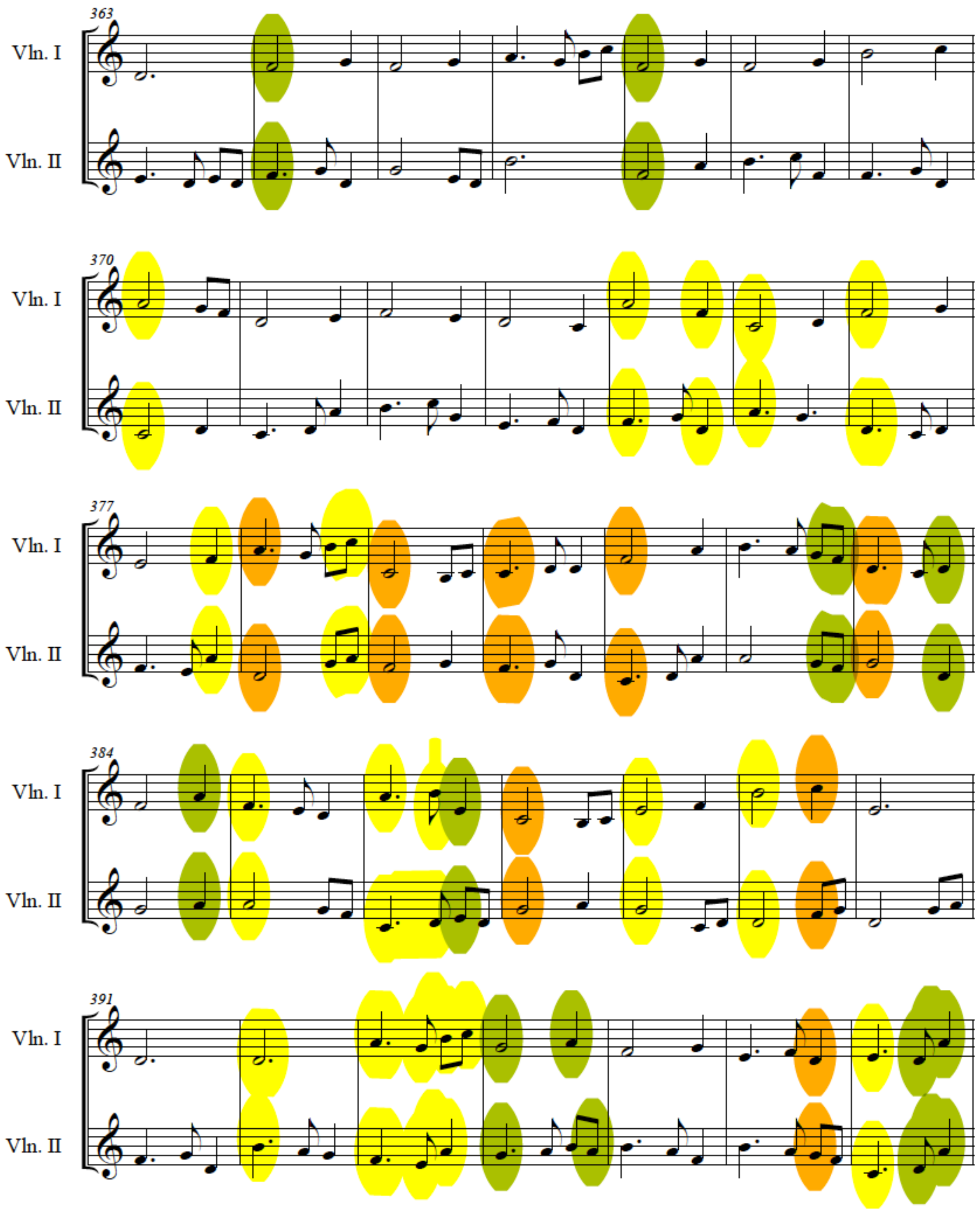
363
Vln. I
Vln. II
370
Vln. I
Vln. II
377
Vln. I
Vln. II
384
Vln. I
Vln. II
391
Vln. I
Vln. II

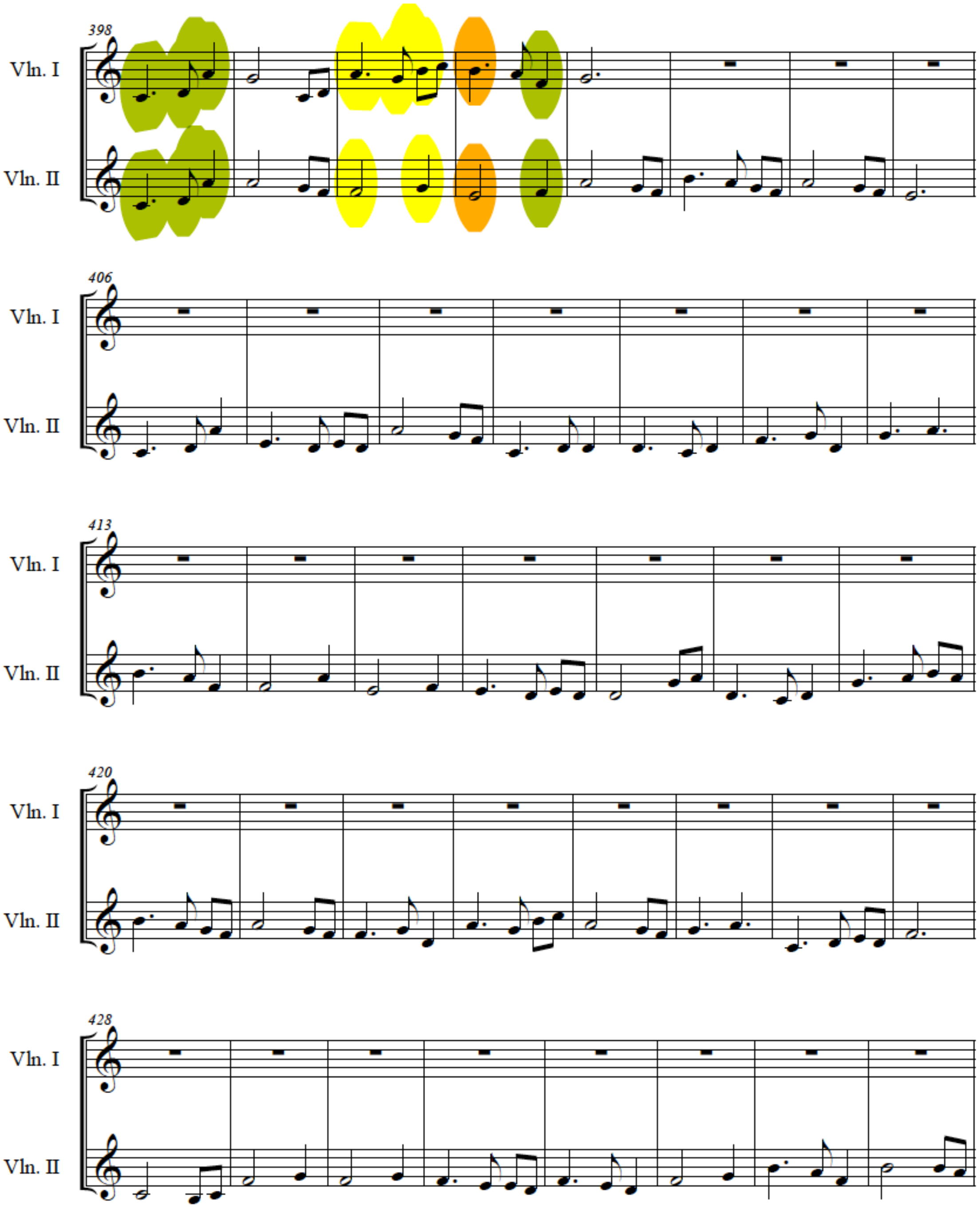
398
Vln. I
Vln. II
406
Vln. I
Vln. II
413
Vln. I
Vln. II
420
Vln. I
Vln. II
428
Vln. I
Vln. II

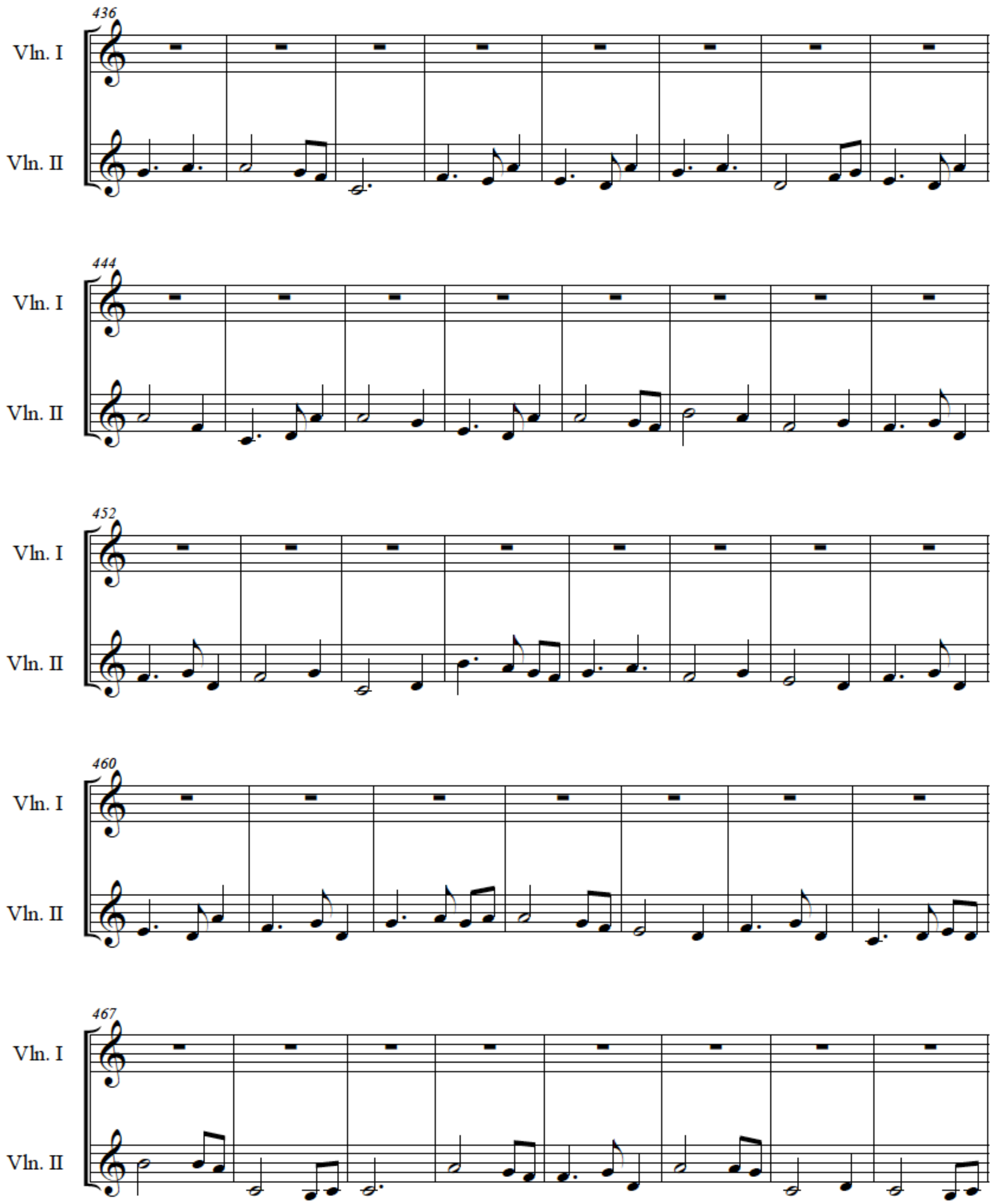
436
Vln. I
Vln. II
444
Vln. I
Vln. II
452
Vln. I
Vln. II
460
Vln. I
Vln. II
467
Vln. I
Vln. II

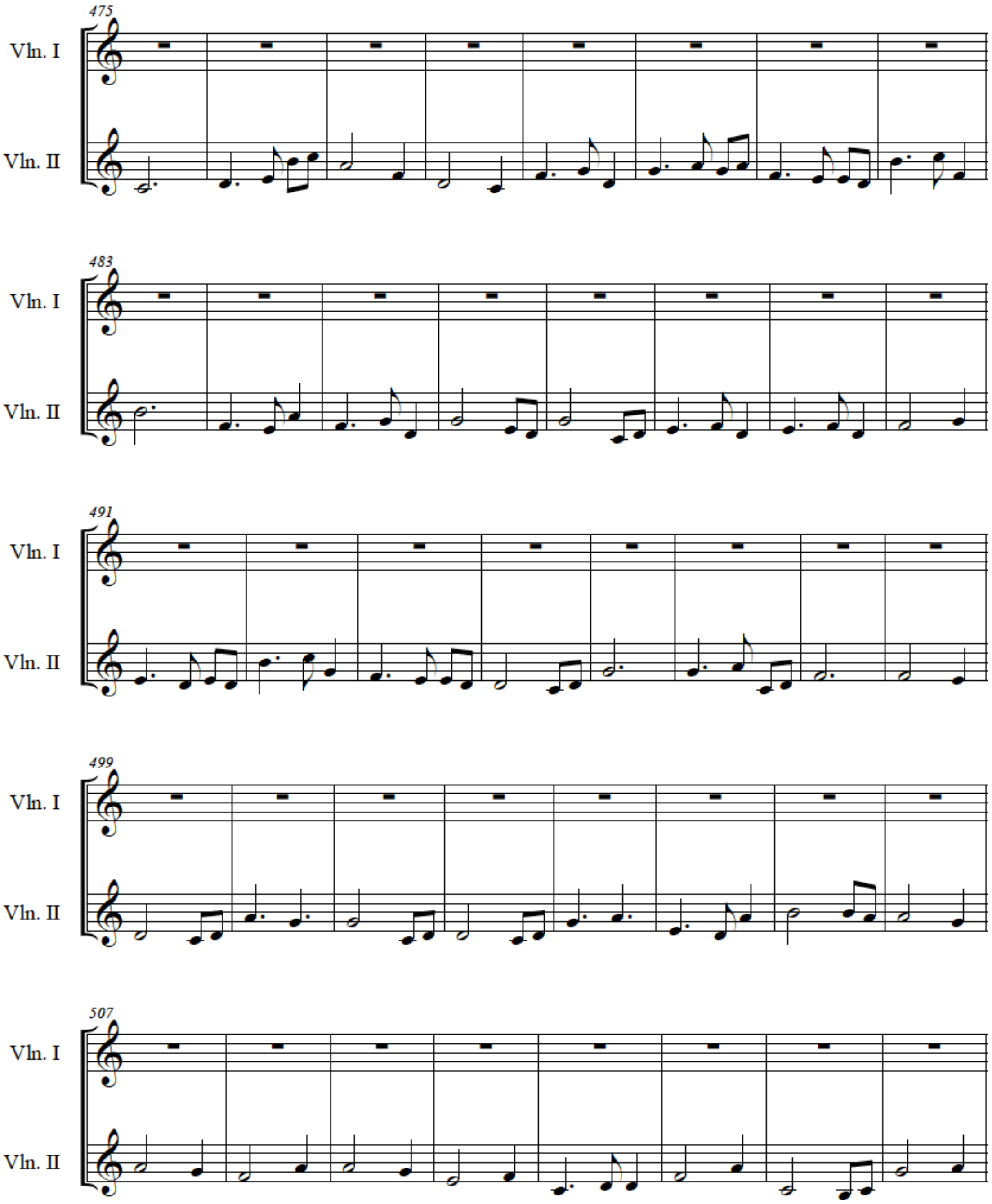
475
Vln. I
Vln. II
483
Vln. I
Vln. II
491
Vln. I
Vln. II
499
Vln. I
Vln. II
507
Vln. I
Vln. II

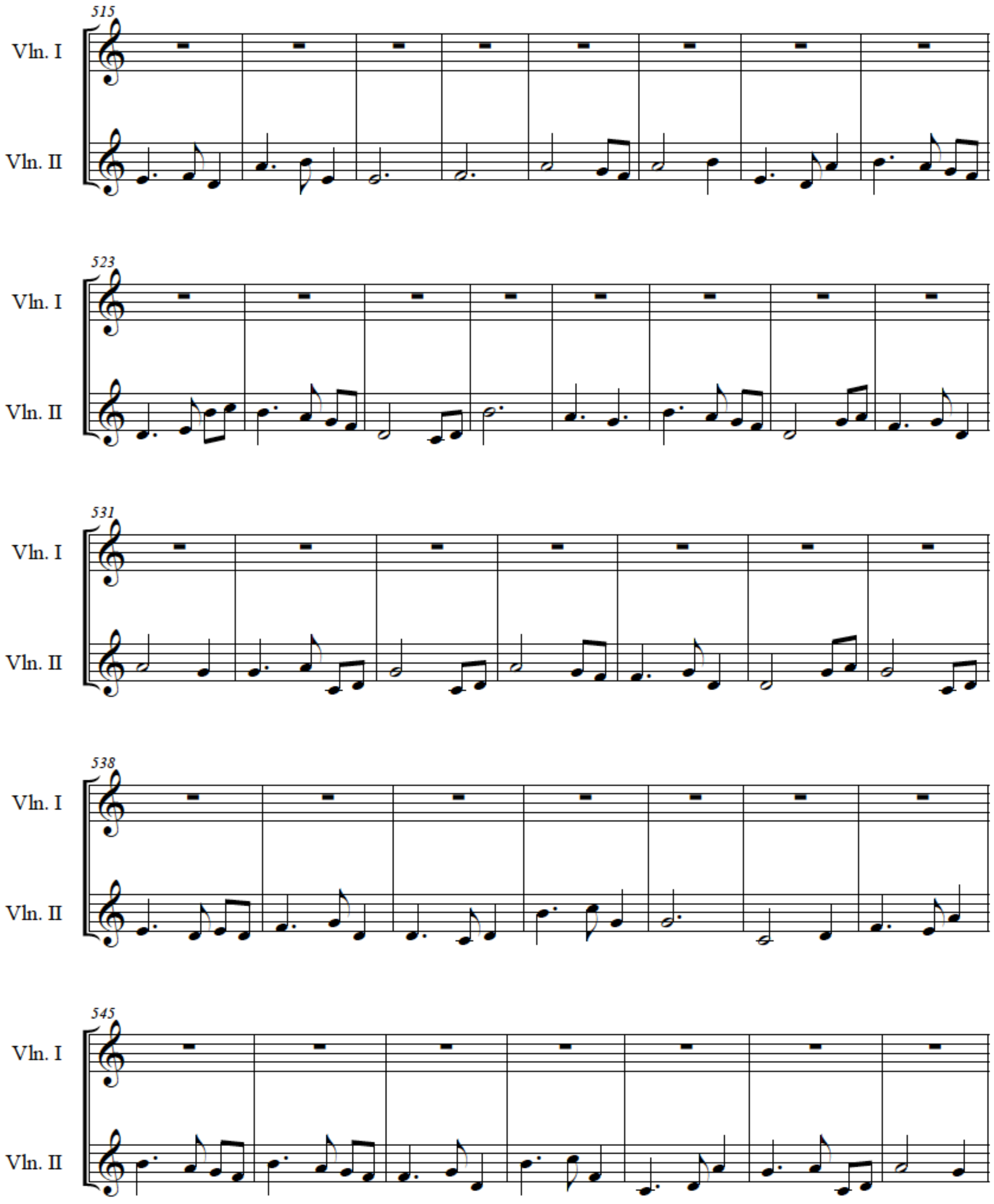
515
Vln. I
Vln. II
523
Vln. I
Vln. II
531
Vln. I
Vln. II
538
Vln. I
Vln. II
545
Vln. I
Vln. II

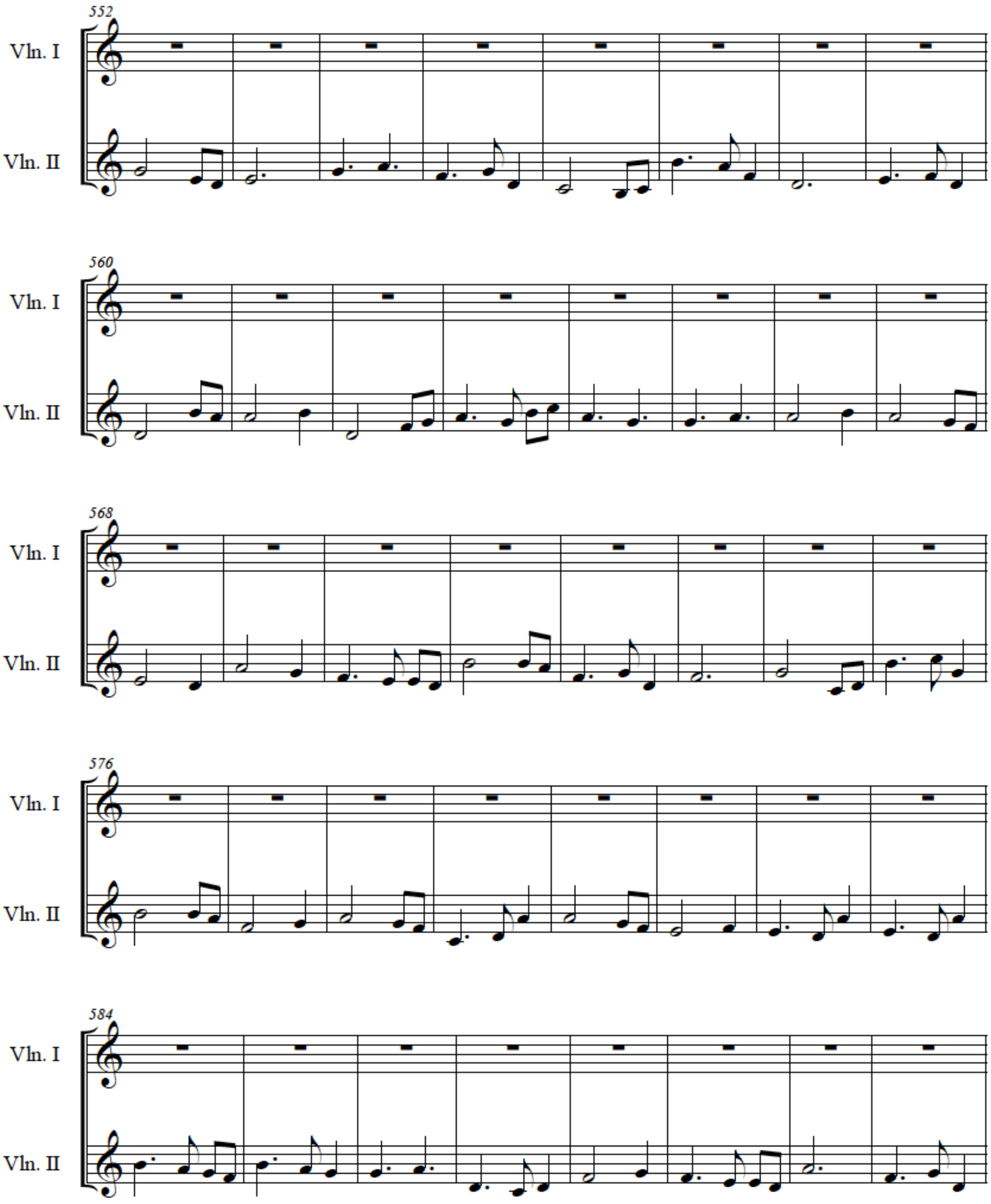
552
Vln. I
Vln. II
560
Vln. I
Vln. II
568
Vln. I
Vln. II
576
Vln. I
Vln. II
584
Vln. I
Vln. II

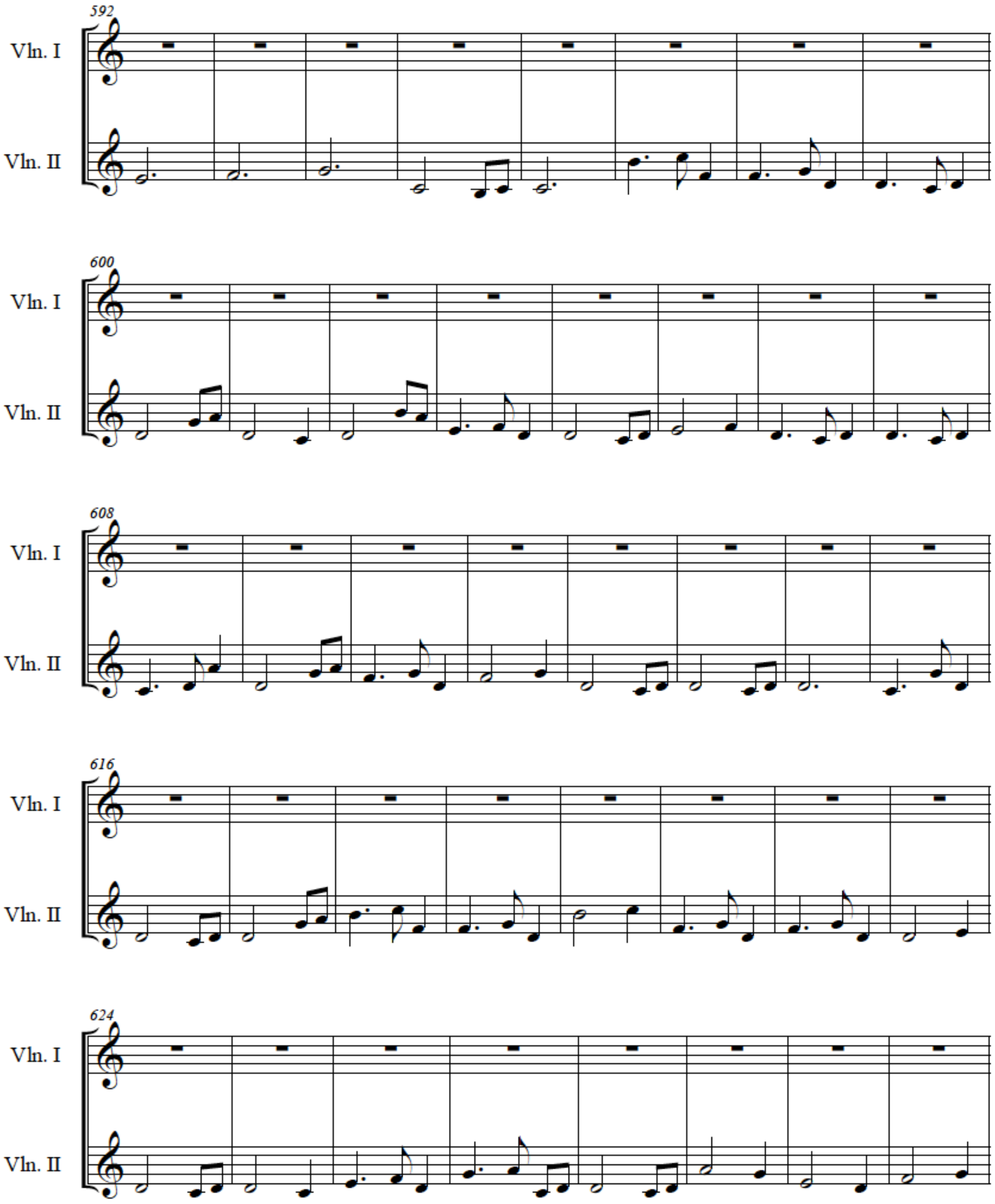
592
Vln. I
Vln. II
600
Vln. I
Vln. II
608
Vln. I
Vln. II
616
Vln. I
Vln. II
624
Vln. I
Vln. II

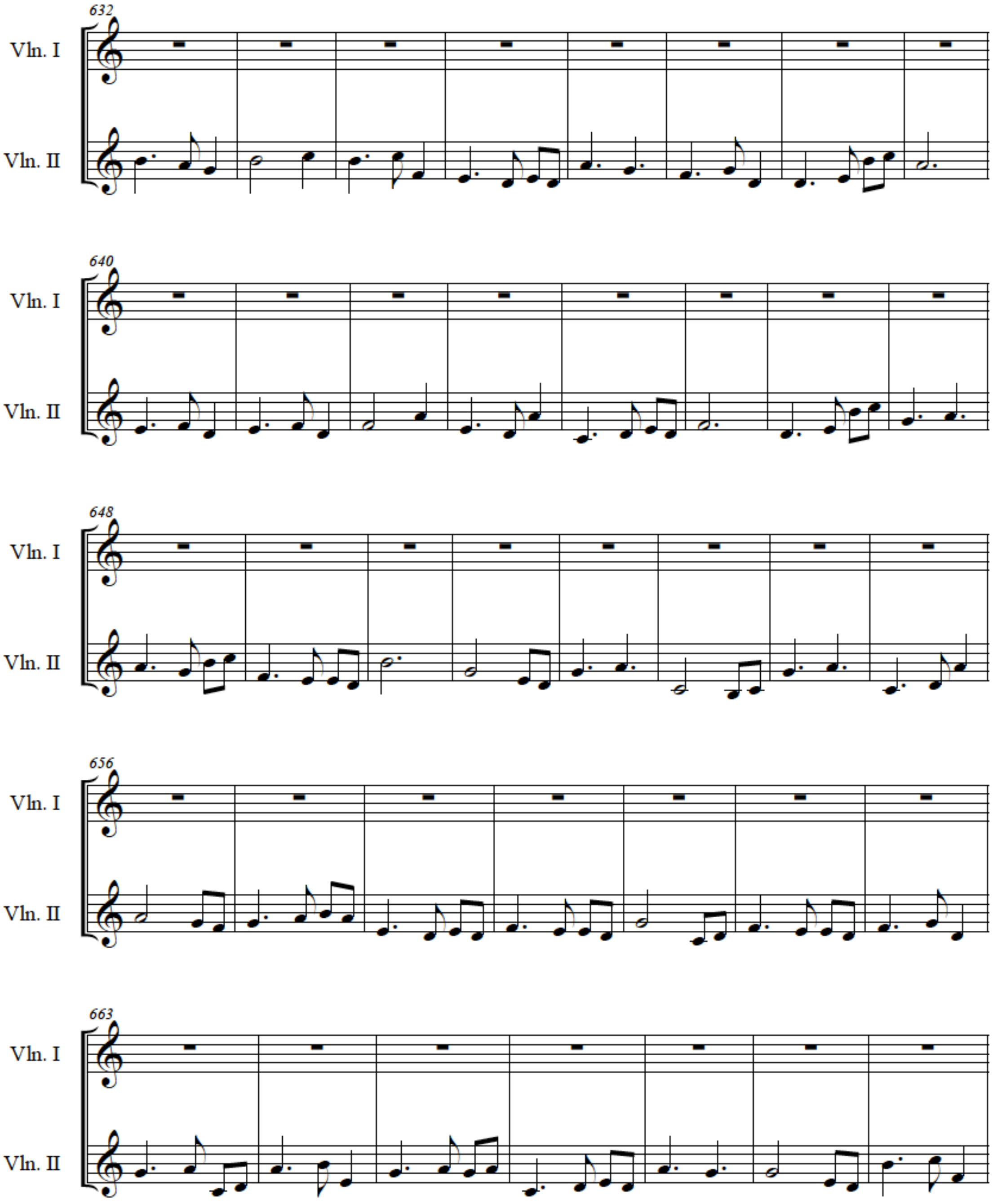
632
Vln. I
Vln. II
640
Vln. I
Vln. II
648
Vln. I
Vln. II
656
Vln. I
Vln. II
663
Vln. I
Vln. II

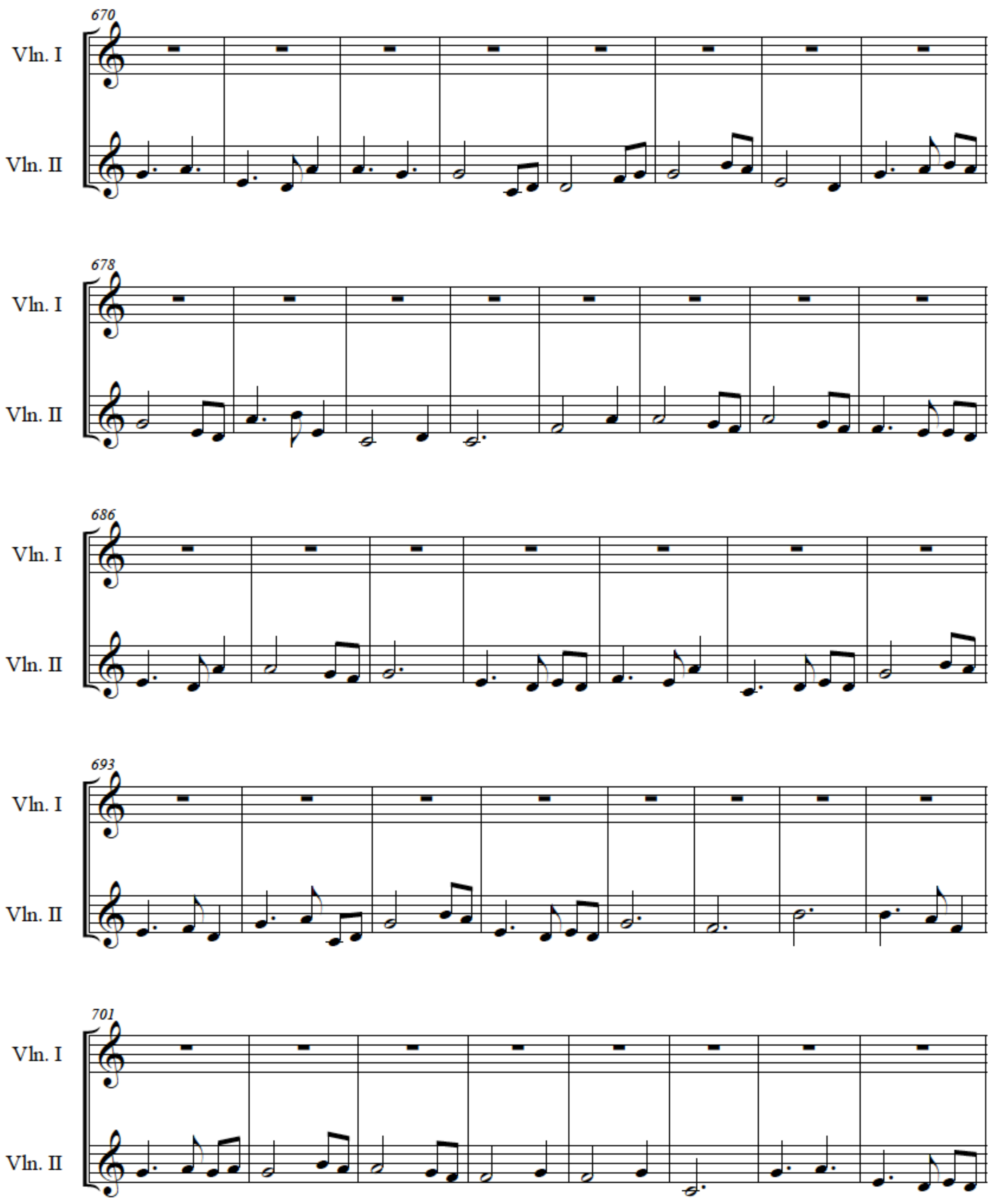
670
Vln. I
Vln. II
678
Vln. I
Vln. II
686
Vln. I
Vln. II
693
Vln. I
Vln. II
701
Vln. I
Vln. II

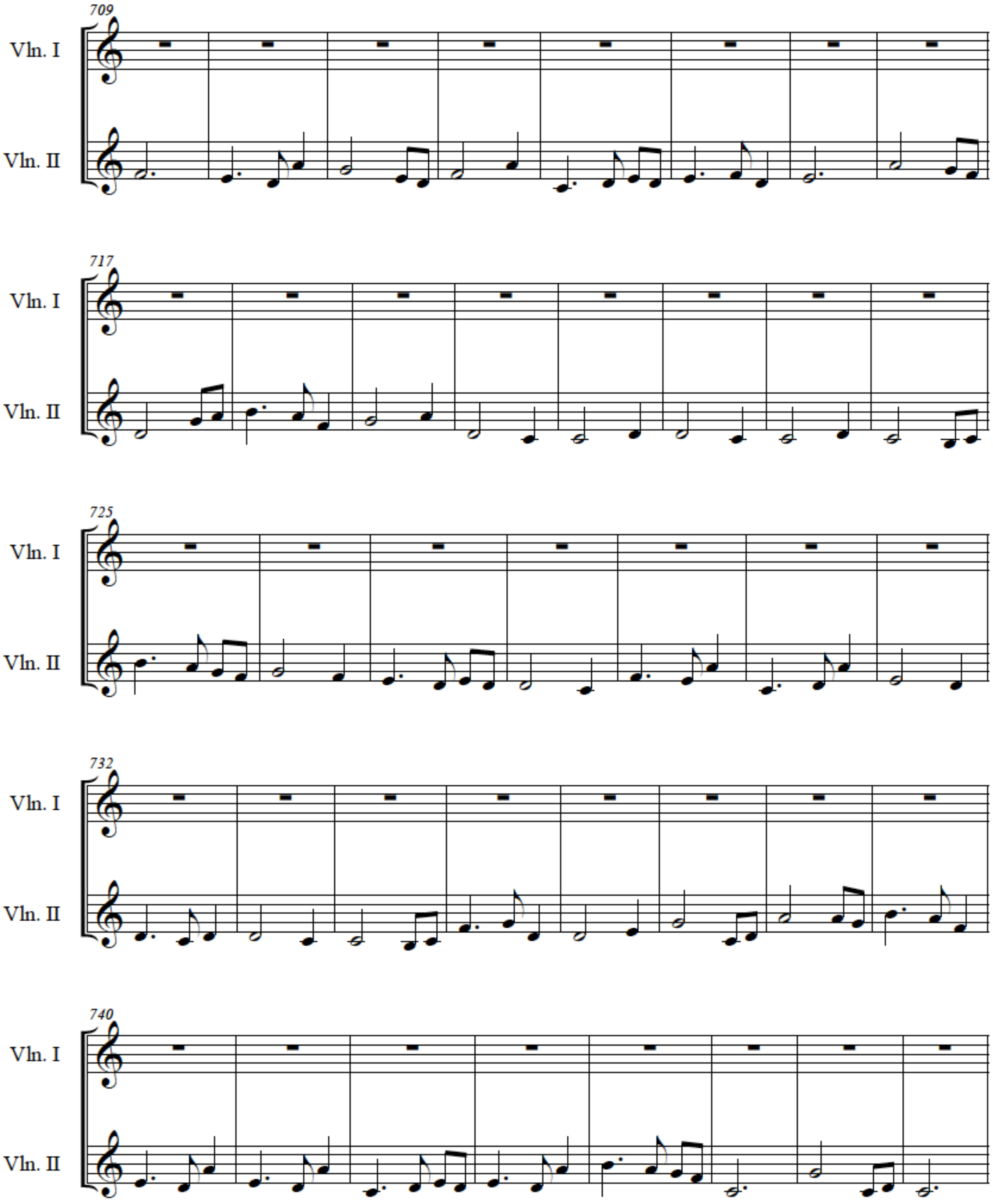
709
Vln. I
Vln. II
717
Vln. I
Vln. II
725
Vln. I
Vln. II
732
Vln. I
Vln. II
740
Vln. I
Vln. II

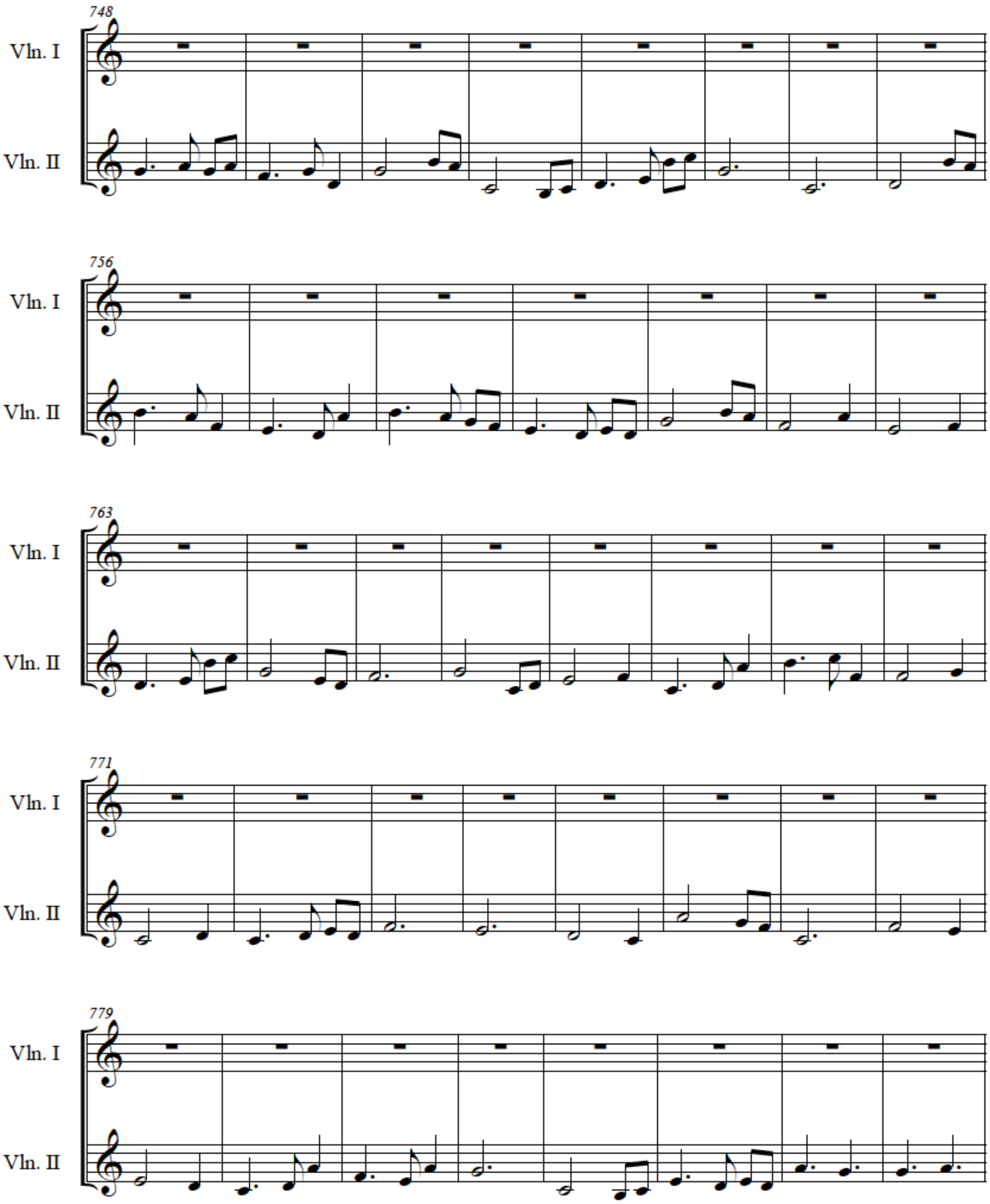
748
Vln. I
Vln. II
756
Vln. I
Vln. II
763
Vln. I
Vln. II
771
Vln. I
Vln. II
779
Vln. I
Vln. II

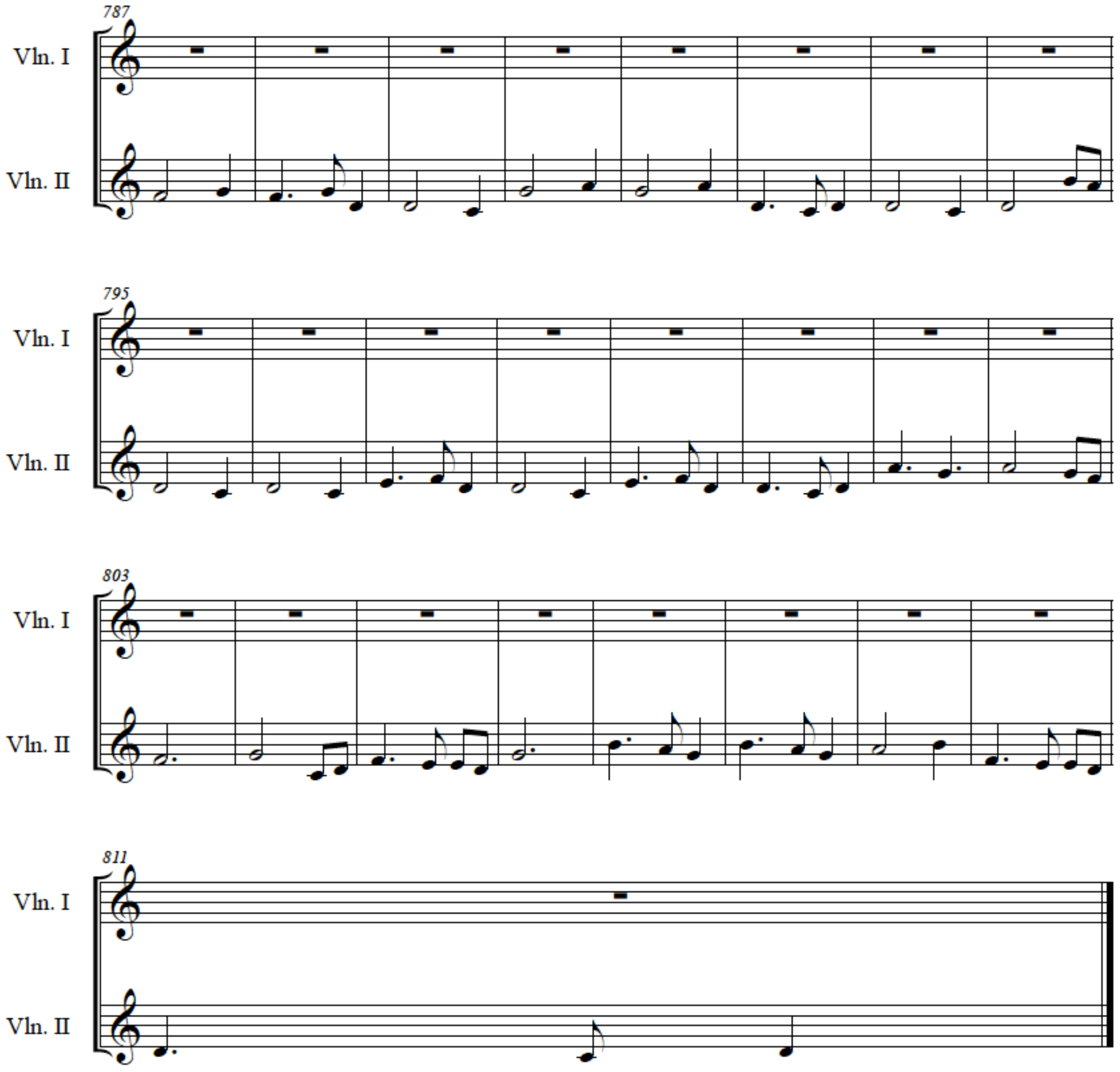
787
Vln. I
Vln. II
795
Vln. I
Vln. II
803
Vln. I
Vln. II
811
Vln. I
Vln. II

Unison

Third / Sixth interval

Fourth / Fifth interval

AKR2-THRAP5

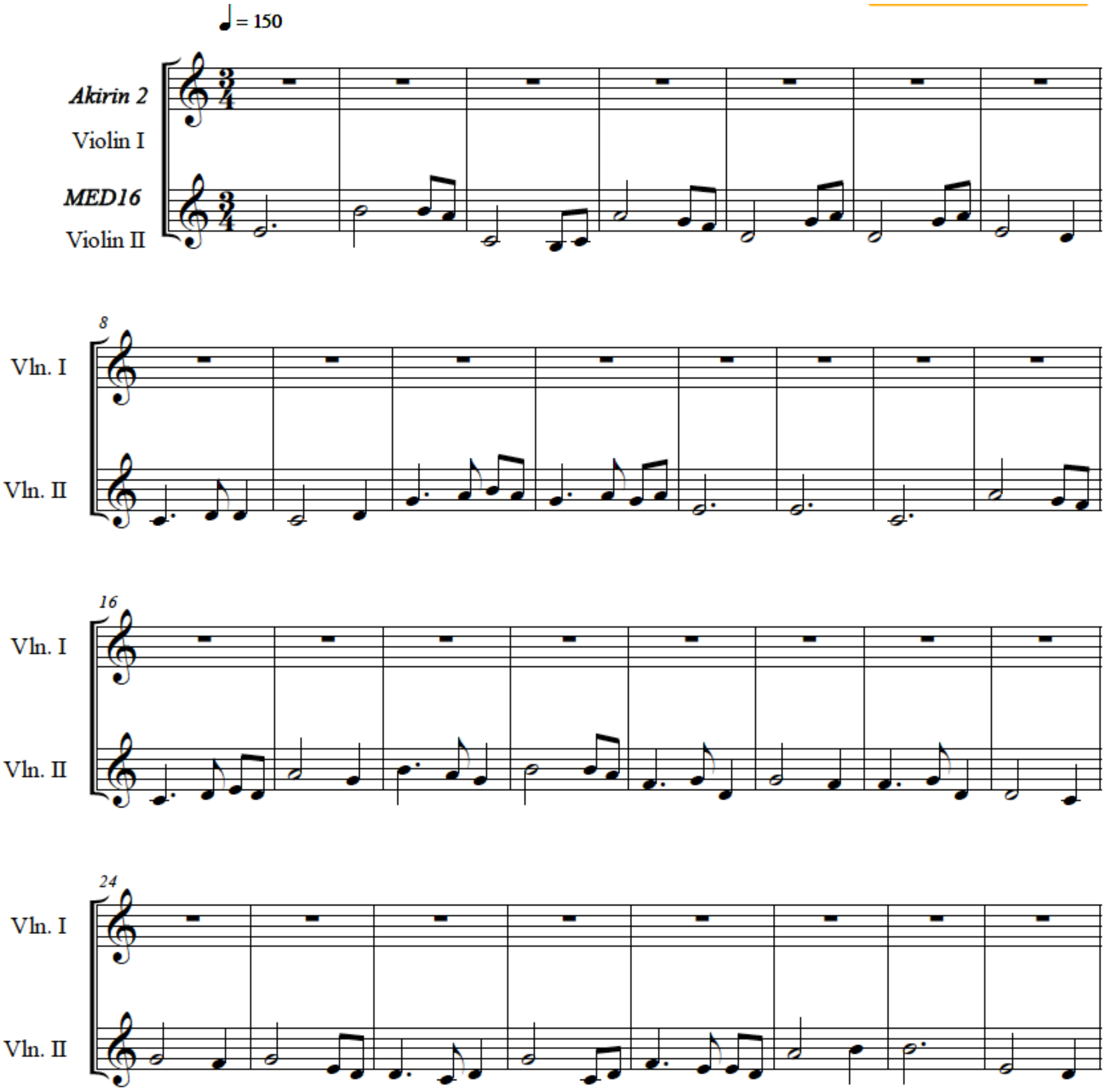

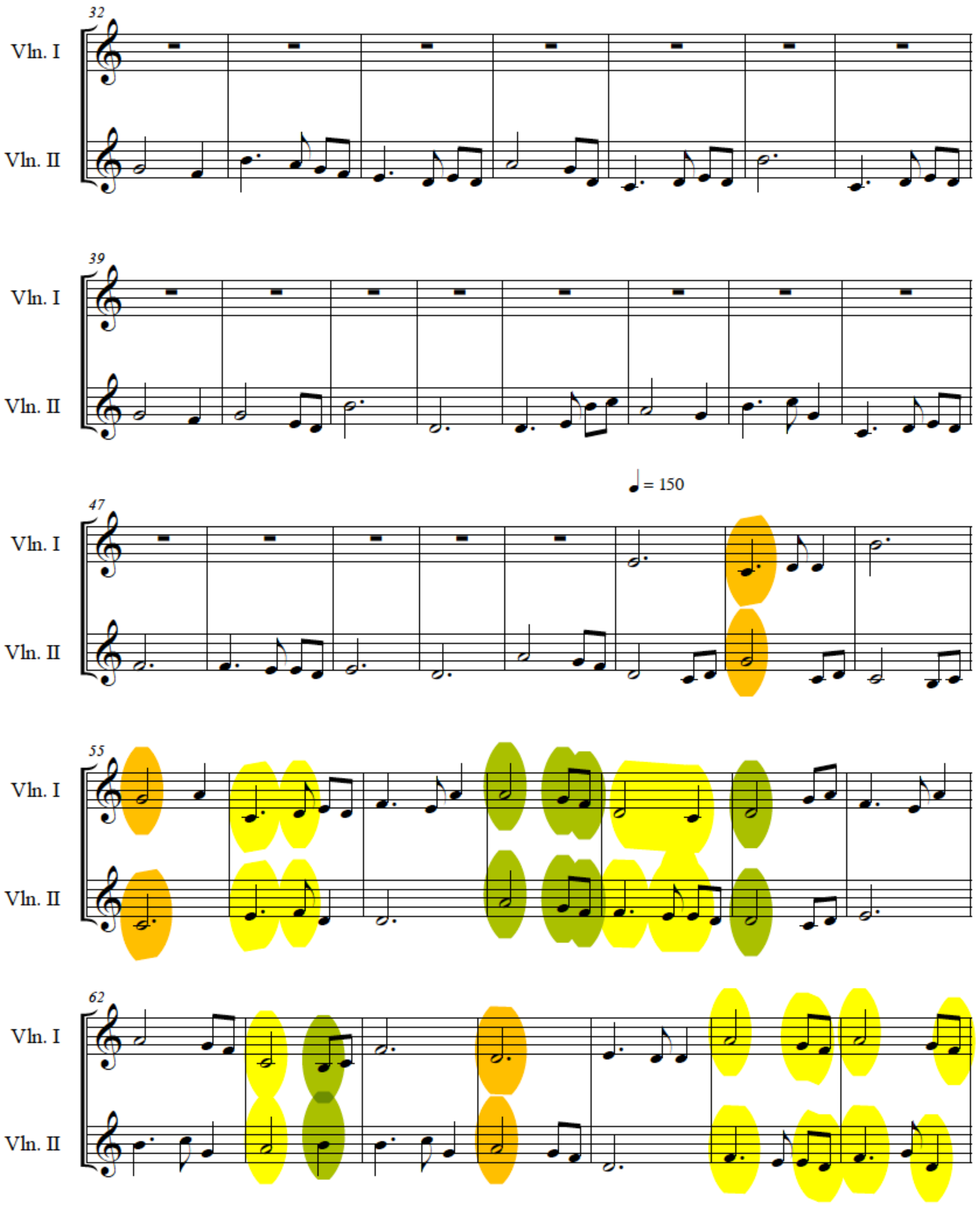
32
Vln. I
Vln. II
39
Vln. I
Vln. II
47
♩ = 150
Vln. I
Vln. II
55
Vln. I
Vln. II
62
Vln. I
Vln. II

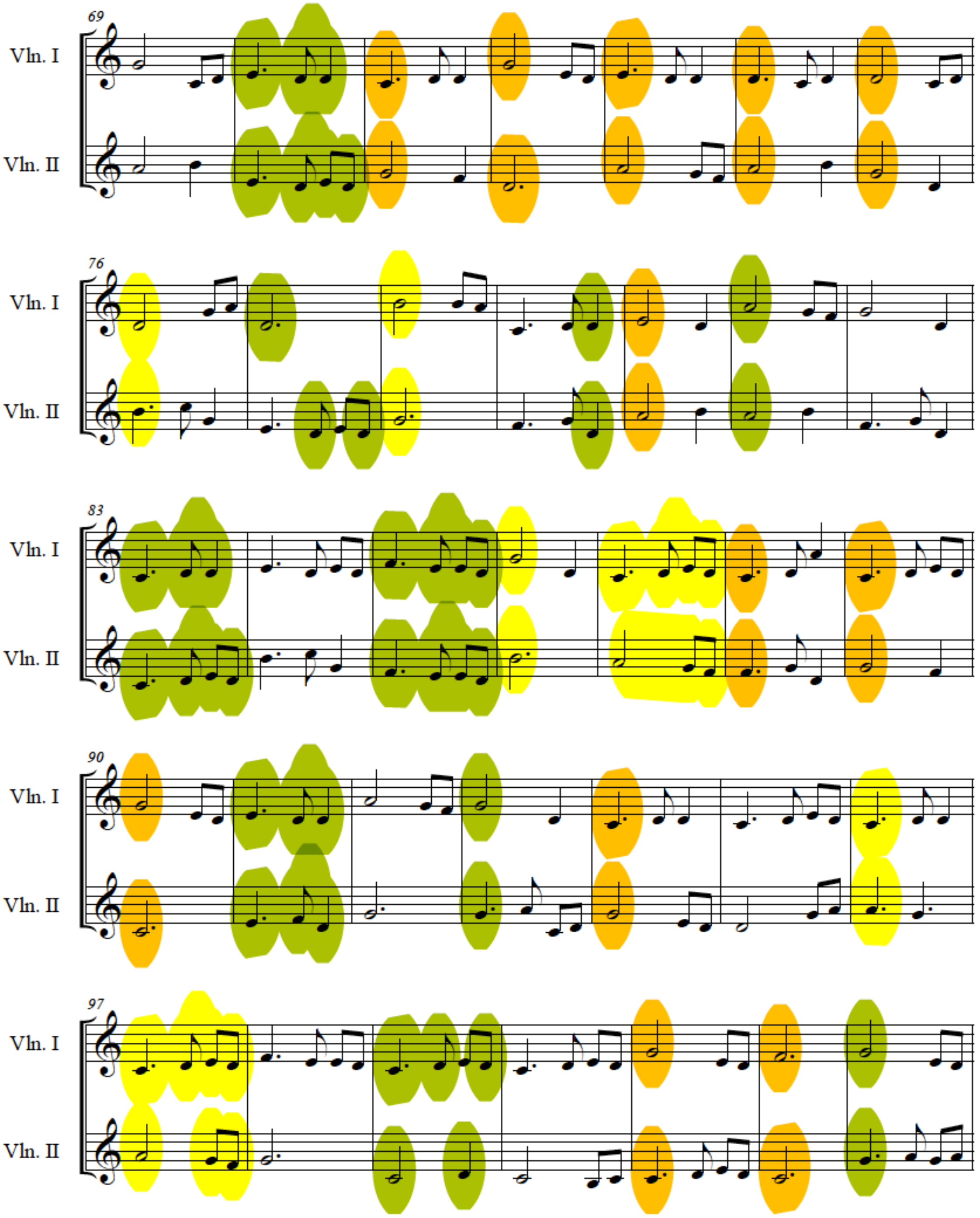
69
Vln. I
Vln. II
76
Vln. I
Vln. II
83
Vln. I
Vln. II
90
Vln. I
Vln. II
97
Vln. I
Vln. II

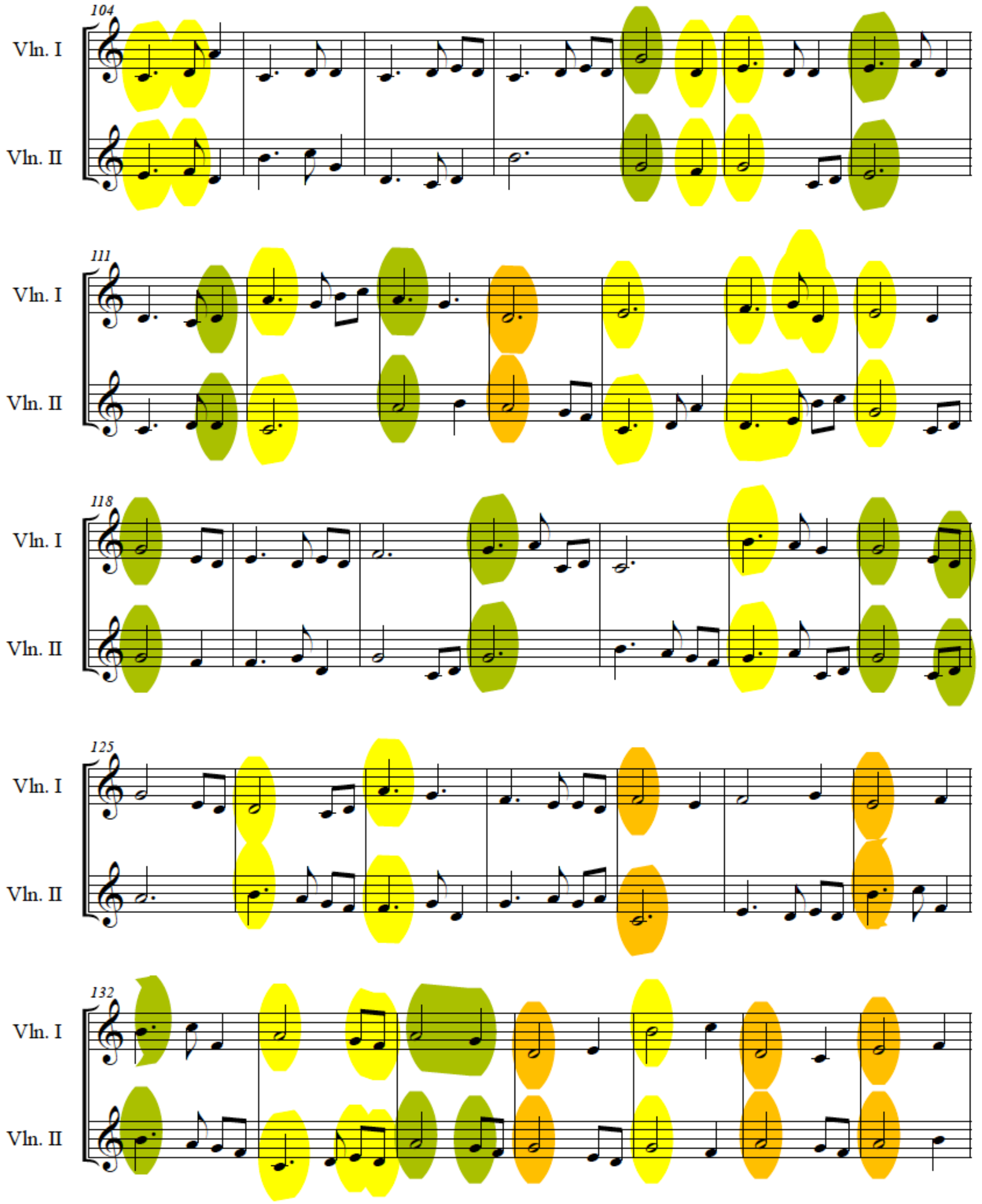
104
Vln. I
Vln. II
111
Vln. I
Vln. II
118
Vln. I
Vln. II
125
Vln. I
Vln. II
132
Vln. I
Vln. II

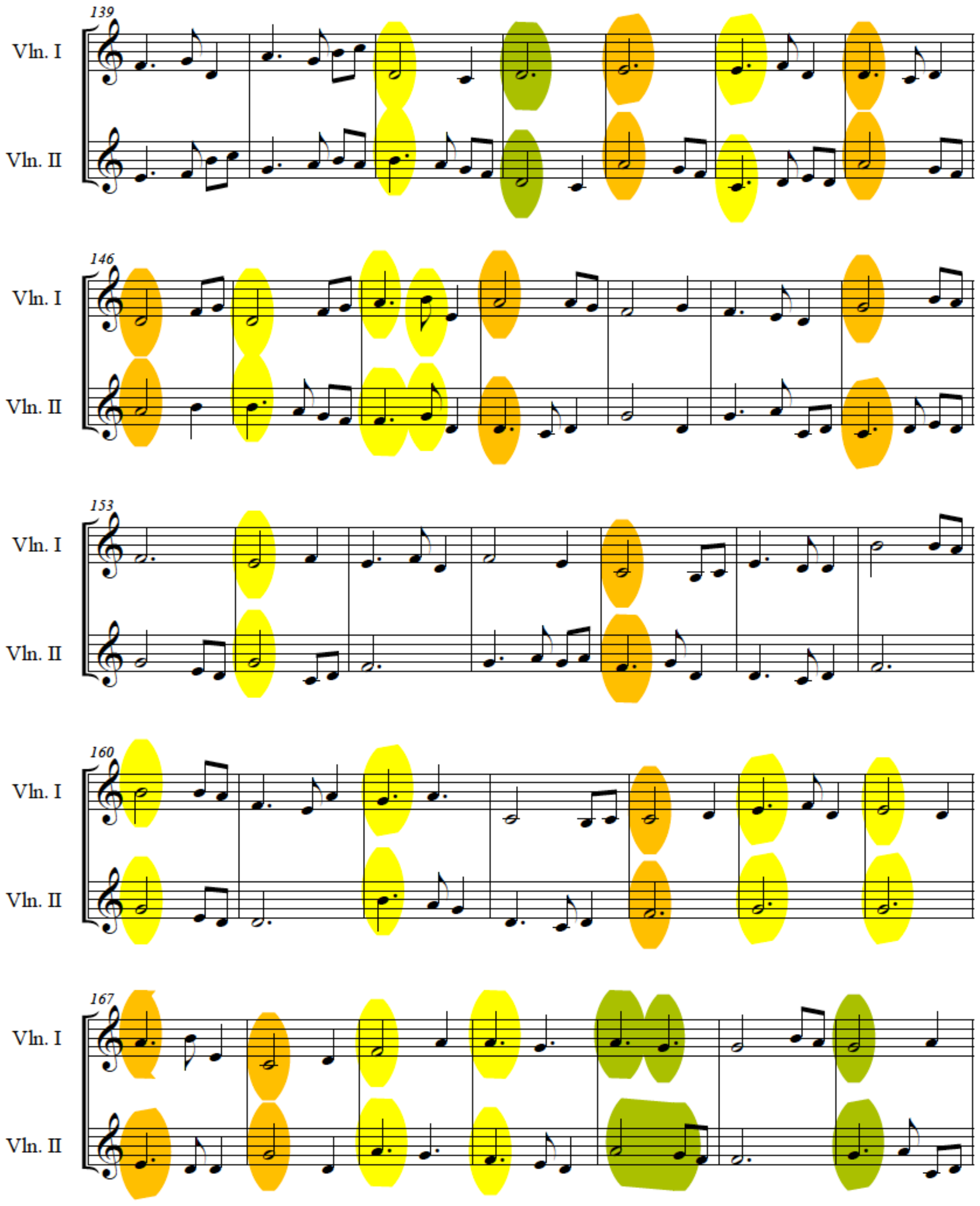
139
Vln. I
Vln. II
146
Vln. I
Vln. II
153
Vln. I
Vln. II
160
Vln. I
Vln. II
167
Vln. I
Vln. II

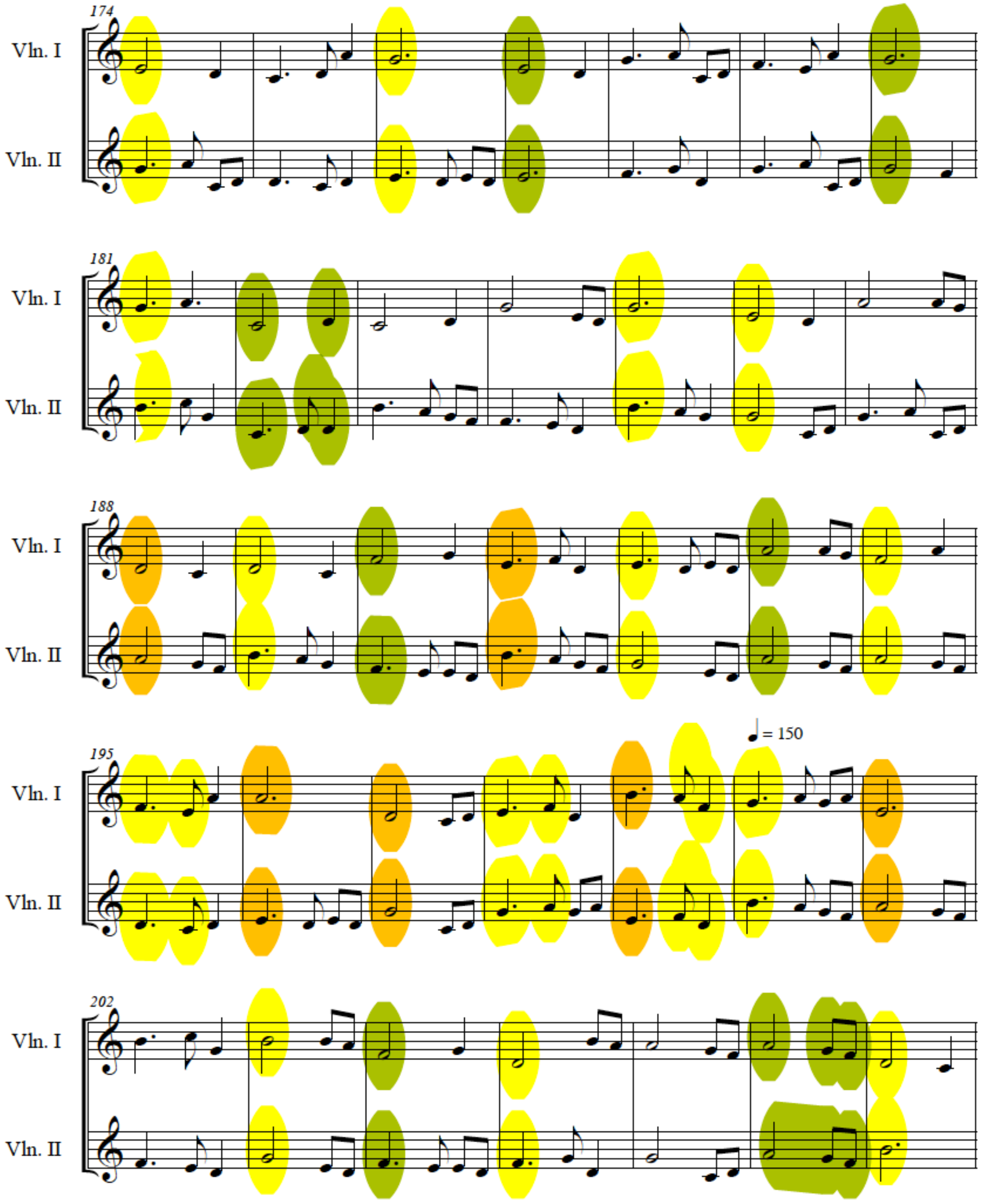
174
Vln. I
Vln. II
181
Vln. I
Vln. II
188
Vln. I
Vln. II
195
= 150
Vln. I
Vln. II
202
Vln. I
Vln. II

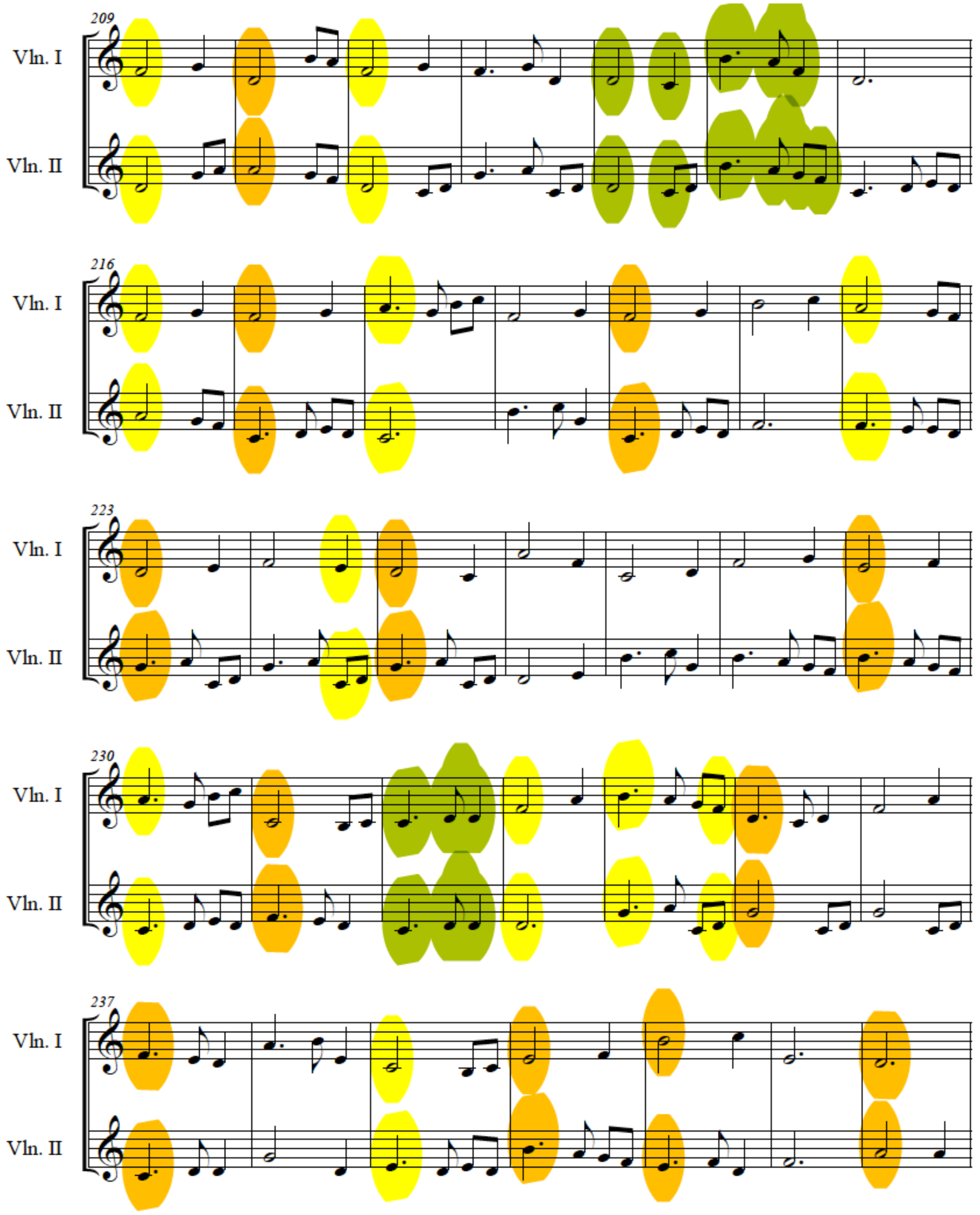
209
Vln. I
Vln. II
216
Vln. I
Vln. II
223
Vln. I
Vln. II
230
Vln. I
Vln. II
237
Vln. I
Vln. II

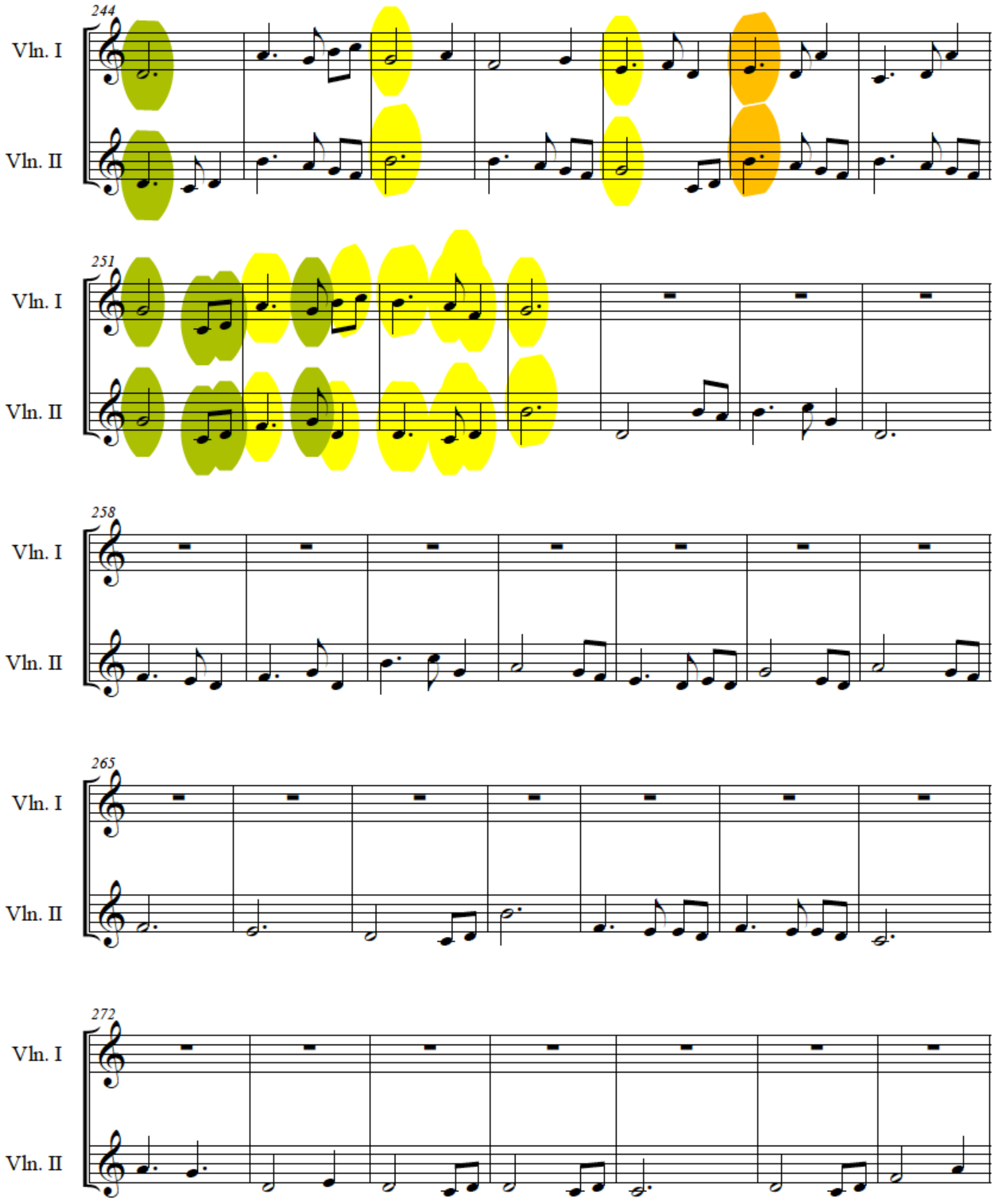
244
Vln. I
Vln. II
251
Vln. I
Vln. II
258
Vln. I
Vln. II
265
Vln. I
Vln. II
272
Vln. I
Vln. II

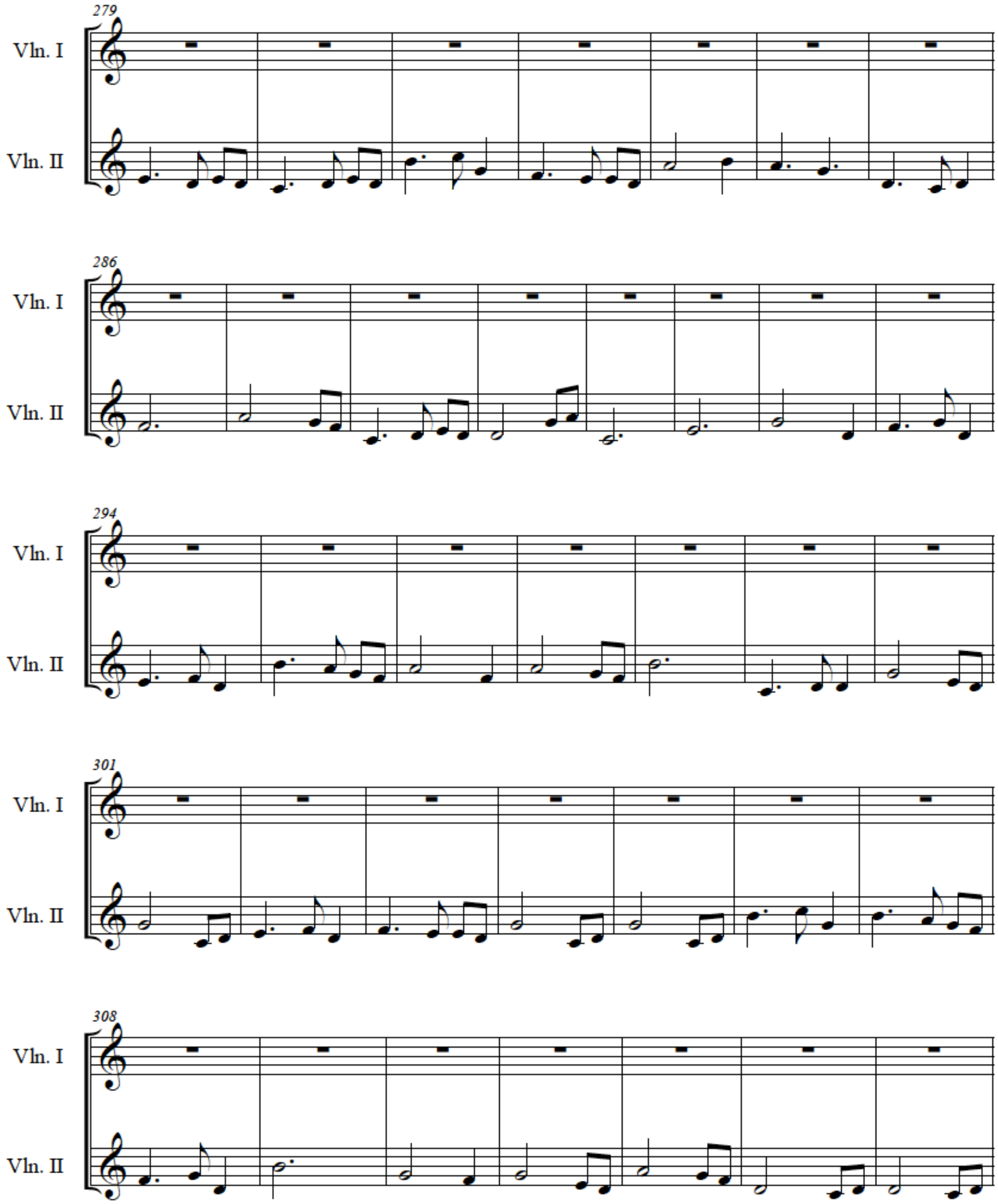
279
Vln. I
Vln. II
286
Vln. I
Vln. II
294
Vln. I
Vln. II
301
Vln. I
Vln. II
308
Vln. I
Vln. II

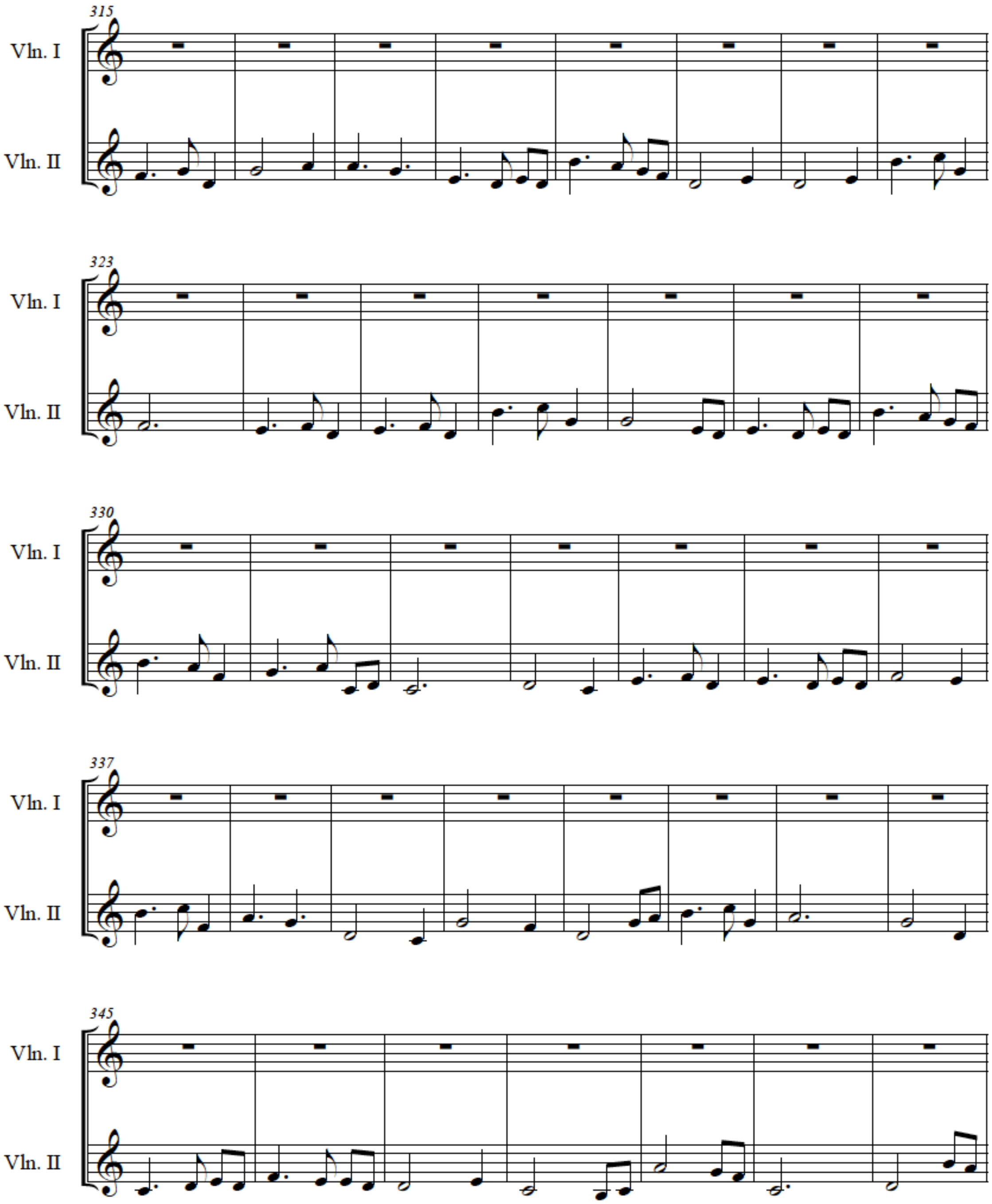
315
Vln. I
Vln. II
323
Vln. I
Vln. II
330
Vln. I
Vln. II
337
Vln. I
Vln. II
345
Vln. I
Vln. II

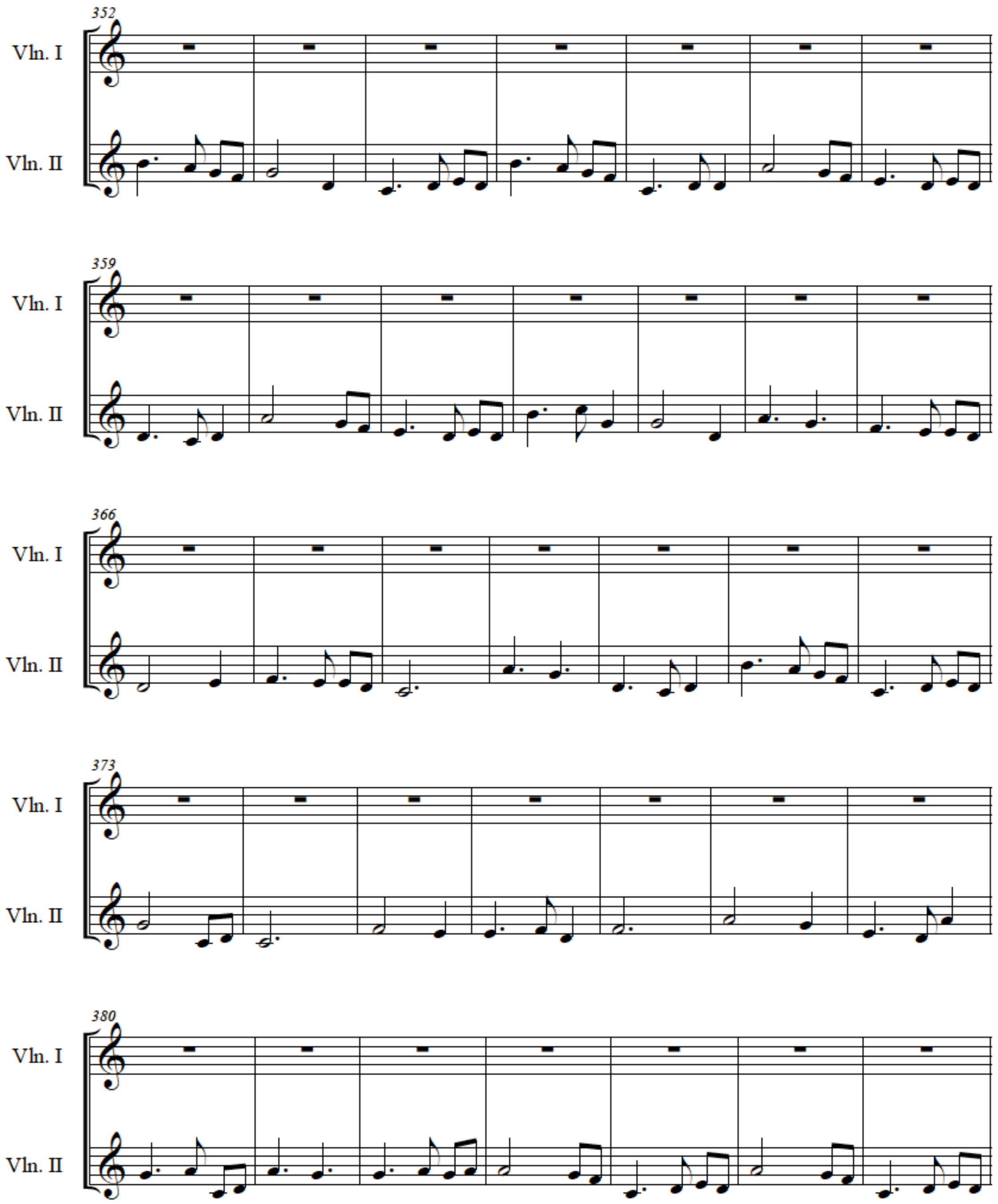
352
Vln. I
Vln. II
359
Vln. I
Vln. II
366
Vln. I
Vln. II
373
Vln. I
Vln. II
380
Vln. I
Vln. II

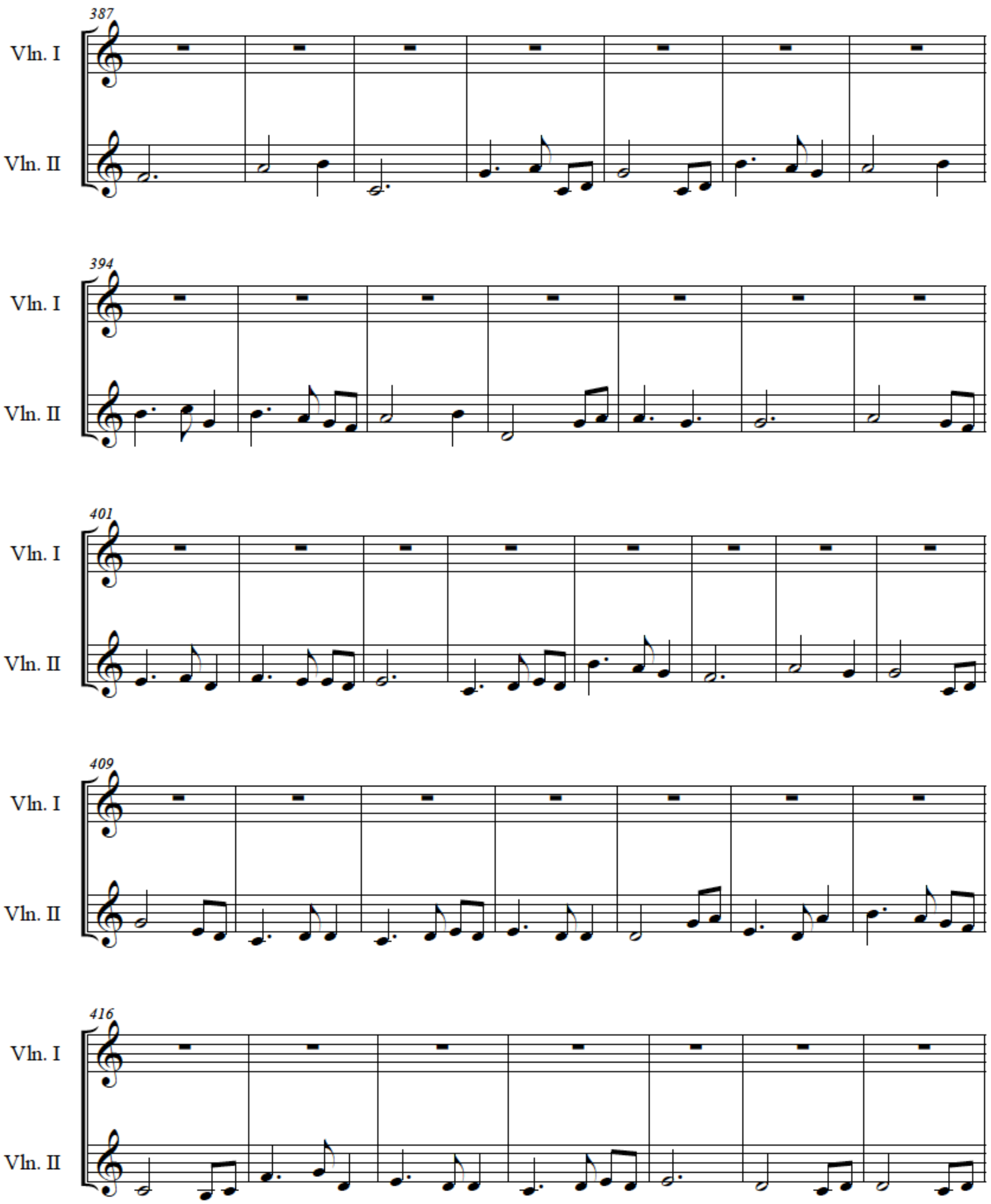
387
Vln. I
Vln. II
394
Vln. I
Vln. II
401
Vln. I
Vln. II
409
Vln. I
Vln. II
416
Vln. I
Vln. II

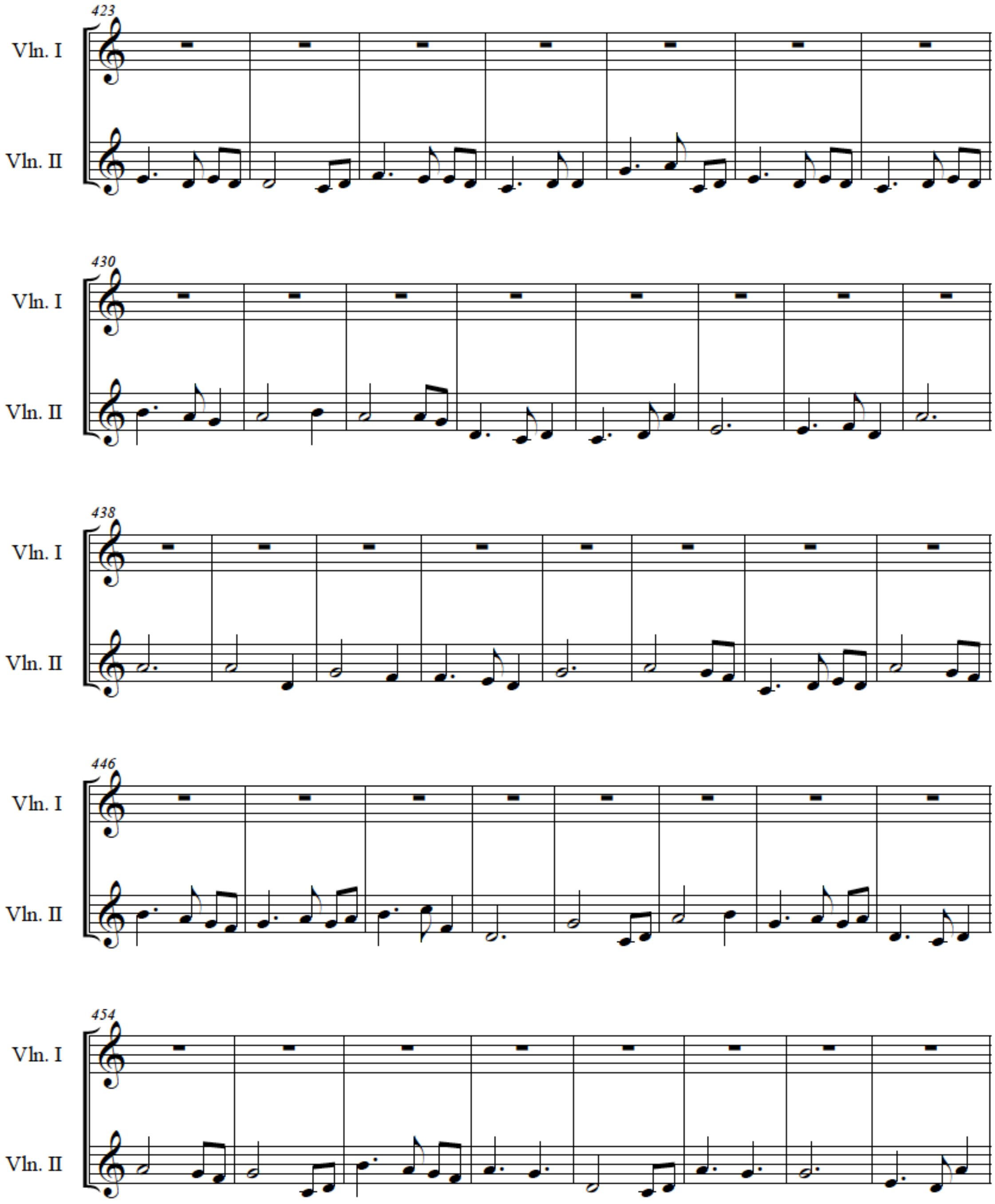
423
Vln. I
Vln. II
430
438
446
454

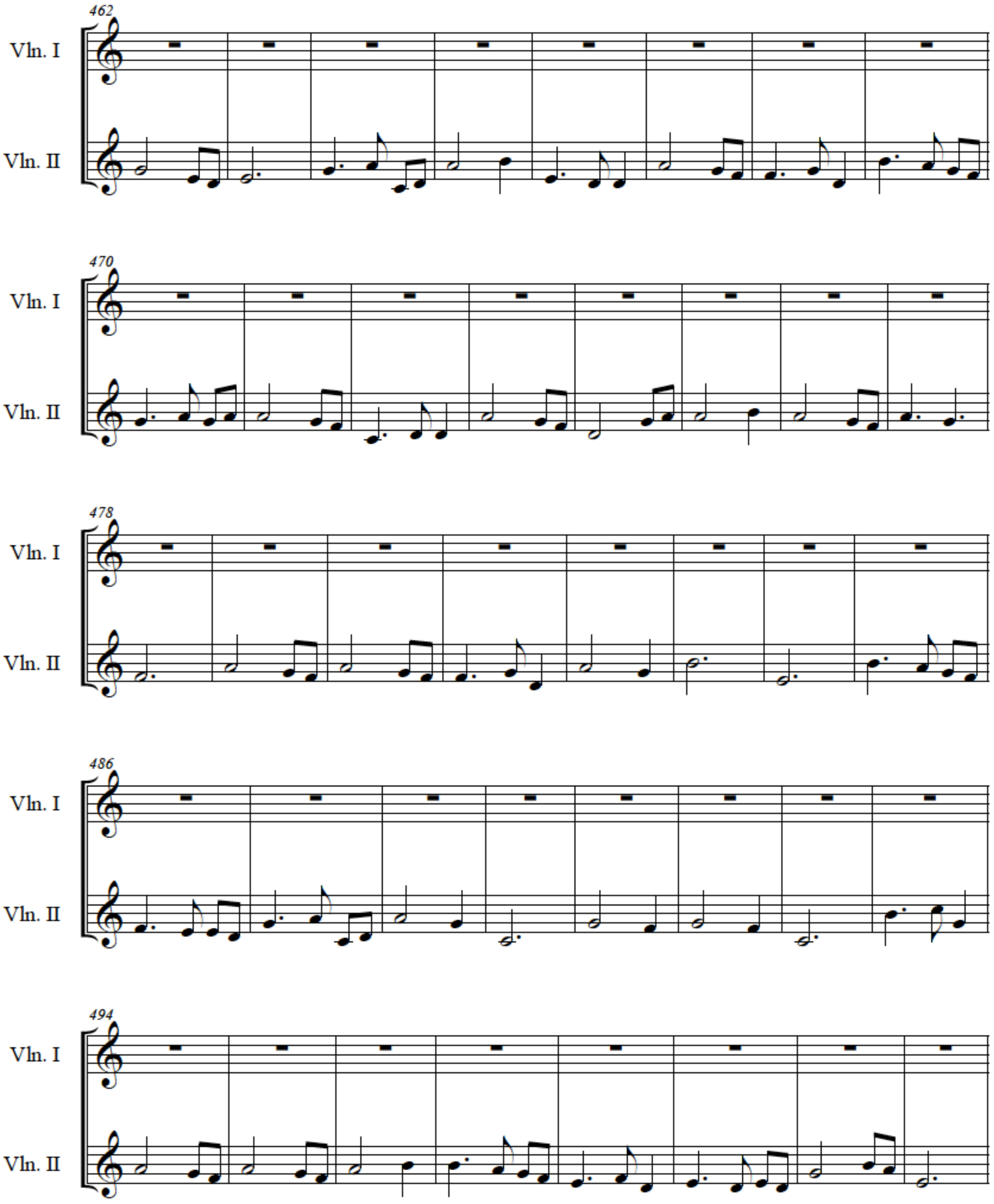
462
Vln. I
Vln. II
470
Vln. I
Vln. II
478
Vln. I
Vln. II
486
Vln. I
Vln. II
494
Vln. I
Vln. II

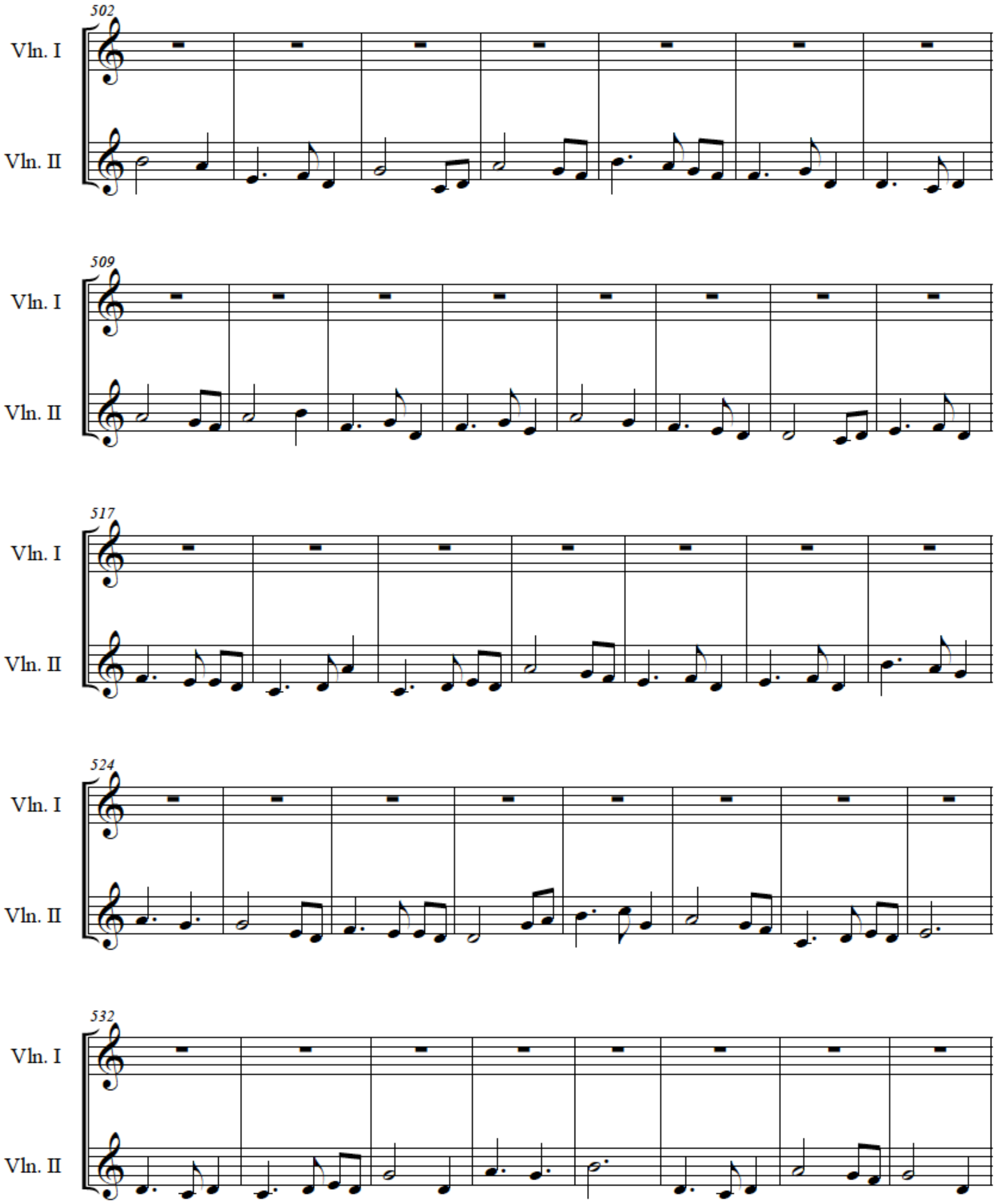
502
Vln. I
Vln. II
509
Vln. I
Vln. II
517
Vln. I
Vln. II
524
Vln. I
Vln. II
532
Vln. I
Vln. II

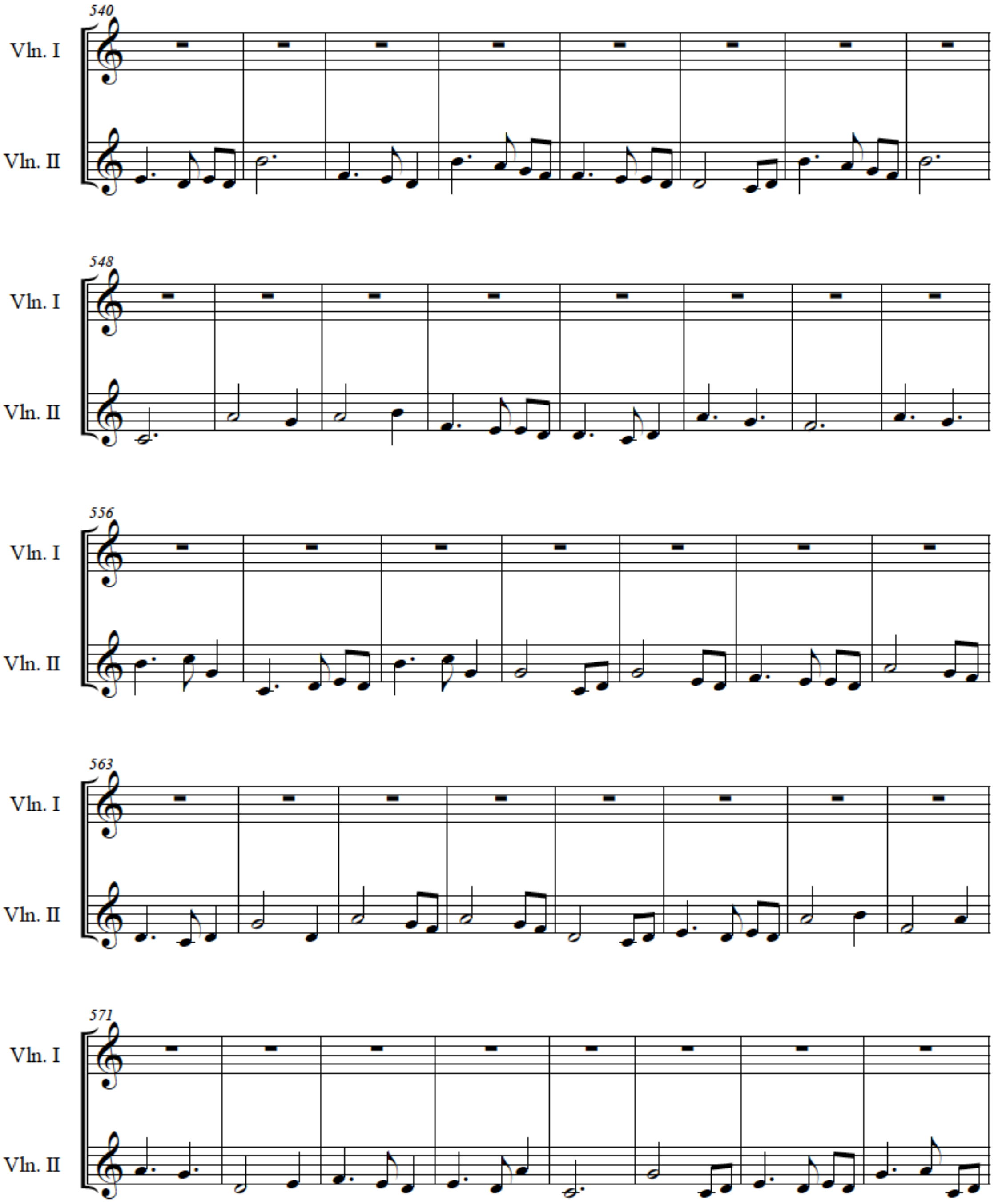
540
Vln. I
Vln. II
548
Vln. I
Vln. II
556
Vln. I
Vln. II
563
Vln. I
Vln. II
571
Vln. I
Vln. II

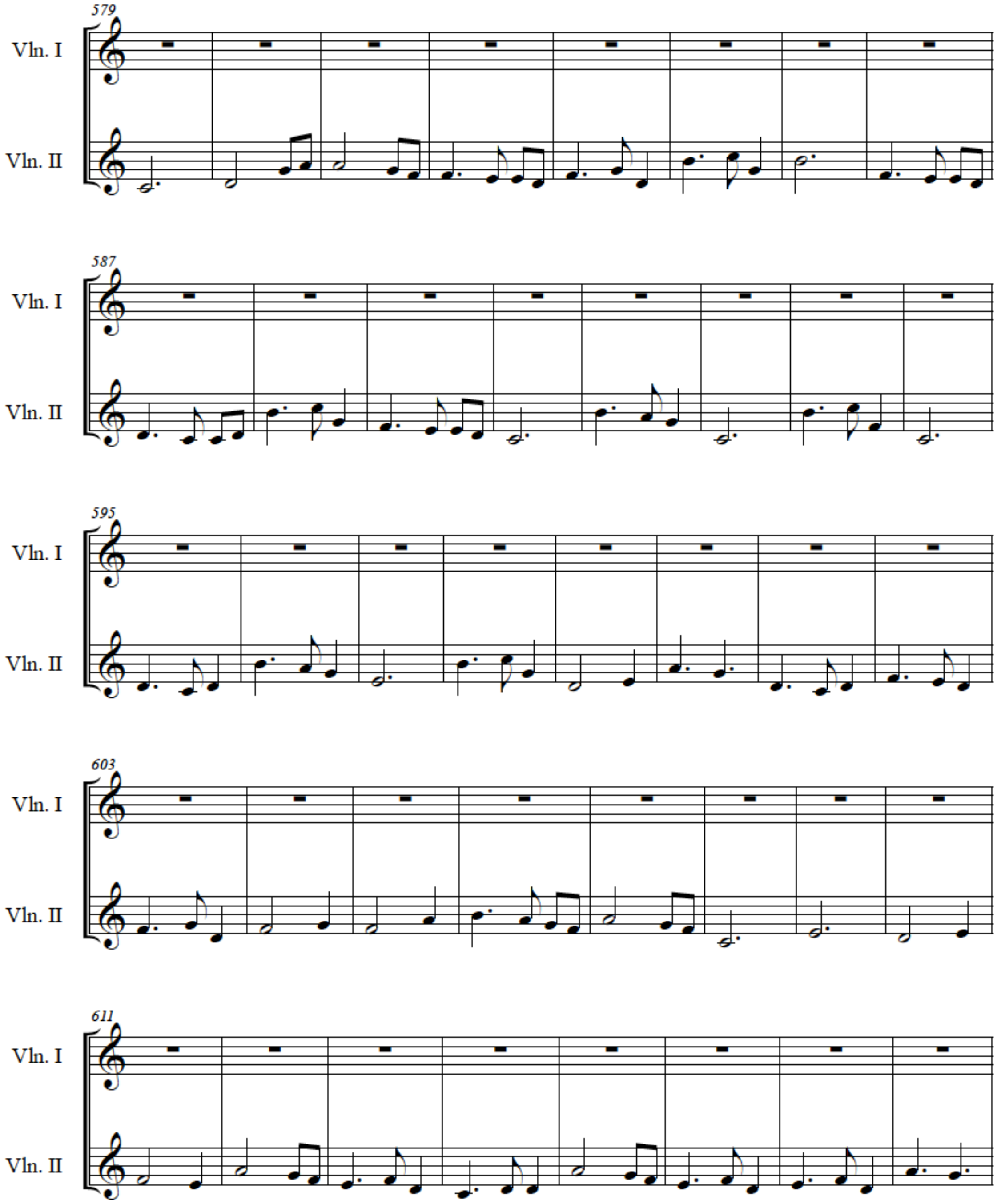
579
Vln. I
Vln. II
587
Vln. I
Vln. II
595
Vln. I
Vln. II
603
Vln. I
Vln. II
611
Vln. I
Vln. II

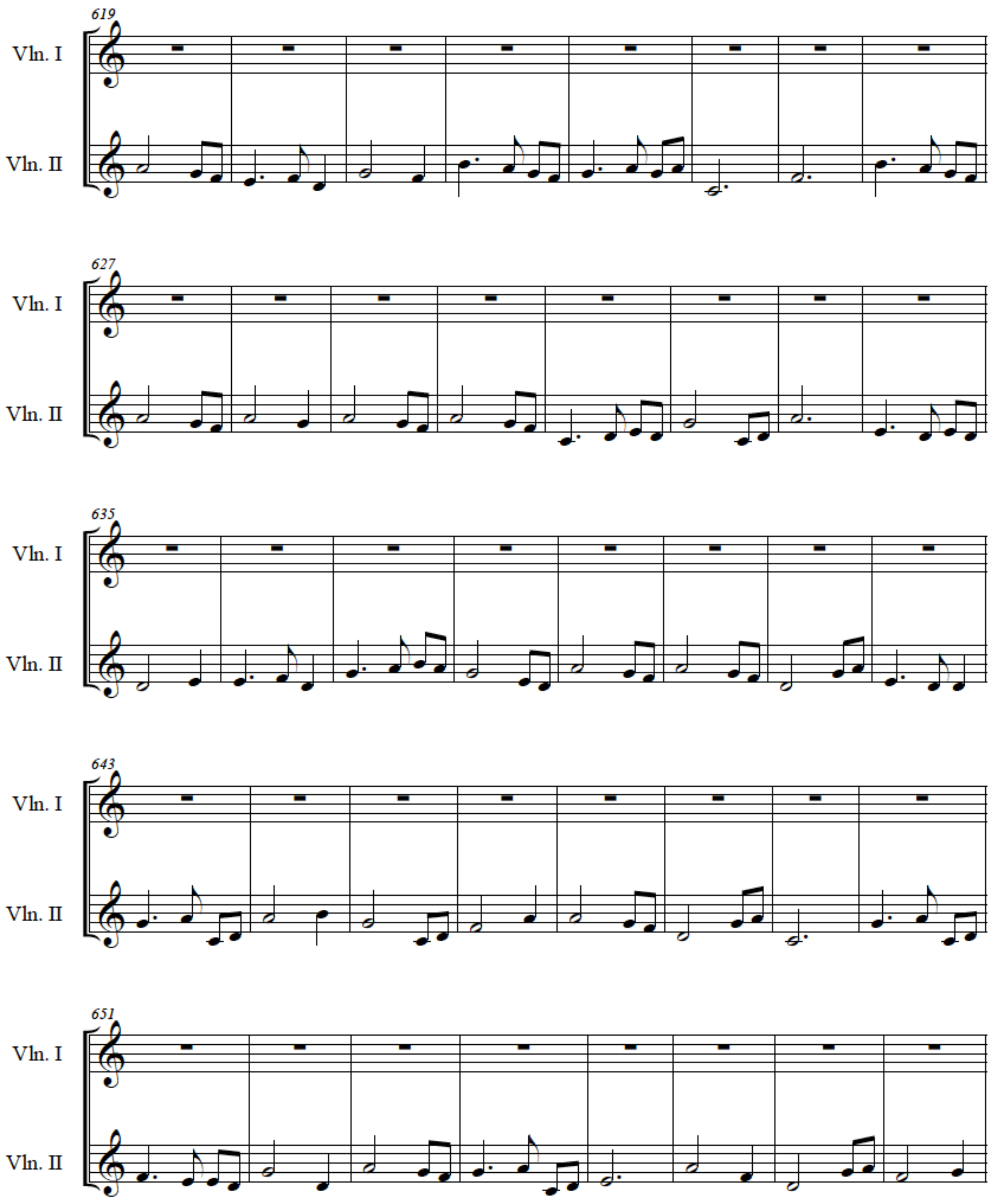
619
Vln. I
Vln. II
627
Vln. I
Vln. II
635
Vln. I
Vln. II
643
Vln. I
Vln. II
651
Vln. I
Vln. II

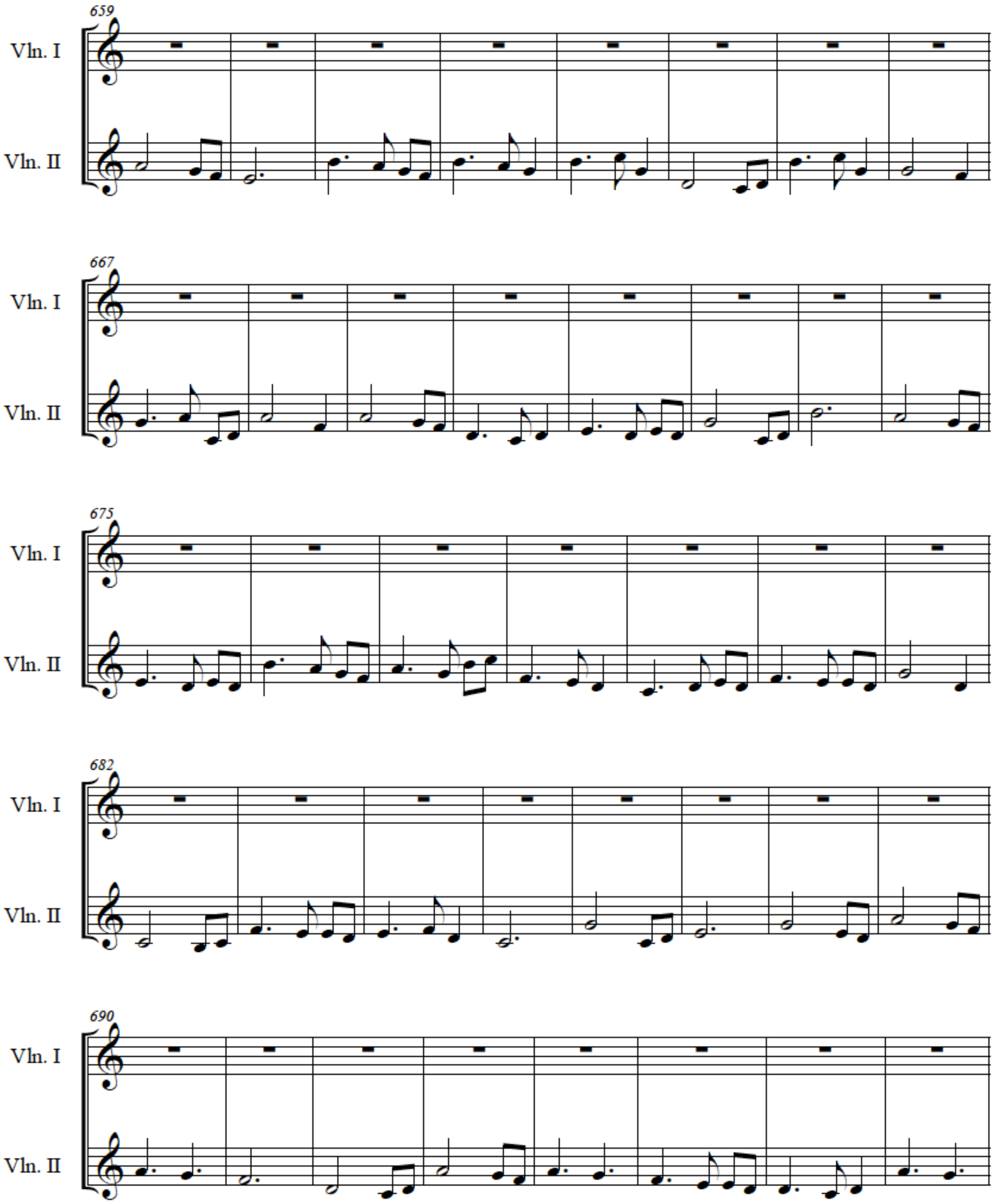
659
Vln. I
Vln. II
667
Vln. I
Vln. II
675
Vln. I
Vln. II
682
Vln. I
Vln. II
690
Vln. I
Vln. II

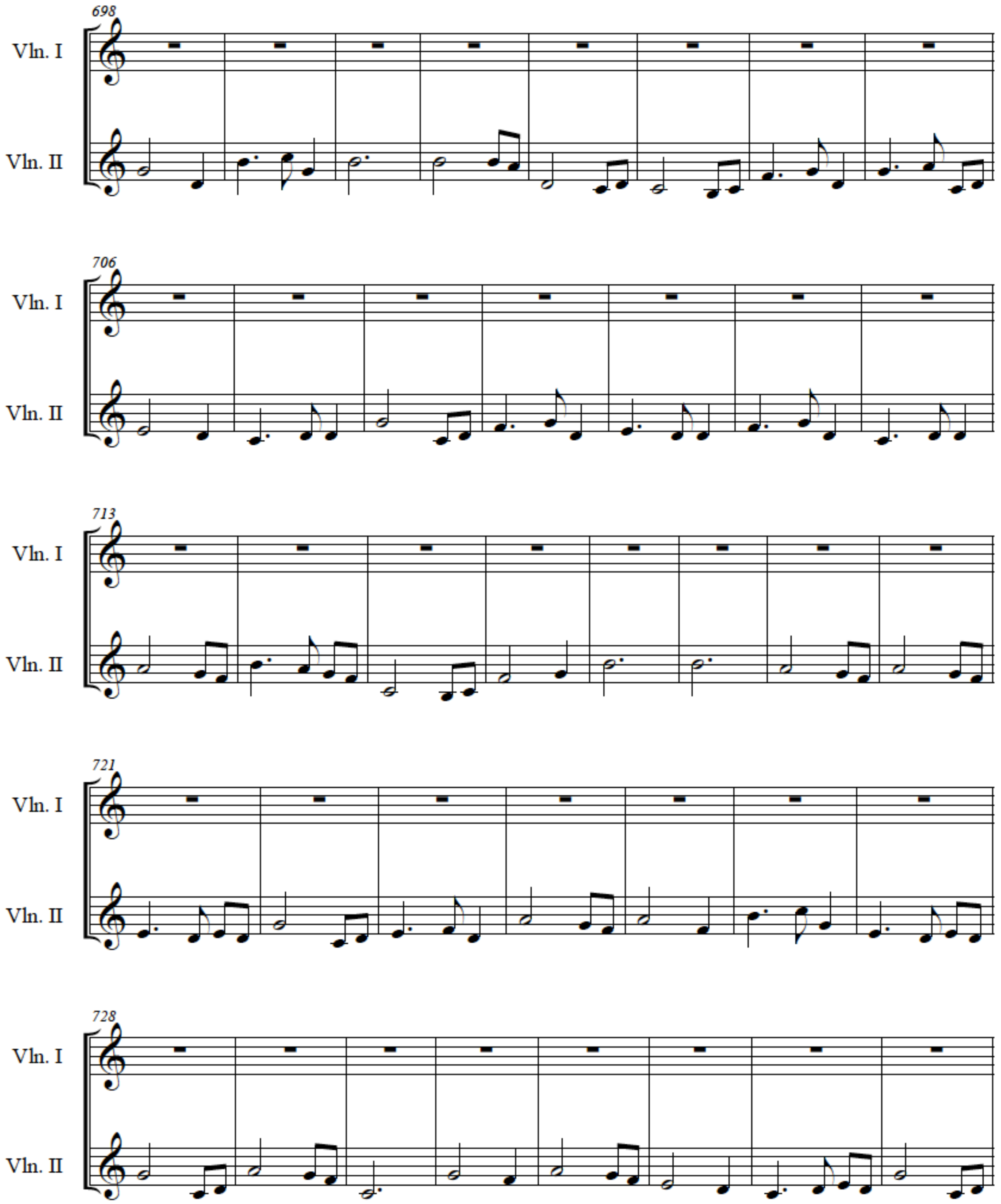
698
Vln. I
Vln. II
706
Vln. I
Vln. II
713
Vln. I
Vln. II
721
Vln. I
Vln. II
728
Vln. I
Vln. II

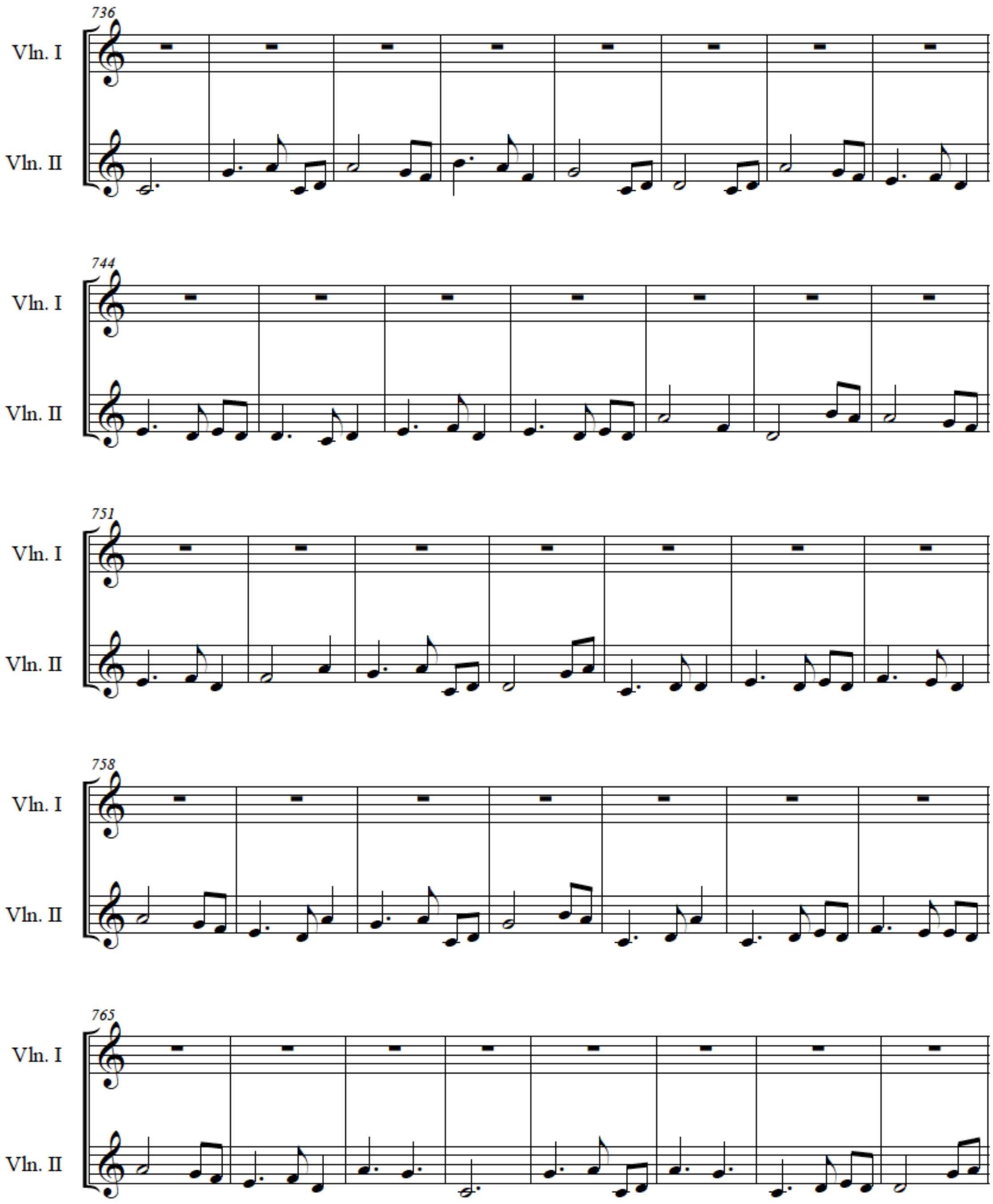
736
Vln. I
Vln. II
744
Vln. I
Vln. II
751
Vln. I
Vln. II
758
Vln. I
Vln. II
765
Vln. I
Vln. II

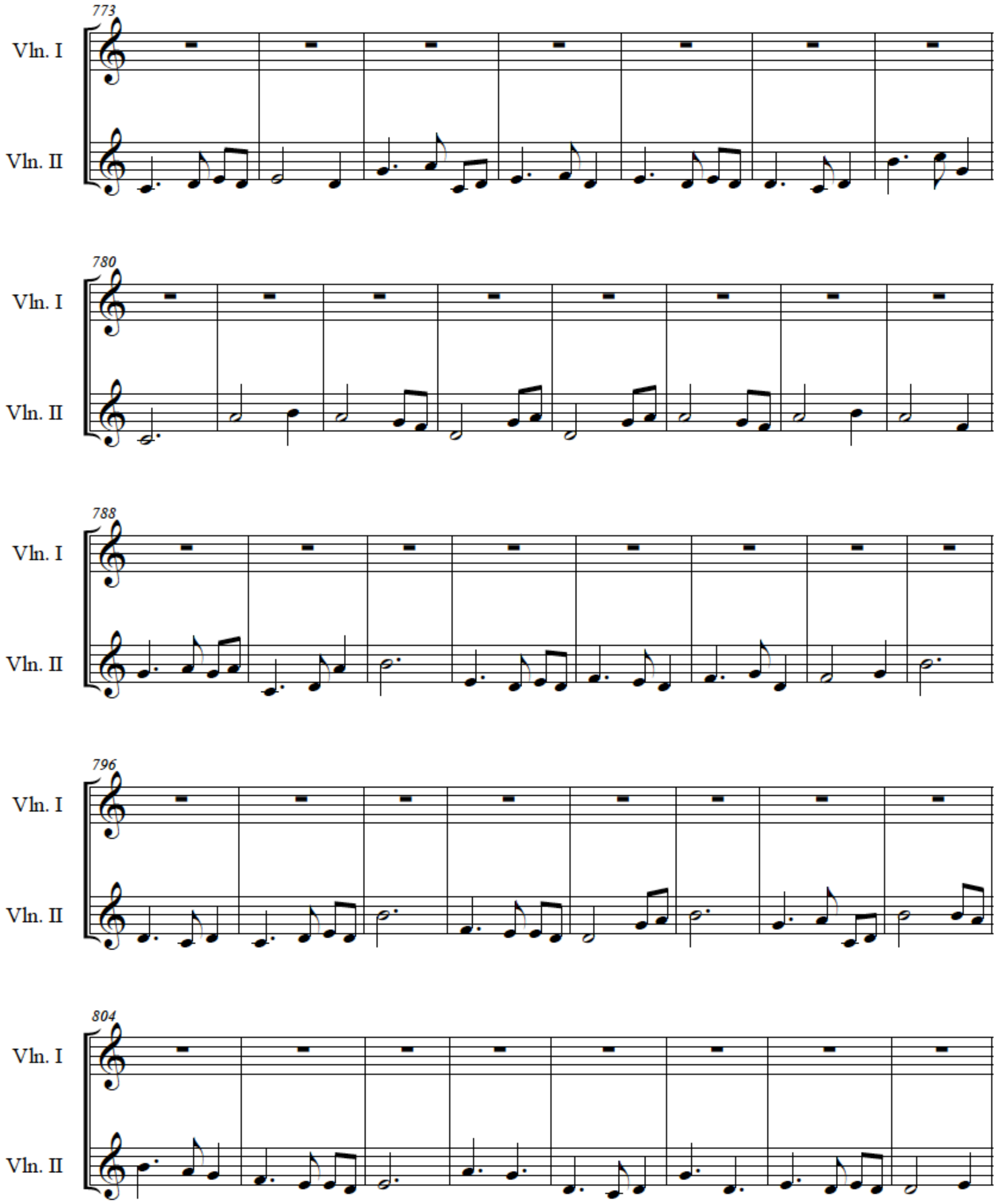
773
Vln. I
Vln. II
780
Vln. I
Vln. II
788
Vln. I
Vln. II
796
Vln. I
Vln. II
804
Vln. I
Vln. II

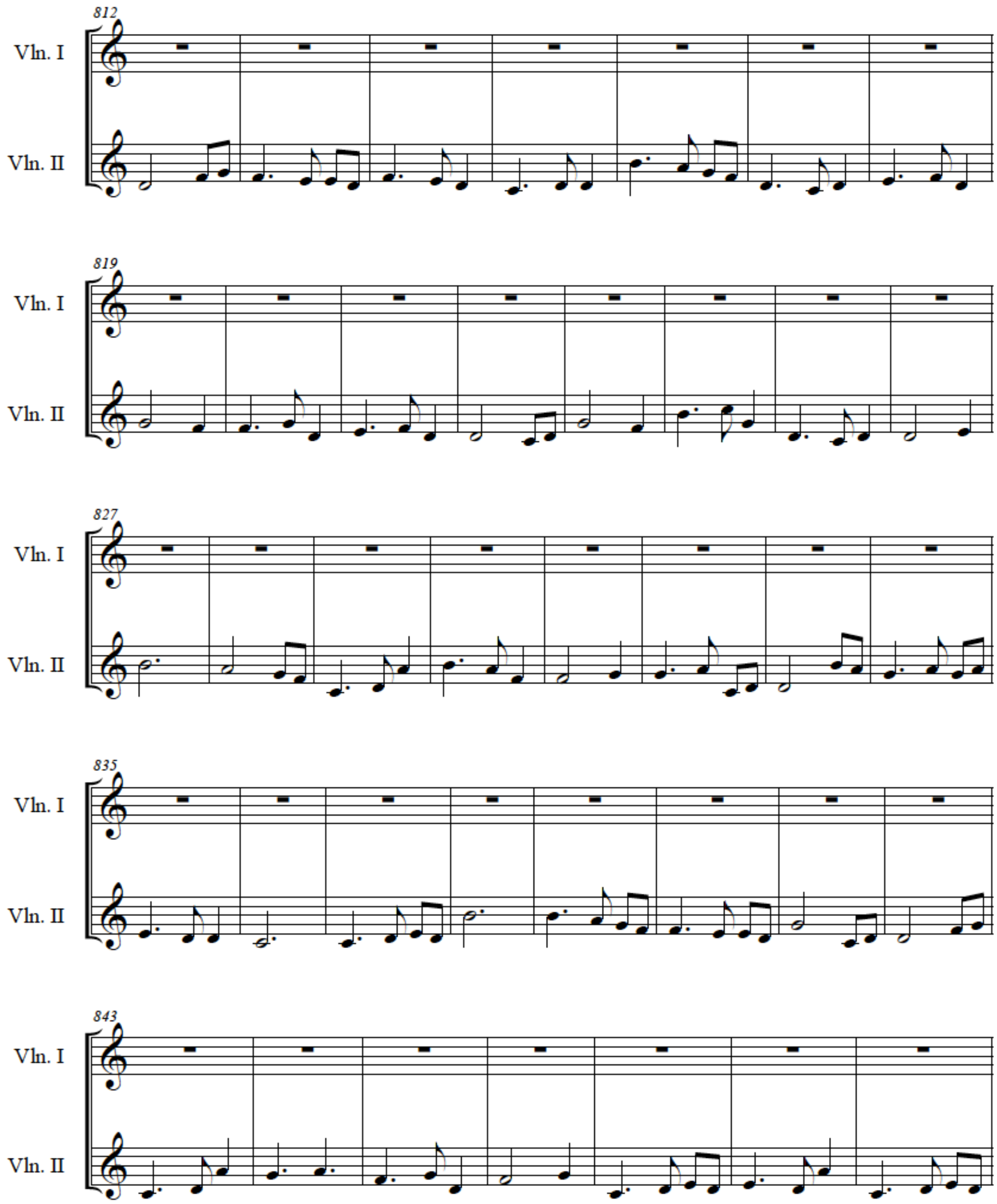
812
Vln. I
Vln. II
819
Vln. I
Vln. II
827
Vln. I
Vln. II
835
Vln. I
Vln. II
843
Vln. I
Vln. II

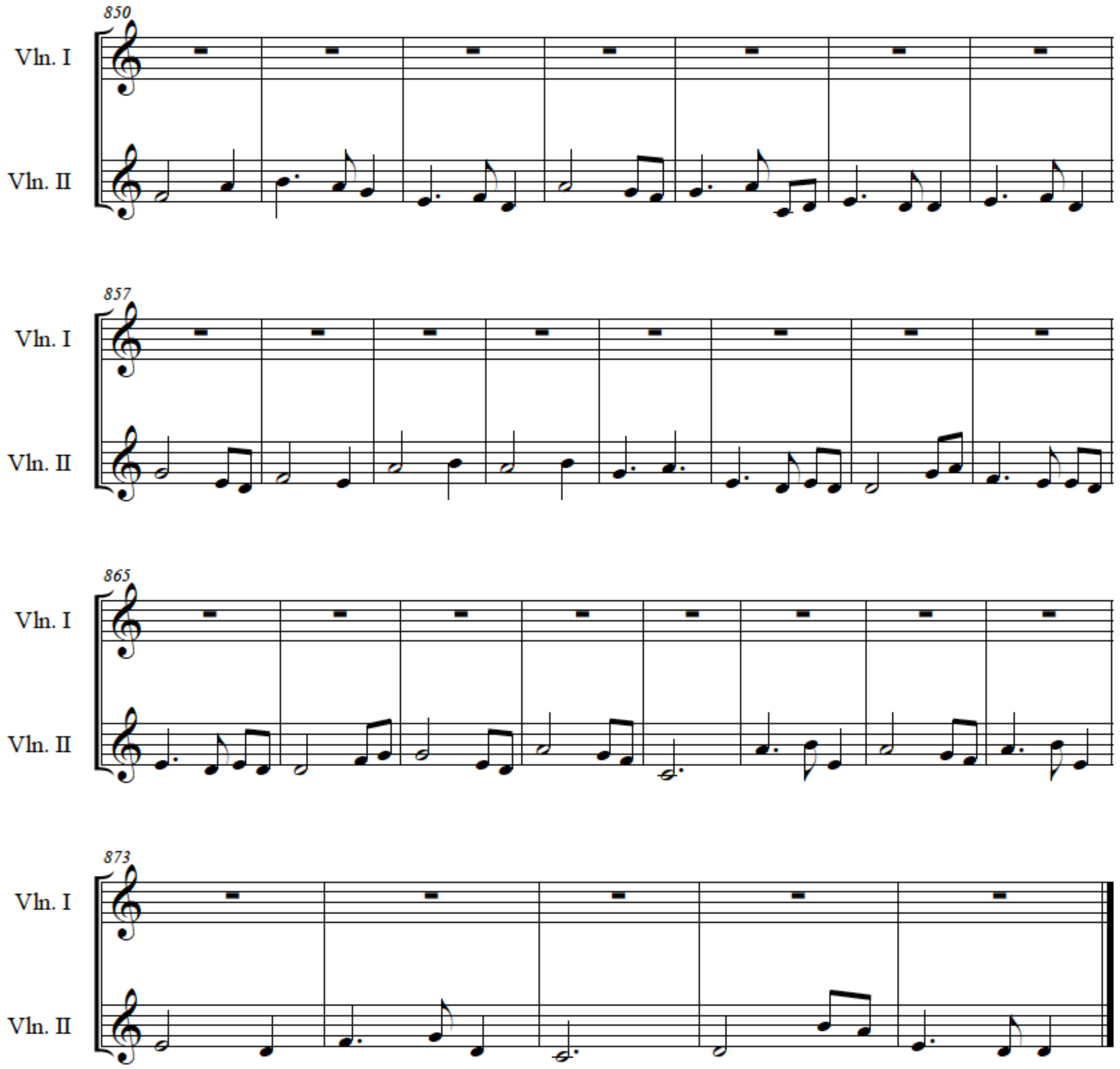

Fig. S4. Musical ensemble of AKR2-protein interactions. Musical scores for *H. sapiens* AKR2 and interacting proteins RNF10 or THRAP5 (Supplementary information, Fig. S1) were ensemble and analyzed for musical patterns.

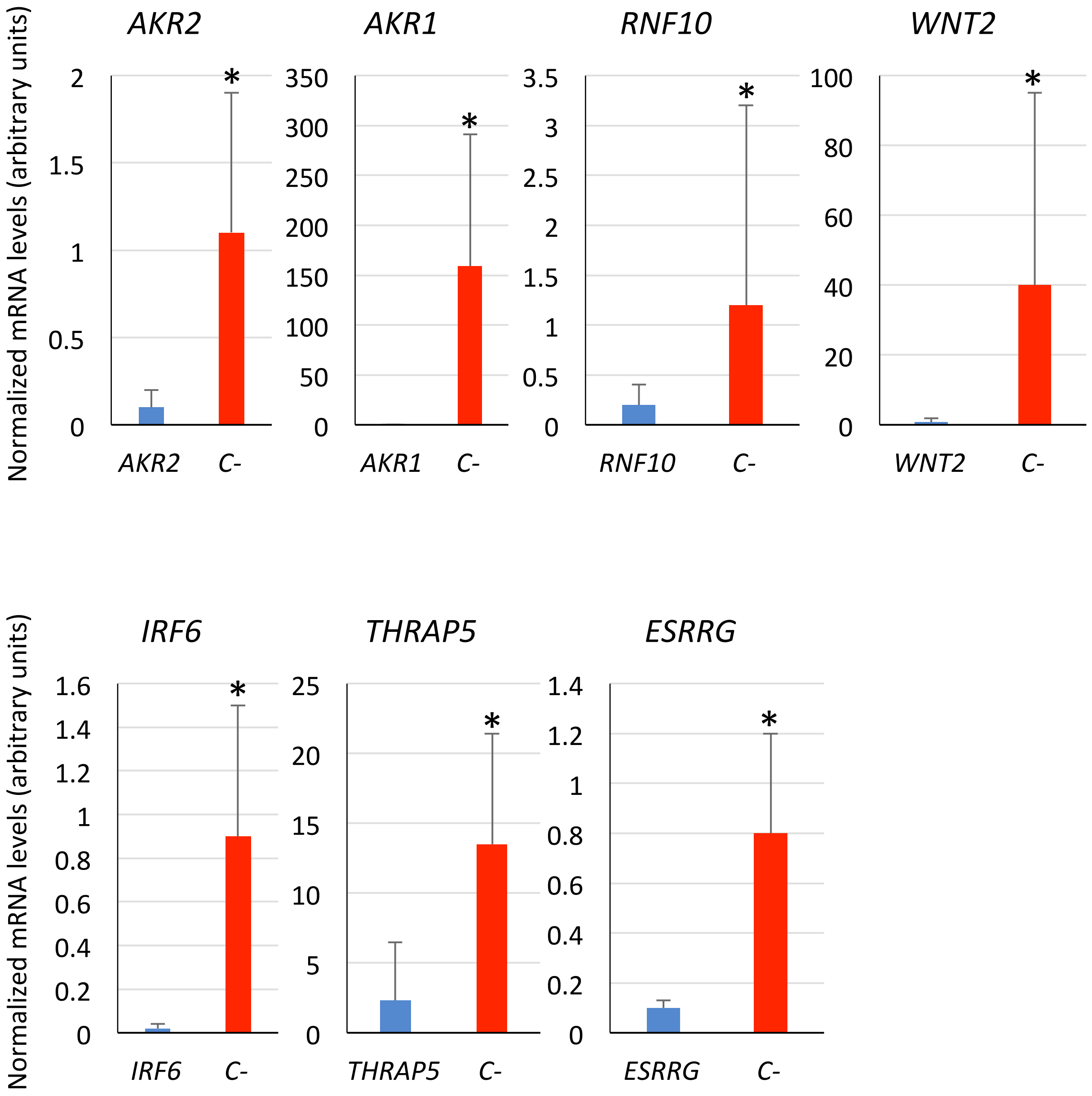

Figure S5. Gene knockdown by RNAi in human placenta cells. The mRNA levels were determined by qRT-PCR and normalized against human *β-actin* using the genNorm Delta-Delta-Ct (ddCt) method as described previously. Normalized Ct values were compared between test siRNAs-treated placenta cells and controls treated with non-targeting siRNA by Student's t test with unequal variance (*p < 0.01; N = 6). The gene knockdown for each gene with respect to the siRNA negative control was of 93±7% (*AKR2*), 100±0% (*AKR1*), 81±16% (*RNF10*), 98±3% (*WNT2*), 98±2% (*IRF6*), 83±31% (*THRAP5*) and 90±3% (*ESRRG*).

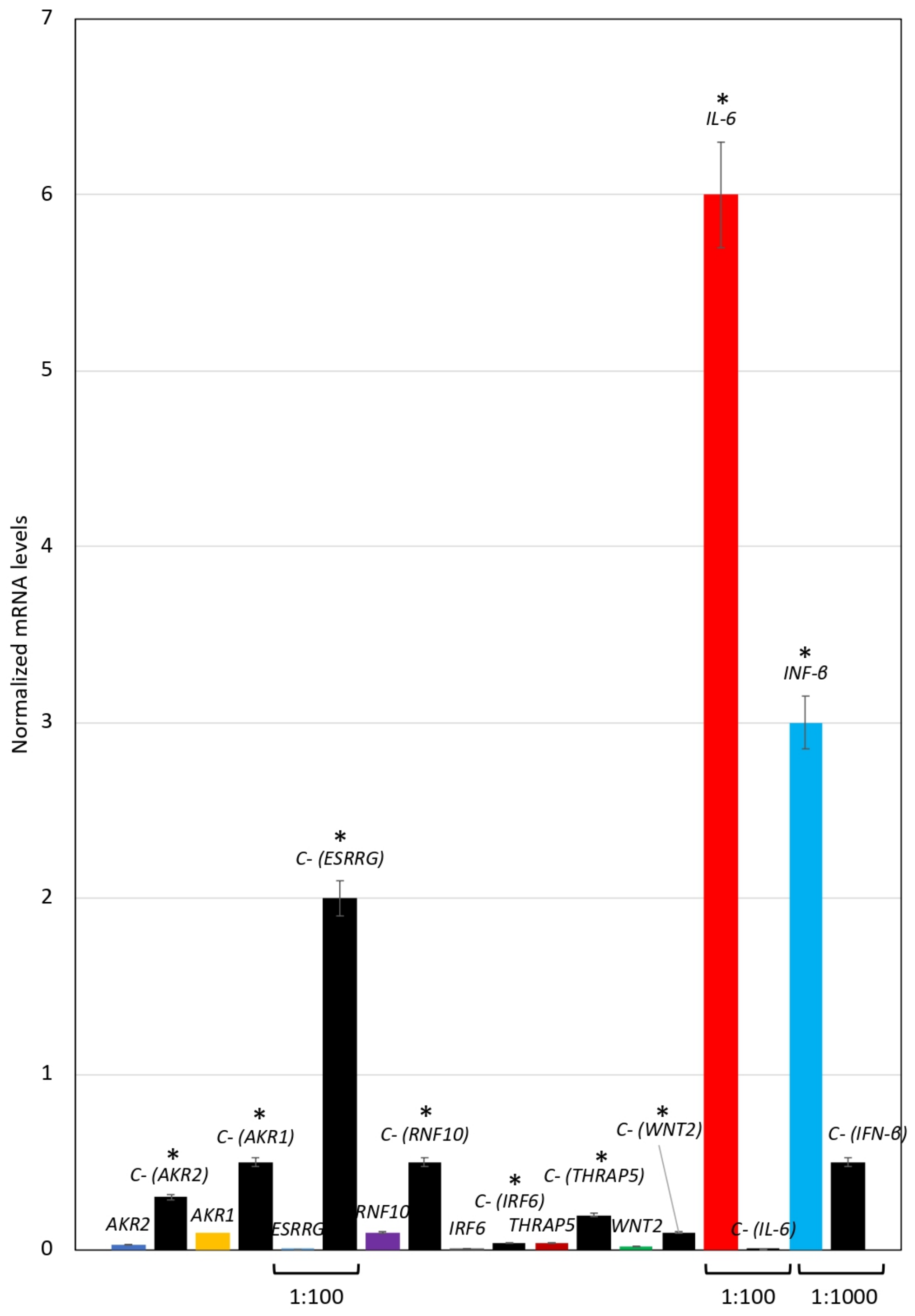

Figure S6. Gene expression in human placenta cells LPS-treated and controls. Human placenta cells were treated with 10 µg/ml of LPS from *Salmonella enterica* serotype typhimurium. LPS-treated and PBS-treated control cells were harvested after 24 h of treatment. The mRNA levels of *AKR2*, *AKR1*, *ESRRG*, *RNF10*, *THRAP5*, *IRF6*, *WNT2*, *IFN-β* and *IL-6* genes were determined by qRT-PCR and normalized against human *β-actin* using the genNorm Delta-Delta-Ct (ddCt) method as described previously. Normalized Ct values were compared between LPS-treated and PBS-treated control (C-) cells by Chi^2 test (*p < 0.01; N = 6 biological replicates). Values are shown 1 in 100 for *ESRRG* and *IL-6*, and 1 in 1000 for *IFN-β*.
